# Site-specific molecular glues for the 14-3-3/Tau pS214 protein–protein interaction *via* reversible covalent imine tethering†

**DOI:** 10.1039/d4md00833b

**Published:** 2025-02-07

**Authors:** Ansgar Oberheide, Maxime C. M. van den Oetelaar, Jakob J. A. Scheele, Jan Borggräfe, Semmy F. H. Engelen, Michael Sattler, Christian Ottmann, Peter J. Cossar, Luc Brunsveld

**Affiliations:** a Laboratory of Chemical Biology, Department of Biomedical Engineering and Institute for Complex Molecular Systems, Eindhoven University of Technology Groene Loper 3 5612 AE Eindhoven The Netherlands c.ottmann@tue.nl l.brunsveld@tue.nl; b Helmholtz Munich, Molecular Targets and Therapeutics Center, Institute of Structural Biology Ingolstädter Landstrasse 1 85764 Neuherberg Germany; c Technical University of Munich, TUM School of Natural Sciences, Bavarian NMR Center and Department of Bioscience Lichtenbergstrasse 4 85747 Garching Germany

## Abstract

Protein–protein interactions (PPIs) are key regulators of various cellular processes. Modulating PPIs with small molecules has gained increasing attention in drug discovery, particularly targeting the 14-3-3 protein family, which interacts with several hundred client proteins and plays a central role in cellular networks. However, targeting a specific PPI of the hub protein 14-3-3, with its plethora of potential client proteins, poses a significant selectivity challenge. This not only involves the selectivity of 14-3-3 PPIs with other client proteins, but also the selective stabilization of a specific 14-3-3 binding site within a protein partner featuring several binding sites. The interaction of 14-3-3 with Tau, characterized by different phospho-site driven binding modes, forms a valuable, disease-relevant, 14-3-3 multivalent model PPI to explore this selectivity issue. This work presents the identification and early-stage optimization of small molecule fragment-like stabilizers for a specific binding site of the 14-3-3/Tau PPI. Using different biophysical assays, the stabilizing potency of the imine-bond forming molecules was mapped and X-ray crystallography studies provided structural data on the binding mode of the ternary complexes. Exploiting the unique topologies and functionalities of the different binding sites enabled the engineering of selectivity for this initial molecular glue matter for the pS214 binding site, over a second 14-3-3 binding site in Tau (pS324). These reversible covalent tool compounds will allow for the further exploration of the role of 14-3-3 in Tau aggregation.

## Abstract 2

The unique molecular topologies of the two different binding sites of the Tau/14-3-3 protein–protein interaction were exploited to develop selective dynamic covalent molecular glues for the Tau pS214 site.

## Graphical abstract

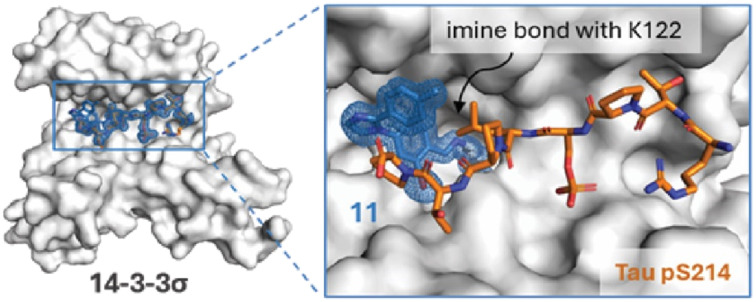

## Introduction

Many physiological and pathological processes in living cells and tissues are regulated *via* protein–protein interactions (PPIs).^1–3^ The modulation of PPIs using small molecular ligands has received increasing attention as a potential therapeutic intervention in drug discovery.^3–6^ The 14-3-3 protein family is an interesting target for studying PPI modulation since, with several hundred described client proteins, 14-3-3 plays a crucial role in cellular PPI networks.^7,8^ The 14-3-3 hub-proteins are a family of eukaryotic, highly conserved proteins of which seven homologous mammalian isoforms are known (σ, ζ, β, γ, η, ε, and τ). They mainly exist as homo- and heterodimers and typically bind to a phosphorylated serine/threonine binding motif of the client protein. By binding to their client proteins, 14-3-3 proteins regulate different biological processes, such as signal transduction, protein trafficking, apoptosis, and cell cycle control.^7,8^ Stabilization of 14-3-3 PPIs with molecular glues, as opposed to their inhibition, is typically the desired mode of pharmacological intervention.^7–10^ However, stabilizing a specific PPI within the vast network of 14-3-3, with its hundreds of client proteins, poses a significant selectivity challenge. This not only entails selectivity over 14-3-3 PPIs with other partner proteins, but also the selective stabilization of a specific 14-3-3 binding site from a client protein that has two or more 14-3-3 interactions motifs. Such selective molecular glue compounds would be of interest both as chemical tool compounds and as informative strategies for drug discovery.^11^

**Fig. 1:**
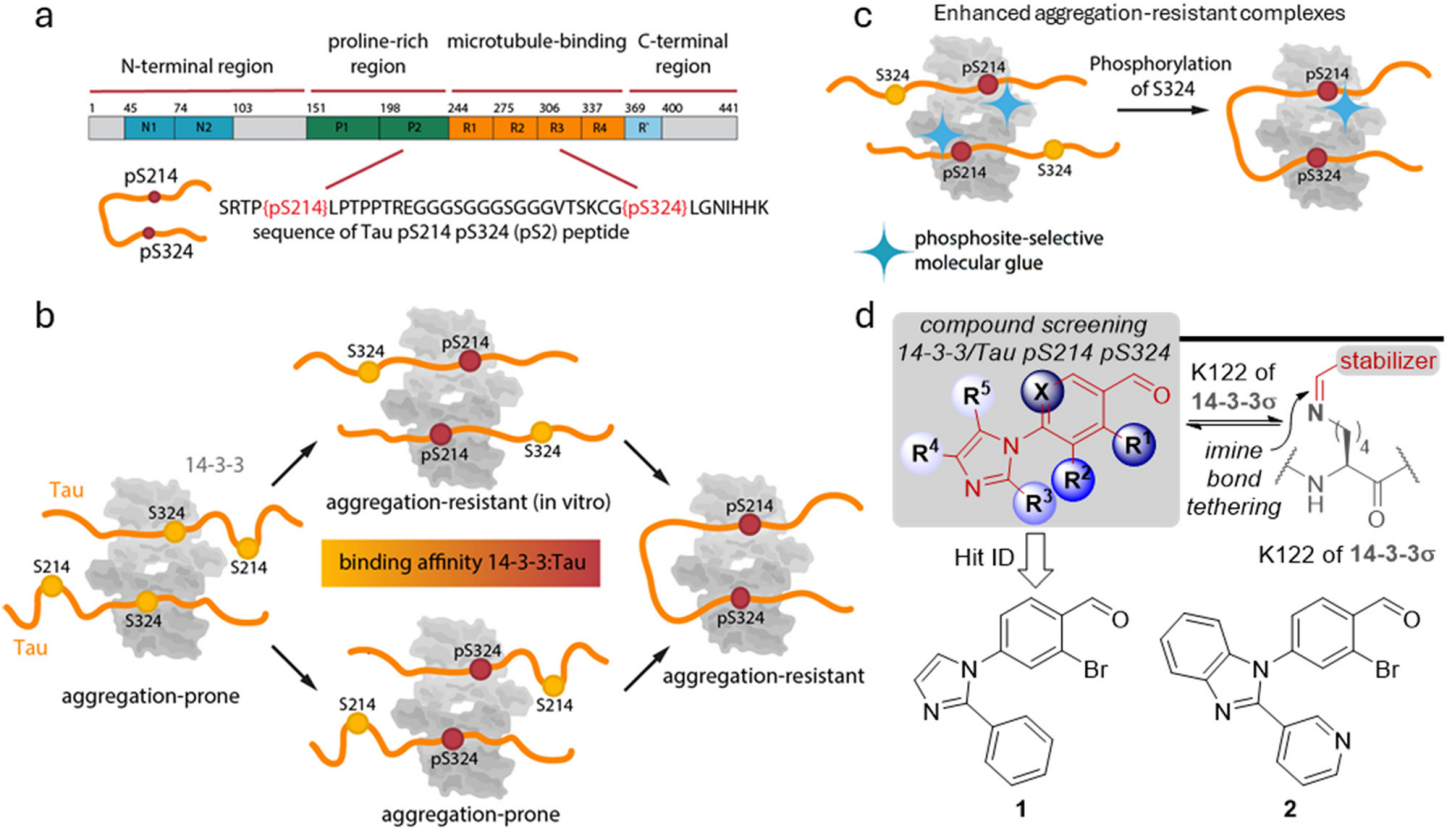
a Primary structure and domains of Tau protein, as well as the sequence of the bivalent Tau pS214–pS324 (pS2) peptide. b Schematic visualization of the different 14-3-3/Tau binding modes depending on the phosphorylation state of Tau with the corresponding binding affinity and aggregation propensity with a focus on the phosphorylation sites S214 and S324. Tau phosphorylated at only S214 or S324 leads analogously as the non-phosphorylated Tau to the formation of a 2 : 2 complex (14-3-3 monomer : Tau) instead of a 1 : 2 complex in case of bivalent Tau. The complex in which either both S214 and S324 are phosphorylated as well as the complex in which only S214 is phosphorylated is expected to be aggregation-resistant, while the other complexes have been reported to induce aggregation. Figure adapted from Chen *et al.*^17^ c Schematic visualization of a phosphosite-selective molecular glue to stabilize the 14-3-3/Tau pS214 interaction and potential to convey aggregation-resistance. d General chemotype used in the screening campaign to identify stabilizers of the 14-3-3/Tau pS2 peptide interaction and chemical structures of hit compound 1 and 2.

14-3-3 proteins are highly expressed in the central nervous system, making up about 1% of all soluble proteins.^12^ 14-3-3ζ and 14-3-3γ were found to be most abundantly expressed in the brain.^13,14^ 14-3-3 proteins were reported to play an important role in neurodegenerative diseases, by binding and thereby modulating several proteins that aggregate in these diseases.^15^ This chaperone-like function of 14-3-3 has been reported^16^ for the microtubule-associated protein Tau, a key protein in Alzheimer's disease (AD) and frontotemporal dementia. The mode of action how 14-3-3 contributes to the homeostasis of Tau aggregation demands further investigation.^17–19^ Tau, an intrinsically disordered protein (IDP) that is mainly found in neurons, is a microtubule-associated protein that binds to and thereby stabilizes the microtubules under physiological conditions.^20–23^ Tau is mainly regulated *via* phosphorylation, inducing the dissociation from the microtubules, with over 80 putative phosphorylation sites reported for Tau (Fig. 1a).^24,25^

14-3-3 has been shown to bind (multivalently) to two phosphorylated serine binding motifs of Tau, surrounding S214 and S324.^17,19,20,26^ Soluble phosphorylated Tau is highly abundant in healthy human brain.^27–29^ Under pathological conditions, Tau is hyperphosphorylated and can start to accumulate, forming toxic neurofibrillary tangles (NFTs) in the cytosol.^20–23^ Previous studies have implied different roles for the phosphorylated Tau S214 and S324 sites and their interactions with 14-3-3 regarding Tau aggregation (Fig. 1b).^17,23^ Especially binding of 14-3-3 to Tau phosphorylated at S214 was demonstrated to inhibit Tau aggregation and condensation.^16,30^ This makes the resulting PPI interface with 14-3-3 an interesting case study for exploring the concept of development of site-specific 14-3-3 molecular glues (Fig. 1c). Here, we report the identification of initial chemical matter, specifically dynamic covalent small molecule stabilizers, for the Tau pS214 binding site with 14-3-3.

## Results and discussion

### Structural analysis of 14-3-3/Tau interaction and stabilization by compound 1

Two phosphorylated serine residues of Tau have been reported to be crucial for 14-3-3 binding affinity, pS214 and pS324.^19^ In a fluorescence anisotropy (FA) assay, both monovalent peptides Tau pS214 and Tau pS324 show relatively weak binding affinities to, the brain relevant, 14-3-3ζ with *K*_D_ values of >100 μM, while the bivalent Tau pS2 peptide has a significantly enhanced 14-3-3ζ binding affinity of 945 ± 70 nM (Fig. 1a).^16^

With these initial data in hand, we were prompted to identify small molecules that stabilized the 14-3-3ζ/Tau pS2 peptide interaction by screening an in-house focused library of imidazol-1-yl-benzaldehydes.^31^ In previous work, this chemotype has been established to efficiently stabilize the interaction of 14-3-3 with Pin1. The interaction and its stabilization was driven by non-covalent, cooperative molecular recognition and strengthened by an imine-bond formation between the benzaldehyde moiety and K122 residue of 14-3-3σ (Fig. 1d).^31^ In our screening campaign, hit compounds 1 and 2 were identified to stabilize the 14-3-3ζ/Tau pS2 peptide complex by 23- and 20-fold, respectively (ESI† Fig. S1 and S2, Table 1).

**Table 1:** Chemical structures of series 1 and 3, quantified CC_50_, apparent *K*^app^_D(250μM)_ and stabilization factor (SF_250μM_) values of compounds based on compound and protein titration curves profiling the 14-3-3ζ/Tau pS2 peptide interaction. SFs were calculated based on the internal DMSO reference. Mean and SD for compounds 1–23 are based on scientific duplicates, mean and SD for compounds 25–31 are based on technical duplicates

Compound	Series	R^1^	R^2^	R^3^	X	CC_50_ (μM)	Apparent *K*_D_ (nM)	SF_250μM_
1	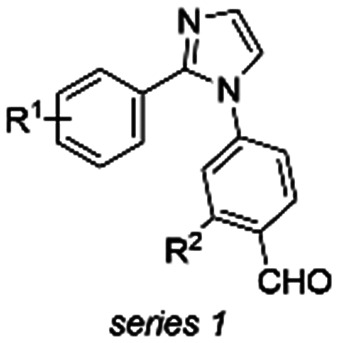	H	Br	—	—	122 ± 57	40 ± 6	23
3		*o*-Me	Br	—	—	149 ± 64	83 ± 4	11
4		*o*-OMe	Br	—	—	—	—	—
5		*o*-Cl	Br	—	—	105 ± 5	141 ± 47	7.5
6		*m*-Me	Br	—	—	160 ± 110	94 ± 12	10
7		*m*-OMe	Br	—	—	—	—	—
8		*m*-Cl	Br	—	—	—	—	—
9		*m*-OH	Br	—	—	—	—	—
10		*m*-C(=O)NH_2_	Br	—	—	—	—	—
11		*p*-Me	Br	—	—	135 ± 78	32 ± 4	29
12		*p*-OMe	Br	—	—	—	—	—
13		*p*-F	Br	—	—	71 ± 19	22 ± 1	33
14		*p*-Cl	Br	—	—	69 ± 14	84 ± 20	8.9
15		*p*-I	Br	—	—	23 ± 5	245 ± 65	3.1
16		*p*-OH	Br	—	—	—	—	—
17		*p*-C(=O)NH_2_	Br	—	—	—	—	—
18		*p*-Et	Br	—	—	15 ± 5	31 ± 8	37
19		*p*-iPr	Br	—	—	—	—	—
20		*p*-CF_3_	Br	—	—	65 ± 5	86 ± 4	13
21		*p*-OCF_3_	Br	—	—	75 ± 7	103 ± 3	11
22		*p*-CN	Br	—	—	46 ± 39	44 ± 24	34
23		*p*-Morpholino	Br	—	—	68 ± 25	70 ± 0.1	16
24		*p*-O(CH_2_)_2_OH	Br	—	—	—	—	—
25		H	Cl	—	—	31 ± 49	69 ± 6	15
26		H	I	—	—	n.d.	594 ± 120	1.8
27		H	CF_3_	—	—	n.d.	175 ± 57	6.1
2	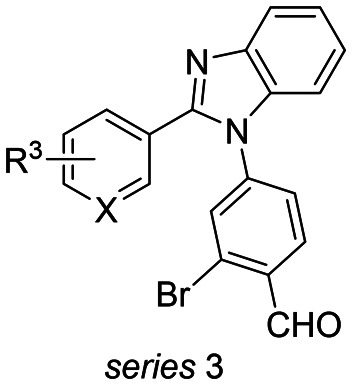	—	—	H	N	85 ± 36	45 ± 11	20
28		—	—	*m*-OMe	C	—	—	—
29		—	—	*p*-OMe	CH	26 ± 3	63 ± 21	8.3
30		—	—	*p*-OMe	N	—	—	
31		—	—	*p*-O(CH_2_)_2_OH	CH	24 ± 9	27 ± 16	19

**Fig. 2:**
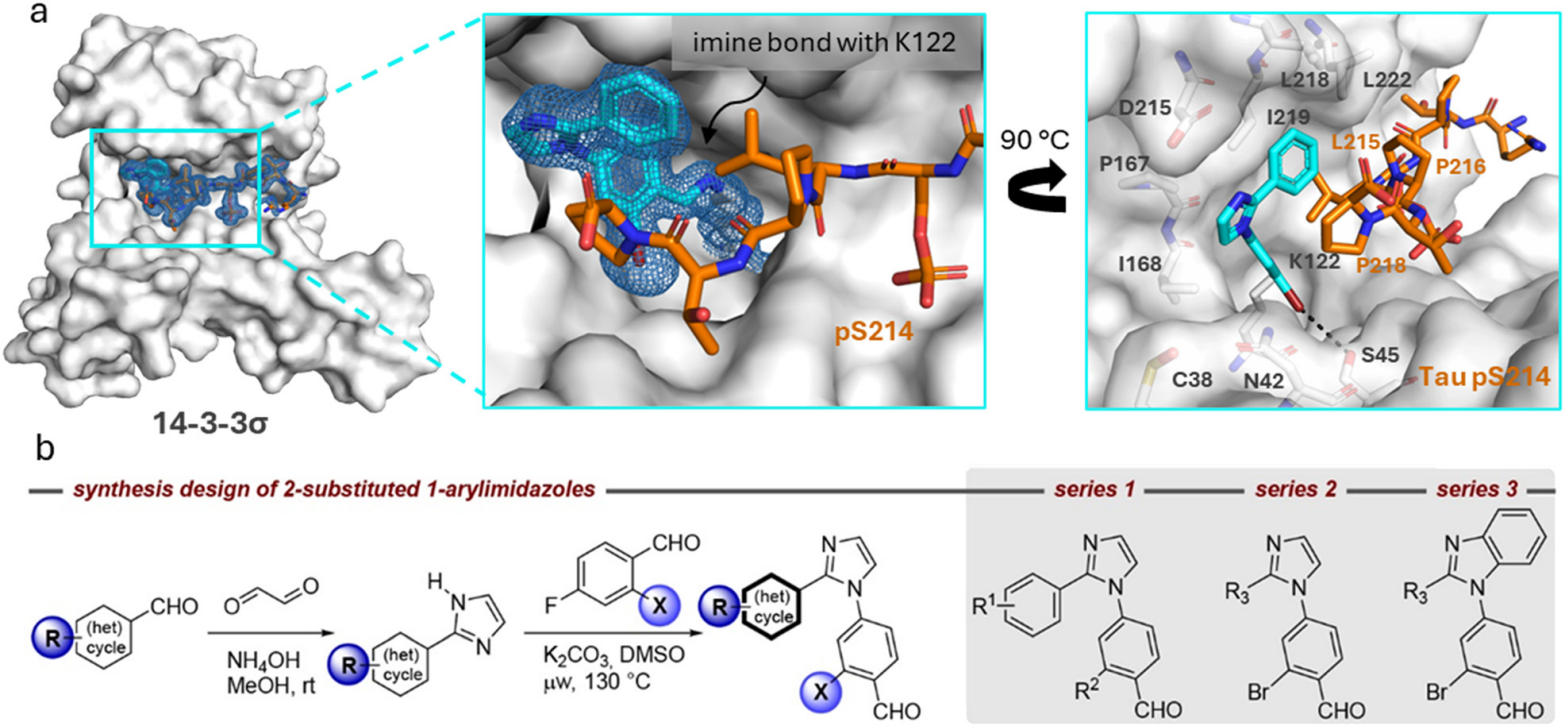
a (Left) Crystal structure of ternary complex 14-3-3σΔC/Tau pS214 peptide/1. (Middle) Zoom-in of the crystallized ternary complex 1 (blue sticks)/14-3-3σΔC (white surface)/Tau pS214 peptide (orange sticks) which shows the imine bond formation between 1 and K122 of 14-3-3σΔC. 2Fo–Fc electron density maps are contoured at 1σ. (Right) Zoom-in demonstrating the halogen-bond of 1 (blue sticks) with its bromo substituent to the hydroxy group of S45 residue of 14-3-3σΔC. The phenyl ring of 1 is positioned in a hydrophobic pocket shaped by residues L215, P216, and P218 of the Tau pS214 peptide (orange sticks) and L218 and I219 of 14-3-3σ (white sticks). Stacking of P218 on the interface of the phenyl group allows for the lone pair–π interaction between P218 and the π-electron system of phenyl ring. Potential chemical space created by 14-3-3 surrounding compound 1 is shown in white sticks. PDB ID: 9FS4. b Synthesis design of 2-substituted 1-arylimidazoles and synthesis route of compound library. General chemical structures of the three compound series 1–3 are shown.

Structural characterization of compounds 1 and 2 soaked into the 14-3-3σΔC/Tau pS214 binary complex using X-ray crystallography confirmed that the stabilizers covalently bind to the K122 residue of the 14-3-3σ protein forming an imine bond (Fig. 2a, ESI† Fig. S3). 14-3-3σ was used for our crystallography studies, since it has been empirically found to crystallize most successfully. Further exploration of this binding mode (Fig. 2a) revealed that 1 forms an additional halogen-bond with its bromo substituent to the hydroxy group of S45 residue of 14-3-3σ. Furthermore, the phenyl ring of 1 is positioned in a hydrophobic pocket shaped by residues L215, P216, and P218 of the Tau pS214 peptide and L218 and I219 of 14-3-3σ. Stacking of P218 on the interface of the phenyl group of 1 allows for a lone pair–π interaction between the lone pair nitrogen atom of P218 and the π-electron system of phenyl ring, respectively.^2^ This characteristic topology and the dynamic covalent nature of the 14-3-3σΔC/Tau pS214 peptide/1 composite interface promised accelerated hit optimization towards more potent and selective stabilizers for this PPI as previously demonstrated for other 14-3-3 protein complexes targeted *via* reversible imine-tethering such as 14-3-3/Pin1 and 14-3-3/p65.^31–33^ The aldehyde moiety offers a finely balanced electrophilic nature resulting in an affinity-driven binding event that is strengthened by a covalent imine bond rather than a purely reactivity-driven binding.^34^ Beyond 14-3-3 PPI stabilization, reversibly covalent lysine targeting by benzaldehyde-based probes has been shown to be effective to develop kinase inhibitors.^35^ By finetuning the electrophilic benzaldehyde, kinase selectivity has been achieved based on differences in ligand residence time^36^ and targeting of non-catalytic lysine residues located at the surface of a protein has been reported.^37^

### Compound design and synthesis

The chemical space surrounding compound 1 provides an interesting case to generate more conceptual insights for the development of molecular glues in the context of protein complex stabilization by analyzing how i) the imidazole scaffold could be further extended towards the entry of the 14-3-3 binding groove to obtain additional hydrophobic interactions with the residues P167 or I168 of 14-3-3, ii) residues N42, C38, or D215 of 14-3-3 could be targeted using H-bond donor or acceptor groups, iii) the Tau pS214 peptide could be addressed by polar interactions with the peptide backbone carbonyl groups, and iv) the hydrophobic pocket surrounding the phenyl ring of compound 1 could be further exploited to occupy the chemical space created by 14-3-3 residues L218, I219, and L222 (Fig. 2a). Moreover, based on the deviations of the two phosphoserine peptide sequences and the structural analysis of the ternary complex 14-3-3σΔC/Tau pS214 peptide/1, we reasoned that the development of phosphosite-selective molecular glues can be achieved by variations of the unsubstituted phenyl ring of hit compound 1.

To introduce structural modifications to the phenyl ring of hit compound 1, a new compound library was constructed. The 2-substituted imidazole scaffold was obtained by condensation reaction of an aldehyde and glyoxal in the presence of ammonia (Fig. 2b). Subsequent S_N_Ar chemistry allowed the access to the 1,2-disubstituted (benz)imidazoles. The resulting focused library of about 45 compounds can be divided into three subseries (Table S1†). Series 1 encompasses the incorporation of various substituents at the phenyl ring of compound 1, series 2 features aromatic and saturated heterocycles, as well as fused ring systems at the imidazole 2 position. Finally, series 3 features a benzimidazole scaffold resembling hit compound 2.

### Stabilization of 14-3-3/Tau pS2 peptide

**Fig. 3:**
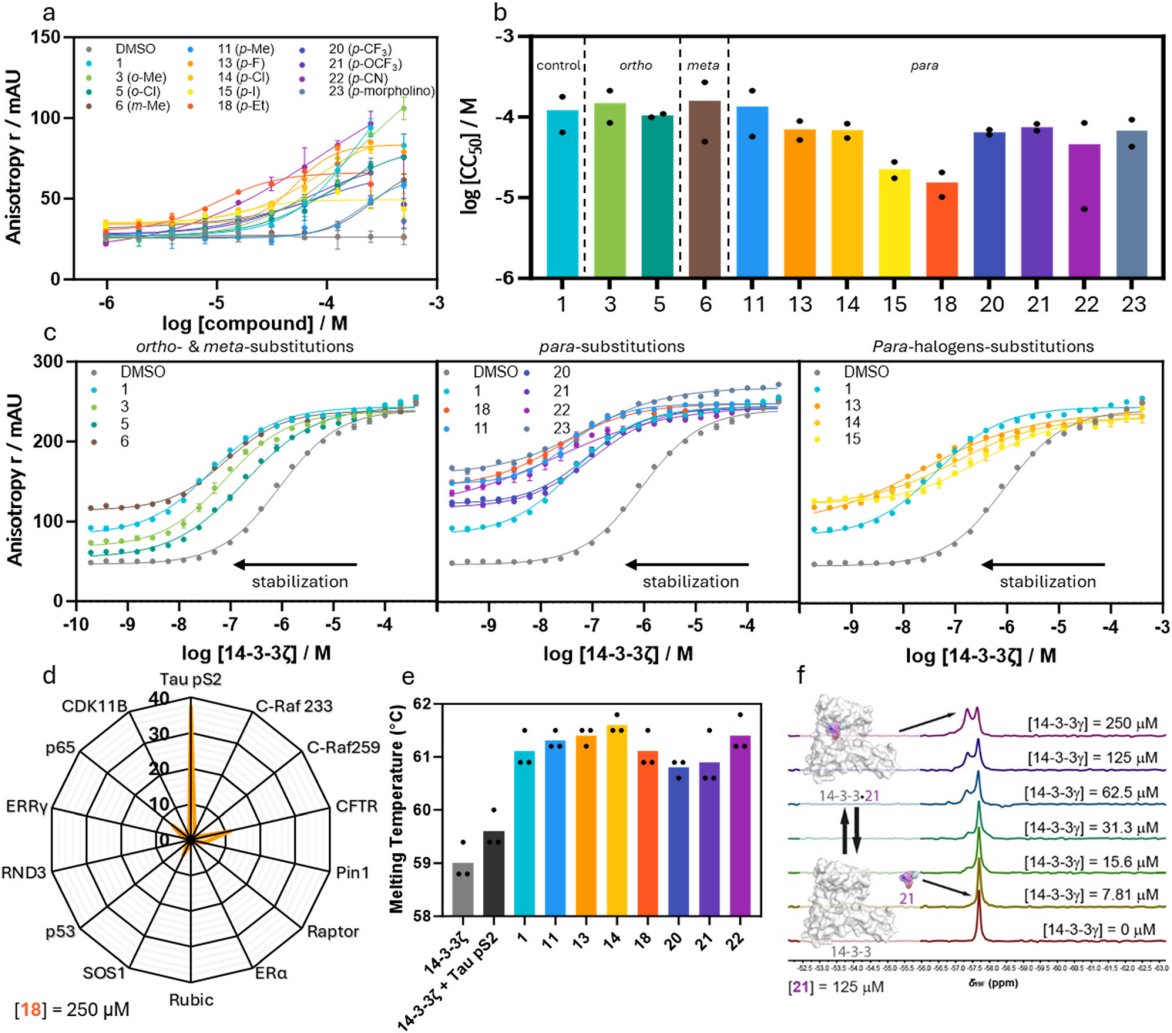
a Dose–response fluorescence anisotropy data of compound titrations to 10 nM FITC-labeled Tau pS2 peptide and 100 nM 14-3-3ζ. Background (without 14-3-3ζ) subtracted, overnight measurement, mean and SD of technical duplicate (see ESI† Fig. S5 for another replicate). b Bar plot representation of CC_50_ values of the compound titrations shown in (a), mean and SD of scientific duplicate. c Dose–response fluorescence anisotropy data of 14-3-3ζ titrations to 10 nM FITC-labeled Tau pS2 peptide and 250 μM compound, mean and SD of technical duplicate (see ESI† Fig. S6 for another replicate). d Radar plot of stabilization factors at 250 μM compound 18 (SF_250_) from 14-3-3ζ FA protein titrations with 10 nM (Tau pS2 peptide) or 100 nM (13 other peptides) FITC-labeled peptide. Mean of three individually performed experiments (see ESI† Fig. S8 for the protein titrations). e Differential scanning fluorimetry assay results. Identified melting temperatures (*T*_m_) of 14-3-3γ alone (2.5 μM) and in the presence of Tau pS2 peptide (25 μM) and additionally with selected compounds (75 μM). Mean of three individually performed experiments. f ^19^F-NMR spectra (565 MHz, 298 K, 10 vol% D_2_O in H_2_O) of compound 21 (125 μM) at varying concentrations of 14-3-3γ (0–250 μM) indicates ligand binding to 14-3-3γ.

With the compound library constructed, we sought to screen the stabilizing activity of the imidazole analogs for the 14-3-3ζ/Tau pS2 peptide interaction. In this regard, a fluorescence anisotropy (FA) assay was performed where each individual compound was titrated against a fixed concentration of 14-3-3ζ (100 nM) and fluorescein-labelled Tau pS2 peptide (10 nM; Fig. 3a and b, ESI† Fig. S4 and S5, Table 1, ESI† Table S1). Thereby, the fixed 14-3-3ζ concentration was based on the EC_20_ value of the binary complex formation to enable a sufficient dynamic window for ternary complex formation. The stabilizing activity can be quantified by the inflection point of the curve, which represents the half-maximum ternary complex formation (CC_50_) value with the ternary complex consisting of 14-3-3ζ/Tau pS2 peptide/compound. For each compound dose–response curve, the background signal (titration without 14-3-3ζ) was subtracted, to exclude possible false positive results.

From the compound screen, it was apparent that only analogs from series 1 and 3 showed PPI stabilizing activity (ESI† Fig. S4, ESI Table S1). Compounds from series 2 which feature heteroarenes, heterocyclic fused ring systems, or saturated heterocycles at the imidazole 2-position showed either no or only minor PPI stabilization (ESI† Fig. S4, ESI Table S1). On the other hand, the PPI stabilizing potency of series 1 compounds was highly dependent on the position and chemical property of the substituent. Analog 3 featuring an *o*-Me substituent showed decreased PPI complex formation with a CC_50_ value of 149 ± 64 μM, compared to the parent compound 1 with a CC_50_ value of 122 ± 57 μM. Compound 5 featuring an *o*-Cl showed a slightly increased PPI stabilization with a CC_50_ value of 105 ± 5 μM. The *meta* position of the phenyl ring was revealed to be delicate for PPI stabilization since only analog 6 featuring a *m*-Me substitution was found active with an CC_50_ value of 160 ± 110 μM. Analogs with other *meta* substituents (Cl, OH, and OMe) did not show any PPI stabilizing activity. Gratifyingly, compounds 11 and 14 featuring *p*-Me and *p*-Cl substituents showed PPI stabilization with CC_50_ values of 135 ± 78 μM and 69 ± 14 μM, respectively. The compound screen for these compounds was further complemented by 14-3-3ζ titration assays in the presence of a constant concentration of compound (250 μM) and Tau pS2 peptide (10 nM) (Fig. 3c, ESI† Fig. S6). This specific compound concentration was selected because maximal ternary complex formation for the most potent compounds was observed in the FA assays.

The apparent *K*_D(250μM)_ represents the affinity of the 14-3-3ζ/Tau pS2 peptide complex in the presence of compound (250 μM) and is compared to the internal DMSO control to determine the fold stabilization (SF_250μM_). Among the five compounds that showed stabilization, only 11 (*K*^app^_D(250μM)_ = 32 ± 4 nM, SF_250μM_ = 29) resulted in an improved increase in stabilization as compared to hit compound 1 (*K*^app^_D(250μM)_ = 40 ± 6 nM, SF_250μM_ = 23). Compounds 3 (SF_250μM_ = 11) and 6 (SF_250μM_ = 10) featuring methyl groups in the *ortho* and *meta* position, respectively, showed stabilization properties, albeit reduced as compared to parent compound 1. This weaker PPI stabilization indicates that less chemical space is available in the *meta* position as compared to the *ortho* position, which has again less chemical space as compared to the *para* position. This statement is strengthened by the diminished stabilizing activity when shifting the more polar chloro substituent from the *para* (14) to the *ortho* (5) to the *meta* position (8), respectively. Moreover, all other profiled *meta*-substituted analogs did not show activity in the compound titration, while aldehyde 9 even slightly inhibited 14-3-3ζ/Tau pS2 peptide complex formation. Modification of the *para*-position was revealed to be preferred compared to the *ortho* and *meta* position. Furthermore, it was found that the electron-donating methoxy and hydroxy substituents led to a strong decrease in stabilization for all positions on the phenyl ring, pointing towards a crucial role for the electron density in the phenyl ring in PPI stabilization.

**Fig. 4:**
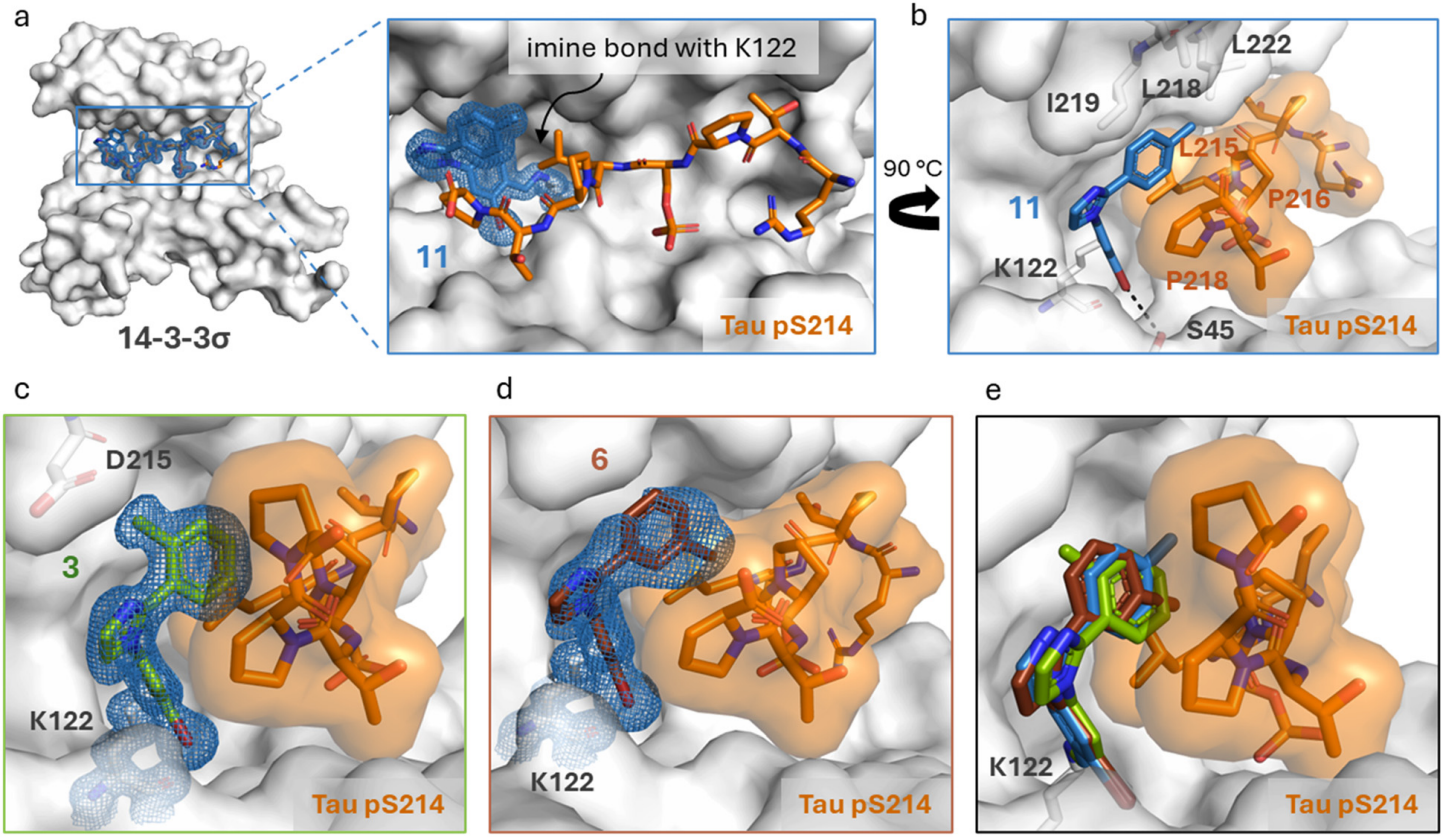
Crystal structures of methylated compounds in complex with 14-3-3σΔC/Tau pS214. a Front view of the ternary complex 14-3-3σΔC (white surface), Tau pS214 (orange sticks) and 11 (blue sticks). Zoom in of the crystallized ternary complex 11 (blue sticks)/14-3-3σΔC (white surface)/Tau pS214 (orange sticks) which shows the imine bond formation between 11 and K122 of 14-3-3σΔC. 2Fo–Fc electron density maps are contoured at 1σ. PDB ID: 9FVP. b Side view of the ternary complex 14-3-3σΔC (white surface), Tau pS214 (orange sticks) and 11 (blue sticks). c Side view of the ternary complex 14-3-3σΔC (white surface), Tau pS214 (orange sticks) and 3 (green sticks). 2Fo–Fc electron density maps are contoured at 1σ. PDB ID: 9FVH. d Side view of the ternary complex 14-3-3σΔC (white surface), Tau pS214 (orange sticks) and 6 (brown sticks). 2Fo–Fc electron density maps are contoured at 1σ. PDB ID: 9FVI. e Overlay of the three methylated compounds inside the 14-3-3σΔC/Tau pS214 complex.

The methyl-substituted compounds 3 (*o*-Me), 6 (*m*-Me), and 11 (*p*-Me) were soaked into co-crystals of 14-3-3σΔC and Tau pS214 (Fig. 4). Analysis of their X-ray co-crystal structures showed clear densities for all molecular glues and provided valuable structural insights into their activity profile. Compound 11 (*p*-Me) showed a similar binding mode as parent compound 1 including a covalent imine bond with K122 of 14-3-3σ and an halogen-bond between the bromine atom and the S45 residue of 14-3-3σ. Additionally, the *p*-Me group of 11 is positioned in the hydrophobic pocket formed by L218, I219, and L222 in the “roof” of 14-3-3σ and L215 and P218 of the Tau pS214 peptide. These additional hydrophobic interactions are expected to cause the observed increase in the stabilization of the 14-3-3ζ/Tau pS2 peptide complex. To compare the binding modes when shifting the methyl group to the *ortho*- and *meta*-position, the crystal structures of the ternary complexes with compounds 3 and 6 are visualized in Fig. 4c and d. The methyl group of 3 points towards the polar residue of D215 and the methyl group of 6 points towards the carbonyl groups of the peptide backbone, which both do not lead to favorable interactions. Moreover, when superimposing the structures of the three compounds 3, 6, and 11, it became apparent that compounds 3 and 6 were slightly shifted in position as compared to 11 (Fig. 4e). This altered stabilizer position indicates that substitutions in the *ortho*- or *para*-position are sterically critical for the compound to engage in the most favorable orientation. In addition, the shift in position of compounds 3 and 6 could potentially distort the lone pair–π interaction between the nitrogen atom lone pair of P218 of 14-3-3 and the π-electron system of the phenyl ring in stabilizer 1.

Since the *para* position was revealed to be crucial for improved PPI stabilization, a new set of analogs was synthesized featuring various *para* substituents to further probe the composite binding pocket surrounding the phenyl ring. Thereby, hydrophobic groups (18, 19), halogens (13, 15), electron-withdrawing groups (20–22), and polar substituents (23, 24) were introduced. The quantified CC_50_, *K*^app^_D(250μM)_, and SF_250μM_ values are shown in Table 1. Substituting the *p*-Me group of 11 (SF_250μM_ = 29) for an *p*-Et group in 18 (SF_250μM_ = 37) resulted in a further increase in protein complex stabilization. However, expanding the ethyl into a sterically more demanding isopropyl group (19) completely diminished PPI stabilization.

This suggests that the hydrophobic pocket at the *para*-position of the phenyl group is rather narrow and branching at the α-carbon of the substituent is not tolerated. In line with this result, 23 (SF_250μM_ = 16) featuring a morpholino substituent showed reduced stabilization compared to reference compound 1 (SF_250μM_ = 23).

A similar trend was observed for the halogen substitutions in compounds 13–15. The analogs featuring lighter halogens stabilize the 14-3-3ζ/Tau pS2 peptide PPI to a greater extent, with the *p*-F substituent in 13 reaching a SF_250μM_ of 33 (14: *p*-Cl, SF_250μM_ = 8.9; 15: *p*-I, SF_250μM_ = 3.1).

Analogous to the previous library, analog 24 with an electron-donating ether substituent did not show an improvement in PPI stabilization. In contrast, compound 22, which features a relatively small and electron-withdrawing nitrile group installed at the *para* position of phenyl ring, improved stabilization (SF_250μM_ = 34). Analogs 20 (SF_250μM_ = 13) and 21 (SF_250μM_ = 11) with the lipophilic, electron-withdrawing groups CF_3_ and OCF_3_, respectively, showed reduced PPI stabilization potency.

To investigate the importance of the 2′-Br substituent influencing the electrophilicity of the benzaldehyde, we profiled a focused set of analogs 25–27 (Table 1). While compound 25 with the relatively small and electronegative 2′-Cl substituent showed a SF_250μM_ of 15, PPI stabilization was almost completely diminished with compound 26 featuring the less electronegative, but also bigger, 2′-I group (SF_250μM_ = 1.8). However, stabilization was not solely driven by electronegativity since analog 27 with a 2′-CF_3_ group (SF_250μM_ = 6.1) elicited less stabilization than 1 (2′-Br, SF_250μM_ = 23). These results suggest that 14-3-3ζ/Tau pS2 peptide stabilization is driven by an interplay of molecular recognition of the composite 14-3-3/Tau pS2 peptide binding pocket and the reactivity of the aldehyde as previously stated by Verhoef *et al.*^34^ Further structural investigation is required to substantiate this statement since no crystal structures were obtained for these compounds.

Within the previous compound sets, decorations of the phenyl group of hit compound 1 were explored, keeping the imidazole core unaltered. During our initial screening campaign, it became apparent that hit compound 2 engaged in the 14-3-3σΔC/Tau pS214 composite pocket with a distinct orientation compared to imidazole 1 but still showed potent PPI stabilization (SF_250μM_ = 20, ESI† Fig. S3b). To probe the altered orientation of the PPI stabilizer in the ternary complex, a few benzimidazole analogs 28–31 were synthesized, their PPI stabilization activity screened, and the ternary complexes structurally characterized (series 3, Table 1). The additional molecular surface of the benzimidazole core was expected to probe the hydrophobic amino acid residues at the entry of the 14-3-3 binding groove as previously outlined. FA data revealed that among series 3, only benzimidazole 31 featuring a polar and electron-donating 2′-hydroxyethoxy group (*K*^app^_D(250μM)_ = 27 ± 16 nM, SF_250μM_ = 19) elicited similar stabilization as compared to lead compound 1 (*K*^app^_D(250μM)_ = 40 ± 6 nM, SF_250μM_ = 23). This could be attributed to enhanced occupancy of the hydrophobic entrance of the 14-3-3 binding groove shaped by P167, I168, and I219 as evident from its X-ray structure (ESI† Fig. S7).

Benzimidazole 29 with a *p*-OMe substituent (*K*^app^_D(250μM)_ = 63 ± 21 nM, SF_250μM_ = 8.3) showed a stronger affinity for the 14-3-3ζ/Tau pS2 peptide complex based on the CC_50_ value but was found less potent compared to lead compound 1 (*K*^app^_D(250μM)_ = 40 ± 6 nM, SF_250μM_ = 23) based on the FA protein titration assays. Its reduced stabilizing activity is expected to be caused by the electron-rich arene, which was previously found to be non-favorable for stabilization in combination with a less efficient occupation of the hydrophobic binding pocket with residues L218, I219, and L222 of 14-3-3 and L215, P216 and P218 of the Tau pS214 peptide compared to the larger *para*-substituent of 31. However, the better occupation of the hydrophobic entrance of the 14-3-3 binding groove, as a result of its benzimidazole scaffold similar to 31, is expected to increase the affinity for binding 14-3-3 leading to a lower CC_50_ compared to parent compound 1.

Since the hub protein 14-3-3 binds to hundreds of phosphoprotein partners, selectivity of PPI stabilization is a major challenge. The interplay of chemical reactivity, and the topology and functionality of the composite binding pocket shaped by 14-3-3 and Tau potentially provide entries towards selective stabilization. To probe this hypothesis, the most potent stabilizer 18 was tested against a panel of 13 varying 14-3-3 client peptides in FA assays (Fig. 3d, ESI† Fig. S8). Here, 14-3-3ζ was titrated to a constant concentration of client peptide (100 nM) and compound (250 μM) to determine the SF_250μM_ values for the particular 14-3-3 PPI. A variety of 14-3-3 binding proteins was represented in this client panel whereby the size and hydrophobicity of the +1 amino acid were varied. Notably, client peptide Pin1 for which the lead compound 1 was initially designed, was included in this panel. Aldehyde 18 proved selective for the 14-3-3ζ/Tau pS2 peptide complex (Fig. 3d). Whereas 1 was found to have a SF_100μM_ of 10 for stabilization of the 14-3-3/Pin1 complex,^31^ the SF_250μM_ of 18 for 14-3-3/Pin1 PPI stabilization was decreased to only 3.9. Aldehyde 18 elicited only some minor off-target effects against CFTR, SOS1, and p65, which contain a +1 Val, Ala and Ile respectively. Compared to Tau pS2 peptide which contains two +1 Leu residues, this finding suggests that small hydrophobic residues are preferred in the +1 position. Moreover, aromatic amino acids Trp, Phe, Tyr in this position as represented by Pin1, Rubic, and Raptor peptides, respectively showed no significant stabilization by 18, which strengthens this hypothesis. Polar +1 amino acids such as Thr, Cys, and Glu in C-Raf, ERRγ/RND3, and p53 respectively did not show significant stabilizing activity of 18.

As an orthogonal method to profile the PPI stabilizing activity of selected compounds, differential scanning fluorimetry (DSF) assays were performed (Fig. 3e). Thereby, a 10-fold excess of Tau pS2 peptide relative to 14-3-3ζ resulted in a slight increase of the 14-3-3 melting temperature as compared to apo-14-3-3ζ (Δ*T*_m_ ∼ 0.6 °C). Upon addition of compounds (1, 11, 13–14, and 20–22) to this PPI complex (30-fold compared to 14-3-3ζ), an additional increase in melting temperature between 0.9 °C and 2.1 °C was observed relative to the binary 14-3-3ζ/Tau pS2 peptide system. These observations are in agreement with the stabilization observed in the FA data and strongly testify to the molecular glue effect of our compound, by enhancing the stability of the PPI complex.

The major mechanism by which ternary complex formation occurs most probably involves the initial binding event of Tau pS2 peptide to 14-3-3ζ, to form a binary complex (*K*_D_ = 945 ± 70 nM), to which subsequently the herein described molecular glues bind with varying affinities (see CC_50_ values). This stepwise mechanism has been described in detail for related benzaldehyde stabilizers and the 14-3-3/Pin1 interaction.^34^ However, the imidazolyl-benzaldehydes might also show an intrinsic binding affinity for 14-3-3. To elucidate the underlying processes, we performed ^19^F-NMR spectroscopy studies (Fig. 3f) with the fluorinated analogs 20 and 21. At a constant stabilizer concentration (125 μM), the 14-3-3 concentration was varied from 0 μM to 250 μM. At higher 14-3-3 concentration, a second peak with a distinct chemical shift appeared in a concentration-dependent manner which strongly indicates the formation of the binary 14-3-3/stabilizer complex. Although in previous studies the affinity of related compounds to 14-3-3 was shown to be very weak as determined by native MS,^34^ we found a 1 : 1 ratio of unbound and bound stabilizer at a 14-3-3/stabilizer ration of 2 : 1. However, we did not further elucidate where the stabilizer bind Apo-14-3-3 which might differ from the binding motif in the ternary 14-3-3/Tau/stabilizer complexes.

### Profiling phosphosite-selective PPI stabilization

**Fig. 5:**
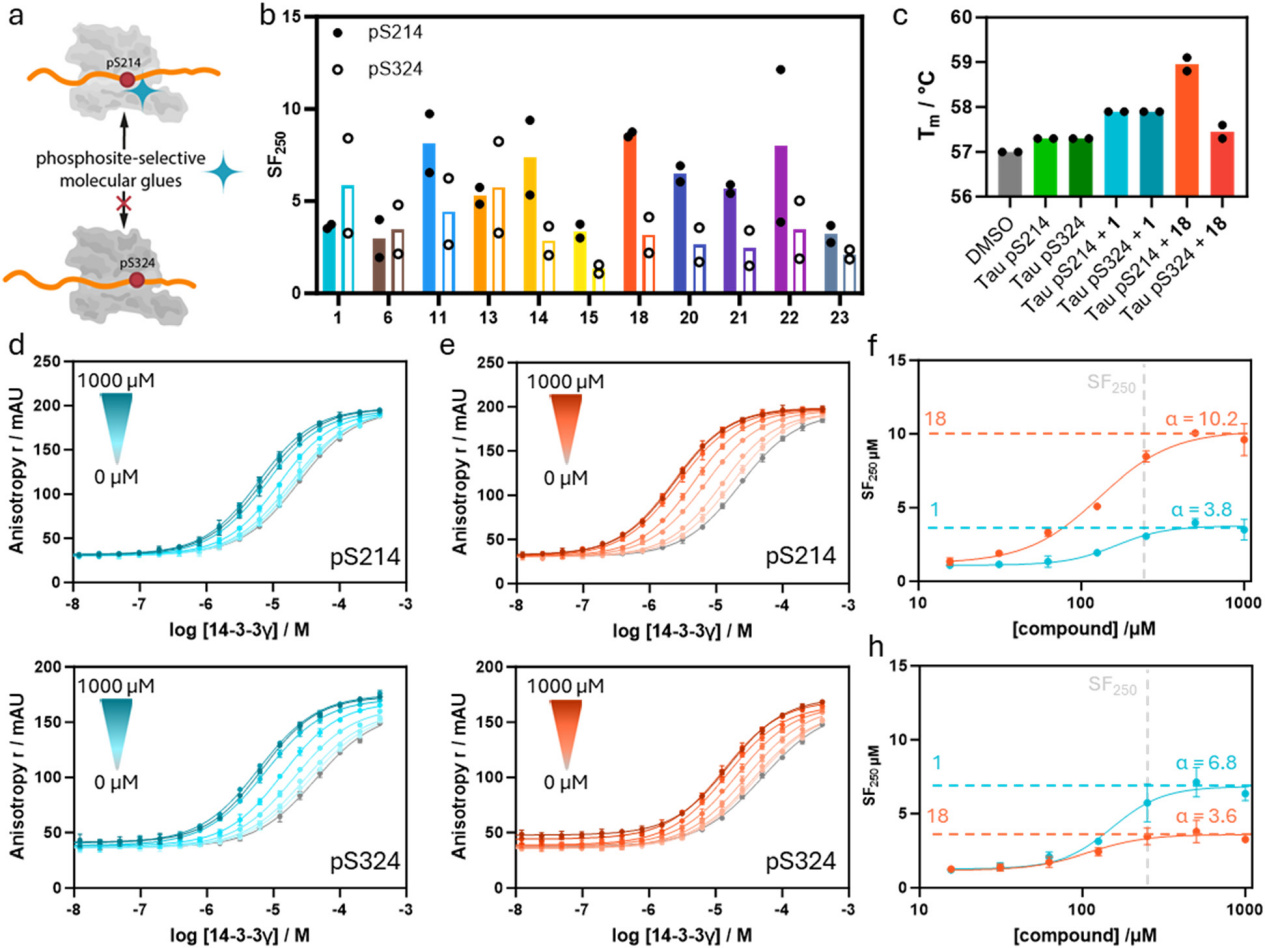
a Phospho-site selective 14-3-3/Tau PPI stabilization. b SF_250μM_ of selected compounds for stabilization of the 14-3-3γ complex with Tau pS214 and Tau pS324 peptides based on the internal DMSO reference. Mean of two separately performed experiments (see ESI† Fig. S9 for the corresponding binding curves). c Differential scanning fluorimetry assay results. Identified melting temperatures (*T*_m_) of 14-3-3γ in the presence of the Tau pS214 or pS324 peptide and compounds 1 and 18. Mean of two separately performed experiments. d and e 14-3-3γ titrations in the presence of increasing concentrations of compounds 1 (d) and 18 (e), in the presence of either 100 nM fluorescein-labeled Tau pS214 or fluorescein-labeled Tau pS324. Overnight measurement, mean and SD of scientific duplicate. f, SF_250μM_ of the 14-3-3γ/Tau pS214 interaction plotted against compound concentrations. The data are derived from 2D titrations (d and e). The saturation of the ratio represents the *α*-factor. (h) Ratio of *K*_D_/apparent *K*_D_ of the 14-3-3γ/Tau pS324 interaction plotted against compound concentrations. The data are derived from 2D titrations (figures d and e). The saturation of the ratio represents the *α*-factor.

To map the Tau phosphosite selectivity of the developed PPI stabilizers, the most potent compounds were screened against the monovalent Tau pS214 and Tau pS324 peptide PPIs with 14-3-3 (Fig. 5a). Although these Tau peptides both feature a +1 Leu residue relative to the phosphorylated serine, it was hypothesized that the exploitation of the unique topology of 14-3-3/Tau pS214 promises a certain degree of phospho-binding site selective stabilization between those two Tau sites.

FA assays in which 14-3-3γ was titrated to a constant concentration of compound (250 μM) in the presence of the distinct monovalent Tau peptide (10 nM) were performed. Due to its higher binding affinity for the monovalent Tau peptides, 14-3-3γ was utilized instead of 14-3-3ζ, enabling easier detection of the underlying PPI. The Tau pS214 peptide itself was found to bind 14-3-3γ with a slightly higher affinity compared to Tau pS324 (*K*_D_ = 24 ± 5 μM and 43 ± 17 μM, respectively, ESI† Fig. S9).

The obtained SF_250μM_ values of each compound for the interaction of 14-3-3γ with Tau pS214 and Tau pS324 are depicted in Fig. 5b. Gratifyingly, the imidazoles 11, 14, 15, 18, and 20–22, which all feature *para*-substitutions on the phenyl ring, stabilize the 14-3-3γ/Tau pS214 interaction to a significantly greater extent compared to the 14-3-3γ/Tau pS324 complex. In line with that, parent compound 1 without any substituent on the phenyl ring did not show a difference in stabilization of the two different 14-3-3/Tau interactions.

Imidazole 18 was found to exert the highest stabilization selectivity for the 14-3-3γ/Tau pS214 complex (SF_250μM_ = 8.6) over the 14-3-3γ/Tau pS324 (SF_250μM_ = 3.2). Imidazole 6 with an *ortho*-substituted phenyl ring showed no significant preference for one of the two monovalent Tau complexes with 14-3-3γ. Additionally, compound 13, with the relatively small *p*-F substituent, also had no preference for Tau pS214, where compounds 14 (*p*-Cl) and 15 (*p*-I) did show a preference for Tau pS214. Since other electron-withdrawing substituents like *p*-CN (22) also showed a preference for Tau pS214, the size of the *para*-substituent on phenyl ring is assumed to play a central role in phospho-binding site selective PPI stabilization.

The determined stabilization factors are concentration-dependent (here 250 μM of compound) and might differ based on assay design. Hence, we aimed to determine the apparent cooperativity factor (*α*) as a concentration-independent measure of apparent (app.) cooperativity towards both 14-3-3/Tau complexes. To assess the app. cooperativity of the ternary complex formation, parent compound 1 and the most potent stabilizer 18 were selected. The app. *α*-factors of these compounds for both 14-3-3/Tau complexes were determined by 14-3-3γ titrations in the presence of varied concentrations of the particular compound (Fig. 5d and e). Both compounds reached saturation for both 14-3-3γ/Tau complexes enabling accurate determination of the *α*-factor. The app. *α*-factors of aldehydes 1 and 18 were plotted for both 14-3-3γ/Tau complexes to investigate phospho-binding site selective cooperativity (Fig. 5f and h). To do so, the stabilization factors at a given compound concentration were plotted against the compound concentration, leading to the determination of the app. *α*-factors. An app. cooperativity value of 10.2 was observed for the 14-3-3γ/Tau pS214/18 complex, whereas for Tau pS324 an app. *α*-factor of 3.6 was obtained, indicating a phospho-binding site favoured cooperativity of analog 18 for the 14-3-3γ/Tau pS214 complex. Intriguingly, parent compound 1 showed a slight preference to stabilize the 14-3-3γ/Tau pS324 complex (app. *α* = 6.8) as compared to the 14-3-3γ/Tau pS314 complex (app. *α* = 3.8).

Differential scanning fluorimetry (DSF) assays were conducted to further validate phospho-site selective stabilization for the different 14-3-3/Tau complexes (Fig. 5c). The addition of Tau pS214 and Tau pS324 (each 10 equiv.) to 14-3-3γ both elicited a minor increase of ∼0.3 °C in melting temperature of 14-3-3γ. The equal increase of the 14-3-3γ melting temperature (∼0.6 °C) observed with the addition of compound 1 (30 equiv.) to both preformed 14-3-3γ/Tau complexes aligns with the previous findings that stabilizer 1 is not selective for either of the two 14-3-3/Tau binding sites. Notably, the addition of compound 18 (30 equiv.) resulted in a ∼1.7 °C increase in melting temperature for the 14-3-3γ/Tau pS214 complex and only in a slight increase of melting temperature of ∼0.2 °C for the 14-3-3γ/Tau pS324 complex compared to 14-3-3γ alone. These results thus further confirm the pS214 site selective stabilization of the 14-3-3/Tau complex.

**Fig. 6:**
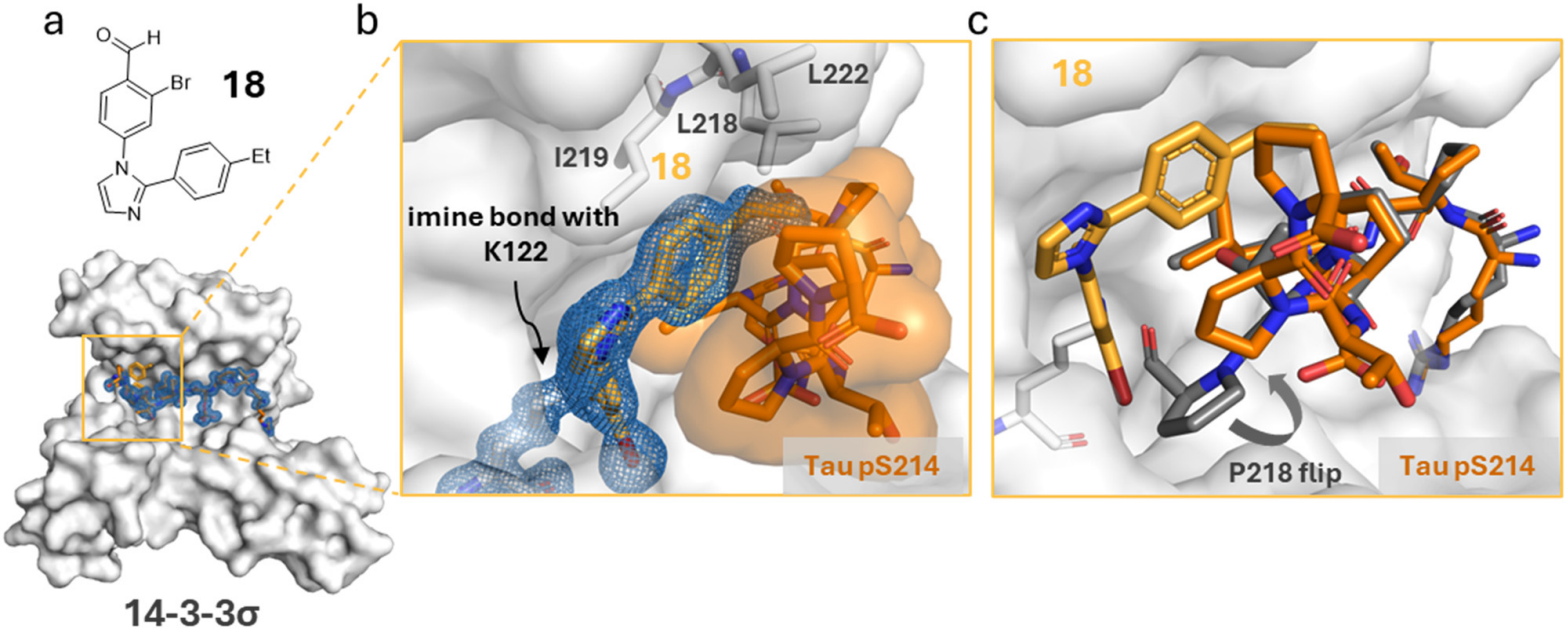
a Chemical structure of compound 18. b Ternary structure of 14-3-3σΔC (white surface), Tau pS214 (orange sticks) and 18 (light orange sticks) complex. Zoom-in of the crystallized ternary complex 18 (light orange sticks)/14-3-3σΔC (white surface)/Tau pS214 (orange sticks) which shows the imine bond formation between 18 and K122 of 14-3-3σΔC and the occupation of the hydrophobic groove of 14-3-3 (white sticks). 2Fo–Fc electron density map (blue mesh) is contoured at 1σ (PDB ID: 9FVG). c Side view of an overlay of the ternary structure of 14-3-3σΔC (white surface)/Tau pS214 (orange stick)/18 (light orange sticks) complex with the binary 14-3-3σΔC/Tau pS214 (grey sticks) complex (PDB ID: 4FL5). The grey arrow represents the conformational change of the P218 residue due to compound 18.

Cooperative PPI/molecular glue complex formation often originates from structural changes at the interface of the PPI complex which translate to increased stability of the ternary complex.^5^ Here, structural insights into the cooperativity and phospho-binding site selectivity were gained by protein crystallography. The crystal structure of the ternary complex including 18 soaked into the co-crystal of 14-3-3σΔC and Tau pS214 revealed a similar binding mode as compared to analog 11 (Fig. 6a and b). The longer alkyl group of 18 shows increased interactions within the hydrophobic pocket formed by L218, I219, and L222 in the “roof” of 14-3-3 and L215 and P218 of the Tau pS214 peptide, explaining its increased SF. It was observed that in the solid-state structure overlay of 14-3-3γ/Tau pS214/18 with the 14-3-3σΔC/Tau pS214 peptide binary complex (PDB code 4FL5), the P218 residue of the Tau peptide rotated in the presence of compound to enable the lone-pair–π interaction (Fig. 6c). This interaction, accompanied with the increased hydrophobic interactions of the *p*-Et group of stabilizer 18, explain the improved PPI stabilization. For the ternary 14-3-3γ/Tau pS324/18 complex, in contrast, only poor electron density of the Tau peptide and stabilizer could be obtained, with only ∼3–4 amino acids of Tau being accurately resolved (ESI† Fig. S10). This obviously reflects the lower stabilization of this Tau binding site by compound 18.

## Conclusion

Targeting a specific PPI of the hub protein 14-3-3 over the other PPIs made by the hundreds of other potential 14-3-3 partner proteins poses the challenge of selectivity. Thereby, not only selectivity towards 14-3-3 complexes with other client proteins but also selective stabilization of the interaction of a specific binding site from a multivalent 14-3-3-binding protein is of high relevance for the development of chemical tools and probes to investigate biological questions.

The interaction of 14-3-3 with Tau, characterized by different phospho-site driven binding modes, forms an excellent case study. Tool compounds that selectively stabilize distinct 14-3-3/Tau binding modes have the potential to reveal mechanistic insights into the multifaceted role of 14-3-3 in Tau aggregation and potentially be utilized to reduce Tau amyloidogenesis, in dedicated follow-up studies.

Here we report the identification and optimization of early-stage small molecule molecular glues for the PPI of 14-3-3 and bivalent Tau pS214–pS324 peptide. The PPI stabilizing potency of the compound library was mapped using FA assays, differential scanning fluorimetry assays, and X-ray crystallography to provide structural insights into the ternary complexes. Beyond the bivalent Tau peptide, exploiting the unique functionalities and topologies of the 14-3-3/Tau peptide composite binding pockets enabled the development of molecular glues preferably stabilizing the pS214 phospho-binding site over the pS324 site of Tau. Cooperativity analysis of ternary complex formation, together with X-ray crystallography, was used to compare and explain the efficacy of the phospho-binding site selective molecular glues. It became apparent that even subtle modifications like the incorporation of a methyl group can result both in higher PPI stabilization potency and in significantly increased selectivity for the 14-3-3/Tau pS214 interaction relative to other client proteins and Tau pS324.

This case study highlights an endeavour toward phospho-binding site selective molecular glues, harnessing structural differences of the composite pockets formed by 14-3-3 and the corresponding partner protein/peptide. Analysis of cooperativity of ternary complex formation provides an analytical tool to guide compound screening or optimization. Even though these molecular glues require further potency optimization and are not fully selective for one specific 14-3-3 PPI, their small, almost fragment-like nature, bodes well for further optimization opportunities. Next to strongly interfacing with the +1 amino acids of the client protein's binding site, building interactions with other amino acids of the client peptide will both enhance potency and further develop selectivity. The fact that compound 18 already shows such promising selectivity for the 14-3-3/Tau pS214 interface, despite the 14-3-3/Tau pS324 interaction also having leucine as +1 amino acid, further strengthens this notion. These compounds and further improved analogs will be valuable tool compounds to help evaluating the multifaceted role of 14-3-3 in Tau aggregation.

## Data availability

The data supporting this article have been included as part of the ESI.† Crystallographic data for 1, 2, 3, 6, 11, 18, and 31 with 14-3-3/Tau pS214 has been deposited at the PDB with IDs: 9FS4, 9GFA, 9FVH, 9FVI, 9FVP, 9FVG, and 9FVN.

## Conflicts of interest

The authors declare the following competing financial interest(s): L. B. and C. O. are scientific co-founders of Ambagon Therapeutics.

## Supplementary Material

MD-016-D4MD00833B-s001
