# Supplementary material for: Site-specific molecular glues for the 14-3-3/Tau pS214 protein–protein interaction via reversible covalent imine tethering
Source: RSC Med Chem. 2025 Feb 7;16(5):2190–201. doi: 10.1039/d4md00833b (PMC11892739; doi:10.1039/d4md00833b)
Supplement: MD-016-D4MD00833B-s001 [file MD-016-D4MD00833B-s001.pdf]

Supplementary Information (SI) for RSC Medicinal Chemistry.
This journal is © The Royal Society of Chemistry 2025

Site-Specific Molecular Glues for the 14-3-3/Tau pS214 Protein-Protein Interaction via Reversible Covalent Imine Tethering

Ansgar Oberheide*, [a], Maxime C. M. van den Oetelaar*, [a], Jakob J. A. Scheele[a], Jan Borggräfe[b, c], Semmy F. H. Engelen[a], Michael Sattler[b, c], Christian Ottmann[a], Peter J. Cossar[a], Luc Brunsveld[a]

* These authors contributed equally

a. Laboratory of Chemical Biology, Department of Biomedical Engineering and Institute for Complex Molecular Systems, Eindhoven University of Technology, Groene Loper 3. 5612 AE Eindhoven, the Netherlands, E-mail: c.ottmann@tue.nl, l.brunsveld@tue.nl

b. Helmholtz Munich, Molecular Targets and Therapeutics Center, Institute of Structural Biology, Ingolstädter Landstrasse 1, 85764 Neuherberg, Germany.

c. Technical University of Munich, TUM School of Natural Sciences, Bavarian NMR Center and Department of Bioscience, Lichtenbergstrasse 4, 85747 Garching, Germany.

Supporting Information

Experimental section

Synthetic procedures

Supporting Tables 1-3

Supporting Figures 1-93

Experimental section

Technical information and experimental procedures

Solvents

All solvents used in this chapter were purchased from Biosolve and were used without further purification. Water was purified by a Milipore purification train. Deuterated solvents were obtained from Cambridge Isotope Laboratories.

Reagents

Reagents were commercially available and were supplied by Sigma-Aldrich, TCI, BLDpharm, Fluorochem, and abcr GmbH.

Thin Layer Chromatography

Reaction progress was monitored by thin-layer liquid chromatography using Merck precoated silica gel plates (60F-254). Compounds were visualized using ultraviolet light irradiation at 254 nm.

Silica Gel Flash Chromatography

Flash column chromatography was performed on a *Biotage Isolera One* using *Büchi FlashPure EcoFlex Silica Cartridges* with 40-63 µm particles. For flash column chromatography, dry loading or liquid loading was performed.

NMR Spectroscopy

^{1}H- and ^{13}C{^{1}H}-NMR spectra were recorded using a *Bruker Avance* 400 MHz spectrometer. Proton spectra are referenced to the internal standard tetramethyl silane (TMS). Carbon spectra are referenced to tetramethyl silane or the solvent peak. NMR spectra are reported as follows: chemical shift, multiplicity
(s = singlet, d = doublet, t = triplet, q = quartet, m = multiplet, dd = doublet of doublet, td = triplet of doublets), coupling constant (*J*) in Hertz (Hz) (if applicable) and integration (proton spectra only). Residual peaks of the particular solvent were used as an internal standard (δ_H($CHCl_3$) = 7.26 ppm, δ_C($CDCl_3$) = 77.16 ppm; δ_H(DMSO-d_5) = 2.50 ppm, δ_C(DMSO-d_6) = 39.52 ppm).

Analytical LC-MS

Analytical liquid chromatography coupled with mass spectrometry (LC-MS) was performed on a C18 Atlantis T3 Column, 100Å, 5 µm, 2.1 mm X 150 mm column using ultrapure water with 0.1% formic acid (FA) and acetonitrile with 0.1% FA, in general with a gradient of 5% to 100% acetonitrile in 10 minutes, connected to a Thermo Fischer LCQ Fleet Ion Trap Mass Spectrometer.

14-3-3γ and 14-3-3σΔc protein expression and purification (FA experiments, DSF & Crystallography)

A pPROEX HTb expression vectors encoding the human 14-3-3 protein gamma (14-3-3γ), the human 14-3-3 zeta (14-3-3ζ) and the human 14-3-3 protein sigma truncated after T231 (14-3-3σΔc, to reduce flexiblity) with an N-terminal his6-tag was transformed by heat shock into NiCo21 (DE3) competent cells. A single colony was selected and cultured overnight in 35 mL LB medium (100 µg/mL ampicillin) at 37°C. After overnight incubation, cultures were transferred to 2 L TB medium (100 µg/mL ampicillin, 1 mM MgCl2) and incubated at 37°C, 140 rpm until an OD600 of 0.6-1.2 was reached. Protein expression was then induced with 0.4 mM isopropyl-β-d-thiogalactoside (IPTG), and cultures were incubated overnight at 18°C, 140 rpm. Cells were harvested by centrifugation (8600 rpm, 20 minutes, 4 °C) and resuspended in lysis buffer (50 mM Hepes, pH 8.0, 300 mM NaCl, 12.5 mM imidazole, 5 mM MgCl2, 2 mM βME) containing cOmplete™ EDTA-free protease inhibitor cocktail tablets (1 tablet/ 100 ml lysate) and benzonase (5 µl/ 100 ml). After lysis using a C3 EmulsiflexC3 homogenizer (Avestin), the cell lysate was cleared by centrifugation (20000 rpm, 30 minutes, 4 °C) and purified using Ni^{2+}-affinity

chromatography (HisTrap high performance cartridges, Cytiva). Typically two 5 mL columns were used for a 2 L culture in which the lysate was loaded on the column, washed with 10 CV wash buffer (50 mM Hepes, pH 8.0, 300 mM NaCl, 25 mM imidazole, 2 mM βME), and eluted with several fractions (2-4 CV) of elution buffer (50 mM Hepes, pH 8.0, 300 mM NaCl, 250 mM imidazole, 2 mM βME). Fractions containing the 14-3-3 protein were combined and dialyzed into 25 mM HEPES pH 8.0, 200 mM NaCl, 10 mM MgCl2, 2 mM βME, followed by dialysis into 25 mM HEPES, 100 mM NaCl, 10 mM MgCl2, 500 µM TCEP (adjusted to pH 8.0). Dialyzed protein was concentrated to ∼40 mg/mL, analyzed by Q-Tof LC/MS, and aliquots were flash-frozen for storage at −80 °C. For 14-3-3σΔc, 1 mg TEV per 100 mg purified protein was added to the dialysis to remove the purification tag. The cleaved sample was then again loaded on a 10 mL Ni-NTA column to separate the cleaved product from the expression tag and residual uncleaved protein. The flowthrough was loaded on a Superdex 75 pg 16/60 size exclusion column (GE Life Sciences) using 25 mM HEPES, 100 mM NaCl, 10 mM MgCl2, 500 µM TCEP (adjusted to pH = 8.0) as running buffer. Fractions containing the 14-3-3 protein were pooled and concentrated to ∼60 mg/mL, analyzed Q-Tof LC/MS, and aliquots were flash-frozen for storage at −80°C.

Fluorescence Anisotropy – compound titration

Compound titration assays were performed by titrating compounds (in 1% DMSO, starting at 500 µM) in a 2-fold dilution series to preformed complex of fluorescein-labeled Tau pS2 (10 nM) and 14-3-3ζ (100 nM) (which represents approximately 20% bound Tau pS2 peptide to 14-3-3ζ to allow further complex formation by addition of fragments[1]) in FA buffer (10 mM HEPES pH 7.4, 150 mM NaCl, 50 µM TCEP, 0.1% (v/v) Tween20, 0.1% (w/v) BSA). Dilution series were made in polystyrene low-volume 384-well plates (Corning #4514, Black Round Bottom). Measurements were performed directly after plate preparation, using a Tecan SPARK plate reader at room temperature (λex: 485±20 nm; λem: 535±25 nm; mirror: automatic; flashes: 30; settle time: 1 ms; gain: optimal; Z-position: calculated from well). Wells containing only fluorescein labelled peptide were used to set as G-factor calibrated from these wells. All data were analyzed using GraphPad Prism (10.0.1) and fitted with a four-parameter logistic model (4PL) to determine binding affinities (dissociation constant, KD). All data shown is background corrected for the signal without 14-3-3ζ and the mean and standard deviation of technical duplicate is plotted, assay performed in a scientific duplicate for hit compounds.

Fluorescence Anisotropy – 14-3-3 titrations in the presence of compound

To characterize the affinity of the Tau peptides for 14-3-3 in the presence of compound, 14-3-3ζ or γ was titrated in a 2-fold dilution series starting at 400 (for the 14-3-3ζ titrations to Tau pS2) or 800 (for the 14-3-3γ titrations to Tau pS214 or pS324) µM. 14-3-3 was added to 10 nM of fluorescein-labeled peptide (Tau pS2, Tau pS214 or Tau pS324) in the presence of 250 µM compound (1% DMSO). The assays were performed in a similar way as described above. Data shown is the average and standard deviation of a technical duplicate, top plateau corrected where needed, assay performed in a scientific duplicate.

Fluorescence Anisotropy – peptide screen

The peptide screen was performed in a similar way as the 14-3-3ζ titration in the precence of compound. 14-3-3ζ was titrated in a 2-fold dilution series (starting at 400 µM 14-3-3ζ) to 100 nM of fluorescein-labeled peptides and 250 µM compound **18** (1% DMSO) in FA buffer (10 mM HEPES pH 7.4, 150 mM NaCl, 50 µM TCEP, 0.1% (v/v) Tween20, 0.1% (w/v) BSA). For the Tau pS2 peptide, the initial data was used with 10 nM fluorescein-labeled peptide. Data shown is the average of a scientific triplicate.

Differential Scanning Fluorimetry (DSF)

DSF was performed using 40 µl samples containing 2.5 µM 14-3-3ζ, 25 µM Tau peptide (Tau pS2, Tau pS214 or Tau pS324), 75 µM Tau (1% DMSO) and 5x ProteoOrange (Lumiprobe, 5000x stock in DMSO) in 10 mM HEPES, 150 mM NaCl, 50 µM TCEP (pH 7.4). The samples were heated from 35 °C to 79°C at a rate of 0.3 °C per 15 s in a CFX96 Touch Real-Time PCR Detection System (Bio-Rad). Fluorescence intensity was determined using excitation (λem=525/20 nm) and emission (λex=570/20 nm) filters.

Based on these melting curves, the negative derivative melting curve is obtained from which the melting temperature Tm was determined. All described melting temperatures are based on two or three independent experiments, from which the average and standard deviations were determined.

X-ray crystallography data collection and refinement

For x-ray crystallography, a C-terminally truncated 14-3-3σ protein (truncated after T231 to reduce flexibility) was used. This 14-3-3σΔC was preincubated with Tau pS214 in complexation buffer (20 mM HEPES pH 7.5, 2 mM MgCl2 and 100 µM TCEP) in a 1:1.5 molar stoichiometry (protein:peptide) at a final protein concentration of 10.0 mg/mL. The protein-peptide complex was used to set up sitting drop crystallography plates in which protein-peptide complex was mixed in a 1:1 ratio (250 nL + 250 nL) with optimized crystallization buffer (0.095 M HEPES (pH 7.1), 0.19 M $CaCl_2$, 28 % (v/v) PEG 400 and 5% (v/v) glycerol). Crystals grew within 7 - 14 days at 4 °C. To fully grown crystals, a mixture of 0.4 µL of a 100 mM stock solution in DMSO within 3.6 µL optimized crystallization buffer was added. Crystals were soaked for minimal 1 week at 4 °C. Crystals were fished and flash-cooled in liquid nitrogen. X-ray diffraction (XRD) data were collected at the European Synchrotron Radiation Facility (ESRF), with beamlines ID30-A1, ID30B and ID23-1, Grenoble, France. autoPROC software (version 1.1.7) was used to index and integrate the diffraction data.[2] The data was further processed using the CCP4i2 suite (version 8.0.002).[3] AIMLESS was used for scaling the data.[4,5] The data was phased with MolRep[6,7] using protein data bank (PDB) entry 4FL5 as a template. Correct peptide sequences were modeled in the electron density in COOT (version 0.9.8.1).[8] For soaked crystal datasets, the presence of soaked fragments was verified by visual inspection of the Fo-Fc and 2Fo-Fc electron density maps in COOT. A three dimensional structure of each compound was generated using AceDRG7[9], which was thereafter built in based on visual inspection Fo-Fc and 2Fo-Fc electron density map. Finally, alternating cycles of model improvement (based on isotropic b-factors and standard set of stereo-chemical restraints: covalent bonds, angels, dihedrals, planarities, chiralities, non-bonded) and refinements were performed using COOT and REFMAC (version 5)[10,11] to improve the quality of the model. Figures were generated with PyMOL (version 2.5.2). 2Fo-Fc electron density maps were contoured at 1σ. See SI Table S2-3 for XRD data collection, structure determination, and refinement statistics. The structures were submitted to the PDB with IDs: 9FS4, 9GFA, 9FVP, 9FVH, 9FVI 9FVN and 9FVG.

Peptides

Acetylated and fluorescein (FITC)-labeled peptides for crystallography and fluorescence anisotropy were purchased from GenScript:

Peptide	Phosphorylation site(s)	Amino acid sequence
**Tau pS2**	S214 + S324	SRTP{pS}LPTPPTREGGGSGGGSGGG VTSKCG{pS}LGNIHHK
**Tau pS214**	S214	SRTP{pS}LPTPPTRE
**Tau pS324**	S324	VTSKCG{pS}LGNIHHK
**Pin1**	S72	LVKHSQSRRPS{pS}WRQEK
**C-Raf 233**	S233	QHRY{pS}TPHAF-$CONH_2$
**C-Raf 259**	S259	QRST{pS}TPNVH- $CONH_2$
**CFTR**	S753 + S768	AILPRI{pS}VISTGPTLQARRRQ{pS}VLNLMT
**ERRγ**	pS179	KRRRK(pS)CQA-$CONH_2$
**SOS1**	S1161	PRRRPE{pS}APAESS
**Rubic**	S248	SERRST{pS}FPLSGP
**Raptor**	S722	RLRSVS{pS}YGNIRA
**CDK11B**	S113	EKRRHR{pS}HSAEGG
**RND3**	S240	TDLRKDKAK{pS}CTVM-COOH
**p65**	S45	EGRSAG{pS}IPGRRS-$CONH_2$
**p53(-wt)**	pT387	KLMFK{pT}EGPDSD-COOH
**ERα**	T594	AEGFPA{pT}V-COOH

Synthetic procedures

2-phenyl-1*H*-imidazole (47)

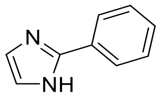

To a solution of benzaldehyde (250 mg, 2.36 mmol) in EtOH (8.5 mL), glyoxal (40% in H_2O, 376 mg, 2.59 mmol) and ammonia (25% in H_2O, 2.97 g, 21.2 mmol) were added. The reaction mixture was stirred for 3 days at room temperature. The reaction mixture was concentrated *in vacuo*. Saturated $NaHCO_3$ solution (aq., 15 ml) was added and the aqueous layer was extracted with EtOAc (3 x 20 ml). The combined extracts were dried with Na_2SO_4, and concentrated *in vacuo*. Flash column chromatography of the residue (12 g silica, gradient CH_2Cl_2/MeOH (0% → 20%)) afforded imidazole **47** as a slightly yellow solid (111 mg, 771 µmol, 33%).
TLC: R_f = 0.29 (MeOH/CH_2Cl_2, 1:19).
^{1}H-NMR (400 MHz, $CDCl_3$): δ = 9.89 (s, 1H), 7.87 – 7.82 (m, 2H), 7.44 – 7.39 (m, 2H), 7.38 – 7.33 (m, 1H), 7.16 (s, 2H) ppm.
^{13}C-NMR (100 MHz, $CDCl_3$): δ = 146.7, 130.2, 128.9, 128.7, 125.2 ppm.

2-phenyl-1*H*-imidazole (47)

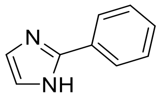

Glyoxal (342 mg, 2.33 mmol), ammonium acetate (1.27 g, 16.49 mmol), and triethylammonium acetate (238 mg, 1.48 mmol) were added to benzaldehyde (250 mg, 2.33 mmol). The reaction mixture was stirred for 2 h at 120 °C under reflux. Saturated $NaHCO_3$ solution (aq., 10 ml) and saturated NaCl (aq., 15 ml) was added and the aqueous layer was extracted with EtOAc (3 x 25 ml). The combined extracts were dried with $MgSO_4$, and concentrated *in vacuo.* Flash column chromatography of the residue (12 g silica, gradient CH_2Cl_2/MeOH (0% → 10%)) afforded imidazole **47** as a slightly red solid (60.0 mg, 416 µmol, 18%).
TLC: R_f = 0.27 (MeOH/CH_2Cl_2, 1:19).
^{1}H-NMR (399 MHz, DMSO-d$_6$): δ = 12.55 (s, 1H), 8.01 – 7.95 (m, 2H), 7.45 (t, *J* = 7.8 Hz, 2H), 7.36 – 7.31 (m, 1H), 7.16 (s, 2H) ppm.
^{13}C-NMR (100 MHz, DMSO-d$_6$): δ = 146.1, 131.4, 129.2, 128.4, 125.3 ppm.

2-phenyl-4,5-dihydro-1*H*-imidazole (48)

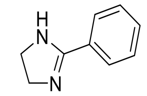

To a solution of benzaldehyde (250 mg, 2.33 mmol) in CH_2Cl_2 (24 ml), ethylenediamine (149 mg, 2.47 mmol) was added. The reaction mixture was stirred for 20 min at 0 °C after which NBS (440 mg, 2.22 mmol) and K_2CO_3 (743 mg, 5.34 mmol) were added. The reaction mixture was then stirred overnight at room temperature. Saturated $NaHCO_3$ solution (aq., 15 ml) was added

to the reaction mixture. The aqueous layer was extracted with CH_2Cl_2 (3 x 25 ml). The combined organic extracts were dried with $MgSO_4$, and concentrated *in vacuo*. Flash column chromatography of the residue (4 g silica, gradient CH_2Cl_2/MeOH (0% → 10%, 1% NEt_3)) afforded imidazole **48** as a slightly yellow solid (108 mg, 741 µmol, 32%).
TLC: R_f = 0.31 (MeOH/CH_2Cl_2, 1:9).
^{1}H-NMR (400 MHz, $CDCl_3$): δ = 7.83 – 7.75 (m, 2H), 7.32 – 7.30 (m, 1H), 6.90 (s, 2H), 3.65 (s, 4H) ppm.

2-(3'-chlorophenyl)-4,5-dihydro-1*H*-imidazole (49)

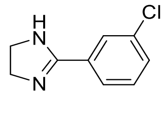

To a solution of 3-chlorobenzaldehyde (250 mg, 1.78 mmol) in t-BuOH (18 ml), ethylenediamine (118 mg, 1.96 mmol) was added. The reaction mixture was stirred for 30 min at room temperature after which I_2 (564 mg, 2.22 mmol) and K_2CO_3 (743 mg, 5.34 mmol) were added. The reaction mixture was then stirred overnight at 70 °C. Saturated Na_2SO_3 solution (aq., 25ml) was added to the reaction mixture. The aqueous layer was extracted with $CHCl_3$ (3 x 25 ml). The combined organic extracts were washed with saturated NaCl solution (aq., 3 ml), dried with $MgSO_4$, and concentrated *in vacuo* to afford imidazoline **49**. Imidazoline **49** was used in the next step without further purification.
TLC: R_f = 0.29 (MeOH/CH_2Cl_2, 1:19).
^{1}H-NMR (400 MHz, $CDCl_3$): δ = 7.78 (t, J = 1.9 Hz, 1H), 7.66 (dt, J = 7.7, 1.4 Hz, 1H), 7.42 (ddd, J = 8.0, 2.1, 1.2 Hz, 1H), 7.34 (t, J = 7.9 Hz, 1H), 3.80 (s, 4H) ppm.

2-(3'-chlorophenyl)-1*H*-imidazole (50)

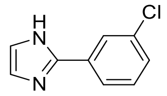

Imidazoline (**49**) (345 mg, 1.91 mmol), K_2CO_3 (290 mg, 2.10 mmol) and (diacetoxyiodo)benzene (677 mg, 2.10 mmol) was added to DMSO (19 ml). The reaction mixture was stirred overnight at room temperature. The reaction mixture was diluted with saturated $NaHCO_3$ solution (aq., 15 ml) and EtOAc (100 ml), and was stirred for an additional 10 min. The organic layer was extracted with saturated $NaHCO_3$ solution (aq., 20 ml) and saturated NaCl solution (aq., 20 ml). The organic extract was dried with $MgSO_4$ and concentrated *in vacuo*. Flash column chromatography of the residue (12 g silica, gradient CH_2Cl_2/MeOH (0% → 10%)) afforded imidazole **50** as a slightly yellow solid (203 mg, 1.14 mmol, 60%).
TLC: R_f = 0.48 (MeOH/CH_2Cl_2, 1:19).
^{1}H-NMR (399 MHz, DMSO-d_6): δ = 12.65 (s, 1H), 7.99 (t, J = 1.9 Hz, 1H), 7.91 (ddd, J = 7.8, 1.6, 1.1 Hz, 1H), 7.48 (t, J = 7.9 Hz, 1H), 7.39 (ddd, J = 8.0, 2.1, 1.1 Hz, 1H), 7.18 (d, J = 92.9 Hz, 2H) ppm.
^{13}C-NMR (100 MHz, DMSO-d_6): δ = 144.6, 134.0, 133.3, 131.2, 128.0, 124.8, 123.7, 118.8 ppm.

8-(1*H*-imidazol-2-yl)quinoline (AO-51)

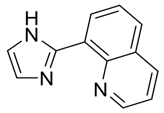

To a solution of quinoline-8-carbaldehyde (200 mg, 1.27 mmol) in *t*BuOH (13 ml), ethylenediamine (93.5 µL, 1.40 mmol), K_2CO_3 (528 mg, 3.82 mmol), and I_2 (404 mg, 1.59 mmol) were added. The reaction mixture was stirred for 3 h at 70 ° Saturated Na_2SO_3 solution (aq., 20 ml) was added to the reaction mixture. The aqueous layer was extracted with CH_2Cl_2 (3 x 30 ml). The combined organic extracts were dried with $MgSO_4$, and concentrated *in vacuo*. The residue was solved in DMSO (12 mL). Diacetoxyiodobenzene (451 mg, 1.40 mmol) and K_2CO_3 (193 mg, 1,40 mmol) were added and the mixture was stirred 16 h at room temperature. Saturated Na_2CO_3 solution (aq., 20 mL) was added. The aqueous layer was extracted with EtOAc (3 x 30 ml). The combined organic extracts were dried with $MgSO_4$, and concentrated *in vacuo*. Flash column chromatography of the residue (24 g silica, gradient heptane/EtOAc (8% → 80%) afforded imidazole **51** as a slightly yellow solid (85.5 mg, 438 µmol, 34%).
^{1}H-NMR (400 MHz, $CDCl_3$): δ = 8.83 – 8.79 (m, 2H), 8.11 (dd, *J* = 8.3, 1.8 Hz, 1H), 7.67 (dd, *J* = 8.1, 1.5 Hz, 1H), 7.60 – 7.54 (m, 1H), 7.35 (dd, *J* = 8.3, 4.3 Hz, 1H) ppm.

5-(1H-imidazol-2-yl)isoquinoline (AO-52)

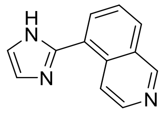

To a solution of quinoline-5-carbaldehyde (100 mg, 637 µmol) in *t*BuOH (13 ml), ethylenediamine (46.7 µL, 700 µmol), K_2CO_3 (264 mg, 1.91 mmol), and I_2 (202 mg, 795 µmol) were added. The reaction mixture was stirred for 3 h at 70 ° Saturated Na_2SO_3 solution (aq., 20 ml) was added to the reaction mixture. The aqueous layer was extracted with CH_2Cl_2 (3 x 30 ml). The combined organic extracts were dried with $MgSO_4$, and concentrated *in vacuo*. The residue was solved in DMSO (12 mL). Diacetoxyiodobenzene (225 mg, 700 µmol) and K_2CO_3 (96.7 mg, 700 µmol) were added and the mixture was stirred 16 h at room temperature. Saturated Na_2CO_3 solution (aq., 20 mL) was added. The aqueous layer was extracted with EtOAc (3 x 30 ml). The combined organic extracts were dried with $MgSO_4$, and concentrated *in vacuo*. Flash column chromatography of the residue (24 g silica, gradient heptane/EtOAc (8% → 80%) afforded imidazole **52** as a slightly yellow solid (42.5 mg, 218 µmol, 34%).
^{1}H-NMR (400 MHz, DMSO-d_6): δ = 12.70 (s, 1H), 9.36 (d, *J* = 1.1 Hz, 1H), 9.08 (dd, *J* = 6.1, 1.0 Hz, 1H), 8.56 (d, *J* = 6.1 Hz, 1H), 8.18 – 8.09 (m, 2H), 7.77 (dd, *J* = 8.1, 7.3 Hz, 1H), 7.30 (s, 2H) ppm.

2-(o-tolyl)-4,5-dihydro-1*H*-imidazole (53)

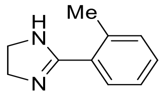

To a solution of 1-methylbenzaldehyde (250 mg, 2.08 mmol) in t-BuOH (20.8 ml), ethylenediamine (138 mg, 2.29 mmol) was added. The reaction mixture was stirred for 30 min at room temperature after which I_2 (660 mg, 2.60 mmol) and K_2CO_3 (863 mg, 6.24 mmol) were

added. The reaction mixture was then stirred at 70 °C for 2.5 h and at room temperature overnight. Saturated Na_2SO_3 solution (aq., 15 ml) and saturated $NaHCO_3$ solution (aq., 10 ml) were added. The aqueous layer was extracted with EtOAc (3 x 30 ml). The combined organic extracts were washed with saturated NaCl solution (aq., 5 ml), dried with $MgSO_4$, and concentrated *in vacuo* to afford imidazoline **53**. Imidazoline **53** was used in the next step without further purification.
TLC: R_f = 0.22 (MeOH/CH_2Cl_2, 1:19).

2-(o-tolyl)-1*H*-imidazole (54)

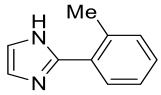

Imidazoline (**53**) (333 mg, 2.08 mmol), K_2CO_3 (316 mg, 2.29 mmol) and (diacetoxyiodo)benzene (737 mg, 2.29 mmol) was added to DMSO (20.8 ml). The reaction mixture was stirred overnight at room temperature. The reaction mixture was diluted with saturated $NaHCO_3$ solution (aq., 15 ml) and EtOAc (100 ml), and was stirred for an additional 10 min. The organic layer was extracted with saturated $NaHCO_3$ solution (aq., 20 ml) and saturated NaCl solution (aq., 20 ml). The organic extract was dried with $MgSO_4$ and concentrated *in vacuo*. Flash column chromatography of the residue (12 g silica, gradient CH_2Cl_2/MeOH (0% → 5%)) afforded imidazole **54** as a slightly yellow oil (89.0 mg, 563 μmol, 27%).
TLC: R_f = 0.29 (MeOH/CH_2Cl_2, 1:19).
^{1}H-NMR (399 MHz, $CDCl_3$): δ = 7.37 (d, 1H), 7.23 – 7.14 (m, 3H), 7.08 (td, *J* = 7.4, 1.7 Hz, 1H), 6.89 (s, 2H), 2.36 (s, 3H) ppm.
^{13}C-NMR (100 MHz, DMSO-d_6): δ = 146.4, 136.3, 131.5, 131.0, 128.8, 128.4, 126.2, 21.6 ppm.

2-(m-tolyl)-4,5-dihydro-1*H*-imidazole (55)

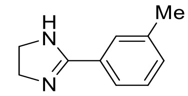

To a solution of 3-methylbenzaldehyde (250 mg, 2.08 mmol) in *t*-BuOH (20.8 ml), ethylenediamine (138 mg, 2.29 mmol) was added. The reaction mixture was stirred for 30 min at room temperature after which I_2 (660 mg, 2.60 mmol) and K_2CO_3 (863 mg, 6.24 mmol) were added. The reaction mixture was then stirred overnight at 70 °C. Saturated Na_2SO_3 solution (aq., 25 ml) and saturated $NaHCO_3$ solution (aq., 10 ml) were added to the reaction mixture and the aqueous layer was extracted with EtOAc (3 x 25 ml). The combined organic extracts were washed with saturated NaCl solution (aq., 10 ml), dried over $MgSO_4$, and concentrated *in vacuo* to afford imidazoline **55**. Imidazoline **55** was used in the next step without further purification.
TLC: R_f = 0.22 (MeOH/CH_2Cl_2, 1:19).

2-(*m*-tolyl)-1*H*-imidazole (56)

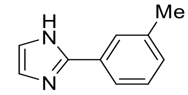

Imidazoline (**55**) (333 mg, 2.08 mmol), K_2CO_3 (316 mg, 2.29 mmol) and (diacetoxyiodo)benzene (737 mg, 2.29 mmol) was added to DMSO (21 ml). The reaction mixture was stirred overnight at room temperature. The reaction mixture was diluted with saturated $NaHCO_3$ solution (aq., 15 ml) and EtOAc (100 ml), and was stirred for an additional 10 min. The organic layer was extracted with saturated $NaHCO_3$ solution (aq., 20 ml) and saturated NaCl solution (aq., 20 ml). The organic extract was dried with $MgSO_4$ and concentrated *in vacuo*. Flash column chromatography of the residue (12 g silica, gradient CH_2Cl_2/MeOH (0% → 5%)) afforded imidazole **56** as a slightly yellow solid (98.0 mg, 619 µmol, 30%).
TLC: R_f = 0.23 (MeOH/CH_2Cl_2, 1:19).
^{1}H-NMR (399 MHz, DMSO-d$_6$): δ = 12.44 (s, 1H), 7.79 – 7.74 (m, 1H), 7.72 (d, 1H), 7.32 (t, J = 6.7 Hz, 2H), 7.22 (s, 1H), 7.14 (dt, J = 7.4, 0.9 Hz, 1H), 7.00 (s, 1H), 2.35 (s, 3H) ppm.
^{13}C-NMR (100 MHz, DMSO-d$_6$): δ = 146.1, 138.2, 131.3, 129.4, 129.1, 129.0, 125.8, 122.4, 118,0 21.6 ppm.

2-(3'-methoxyphenyl)-4,5-dihydro-1*H*-imidazole (57)

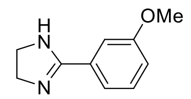

To a solution of 2-methoxybenzaldehyde (250 mg, 1.84 mmol) in t-BuOH (18.4 ml), ethylenediamine (121 mg, 2.02 mmol) was added. The reaction mixture was stirred for 30 min at room temperature after which I_2 (582 mg, 2.30 mmol) and K_2CO_3 (761 mg, 5.51 mmol) were added. The reaction mixture was then stirred overnight at 70 °C. Saturated Na_2SO_3 solution (aq., 25ml) was added to the reaction mixture. The aqueous layer was extracted with $CHCl_3$ (3 x 25 ml). The combined organic extracts were washed with saturated NaCl solution (aq., 3 ml), dried with $MgSO_4$, and concentrated *in vacuo* to afford imidazoline **57**. Imidazoline **57** was used in the next step without further purification.
TLC: R_f = 0.31 (MeOH/CH_2Cl_2, 1:19).

2-(3'-methoxyphenyl)-1*H*-imidazole (58)

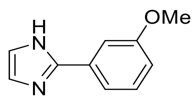

Imidazoline (**57**) (237 mg, 1.34 mmol), K_2CO_3 (223 mg, 1.61 mmol) and (diacetoxyiodo)benzene (519 mg, 1.61 mmol) was added to DMSO (13.4 ml). The reaction mixture was stirred overnight at room temperature. The reaction mixture was diluted with saturated $NaHCO_3$ solution (aq., 15 ml) and EtOAc (100 ml), and was stirred for an additional 10 min. The organic layer was extracted with saturated $NaHCO_3$ solution (aq., 20 ml) and saturated NaCl solution (aq., 20 ml). The organic extract was dried with $MgSO_4$ and concentrated *in vacuo*. Flash column chromatography of the residue (12 g silica, gradient CH_2Cl_2/MeOH (0% → 5%)) afforded imidazole **58** as a slightly yellow solid (101 mg, 579 µmol, 43%).
TLC: R_f = 0.41 (MeOH/CH_2Cl_2, 1:19).

^{1}H-NMR (399 MHz, DMSO-d$_6$) δ = 12.49 (s, 1H), 7.55 – 7.49 (m, 2H), 7.34 (t, J = 8.2 Hz, 1H), 7.24 (s, 1H), 7.01 (s, 1H), 6.93 – 6.86 (m, 1H), 3.81 (s, 3H) ppm.
^{13}C-NMR (100 MHz, DMSO-d$_6$): δ = 160.0, 145.9, 132.6, 130.3, 129.4, 118.2, 117.6, 114.2, 110.5, 55.6 ppm.

General Procedure 1 (GP 1)

A solution of aldehyde (1 eq.), glyoxal (1.2 eq.; 40% aq.), and NH_3 (5 eq.; 25% aq.) in MeOH (0.1 mol/L) was stirred at room temperature for 4-7 h. MeOH was evaporated under reduced pressure. Water was added and the aqueous layer was extracted with EtOAc (4 x). The combined organic extracts were treated with Na_2SO_4 and evaporated under reduced pressure. Automated flash column chromatography (silica, CH_2Cl_2/MeOH) of the residue afforded the corresponding 2-substituted imidazole.

2-(2-bromothiophen-3-yl)-1*H*-imidazole (59)

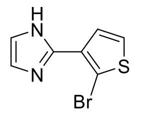

According to GP1, imidazole **59** was synthesized with 13% yield (76.1 mg, 322 µmol). Purification was performed by FCC (24 g silica, heptane/EtOAc 8% → 80%).
^{1}H-NMR (399 MHz, $CDCl_3$): δ = 10.36 (s, 1H), 7.25 (d, J = 5.4 Hz, 1H), 7.14 (s, 2H), 6.99 (d, J = 5.2 Hz, 1H) ppm.
^{13}C{^{1}H}-NMR (101 MHz, $CDCl_3$): δ = 140.6, 131.2, 130.8, 129.5, 126.3, 116.6, 105.8 ppm.

2-(5-bromofuran-2-yl)-1*H*-imidazole (60)

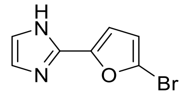

According to GP1, imidazole **60** was synthesized with 77% yield (201 mg, 944 µmol). Purification was performed by FCC (24 g silica, heptane/EtOAc 8% → 80%).
^{1}H-NMR (399 MHz, DMSO-d$_6$): δ = 12.68 – 12.56 (m, 1H), 7.20 (s, 1H), 7.01 (s, 1H), 6.82 (d, J = 3.5 Hz, 1H), 6.70 (d, J = 3.4 Hz, 1H) ppm.
^{13}C{^{1}H}-NMR (101 MHz, DMSO-d$_6$): δ = 148.4, 137.5, 129.0, 121.0, 117.5, 113.7, 108.8 ppm.

8-(1*H*-imidazol-2-yl)isoquinoline (61)

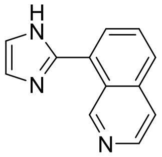

According to GP1, imidazole **61** was synthesized with 29% yield (54.9 mg, 281 µmol). Purification was performed by FCC (12 g silica, CH_2Cl_2/MeOH 2% → 10%).

^{1}H-NMR (399 MHz, $CDCl_3$): δ = 9.81 (s, 1H), 8.38 (d, J = 5.7 Hz, 1H), 7.75 (d, J = 8.3 Hz, 1H), 7.73 – 7.70 (m, 1H), 7.62 (dd, J = 6.6, 5.4 Hz, 2H), 7.19 (s, 2H), 6.98 – 6.96 (m, 1H) ppm.
^{13}C{^{1}H}-NMR (100 MHz, $CDCl_3$): δ = 150.8, 144.3, 141.9, 136.3, 134.9, 130.0, 129.0, 128.1, 127.3, 125.8, 123.5, 121.5, 120.9 ppm.

2-(benzofuran-7-yl)-1*H*-imidazole (62)

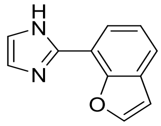

According to GP1, imidazole **62** was synthesized with 56% yield (106 mg, 576 µmol). Purification was performed by FCC (24 g silica, CH_2Cl_2/MeOH 2% → 10%).
^{1}H-NMR (399 MHz, $CDCl_3$): δ = 10.54 (s, 1H), 8.13 (dd, J = 7.7, 1.3 Hz, 1H), 7.62 (d, J = 2.2 Hz, 1H), 7.53 (dd, J = 7.8, 1.3 Hz, 1H), 7.27 (t, J = 7.7 Hz, 1H), 7.21 (s, 2H), 6.79 (d, J = 2.2 Hz, 1H) ppm.
^{13}C{^{1}H}-NMR (100 MHz, $CDCl_3$): δ = 150.7, 144.8, 143.2, 127.9, 123.6, 123.0, 122.0, 121.4, 114.7, 107.2 ppm.

2-(benzo[b]thiophen-4-yl)-1*H*-imidazole (63)

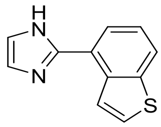

According to GP1, imidazole **63** was synthesized with 34% yield (46.9 mg, 234 µmol). Purification was performed by FCC (12 g silica, heptane/EtOAc 8% → 80%).
^{1}H-NMR (399 MHz, DMSO-d_6): δ = 12.60 (s, 1H), 8.58 – 8.53 (m, 1H), 8.01 (d, J = 8.0 Hz, 1H), 7.84 – 7.82 (m, 1H), 7.81 (d, J = 1.3 Hz, 1H), 7.45 (t, J = 7.7 Hz, 1H), 7.32 (s, 1H), 7.16 (s, 1H) ppm.
^{13}C{^{1}H}-NMR (100 MHz, DMSO-d_6): δ = 145.3, 140.6, 136.0, 129.2, 127.4, 125.8, 125.1, 124.1, 122.4, 121.9, 117.7 ppm.

tert-butyl 4-(1H-imidazol-2-yl)piperidine-1-carboxylate (64)

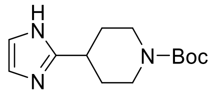

According to GP1, imidazole **64** was synthesized with 53% yield (312 mg, 1.24 mmol). Purification was performed by FCC (12 g silica, heptane/EtOAc 8% → 80%).
^{1}H-NMR (399 MHz, $CDCl_3$): δ = 6.95 (s, 2H), 4.13 (s, 2H), 2.92 (tt, J = 11.7, 3.7 Hz, 1H), 2.82 (s, 2H), 2.01 – 1.95 (m, 2H), 1.71 (dtd, J = 13.3, 12.0, 4.3 Hz, 2H), 1.44 (s, 9H) ppm.
^{13}C{^{1}H}-NMR (100 MHz, $CDCl_3$): δ = 154.9, 151.0, 121.6, 79.9, 43.6, 36.2, 31.0, 28.6 ppm.

tert-butyl (S)-2-(1H-imidazol-2-yl)pyrrolidine-1-carboxylate (65)

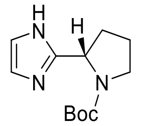

According to GP1, imidazole **65** was synthesized with 44% yield (261 mg, 1.10 mmol). Purification was performed by FCC (12 g silica, heptane/EtOAc 8% → 100%).

^{1}H-NMR (400 MHz, $CDCl_3$): δ = 10.48 (s, 1H), 6.94 (s, 2H), 4.92 (dd, J = 7.7, 2.4 Hz, 1H), 3.37 (dd, J = 8.1, 4.8 Hz, 2H), 2.91 (dt, J = 6.0, 2.8 Hz, 1H), 2.10 (qd, J = 10.1, 9.5, 5.0 Hz, 2H), 1.94 (dt, J = 5.3, 2.8 Hz, 1H), 1.47 (s, 9H) ppm.

^{13}C{^{1}H}-NMR (101 MHz, $CDCl_3$): δ = 156.6, 149.0, 127.0, 116.4, 80.4, 54.1, 47.4, 28.6, 28.1, 25.0 ppm.

4-(4-(1H-imidazol-2-yl)phenyl)morpholine (66)

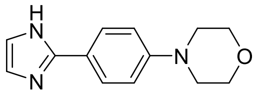

According to GP1, imidazole **66** was synthesized with 38% yield (136 mg, 593 µmol). Purification was performed by FCC (12 g silica, CH_2Cl_2/MeOH 2% → 10%).

^{1}H-NMR (400 MHz, DMSO-d_6): δ = 12.21 (s, 1H), 7.78 (d, J = 8.6 Hz, 2H), 7.16 – 6.88 (m, 4H), 3.74 (sb, 4H), 3.15 (sb, 4H) ppm.

^{13}C{^{1}H}-NMR (101 MHz, DMSO-d_6): δ = 150.6, 145.9, 125.7, 121.9, 114.7, 66.0, 48.0 ppm.

2-(3-(methylsulfonyl)phenyl)-1H-imidazole (67)

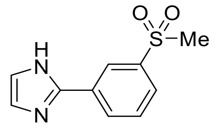

According to GP1, imidazole **67** was synthesized with 25% yield (91.5 mg, 412 µmol). Purification was performed by FCC (12 g silica, CH_2Cl_2/MeOH 2% → 10%).

^{1}H-NMR (400 MHz, $CDCl_3$): δ = 8.36 (t, J = 1.8 Hz, 1H), 8.14 (dt, J = 7.8, 1.3 Hz, 1H), 7.77 (dt, J = 7.9, 1.2 Hz, 1H), 7.47 (t, J = 7.9 Hz, 1H), 7.16 (s, 2H), 2.97 (s, 3H) ppm.

^{13}C{^{1}H}-NMR (101 MHz, $CDCl_3$): δ = 145.1, 141.0, 132.0, 130.8, 130.1, 126.7, 124.0, 44.3 ppm.

2-(benzofuran-4-yl)-1H-imidazole (68)

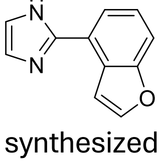

According to GP1, imidazole **68** was synthesized with 26% yield (32.2 mg, 175 µmol). Purification was performed by FCC (12 g silica, CH_2Cl_2/MeOH 2% → 10%).

^{1}H-NMR (400 MHz, $CDCl_3$:methanol-d_4 = 3:1): δ = 7.68 (d, J = 2.3 Hz, 1H), 7.55 (dd, J = 7.6, 1.0 Hz, 1H), 7.47 (dt, J = 8.3, 0.9 Hz, 1H), 7.35 – 7.29 (m, 2H), 7.13 (d, J = 4.6 Hz, 2H) ppm.

$^{13}C\{^1H\}$**-NMR** (101 MHz, $CDCl_3$:methanol-d_4 = 3:1): δ = 155.3, 145.9, 145.5, 124.8, 124.0, 123.5, 122.8, 120.4, 111.4, 106.5 ppm.

2-(4-chlorophenyl)-1*H*-imidazole (69)

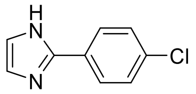

According to GP1, imidazole **69** was synthesized with 33% yield (213 mg, 1.19 mmol). Purification was performed by FCC (12 g silica, CH_2Cl_2/MeOH 2% → 10%).
^1H**-NMR** (400 MHz, methanol-d_4): δ = 7.85 – 7.80 (m, 2H), 7.47 – 7.42 (m, 2H), 7.14 (s, 2H) ppm.
$^{13}C\{^1H\}$**-NMR** (101 MHz, methanol-d_4): δ = 146.9, 135.5, 130.2, 130.1, 127.8 ppm.

2-(4-iodophenyl)-1*H*-imidazole (70)

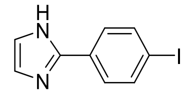

According to GP1, imidazole **70** was synthesized with 43% yield (149 mg, 552 µmol). Purification was performed by FCC (12 g silica, CH_2Cl_2/MeOH 2% → 10%). Imidazole was used in the subsequent step without further characterization.

2-(4-fluorophenyl)-1*H*-imidazole (71)

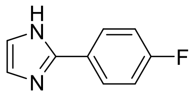

According to GP1, imidazole **71** was synthesized with 38% yield (246 mg, 1.52 mmol). Purification was performed by FCC (12 g silica, CH_2Cl_2/MeOH 2% → 10%).
^1H**-NMR** (400 MHz, $CDCl_3$:methanol-d_4 = 4:1): δ = 7.84 (dd, *J* = 8.9, 5.2 Hz, 2H), 7.11 (t, *J* = 8.8 Hz, 2H), 7.07 (s, 2H) ppm.
$^{13}C\{^1H\}$**-NMR** (101 MHz, $CDCl_3$:methanol-d_4 = 4:1): δ = 164.4, 161.9, 146.2, 127.5, 127.4, 126.8, 126.8, 116.1, 115.9 ppm.

2-(4-ethylphenyl)-1*H*-imidazole (72)

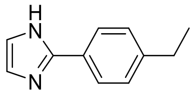

According to GP1, imidazole **72** was synthesized with 51% yield (658 mg, 3.82 mmol). Purification was performed by FCC (12 g silica, CH_2Cl_2/MeOH 2% → 10%).
^1H**-NMR** (400 MHz, DMSO-d_6): δ = 12.41 (s, 1H), 7.88 – 7.82 (m, 2H), 7.30 – 7.24 (m, 2H), 7.10 (s, 2H), 2.62 (q, *J* = 7.6 Hz, 2H), 1.19 (t, *J* = 7.6 Hz, 3H) ppm.
$^{13}C\{^1H\}$**-NMR** (101 MHz, DMSO-d_6): δ = 145.7, 143.6, 128.5, 128.0, 124.8, 27.9, 15.4 ppm.

2-(*p*-tolyl)-1*H*-imidazole (73)

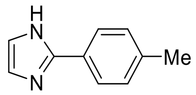

According to GP2, 1-2-disubstituted imidazole **73** was synthesized with 41% yield (271 mg, 1.71 mmol). Purification was performed by FCC (12 g CH_2Cl_2/MeOH 0% → 8%).
TLC: R_f = 0.29 (MeOH/CH_2Cl_2, 1:19).
^{1}H-NMR (399 MHz, DMSO-d$_6$): δ = 12.38 (s, 1H), 7.81 (d, J = 8.1 Hz, 2H), 7.24 (d, J = 7.9 Hz, 2H), 7.20 (s, 1H), 6.98 (s, 1H), 2.32 (s, 3H) ppm.
^{13}C-NMR (100 MHz, DMSO-d$_6$): δ = 146.1, 137.7, 129.7, 129.2, 128.7, 125.2, 117.8, 21.3 ppm.

2-(2'-methoxyphenyl)-1*H*-imidazole (74)

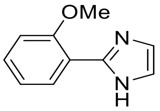

According to GP1, imidazole **74** was synthesized with 56% yield (359 mg, 2.06 mmol). Purification was performed by FCC (12 g silica, CH_2Cl_2/MeOH 0% → 8%).
TLC: R_f = 0.30 (MeOH/CH_2Cl_2, 1:19).
^{1}H-NMR (399 MHz, DMSO-d$_6$): δ = 11.78 (s, 1H), 8.10 (dd, J = 7.8, 1.8 Hz, 1H), 7.32 (ddd, J = 8.6, 7.4, 1.8 Hz, 1H), 7.19 – 7.10 (m, 2H), 7.07 – 6.98 (m, 2H), 3.94 (s, 3H) ppm.
^{13}C-NMR (100 MHz, DMSO-d$_6$): δ = 155.9, 143.3, 129.6, 128.5, 128.4, 121.1, 119.4, 117.7, 112.2, 55.9 ppm.

2-(2'-chlorophenyl)-1*H*-imidazole (75)

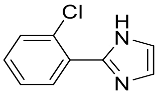

According to GP1, imidazole **75** was synthesized with 53% yield (328 mg, 1.89 mmol). Purification was performed by FCC (12 g silica, cyclohexane/EtOAc 0% → 100%).
TLC: R_f = 0.42 (MeOH/CH_2Cl_2, 1:19).
^{1}H-NMR (399 MHz, DMSO-d$_6$): δ = 12.28 (s, 1H), 7.82 – 7.75 (m, 1H), 7.59 – 7.52 (m, 1H), 7.46 – 7.37 (m, 2H), 7.17 (d, J = 79.4 Hz, 2H) ppm.
^{13}C-NMR: (100 MHz, DMSO-d$_6$): δ = 143.4, 131.6, 131.1, 130.7, 130.6, 130.3, 129.2, 127.7, 118.5 ppm.

2-(4'-methoxyphenyl)-1*H*-imidazole (76)

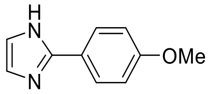

According to GP1, imidazole **76** was synthesized with 27% yield (174 mg, 998 µmol). Purification was performed by FCC (24 g silica, CH_2Cl_2/MeOH 0% → 5%).
TLC: R_f = 0.36 (MeOH/CH_2Cl_2, 1:19).
^{1}H-NMR (399 MHz, DMSO-d$_6$): δ = 12.32 (s, 1H), 7.91 – 7.82 (m, 2H), 7.23 – 6.92 (m, 4H), 3.79 (s, 3H) ppm.
^{13}C-NMR (100 MHz, DMSO-d$_6$): δ = 159.6, 146.1, 129.0, 126.7, 124.2, 117.5, 114.6, 55.6 ppm.

3-(1'*H*-imidazol-2'-yl)benzamide (77)

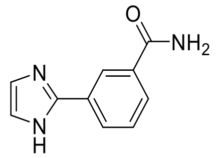

According to GP1, imidazole **77** was synthesized with 53% yield (100 mg, 535 µmol). Purification was performed by FCC (4 g silica, CH_2Cl_2/MeOH 0% → 18%).
TLC: R_f = 0.47 (MeOH/CH_2Cl_2, 1:19).
^{1}H-NMR (399 MHz, DMSO-d_6): δ = 12.59 (s, 1H), 8.51 (t, *J* = 1.8 Hz, 1H), 8.11 (dt, *J* = 7.8, 1.5 Hz, 1H), 8.08 (s, 1H), 7.86 (dt, *J* = 7.9, 1.4 Hz, 1H), 7.57 (t, *J* = 7.7 Hz, 1H), 7.48 (s, 1H), 7.21 (s, 2H) ppm.
^{13}C-NMR (100 MHz, DMSO-d_6): δ = 168.3, 145.6, 135.6, 135.4, 131.4, 129.1, 127.8, 127.1, 124.7 ppm.

4-(1'*H*-imidazol-2'-yl)benzamide (78)

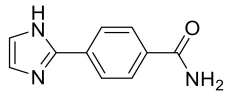

According to GP1, imidazole **78** was synthesized with 36% yield (68.5 mg, 366 µmol). Purification was performed by FCC (12 g silica, CH_2Cl_2/MeOH 3% → 22%).
TLC: R_f = 0.42 (MeOH/CH_2Cl_2, 1:19).
^{1}H-NMR (399 MHz, DMSO-d_6): δ = 12.64 (s, 1H), 8.00 (d, *J* = 8.3 Hz, 3H), 7.94 (d, *J* = 8.3 Hz, 2H), 7.39 (s, 1H), 7.19 (s, 2H) ppm.
^{13}C-NMR (100 MHz, DMSO-d_6): δ = 167.9, 145.3, 133.73, 133.66, 129.8, 128.6, 128.5, 124.8 ppm.

2-(4'-isopropylphenyl)-1*H*-imidazole (79a)

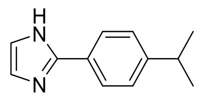

According to GP1, imidazole **79a** was synthesized with 41% yield (256 mg, 1.37 mmol). Purification was performed by FCC (24 g silica, heptane/EtOAc 10% → 100%).
TLC: R_f = 0.33 (hexane:EtOAc, 1:1).
^{1}H-NMR (399 MHz, DMSO-d_6): δ = 12.41 (s, 1H), 7.85 (d, *J* = 8.0 Hz, 2H), 7.30 (d, *J* = 8.1 Hz, 2H), 7.10 (s, 2H), 2.90 (hept, *J* = 6.9 Hz, 1H), 1.22 (d, *J* = 6.9 Hz, 6H) ppm.
^{13}C-NMR (100 MHz, DMSO-d_6): δ = 148.1, 145.6, 128.5, 126.5, 124.7, 33.1, 23.7 ppm.

2-(2`-chloro-4'-methyl-phenyl)-1*H*-imidazole (79b)

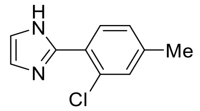

According to GP1, imidazole **79b** was synthesized with 44% yield (277 mg, 1.44 mmol). Purification was performed by FCC (24 g silica, heptane/EtOAc 5% → 75%).

TLC: R_f = 0.36 (hexane:EtOAc, 1:1).
^{1}H-NMR (399 MHz, $CDCl_3$): δ = 7.67 (d, J = 7.9 Hz, 1H), 7.31 (d, J = 55.4 Hz, 2H), 7.13 (d, J = 69.9 Hz, 2H), 2.34 (s, 3H) ppm.
^{13}C-NMR (100 MHz, $CDCl_3$): δ = 148.2, 145.0, 136.1, 135.6, 135.5, 133.7, 133.2, 132.5, 123.0, 25.6 ppm.

2-(4'-(1''*H*-imidazol-2''-yl)phenoxy)ethan-1-ol (80)

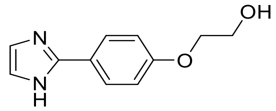

According to GP1, imidazole **80** was synthesized with 29% yield (175 mg, 1.44 mmol). Purification was performed by FCC (24 g silica, CH_2Cl_2/MeOH 0% → 11%).
TLC: R_f = 0.51 (MeOH/CH_2Cl_2, 1:9).
^{1}H-NMR (399 MHz, DMSO-d_6): δ = 12.22 (s, 1H), 7.88 – 7.81 (m, 2H), 7.07 (s, 2H), 7.02 – 6.98 (m, 2H), 4.02 (dd, J = 5.4, 4.6 Hz, 2H), 3.73 (t, J = 5.0 Hz, 2H) ppm.
^{13}C-NMR (100 MHz, DMSO-d_6): δ = 159.0, 146.1, 135.6, 126.7, 124.1, 115.1, 70.0, 60.0 ppm.

2-(4-(trifluoromethyl)phenyl)-1*H*-imidazole (81)

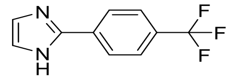

According to GP1, imidazole **81** was synthesized with 37% yield (225 mg, 1.06 mmol). Purification was performed by FCC (40 g silica, cyclohexane/EtOAc 20% → 80%).
^{1}H-NMR (399 MHz, DMSO-d_6): δ = 12.80 (s, 1H), 8.16 (d, J = 8.4 Hz, 2H), 7.83 (d, J = 8.0, 2H), 7.23 (s, 2H) ppm.
^{13}C-NMR (100 MHz, DMSO-d_6): δ = 144.6, 134.9, 128.5, 128.1, 126.3, 126.2, 126.2, 125.6, 123.4 ppm.

2-(4-(trifluoromethoxy)phenyl)-1*H*-imidazole (82)

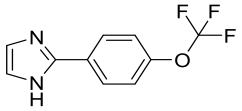

According to GP1, imidazole **82** was synthesized with 40% yield (242 mg, 1.06 mmol). Purification was performed by FCC (40 g silica, cyclohexane/EtOAc 20% → 80%).
^{1}H-NMR (399 MHz, DMSO-d_6): δ = 12.64 (s, 1H), 8.06 (d, J = 8.08 Hz, 2H), 7.46 (d, J = 8.0 Hz, 2H), 7.17 (s, 2H) ppm.
^{13}C-NMR (100 MHz, DMSO-d_6): δ = 166.7, 148.3, 144.8, 132.1, 130.6, 127.0, 124.4, 121.9, 121.1, 119.3 ppm.

2-(4-cyanophenyl)-1*H*-imidazole (83)

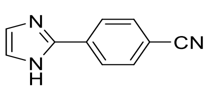

According to GP1, imidazole **83** was synthesized with 40% yield (258 mg, 1.53 mmol). Purification was performed by FCC (40 g silica, cyclohexane/EtOAc 20% → 80%).
^{1}H-NMR (400 MHz, DMSO-d$_6$): δ = 12.86 (s, 1H), 8.12 (d, J = 8.5 Hz, 2H), 7.91 (d, J = 10.8, 2H), 7.38 (s, 1H), 7.14 (s, 1H) ppm.
^{13}C-NMR (100 MHz, DMSO-d$_6$): δ = 144.4, 135.2, 133.3, 130.5, 125.6, 119.7, 119.4, 110.4 ppm.

2-(4-fluoro-3-trifluoromethoxy)-1*H*-imidazole (84)

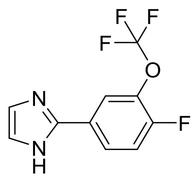

According to GP1, imidazole **84** was synthesized with 38% yield (222 mg, 902 µmol). Purification was performed by FCC (40 g silica, cyclohexane/EtOAc 20% → 80%).
^{1}H-NMR (400 MHz, DMSO-d$_6$): δ = 12.72 (s, 1H), 8.08 (d, J = 7.2 Hz, 1H), 8.03 -7.99 (m, 1H), 7.64-7.59 (m, 1H), 7.20 (s, 2H) ppm.
^{13}C-NMR (100 MHz, DMSO-d$_6$): δ = 154.8, 152.3, 143.8, 136.1, 135.9, 129.1, 125.9, 124.4, 121.8, 120.3, 119.3, 118.7, 118.5 ppm.

General Procedure 2 (GP 2)

A mixture of 2-substituted imidazole (1 eq.), 2-bromo-4-fluoro-benzaldehyde (1.25 eq.), K_2CO_3 (1.25 eq.), and DMSO (1 mL) were stirred 1 h at 130 °C under microwave irradiation. Aqueous NaCl solution (sat., 10 mL) was added and the aqueous layer was extracted with EtOAc (4 x 10 mL). The combined organic extracts were treated with Na_2SO_4 and evaporated under reduced pressure.
Automated flash column chromatography (silica, CH_2Cl_2/MeOH or hexanes/EtOAc) of the residue afforded the corresponding 1,2-disubstituted imidazole.

2-bromo-4-(2'-(3''-chlorophenyl)-1'*H*-imidazol-1'-yl)benzaldehyde (8)

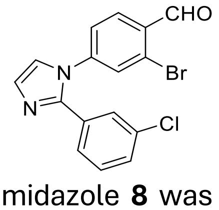

According to GP2, 1,2-disubstituted imidazole **8** was synthesized with 29% yield (53.6 mg, 148 µmol). Purification was performed by FCC (4 g silica, cyclohexane/EtOAc 0% → 100%).
TLC: R_f = 0.24 (EtOAc/hexane, 1:1).
^{1}H-NMR (400 MHz, $CDCl_3$): δ = 10.36 (d, J = 0.8 Hz, 1H), 7.94 (d, J = 8.3 Hz, 1H), 7.58 (d, J = 2.1 Hz, 1H), 7.54 (t, J = 1.9 Hz, 1H), 7.33 (ddd, J = 8.0, 2.1, 1.1 Hz, 1H), 7.30 (d, J = 1.4 Hz, 1H), 7.26 – 7.20 (m, 3H), 7.13 (dt, 1H) ppm.
^{13}C-NMR (101 MHz, $CDCl_3$): δ = 190.3, 145.4, 143.3, 134.7, 132.8, 131.3, 130.9, 130.34, 130.25, 129.7, 129.2, 128.9, 127.5, 126.7, 125.1, 122.5 ppm.
LC-MS (ESI-TOF): t_R = 8.65 min, calculated for $C_{16}H_{11}BrClN_2O$ $[M+H]^+$ 360.97; found 361.25.

HRMS (ESI-TOF) calculated for $C_{16}H_{11}BrClN_2O$ $[M+H]^+$ 360.9743; found 360.9741.

2-bromo-4-(2'-(o-tolyl)-1'*H*-imidazol-1'-yl)benzaldehyde (3)

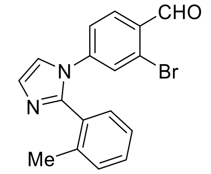

According to GP2, 1,2-disubstituted imidazole **3** was synthesized with 34% yield (61.2 mg, 179 µmol). Purification was performed by FCC (4 g silica, cyclohexane/EtOAc 20% → 80%).
TLC: R_f = 0.21 (hexane/EtOAc, 1:1).
^{1}H-NMR (400 MHz, $CDCl_3$) δ = 10.28 (d, J = 0.8 Hz, 1H), 7.81 (d, J = 8.4 Hz, 1H), 7.42 (d, J = 2.1 Hz, 1H), 7.35 – 7.27 (m, 3H), 7.24 – 7.15 (m, 3H), 7.07 (ddd, J = 8.3, 2.1, 0.9 Hz, 1H), 2.10 (s, 3H) ppm.
^{13}C-NMR: (100 MHz, $CDCl_3$) δ = 190.4, 146.8, 143.2, 137.7, 131.9, 130.72, 130.68, 130.6, 129.9, 129.8, 129.7, 128.9, 127.2, 126.0, 123.6, 120.1, 19.9 ppm.
LC-MS (ESI-TOF): t_R = 7.17 min, calculated for $C_{17}H_{14}BrN_2O$ $[M+H]^+$ 341.03; found 341.25.
HRMS (ESI-TOF) calculated for $C_{17}H_{14}BrN_2O$ $[M+H]^+$ 341.0290; found 341.0285.

2-bromo-4-(2'-(*m*-tolyl)-1'*H*-imidazol-1'-yl)benzaldehyde (6)

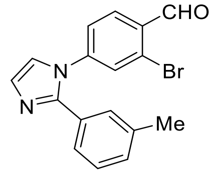

According to GP2, 1,2-disubstituted imidazole **6** was synthesized with 33% yield (62.6 mg, 183 µmol). Purification was performed by FCC (4 g silica, cyclohexane/EtOAc 0% → 100%).
TLC: R_f = 0.25 (hexane/EtOAc, 1:1).
^{1}H-NMR (400 MHz, $CDCl_3$): δ = 10.35 (d, J = 0.9 Hz, 1H), 7.90 (d, J = 8.3 Hz, 1H), 7.57 (d, J = 2.0 Hz, 1H), 7.38 (q, J = 1.1 Hz, 1H), 7.28 (d, J = 1.4 Hz, 2H), 7.23 (ddd, J = 8.3, 2.1, 0.8 Hz, 2H), 7.18 (dd, J = 12.6, 1.3 Hz, 4H), 7.18 – 7.13 (m, 2H), 7.04 – 6.97 (m, 1H), 2.32 (s, 3H) ppm.
^{13}C-NMR (101 MHz, $CDCl_3$): δ = 190.4, 147.1, 143.8, 138.6, 132.4, 130.7, 130.14, 130.05, 129.9, 129.6, 129.5, 128.3, 127.3, 125.8, 125.1, 121.9, 21.4 ppm.
LC-MS (ESI-TOF): t_R = 7.24 min, calculated for $C_{17}H_{14}BrN_2O$ $[M+H]^+$ 341.03; found 341.25.
HRMS (ESI-TOF) calculated for $C_{17}H_{14}BrN_2O$ $[M+H]^+$ 341.0290; found 341.0290.

2-bromo-4-(2'-(3''-methoxyphenyl)-1'*H*-imidazol-1'-yl)benzaldehyde (7)

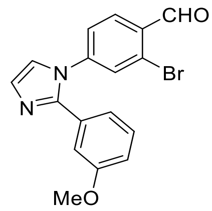

According to GP2, 1,2-disubstituted imidazole **7** was synthesized with 51% yield (87.7 mg, 246 µmol). Purification was performed by FCC (4 g silica, cyclohexane/EtOAc 20% → 80%).
TLC: R_f = 0.16 (hexane/EtOAc, 1:1).
^{1}H-NMR (399 MHz, $CDCl_3$): δ = 10.35 (s, 1H), 7.91 (d, J = 8.3 Hz, 1H), 7.58 (d, J = 2.0 Hz, 1H), 7.29 – 7.14 (m, 4H), 7.07 (t, J = 2.1 Hz, 1H), 6.90 (dd, J = 8.3, 2.6 Hz, 1H), 6.81 (d, J = 7.6 Hz, 1H), 3.77 (s, 3H) ppm.
^{13}C-NMR (100 MHz, $CDCl_3$): δ = 190.4, 159.7, 146.8, 143.7, 132.5, 130.8, 130.7, 130.2, 130.1, 129.5, 127.4, 125.1, 122.1, 121.2, 115.4, 113.9, 55.3 ppm.
LC-MS (ESI-TOF): t_R = 7.25 min, calculated for $C_{17}H_{14}BrN_2O_2$ $[M+H]^+$ 357.02; found 357.25.

2-bromo-4-(2-(naphthalen-1-yl)-1H-imidazol-1-yl)benzaldehyde (32)

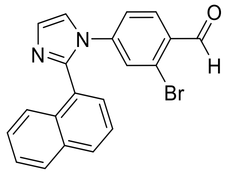

According to GP2, 1-2-disubstituted imidazole **32** was synthesized with 75% yield (78.5 mg, 208 µmol). Purification was performed by FCC (24 g silica, heptane/EtOAc 8% → 60%).
^{1}H-NMR (400 MHz, $CDCl_3$): δ = 10.20 (s, 1H), 7.93 – 7.85 (m, 3H), 7.66 (d, J = 8.4 Hz, 1H), 7.50 – 7.42 (m, 4H), 7.41 – 7.36 (m, 2H), 7.32 (dd, J = 7.1, 1.4 Hz, 1H), 6.99 (dd, J = 8.4, 2.2 Hz, 1H) ppm.
^{13}C{^{1}H}-NMR (100 MHz, $CDCl_3$): δ = 190.4, 145.9, 143.3, 133.8, 132.1, 132.1, 130.6, 130.3, 130.2, 129.3, 129.3, 128.5, 127.4, 127.3, 127.2, 126.5, 125.5, 125.0, 124.0, 120.9 ppm.
LC-MS (ESI-TOF): t_R = 8.12 min, calculated for $C_{20}H_{14}BrN_2O$ $[M+H]^+$ 377.03; found 377.33.

2-bromo-4-(2-(quinolin-8-yl)-1H-imidazol-1-yl)benzaldehyde (33)

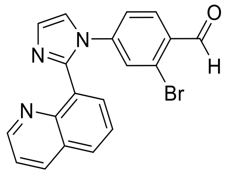

According to GP2, 1-2-disubstituted imidazole **33** was synthesized with 31% yield (27.0 mg, 71.4 µmol). Purification was performed by FCC (4 g silica, heptane/EtOAc 8% → 60%).
^{1}H-NMR (400 MHz, $CDCl_3$): δ = 10.16 (s, 1H), 8.56 (dd, J = 4.2, 1.8 Hz, 1H), 8.10 (d, J = 8.3 Hz, 1H), 8.05 (d, J = 7.2 Hz, 1H), 7.91 (d, J = 8.2 Hz, 1H), 7.64 (t, J = 7.7 Hz, 1H), 7.59 (d, J = 8.4 Hz, 1H), 7.48 (d, J = 1.9 Hz, 1H), 7.41 (d, J = 4.9 Hz, 2H), 7.27 – 7.23 (m, 1H), 7.05 (dd, J = 8.3, 2.1 Hz, 1H) ppm.
^{13}C{^{1}H}-NMR (100 MHz, $CDCl_3$): δ = 190.6, 150.4, 145.8, 145.7, 144.7, 136.1, 132.5, 131.5, 130.3, 130.2, 130.1, 129.9, 129.2, 128.4, 126.8, 126.5, 123.6, 121.4, 121.2 ppm.
LC-MS (ESI-TOF): t_R = 6.77 min, calculated for $C_{19}H_{13}BrN_3O$ $[M+H]^+$ 378.02; found 378.25.

2-bromo-4-(2-(isoquinolin-5-yl)-1*H*-imidazol-1-yl)benzaldehyde (34)

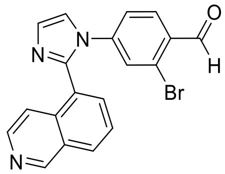

According to GP2, 1-2-disubstituted imidazole **34** was synthesized with 65% yield (32.2 mg, 85.1 µmol). Purification was performed by FCC (4 g silica, heptane/EtOAc 8% → 80%).

^{1}H-NMR (400 MHz, $CDCl_3$): δ = 10.21 (s, 1H), 9.28 (s, 1H), 8.51 (d, *J* = 6.0 Hz, 1H), 8.02 (d, *J* = 7.7 Hz, 1H), 7.85 (d, *J* = 6.0 Hz, 1H), 7.71 (d, *J* = 8.3 Hz, 1H), 7.55 – 7.46 (m, 2H), 7.46 – 7.41 (m, 2H), 7.38 (d, *J* = 1.5 Hz, 1H), 7.00 (dd, *J* = 8.3, 2.5 Hz, 1H) ppm.

^{13}C{^{1}H}-NMR (100 MHz, $CDCl_3$): δ = 190.3, 153.0, 144.3, 143.0, 134.7, 133.2, 132.4, 130.8, 130.6, 129.7, 129.5, 128.9, 127.4 126.4, 124.2, 121.6, 118.4 ppm.

LC-MS (ESI-TOF): t_R = 6.73 min, calculated for $C_{19}H_{13}BrN_3O$ [M+H]$^+$ 378.02; found 378.25.

2-bromo-4-(2-(isoquinolin-8-yl)-1H-imidazol-1-yl)benzaldehyde (35)

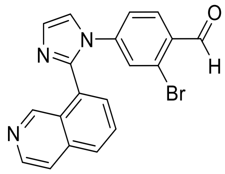

According to GP2, 1-2-disubstituted imidazole **35** was synthesized with 49% yield (29.4 mg, 77.7 µmol). Purification was performed by FCC (24 g silica, heptane/EtOAc 8% → 80%).

^{1}H-NMR (400 MHz, $CDCl_3$): δ =10.23 (s, 1H), 9.38 (s, 1H), 8.56 (d, *J* = 5.7 Hz, 1H), 7.88 (d, *J* = 8.3 Hz, 1H), 7.73 (d, *J* = 8.4 Hz, 1H), 7.68 (d, J = 5.8 Hz, 1H), 7.62 (t, *J* = 7.7 Hz, 1H), 7.49 – 7.44 (m, 2H), 7.44 – 7.40 (m, 2H), 7.08 – 7.01 (m, 1H) ppm.

^{13}C{^{1}H}-NMR (100 MHz, $CDCl_3$): δ = 190.4, 150.8, 144.2, 143.7, 143.0, 136.4, 132.5, 130.83, 130.79, 130.4, 129.6, 129.5 128.6, 128.2, 127.5, 127.1, 124.3, 121.7, 120.7 ppm.

LC-MS (ESI-TOF): t_R = 6.80 min, calculated for $C_{19}H_{13}BrN_3O$ [M+H]$^+$ 378.02; found 378.25.

4-(2-(benzo[b]thiophen-4-yl)-1H-imidazol-1-yl)-2-bromobenzaldehyde (37)

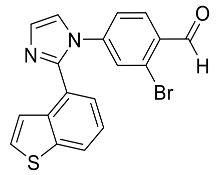

According to GP2, 1-2-disubstituted imidazole **37** was synthesized with 32% yield (18.6 mg, 48.5 µmol). Purification was performed by FCC (4 g silica, heptane/EtOAc 8% → 80%).

^{1}H-NMR (400 MHz, $CDCl_3$): δ = 10.28 (d, *J* = 0.9 Hz, 1H), 7.90 (dt, *J* = 8.1, 1.0 Hz, 1H), 7.79 (d, *J* = 8.4 Hz, 1H), 7.58 (dd, *J* = 5.6, 0.9 Hz, 1H), 7.50 (d, *J* = 2.1 Hz, 1H), 7.48 (d, *J* = 5.5 Hz, 1H), 7.39 (d, *J* = 1.5 Hz, 1H), 7.31 (d, *J* = 1.5 Hz, 1H), 7.23 (t, *J* = 7.8 Hz, 1H), 7.10 (ddd, *J* = 8.4, 2.1, 0.9 Hz, 1H), 7.07 (dd, *J* = 7.3, 1.0 Hz, 1H) ppm.

^{13}C{^{1}H}-NMR (101 MHz, $CDCl_3$): δ = 190.5, 146.0, 143.5, 141.1, 138.6, 132.3, 130.8, 130.4, 129.6, 127.8, 127.4, 126.3, 124.8, 124.5, 123.8, 123.8, 123.6, 121.4 ppm.

LC-MS (ESI-TOF): t_R = 8.18 min, calculated for $C_{18}H_{12}BrN_2OS$ $[M+H]^+$ 382.98; found 383.25.

4-(2-(benzofuran-7-yl)-1H-imidazol-1-yl)-2-bromobenzaldehyde (36)

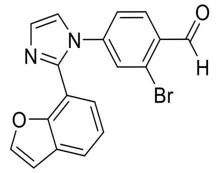

According to GP2, 1-2-disubstituted imidazole **36** was synthesized with 53% yield (52.8 mg, 144 µmol). Purification was performed by FCC (4 g silica, heptane/EtOAc 8% → 80%).
^{1}H-NMR (400 MHz, $CDCl_3$): δ = 10.25 (s, 1H), 7.75 (d, *J* = 8.4 Hz, 1H), 7.61 (dd, *J* = 7.8, 1.3 Hz, 1H), 7.52 (d, *J* = 2.1 Hz, 1H), 7.40 (d, *J* = 2.2 Hz, 1H), 7.38 (d, *J* = 1.5 Hz, 1H), 7.34 (dd, *J* = 7.6, 1.3 Hz, 1H), 7.31 (d, *J* = 1.6 Hz, 1H), 7.23 (t, *J* = 7.6 Hz, 1H), 7.14 (dd, *J* = 8.4, 2.6 Hz, 1H), 6.70 (d, *J* = 2.3 Hz, 1H) ppm.
^{13}C{^{1}H}-NMR (101 MHz, $CDCl_3$): δ = 190.5, 151.7, 145.444 143.8, 143.2, 132.2, 130.7, 130.5, 129.4, 128.4, 127.1, 125.7, 124.0, 123.2, 122.8, 121.7, 114.5, 106.8 ppm.
LC-MS (ESI-TOF): t_R = 7.89 min, calculated for $C_{18}H_{12}BrN_2O_2$ $[M+H]^+$ 367.01; found 367.25.

2-bromo-4-(2-(5-bromofuran-2-yl)-1*H*-imidazol-1-yl)benzaldehyde (40)

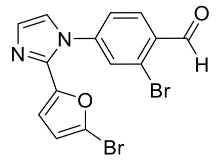

According to GP2, 1-2-disubstituted imidazole **40** was synthesized with 62% yield (57.3 mg, 145 µmol). Purification was performed by FCC (12 g silica, heptane/EtOAc 12% → 80%).
^{1}H-NMR (400 MHz, $CDCl_3$): δ = 10.37 (d, *J* = 0.9 Hz, 1H), 8.00 (d, *J* = 8.2 Hz, 1H), 7.66 (d, *J* = 2.1 Hz, 1H), 7.39 (ddd, *J* = 8.3, 2.1, 0.9 Hz, 1H), 7.24 (d, *J* = 1.5 Hz, 1H), 7.11 (d, *J* = 1.3 Hz, 1H), 6.47 (d, *J* = 3.5 Hz, 1H), 6.32 (d, *J* = 3.4 Hz, 1H) ppm.
^{13}C{^{1}H}-NMR (101 MHz, $CDCl_3$): δ = 190.5, 146.1, 143.0, 137.8, 133.3, 131.2, 130.7, 130.5, 127.2, 125.6, 123.3, 122.1, 113.4, 113.0 ppm.
LC-MS (ESI-TOF): t_R = 8.18 min, calculated for $C_{14}H_9Br_2N_2O$ $[M+H]^+$ 394.90; found 395.25.

2-bromo-4-(2-(2-bromothiophen-3-yl)-1H-imidazol-1-yl)benzaldehyde (39)

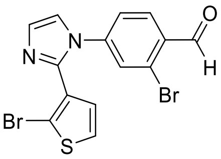

According to GP2, 1-2-disubstituted imidazole **39** was synthesized with 30% yield (18.6 mg, 45.1 µmol). Purification was performed by FCC (4 g silica, heptane/EtOAc 8% → 80%).
^{1}H-NMR (400 MHz, $CDCl_3$): δ = 10.30 (s, 1H), 7.88 (d, *J* = 8.3 Hz, 1H), 7.52 (d, *J* = 2.3 Hz, 1H), 7.41 (d, *J* = 5.4 Hz, 1H), 7.35 – 7.30 (m, 2H), 7.23 – 7.19 (m, 1H), 6.95 (d, *J* = 5.2 Hz, 1H) ppm.

$^{13}C\{^1H\}$**-NMR** (101 MHz, $CDCl_3$): δ = 190.5, 143.0, 138.9, 132.5, 131.0, 130.81, 130.79, 129.6, 129.0, 127.3, 126.8, 124.0, 121.9, 113.1 ppm.
LC-MS (ESI-TOF): t_R = 8.18 min, calculated for $C_{14}H_9Br_2N_2OS$ $[M+H]^+$ 410.88; found 411.25.

tert-butyl 4-(1-(3-bromo-4-formylphenyl)-1H-imidazol-2-yl)piperidine-1-carboxylate (85)

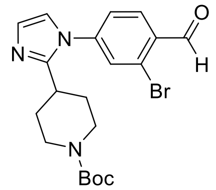

According to GP2, 1-2-disubstituted imidazole **85** was synthesized with 50% yield (250 mg, 576 µmol). Purification was performed by FCC (4 g silica, CH_2Cl_2/MeOH 2% → 10%).
^1H**-NMR** (399 MHz, $CDCl_3$): δ = 10.40 (s, 1H), 8.07 (d, *J* = 8.3 Hz, 1H), 7.63 (d, *J* = 2.4 Hz, 1H), 7.39 (dd, *J* = 8.2, 2.4 Hz, 1H), 7.11 (d, *J* = 1.3 Hz, 1H), 6.99 (d, *J* = 1.3 Hz, 1H), 4.15 (s, 2H), 2.78 (tt, *J* = 10.6, 3.7 Hz, 1H), 2.71 (d, *J* = 12.5 Hz, 2H), 1.87 (d, *J* = 12.1 Hz, 2H), 1.73 (d, *J* = 11.0 Hz, 2H), 1.44 (s, 9H) ppm.
$^{13}C\{^1H\}$**-NMR** (100 MHz, $CDCl_3$): δ = 190.5, 154.7, 150.8, 143.2, 133.3, 131.2, 131.0, 129.0, 127.7, 125.4, 120.5, 79.7, 34.2, 31.202 28.6 ppm.
LC-MS (ESI-TOF): t_R = 7.30 min, calculated for $C_{20}H_{25}BrN_3O_3$ $[M+H]^+$ 434.11; found 434.00.

tert-butyl (*R*)-2-(1-(3-bromo-4-formylphenyl)-1*H*-imidazol-2-yl)pyrrolidine-1-carboxylate (41)

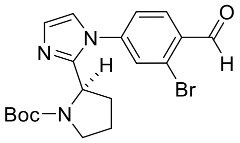

According to GP2, 1-2-disubstituted imidazole **41** was synthesized with 59% yield (182 mg, 433 µmol). Purification was performed by FCC (4 g silica, CH_2Cl_2/MeOH 2% → 10%).
^1H**-NMR** (400 MHz, $CDCl_3$): δ = 10.39 (s, 1H), 8.04 (d, *J* = 7.7 Hz, 1H), 7.69 (d, *J* = 12.4 Hz, 1H), 7.13 (d, *J* = 14.4 Hz, 1H), 6.99 (d, *J* = 15.7 Hz, 1H), 4.89 (d, J = 5.1 Hz, 0.5H), 4.78 (sb, 0.5H), 3.75 – 3.63 (m, 1H), 3.54 – 3.37 (m, 1H), 2.24 (d, *J* = 23.6 Hz, 1.5H), 2.14 – 1.97 (m, 1.3H), 1.93 – 1.83 (m, 1.5H), 1.42 (s, 5H), 1.24 (s, 4H) ppm.
LC-MS (ESI-TOF): t_R = 8.18 min, calculated for $C_{19}H_{23}BrN_3O_3$ $[M+H]^+$ 420.09; found 420.00.

2-bromo-4-(2-(3-hydroxyphenyl)-1H-imidazol-1-yl)benzaldehyde (9)

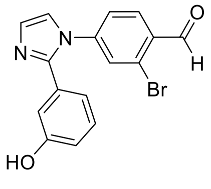

According to GP2, 1-2-disubstituted imidazole **9** was synthesized with 17% yield (8.9 mg, 25.9 μmol). Purification was performed by FCC (4 g silica, CH_2Cl_2/MeOH 2% → 10%).

^{1}H-NMR (400 MHz, $CDCl_3$:methanol-d_4 = 9:1): δ = 10.24 (d, J = 4.7 Hz, 1H), 7.90 (dd, J = 8.7, 5.2 Hz, 1H), 7.80 – 7.75 (m, 1H), 7.63 (q, J = 2.4 Hz, 1H), 7.49 (t, J = 8.0 Hz, 1H), 7.36 (d, J = 5.1 Hz, 1H), 7.22 (dd, J = 5.1, 2.5 Hz, 1H), 7.12 (d, J = 1.6 Hz, 2H), 7.04 (ddd, J = 15.0, 8.4, 2.6 Hz, 3H) ppm.

^{13}C{^{1}H}-NMR (100 MHz, $CDCl_3$:methanol-d_4 = 9:1): δ = 191.0, 163.2, 155.0, 145.5, 132.6, 131.5, 130.8, 128.6, 128.3, 122.2, 121.8, 120.3, 117.3, 116.8 ppm.

LC-MS (ESI-TOF): t_R = 6.79 min, calculated for $C_{16}H_{12}BrN_2O_2$ $[M+H]^+$ 343.01; found 343.25.

2-bromo-4-(2-(4-morpholinophenyl)-1H-imidazol-1-yl)benzaldehyde (23)

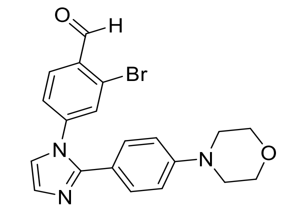

According to GP2, 1-2-disubstituted imidazole **23** was synthesized with 38% yield (39.5 mg, 958 μmol). Purification was performed by FCC (4 g silica, CH_2Cl_2/MeOH 2% → 10%).

^{1}H-NMR (400 MHz, $CDCl_3$): δ = 10.35 (d, J = 0.9 Hz, 1H), 7.90 (d, J = 8.4 Hz, 1H), 7.60 (d, J = 2.0 Hz, 1H), 7.29 (s, 1H), 7.27 (d, J = 2.0 Hz, 1H), 7.24 (dd, J = 9.1, 1.8 Hz, 2H), 7.15 (d, J = 1.5 Hz, 1H), 6.84 – 6.79 (m, 2H), 3.87 – 3.82 (m, 4H), 3.22 – 3.17 (m, 4H) ppm.

^{13}C{^{1}H}-NMR (100 MHz, $CDCl_3$): δ = 190.6, 151.5, 147.2, 144.2, 132.5, 130.8, 130.3, 130.0, 129.9, 127.5, 125.3, 121.6, 120.6, 114.8, 66.8, 48.5 ppm.

LC-MS (ESI-TOF): t_R = 6.85 min, calculated for $C_{20}H_{19}BrN_3O_2$ $[M+H]^+$ 412.07; found 412.25.

2-bromo-4-(2-(3-(methylsulfonyl)phenyl)-1*H*-imidazol-1-yl)benzaldehyde (86)

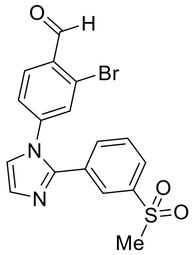

According to GP2, 1-2-disubstituted imidazole **86** was synthesized with 38% yield (34.1 mg, 84.1 μmol). Purification was performed by FCC (4 g silica, CH_2Cl_2/MeOH 2% → 17%).

^{1}H-NMR (400 MHz, $CDCl_3$): δ = 10.35 (s, 1H), 8.08 (t, J = 1.9 Hz, 1H), 7.96 (d, J = 8.2 Hz, 1H), 7.93 (dt, J = 7.8, 1.7 Hz, 1H), 7.61 – 7.57 (m, 2H), 7.52 (t, J = 7.8 Hz, 1H), 7.34 (d, J = 1.6 Hz, 1H), 7.30 – 7.27 (m, 2H), 3.00 (s, 3H) ppm.

^{13}C{^{1}H}-NMR (100 MHz, $CDCl_3$): δ = 190.2, 144.8, 143.0, 141.4, 133.5, 133.2, 131.2, 131.2, 130.7, 130.5, 129.7, 127.8, 127.7, 127.6, 125.2, 123.1, 44.4 ppm.

LC-MS (ESI-TOF): t_R = 7.84 min, calculated for $C_{17}H_{14}BrN_2O_3S$ $[M+H]^+$ 404.99; found 405.25.

4-(2-(benzofuran-4-yl)-1H-imidazol-1-yl)-2-bromobenzaldehyde (38)

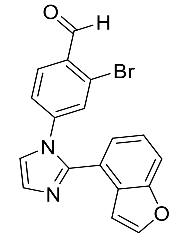

According to GP2, 1-2-disubstituted imidazole **38** was synthesized with 32% yield (25.5 mg, 69.4 µmol). Purification was performed by FCC (4 g silica, CH_2Cl_2/MeOH 2% → 10%).
^{1}H-NMR (400 MHz, $CDCl_3$): δ = 10.30 (s, 1H), 7.84 (dd, J = 8.4, 0.9 Hz, 1H), 7.63 (d, J = 3.2 Hz, 1H), 7.56 – 7.54 (m, 1H), 7.51 (d, J = 8.2 Hz, 1H), 7.38 (d, J = 1.3 Hz, 1H), 7.28 (d, J = 2.8 Hz, 1H), 7.20 – 7.15 (m, 2H), 6.97 (d, J = 7.5 Hz, 1H), 6.93 (d, J = 2.6 Hz, 1H) ppm.
^{13}C{^{1}H}-NMR (100 MHz, $CDCl_3$): δ = 190.5, 155.3, 146.1, 145.6, 143.6, 132.5, 130.9, 130.4, 129.9, 127.5, 127.1, 124.7, 124.1, 124.0, 124.0, 122.5, 121.8, 112.6, 106.8 ppm.
LC-MS (ESI-TOF): t_R = 7.91 min, calculated for $C_{18}H_{12}BrN_2O_2$ $[M+H]^+$ 367.01; found 367.25.

2-bromo-4-(2-(4-iodophenyl)-1*H*-imidazol-1-yl)benzaldehyde (15)

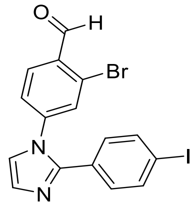

According to GP2, 1-2-disubstituted imidazole **15** was synthesized with 56% yield (29.8 mg, 65.8 µmol). Purification was performed by FCC (4 g silica, CH_2Cl_2/MeOH 1% → 5%).
^{1}H-NMR (400 MHz, $CDCl_3$): δ = 10.36 (s, 1H), 7.93 (d, J = 8.3 Hz, 1H), 7.66 (d, J = 8.3 Hz, 2H), 7.59 (d, J = 2.7 Hz, 1H), 7.30 (d, J = 1.2 Hz, 1H), 7.24 – 7.20 (m, 2H), 7.14 – 7.10 (m, 2H) ppm.
^{13}C{^{1}H}-NMR (100 MHz, $CDCl_3$): δ = 190.4, 146.1, 143.5, 137.9, 133.0, 131.0, 130.4, 130.4, 129.1, 127.6, 125.3, 122.6, 95.6 ppm.
LC-MS (ESI-TOF): t_R = 8.94 min, calculated for $C_{16}H_{11}BrIN_2O$ $[M+H]^+$ 452.91; found 453.25.
HRMS (ESI-TOF) calculated for $C_{16}H_{11}BrIN_2O$ $[M+H]^+$ 452.9099; found 452.9096.

2-bromo-4-(2-(4-chlorophenyl)-1*H*-imidazol-1-yl)benzaldehyde (14)

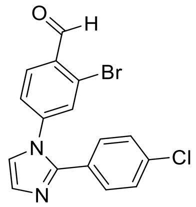

According to GP2, 1-2-disubstituted imidazole **14** was synthesized with 53% yield (53.3 mg, 147 µmol). Purification was performed by FCC (4 g silica, CH_2Cl_2/MeOH 1% → 5%).
^{1}H-NMR (400 MHz, $CDCl_3$): δ = 10.35 (s, 1H), 7.93 (d, J = 8.3 Hz, 1H), 7.58 (m, 1H), 7.30 (m, 5H), 7.24 – 7.20 (m, 2H) ppm.

$^{13}C\{^{1}H\}$-NMR (100 MHz, $CDCl_3$): δ = 190.4, 145.9, 143.5, 135.3, 132.8, 131.0, 130.4, 130.3, 130.1, 129.0, 128.1, 127.6, 125.2, 122.5 ppm.
LC-MS (ESI-TOF): t_R = 8.53 min, calculated for $C_{16}H_{11}BrClN_2O$ $[M+H]^+$ 360.97; found 361.25.
HRMS (ESI-TOF) calculated for $C_{16}H_{11}BrClN_2O$ $[M+H]^+$ 360.9743; found 360.9740.

2-bromo-4-(2-(4-fluorophenyl)-1*H*-imidazol-1-yl)benzaldehyde (13)

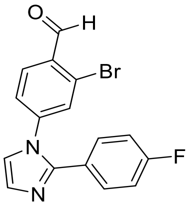

According to GP2, 1-2-disubstituted imidazole **13** was synthesized with 61% yield (65.3 mg, 189 µmol). Purification was performed by FCC (4 g silica, CH_2Cl_2/MeOH 1% → 5%).
^{1}H-NMR (400 MHz, $CDCl_3$): δ = 10.34 (s, 1H), 7.92 (d, *J* = 8.4 Hz, 1H), 7.56 (d, *J* = 2.7 Hz, 1H), 7.40 – 7.35 (m, 2H), 7.28 (d, *J* = 1.3 Hz, 1H), 7.23 (dd, *J* = 8.3, 2.3 Hz, 1H), 7.21 (d, *J* = 1.2 Hz, 1H), 7.02 (t, *J* = 8.6 Hz, 2H) ppm.
$^{13}C\{^{1}H\}$-NMR (100 MHz, $CDCl_3$): δ = 190.4, 164.4, 161.9, 146.4, 143.6, 132.7, 130.91, 130.87, 130.8, 130.4, 130.2, 127.5, 125.9, 125.9, 125.1, 122.2, 116.0, 115.7 ppm.
LC-MS (ESI-TOF): t_R = 7.68 min, calculated for $C_{16}H_{11}BrFN_2O$ $[M+H]^+$ 345.00; found 345.25.
HRMS (ESI-TOF) calculated for $C_{16}H_{11}BrFN_2O$ $[M+H]^+$ 345.0039; found 345.0037.

2-bromo-4-(2-(4-hydroxyphenyl)-1*H*-imidazol-1-yl)benzaldehyde (16)

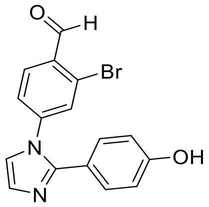

According to GP2, 1-2-disubstituted imidazole **16** was synthesized with 20% yield (31.6 mg, 92.1 µmol). Purification was performed by FCC (4 g silica, CH_2Cl_2/MeOH 1% → 5%).
^{1}H-NMR (400 MHz, $CDCl_3$): δ = 10.23 (s, 1H), 7.94 – 7.90 (m, 2H), 7.88 (dd, *J* = 8.7, 1.2 Hz, 1H), 7.17 (d, *J* = 3.2 Hz, 3H), 7.06 (dd, *J* = 8.6, 1.4 Hz, 2H), 6.96 (dd, *J* = 8.6, 2.6 Hz, 1H) ppm.
$^{13}C\{^{1}H\}$-NMR (100 MHz, $CDCl_3$): δ = 190.7, 162.9, 155.1, 146.0, 131.7, 128.8, 128.7, 127.8, 127.4, 122.1, 121.0, 117.1 ppm.
LC-MS (ESI-TOF): t_R = 6.91 min, calculated for $C_{16}H_{12}BrN_2O_2$ $[M+H]^+$ 343.01; found 343.25.

2-bromo-4-(2-(4-ethylyphenyl)-1*H*-imidazol-1-yl)benzaldehyde (18)

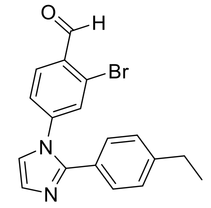

According to GP1, 1-2-disubstituted imidazole **18** was synthesized with 39% yield (79.8 mg, 225 µmol). Purification was performed by FCC (4 g silica, heptane/EtOAc 12% → 60%).
^{1}H-NMR (400 MHz, $CDCl_3$): δ = 10.33 (s, 1H), 7.89 (d, *J* = 8.3 Hz, 1H), 7.57 (d, *J* = 2.2 Hz, 1H), 7.31 – 7.25 (m, 3H), 7.22 (dd, *J* = 8.3, 2.3 Hz, 1H), 7.19 (d, *J* = 1.5 Hz, 1H), 7.14 (d, *J* = 8.3 Hz, 2H), 2.64 (q, *J* = 7.7 Hz, 2H), 1.22 (t, *J* = 7.6 Hz, 3H) ppm.
^{13}C{^{1}H}-NMR (100 MHz, $CDCl_3$): δ = 190.5, 147.1, 145.4, 143.9, 132.5, 130.7, 130.2, 130.0, 128.880, 128.1, 127.4, 127.0, 125.2, 121.9, 28.7, 15.3 ppm.
LC-MS (ESI-TOF): t_R = 7.51 min, calculated for $C_{18}H_{16}BrN_2O$ $[M+H]^+$ 355.05; found 355.25.
HRMS (ESI-TOF) calculated for $C_{18}H_{16}BrN_2O$ $[M+H]^+$ 355.0446; found 355.0438.

2-bromo-4-(2'-(*p*-tolyl)-1'*H*-imidazol-1'-yl)benzaldehyde (11)

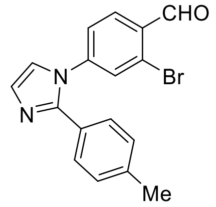

According to GP2, 1-2-disubstituted imidazole **11** was synthesized with 33% yield (65.0 mg, 191 µmol). Purification was performed by FCC (4 g silica, cyclohexane/EtOAc 0% → 100%).
TLC: R_f = 0.29 (hexane/EtOAc, 1:1).
^{1}H-NMR (400 MHz, $CDCl_3$): δ = 10.35 (d, *J* = 0.9 Hz, 1H), 7.90 (d, *J* = 8.3 Hz, 1H), 7.58 (d, *J* = 2.0 Hz, 1H), 7.29 – 7.25 (m, 3H), 7.21 (ddd, *J* = 8.3, 2.1, 0.8 Hz, 1H), 7.18 (d, *J* = 1.4 Hz, 1H), 7.14 – 7.10 (m, 2H), 2.35 (s, 3H) ppm.
^{13}C-NMR (100 MHz, $CDCl_3$): δ = 190.4, 147.1, 143.9, 139.2, 132.4, 130.7, 130.2, 130.0, 129.3, 128.7, 127.4, 126.7, 125.2, 121.8, 21.3 ppm.
LC-MS (ESI-TOF): calculated for $C_{17}H_{14}BrN_2O$ $[M+H]^+$ 341.03; found 341.25.
HRMS (ESI-TOF) calculated for $C_{17}H_{14}BrN_2O$ $[M+H]^+$ 341.0290; found 341.0279.

2-bromo-4-(2'-(2''-methoxyphenyl)-1'*H*-imidazol-1'-yl)benzaldehyde (4)

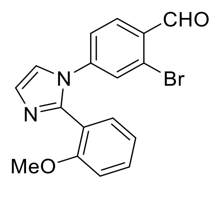

According to GP2, 1,2-disubstituted imidazole **4** was synthesized with 47% yield (87.4 mg, 245 µmol). Purification was performed by FCC (12 g silica, cyclohexane/EtOAc 0% → 80%).
TLC: R_f = 0.23 (hexane/EtOAc, 1:1).
^{1}H-NMR (400 MHz, $CDCl_3$): δ = 10.30 (d, *J* = 0.8 Hz, 1H), 7.84 (d, *J* = 8.4 Hz, 1H), 7.60 (dd, *J* = 7.5, 1.8 Hz, 1H), 7.48 (d, *J* = 2.0 Hz, 1H), 7.39 (ddd, *J* = 8.3, 7.5, 1.8 Hz, 1H), 7.30 (d, *J* = 1.4 Hz, 1H), 7.26 (d, *J* = 1.4 Hz, 1H), 7.15 (ddd, *J* = 8.4, 2.1, 0.9 Hz, 1H), 7.07 (td, *J* = 7.5, 1.0 Hz, 1H), 6.76 (dd, *J* = 8.3, 1.0 Hz, 1H), 3.32 (s, 3H) ppm.
^{13}C-NMR (101 MHz, $CDCl_3$): δ = 190.5, 156.3, 144.9, 144.5, 131.8, 131.7, 131.4, 130.3, 130.1, 128.5, 126.9, 123.0, 121.2, 120.5, 119.4, 111.0, 54.7 ppm.
LC-MS (ESI-TOF): calculated for $C_{17}H_{14}BrN_2O_2$ $[M+H]^+$ 357.02; found 357.25.

2-bromo-4-(2'-(2'' -chlorophenyl)-1'*H*-imidazol-1'-yl)benzaldehyde (5)

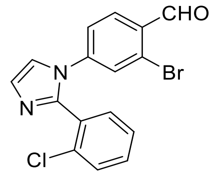

According to GP2, 1,2-disubstituted imidazole **5** was synthesized with 29% yield (58.5 mg, 162 µmol). Purification was performed by FCC (4 g silica, cyclohexane/EtOAc 15% → 90%).
TLC: R_f = 0.23 (hexane/EtOAc, 1:1).
^{1}H-NMR (400 MHz, $CDCl_3$): δ = 10.29 (d, *J* = 0.8 Hz, 1H), 7.58 – 7.51 (m, 1H), 7.45 (d, *J* = 2.1 Hz, 1H), 7.40 – 7.25 (m, 6H), 7.12 (ddd, *J* = 8.4, 2.1, 0.8 Hz, 1H) ppm.
^{13}C-NMR (101 MHz, $CDCl_3$): δ = 190.4, 144.5, 143.1, 134.0, 132.5, 132.1, 131.2, 130.6, 130.2, 130.0, 129.8, 129.0, 127.22, 127.19, 123.1, 120.7 ppm.
LC-MS (ESI-TOF): t_R = 8.50 min, calculated for $C_{16}H_{11}BrClN_2O$ $[M+H]^+$ 360.97; found 361.25.
HRMS (ESI-TOF) calculated for $C_{16}H_{11}BrClN_2O$ $[M+H]^+$ 360.9743; found 360.9725.

2-bromo-4-(2'-(4''-methoxyphenyl)-1'*H*-imidazol-1'-yl)benzaldehyde (12)

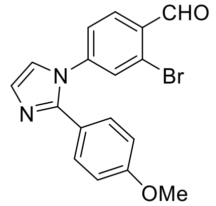

According to GP2, 1,2-disubstituted imidazole **12** was synthesized with 36% yield (74.6 mg, 209 µmol). Purification was performed by FCC (4 g silica, cyclohexane/EtOAc 15% → 90%).
TLC: R_f = 0.64 ($MeOH/CH_2Cl_2$, 1:19).
^{1}H-NMR (400 MHz, $CDCl_3$): δ = 10.35 (d, *J* = 0.8 Hz, 1H), 7.91 (d, *J* = 8.3 Hz, 1H), 7.58 (d, *J* = 2.0 Hz, 1H), 7.34 – 7.29 (m, 2H), 7.26 (d, *J* = 1.4 Hz, 1H), 7.23 (ddd, *J* = 8.3, 2.1, 0.8 Hz, 1H), 7.17 (d, *J* = 1.5 Hz, 1H), 6.86 – 6.82 (m, 2H), 3.81 (s, 3H) ppm.
^{13}C-NMR (101 MHz, $CDCl_3$): δ = 190.5, 160.2, 146.9, 143.9, 132.4, 130.7, 130.23, 130.18, 129.9, 127.4, 125.1, 122.0, 121.6, 114.0, 55.3 ppm.
LC-MS (ESI-TOF): t_R = 6.83 min, calculated for $C_{17}H_{14}BrN_2O_2$ $[M+H]^+$ 357.02; 357.33.

3-(1'-(3''-bromo-4''-formylphenyl)-1'*H*-imidazol-2'-yl)benzamide (10)

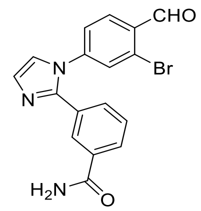

According to GP2, 1,2-disubstituted imidazole **10** was synthesized with 11% yield (10.6 mg, 28.6 µmol). Purification was performed by FCC (4 g silica, CH_2Cl_2/MeOH 0% → 11%).
TLC: R_f = 0.44 (MeOH/CH_2Cl_2, 1:19).
^{1}H-NMR: (399 MHz, DMSO-d$_6$) δ = 10.20 (s, 1H), 8.04 – 7.98 (m, 2H), 7.89 – 7.83 (m, 3H), 7.71 (d, J = 1.4 Hz, 1H), 7.44 – 7.36 (m, 4H), 7.27 (d, J = 1.4 Hz, 1H).
^{13}C-NMR (100 MHz, DMSO-d$_6$) δ = 191.2, 167.6, 145.8, 143.4, 135.0, 132.7, 131.6, 131.4, 130.9, 130.5, 129.9, 128.8, 128.5, 128.1, 126.5, 126.0, 124.0 ppm.
LC-MS (ESI-TOF): t_R = 6.31 min, calculated for $C_{17}H_{13}BrN_3O_2$ [M+H]$^+$ 370.02; found 370.25.

4-(1'-(3''-bromo-4''-formylphenyl)-1'*H*-imidazol-2'-yl)benzamide (17)

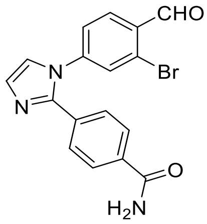

According to GP2, 1,2-disubstituted imidazole **17** was synthesized with 20% yield (19.3 mg, 52.1 µmol). Purification was performed by FCC (4 g silica, CH_2Cl_2/MeOH 0% → 11%).
TLC: R_f = 0.38 (MeOH/CH_2Cl_2, 1:19).
^{1}H-NMR (399 MHz, DMSO-d$_6$): δ = 10.22 (s, 1H), 8.00 (s, 1H), 7.88 (dd, J = 5.2, 3.1 Hz, 2H), 7.83 (d, J = 8.2 Hz, 2H), 7.71 (d, J = 1.4 Hz, 1H), 7.47 – 7.40 (m, 4H), 7.28 (d, J = 1.3 Hz, 1H).
^{13}C-NMR (100 MHz, DMSO-d$_6$): δ = 191.3, 167.7, 145.6, 143.5, 134.6, 132.8, 131.4, 131.0, 130.1, 128.8, 128.1, 126.5, 126.2, 124.4 ppm.
LC-MS (ESI-TOF): t_R = 6.33 min, calculated for $C_{17}H_{13}BrN_3O_2$ [M+H]+ 370.02; found 370.25.

2-bromo-4-(2'-(4''-isopropylphenyl)-1'*H*-imidazol-1'-yl)benzaldehyde (19)

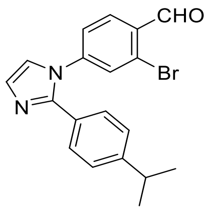

According to GP2, 1,2-disubstituted imidazole **19** was synthesized with 41% yield (81.0 mg, 219 µmol). Purification was performed by FCC (4 g silica, heptane/EtOAc 10% → 100%).
TLC: R_f = 0.49 (MeOH/CH_2Cl_2, 1:9).

^{1}H-NMR (399 MHz, DMSO-d$_6$): δ = 10.20 (d, J = 0.8 Hz, 1H), 7.86 (d, J = 8.3 Hz, 1H), 7.81 (d, J = 2.0 Hz, 1H), 7.63 (d, J = 1.4 Hz, 1H), 7.40 (ddd, J = 8.3, 2.1, 0.7 Hz, 1H), 7.30 – 7.20 (m, 5H), 2.88 (hept, J = 7.0 Hz, 1H), 1.19 (d, J = 6.9 Hz, 6H) ppm.
^{13}C-NMR (100 MHz, DMSO-d$_6$): δ = 190.7, 148.9, 145.8, 143.2, 132.1, 130.8, 130.3, 129.1, 128.4, 127.4, 126.3, 125.9, 125.5, 123.1, 33.0, 23.5 ppm.
LC-MS: t_R = 7.97 min, calculated for $C_{19}H_{18}BrN_2O$ $[M+H]^+$ 369.06; found 369.33.

2-bromo-4-(2'-(2''-chloro-4''-methylphenyl)-1'*H*-imidazol-1'-yl)benzaldehyde (87)

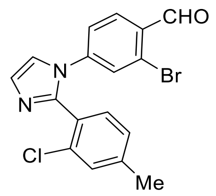

According to GP2, 1,2-disubstituted imidazole **87** was synthesized with 53% yield (104 mg, 277 µmol). Purification was performed by FCC (12 g silica, CH_2Cl_2/MeOH 0% → 6%).
TLC: R_f = 0.46 (MeOH/CH_2Cl_2, 1:9).
^{1}H-NMR (399 MHz, DMSO-d$_6$): δ = 10.13 (d, J = 0.7 Hz, 1H), 7.81 – 7.78 (m, 2H), 7.64 (d, J = 2.1 Hz, 1H), 7.49 (d, J = 7.7 Hz, 1H), 7.31 (dd, J = 1.6, 0.9 Hz, 1H), 7.29 – 7.28 (m, 1H), 7.27 – 7.26 (m, 1H), 7.26 (d, J = 1.4 Hz, 1H), 2.34 (s, 3H) ppm.
^{13}C-NMR (100 MHz, DMSO-d$_6$): δ = 191.1, 144.0, 143.0, 142.1, 133.1, 133.0, 132.2, 131.3, 130.3, 129.8, 129.5, 128.7, 127.4, 126.2, 124.2, 122.2, 21.0 ppm.
LC-MS (ESI-TOF): t_R = 8.71 min, calculated for $C_{17}H_{13}BrClN_2O$ $[M+H]^+$ 374.99; found 375.25.

2-bromo-4-(2'-(4''-(2'''-hydroxyethoxy)phenyl)-1'H-imidazol-1'-yl)benzaldehyde (24)

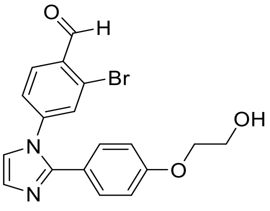

According to GP2, 1,2-disubstituted imidazole **24** was synthesized with 3% yield (5.30 mg, 13.7 µmol). Purification was performed by FCC (4 g silica, CH_2Cl_2/MeOH 2% → 7%).
TLC: R_f = 0.36 (MeOH/CH_2Cl_2, 1:9).
^{1}H-NMR (399 MHz, $CDCl_3$): δ = 10.35 (d, J = 0.8 Hz, 1H), 7.91 (d, 1H), 7.57 (d, J = 2.0 Hz, 1H), 7.31 (d, J = 8.8 Hz, 2H), 7.26 (s, 1H), 7.18 (d, J = 1.4 Hz, 1H), 7.12 (s, 1H), 6.85 (d, J = 8.8 Hz, 2H), 4.08 (dd, J = 5.2, 3.8 Hz, 2H), 3.97 (dd, J = 5.2, 3.8 Hz, 2H) ppm.
^{13}C-NMR (100 MHz, $CDCl_3$): δ = 190.6, 159.3, 143.8, 132.5, 130.7, 130.3, 130.2, 129.9, 126.7, 125.1, 122.4, 121.7, 115.0, 114.6, 69.2, 61.3 ppm.

2-bromo-4-(2'-(4''-(trifluoromethyl)phenyl)-1'*H*-imidazol-1'-yl)benzaldehyde (20)

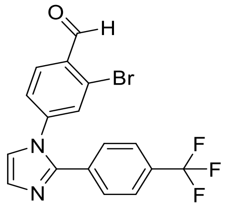

According to GP2, 1,2-disubstituted imidazole **20** was synthesized with 36% yield (67.5 mg, 171 µmol). Purification was performed by FCC (4 g silica, cyclohexane/EtOAc 20% → 80%).
^{1}H-NMR (399 MHz, $CDCl_3$): δ = 10.36 (s, 1H), 7.96 (d, *J* = 8.0, 1H), 7.60 (d, *J* = 3.99, 2H), 7.58 (s, 1H), 7.53 (s, 1H), 7.51 (s, 1H), 7.33 (s, 1H), 7.25 (s, 1H), 7.23 (s, 1H) ppm.
^{13}C-NMR (100 MHz, $CDCl_3$): δ = 166.7, 148.3, 144.8, 132.1, 130.6, 127.0, 124.4, 121.9, 121.1, 119.3 ppm.
^{19}F-NMR ($CDCl_3$, 376 MHz): δ = -56.43 ppm.
LC-MS: t_R = 9.50 min, calculated for $C_{17}H_{11}BrF_3N_2O$ $[M+H]^+$ 395.00: found 395.25.
HRMS (ESI-TOF) calculated for $C_{17}H_{11}BrF_3N_2O$ $[M+H]^+$ 395.0007; found 395.0006.

2-bromo-4-(2'-(4''-(trifluoromethoxy)phenyl)-1'*H*-imidazol-1'-yl)benzaldehyde (21)

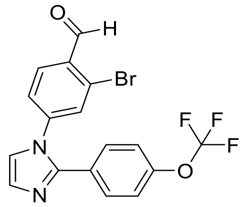

According to GP2, 1,2-disubstituted imidazole **21** was synthesized with 34% yield (61.2 mg, 151 µmol). Purification was performed by FCC (4 g silica, cyclohexane/EtOAc 20% → 80%).
^{1}H-NMR (399 MHz, $CDCl_3$): δ = 10.35 (s, 1H), 7.95 (d, *J* = 8.4 Hz,1H), 7.58 (d, *J* = 4.0 Hz, 1H), 7.44 (d, *J* = 8.8 Hz, 2H), 7.30 (d, *J* = 1.6 Hz, 1H), 7.27-7.24 (m, 1H), 7.22 (d, *J* = 2.0, 1H), 7.19 (d, *J* = 8.8, 2H) ppm.
^{13}C-NMR (100 MHz, $CDCl_3$): δ = 190.3, 149.7, 145.5, 143.4, 132.8, 130.9, 130.3, 130.3, 128.2, 127.5, 125.1, 124.2, 122.5, 121.6, 120.9, 119.1, 116.5 ppm.
LC-MS: t_R = 9.11 min, calculated for $C_{17}H_{11}BrF_3N_2O_2$ $[M+H]^+$ 411.00: found 411.25.
HRMS (ESI-TOF) calculated for $C_{17}H_{11}BrF_3N_2O_2$ $[M+H]^+$ 410.9956; found 410.9951.

2-bromo-4-(2'-(4''-(cyano)phenyl)-1'*H*-imidazol-1'-yl)benzaldehyde (22)

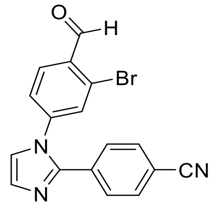

According to GP2, 1,2-disubstituted imidazole **22** was synthesized with 22% yield (46.0 mg, 131 µmol). Purification was performed by FCC (4 g silica, CH_2Cl_2/acetone 1% → 5%).
^{1}H-NMR (399 MHz, $CDCl_3$): δ = 10.37 (s, 1H), 7.93 (d, *J* = 8.0 Hz, 1H), 7.62 (s, 1H), 7.60 (s, 1H), 7.59 (s, 1H), 7.53 (s, 1H), 7.50 (s, 1H), 7.34 (s, 1H), 7.25 (s, 1H) ppm.
^{13}C-NMR (100 MHz, $CDCl_3$): δ = 190.1, 144.8, 143.1, 133.8, 133.2, 132.4, 131.1, 130.4, 129.0, 127.6, 125.1, 123.4, 118.3, 112.6 ppm.

LC-MS: t_R = 8.56 min, calculated for $C_{17}H_{11}BrN_3O$ $[M+H]^+$ 352.01: found 352.25.
HRMS (ESI-TOF) calculated for $C_{17}H_{11}BrN_3O$ $[M+H]^+$ 352.0085; found 352.0090.

2-bromo-4-(2'-(4''-fluoro-3``-trifluoromethoxy)phenyl)-1'*H*-imidazol-1'-yl)benzaldehyde (88)

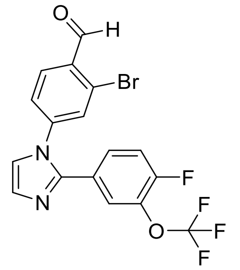

According to GP2, 1,2-disubstituted imidazole **88** was synthesized with 35% yield (61.4 mg, 143 µmol). Purification was performed by FCC (4 g silica, CH_2Cl_2/acetone 1% → 5%).
^{1}H-NMR (399 MHz, $CDCl_3$): δ = 10.37 (s, 1H), 8.00 (d, J =8.0 Hz, 1H), 7.57 (d, J = 2.4 Hz, 1H), 7.38-7.34 (m, 1H), 7.31 (s, 2H), 7.26 (s, 1H), 7.20 (dd, J = 17.2 Hz, 2H) ppm.
^{13}C-NMR (100 MHz, $CDCl_3$): δ = 190.2, 156.1, 153.5, 144.5, 143.0, 133.1, 131.1, 130.5, 130.3, 128.6, 128.6, 127.6, 126.6, 125.1, 124.2, 122.7, 117.9, 117.7 ppm.
^{19}F-NMR ($CDCl_3$, 377 MHz): δ = -58.59, -126.59 ppm.
LC-MS: t_R = 9.82 min, calculated for $C_{17}H_{10}BrF_4N_2O_2$ $[M+H]^+$ 428.99: found 429.25.

General Procedure 3 (GP3)

1,2-Benzenediamine (1 equiv.) and benzaldehyde (1 equiv.) were dissolved in DMF/H_2O (0.1 mol/L; 9/1 v/v) and the mixture was stirred on air at 80 °C for the given time. The solvents were removed under reduced pressure. The residue was purified by flash chromatography to yield the desired modified benzimidazoles.

2-(3`-methoxyphenyl)-benzimidazole (89)

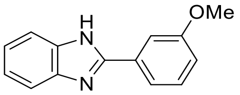

According to GP3, 3-methoxybenzaldehyde (446 µL, 3.67 mmol) and 1,2-benzenediamine (397 mg, 3.67 mmol) were dissolved in DMF (37 mL) and stirred at 80 °C for 21 h. Flash chromatography (40 g silica, heptane/EtOAc, 12% → 100%) afforded benzimidazole **89** as a yellow solid (82.9 mg, 370 µmol, 10%).
^{1}H-NMR (DMSO-d_6, 400 MHz): δ = 12.92-12.87 (s, 1H), 7.79-7.74 (d, J = 6.8 Hz, 2H), 7.70- 7.64 (d, J = 8.4 Hz, 1H), 7.56-7.51 (d, J = 7.6 Hz, 1H), 7.49-7.44 (t, J = 8.4 Hz, 1H), 7.26-7.16 (t, J = 8.0 Hz, 2H), 7.09-7.05 (q, J = 8.0 Hz, 1H), 3.90-3.85 (s, 3H) ppm.
^{13}C{1H}-NMR (DMSO-d_6, 100 MHz): δ = 160.1, 151.6, 144.2, 135.4, 131.9, 130.6, 123.1, 122.2, 119.3, 116.3, 111.8, 55.8 ppm.

2-(4`-methoxyphenyl)-benzimidazole (90)

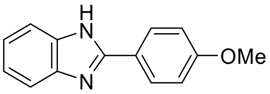

According to GP3, 4-methoxybenzaldehyde (446 µL, 3.67 mmol) and 1,2-benzenediamine (397 mg, 3.67 mmol) were dissolved in DMF (37 mL) and stirred at 80 °C for 28 h. Flash chromatography (40 g silica, heptane/EtOAc 12% → 100%) afforded benzimidazole **90** as a yellow solid (693 mg, 3.09 mmol, 84%).

^{1}H-NMR (DMSO-d$_6$, 400 MHz): δ = 12.77-12.70 (s, 1H), 8.16-8.10 (d, *J* = 8.8 Hz, 2H), 7.66- 7.60 (d, *J* = 7.2 Hz, 1H), 7.54-7.47 (d, *J* = 6.8 Hz, 1H), 7.22-7.15 (m, 2H), 7.14-7.09 (d, *J* = 9.2 Hz, 2H), 3.88-3.81 (s, 3H) ppm.

^{13}C{1H}-NMR (DMSO-d$_6$, 100 MHz): δ = 161.1, 151.8, 144.4, 135.5, 128.5, 123.2, 122.5, 121.9, 119.0, 114.8, 111.5, 55.8 ppm

2-(6`-Methoxy-3`-pyridinecarboxaldehyde)-benzimidazole (91)

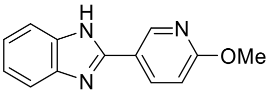

According to GP3, 6-methoxy-3-pyridinecarboxaldehyde (249 mg, 1.82 mmol) and 1,2-benzenediamine (197 mg, 1.82 mmol) were dissolved in DMF (37 mL) and stirred at 80 °C for 24 h. Flash chromatography (40 g silica, DCM/MeOH 0% → 10%) afforded benzimidazole **91** as a yellow solid (375 mg, 1.67 mmol, 92%).

^{1}H-NMR (DMSO-d$_6$, 400 MHz): δ = 13.0-12.8 (s, 1H), 8.98-8.95 (d, *J* = 2.4 Hz, 1H), 8.45-8.40 (q, *J* = 6.4 Hz, 1H), 7.71-7.64 (d, *J* = 7.2 Hz, 1H), 7.57-7.50 (d, *J* = 4.4 Hz, 1H), 7.26-7.26 (m, 2H), 7.04-6.99 (d, *J* = 8.8 Hz, 1H), 3.97-3.92 (s, 3H) ppm.

^{13}C{1H}-NMR (DMSO-d$_6$, 100 MHz): δ = 164.8, 149.7, 145.9, 144.2, 137.6, 135.3, 122.9, 122.2, 120.6, 119.2, 111.7, 111.4, 54.1 ppm

2-[4`-(Benzimidazol-2-yl)phenoxy]ethanol (92)

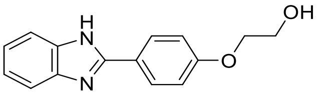

According to GP3, 4-(2-hydroxyethoxy)benzaldehyde (550 mg, 3.01 mmol) and 1,2-benzenediamine (325 mg, 3.01 mmol) were dissolved in DMF (37 mL) and stirred at 80 °C for 24 h. Flash chromatography (40 g silica, DCM/MeOH 0% → 10%) afforded benzimidazole **92** as a yellow solid (620 mg, 2.44 mmol, 81%).

^{1}H-NMR (DMSO-d$_6$, 400 MHz): δ = 12.92-12.57 (s, 1H), 8.19-8.05 (d, *J* = 8.0 Hz, 2H), 7.76- 7.37 (s, 2H), 7.23-7.06 (m, 4H), 5.07-4.81 (s, 1H), 4.20-4.00 (t, *J* = 2.6 Hz, 2H), 3.82-3.69 (s, 2H), ppm.

^{13}C{1H}-NMR (DMSO-d$_6$, 100 MHz): δ = 160.6, 151.9, 128.5, 123.1, 115.3, 70.2, 60.0 ppm

2-bromo-4-(2`-(3``-methoxyphenyl)-benzimidazole)-benzaldehyde (28)

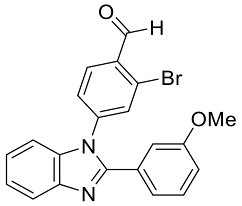

According to GP2, a mixture of 2-bromo-4-fluorobenzaldehyde (105 mg, 517 mmol), K_2CO_3 (71.7 mg, 518 mmol), 2-(3-methoxyphenyl)-benzimidazole (49.5 mg, 221 mmol) and DMSO (1 mL) was stirred for 60 minutes at 130 °C. Flash chromatography (24 g silica, heptane/EtOAc 12% → 100%) afforded benzimidazole **28** as a yellow solid (60.6 mg, 149 µmol, 67%).

^{1}H-NMR ($CDCl_3$, 400 MHz): *δ* = 10.41-10.38 (s, 1H), 8.04-7.99 (d, *J* = 8.4 Hz, 1H), 7.93-7.88 (d, *J* = 8.4 Hz, 1H), 7.71-7.67 (d, J = 2.8 Hz, 1H), 7.42-7.29 (m, 4H), 7.24-7.19 (d, *J* = 8.4 MHz, 2H), 6.70-6.93 (m, 2H), 3.84-3.74 (s, 3H) ppm.

^{13}C{1H}-NMR ($CDCl_3$, 100 MHz): *δ* = 190.5, 159.7, 152.0, 143.1, 142.7, 136.2, 132.9, 131.9, 131.0, 130.4, 129.7, 127.5, 126.8, 124.1, 123.8, 121.9, 120.4, 116.4, 114.6, 110.0, 55.4 ppm.

LC-MS: t_R = 10.35 min, calc for $C_{21}H_{16}N_2O_2Br$ $[M+H]^+$ 407.04: found 407.33.

2-bromo-4-(2`-(4``-methoxyphenyl)-benzimidazole)-benzaldehyde (29)

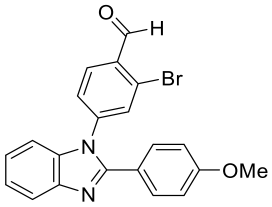

According to GP2, a mixture of 2-bromo-4-fluorobenzaldehyde (103 mg, 506 mmol), K_2CO_3 (71.1 mg, 514 mmol), 2-(4-methoxyphenyl)-benzimidazole (100 mg, 448 mmol) and DMSO (1 mL) was stirred for 60 minutes at 130 °C. Flash chromatography (24 g silica, heptane/EtOAc 12% → 100%) afforded benzimidazole **29** as a white powder (111 mg, 272 µmol, 61%).

^{1}H-NMR ($CDCl_3$, 400 MHz): *δ* = 10.42-10.39 (s, 1H), 8.05-7.99 (d, J = 8.0 Hz, 1H), 7.89-7.85 (d, *J* = 8.4 Hz, 1H), 7.71-7.68 (d, *J* = 2.0 Hz, 1H), 7.52-7.44 (dt, *J* = 9.2 Hz ,2H), 7.40-7.27 (m, 4H), 6.90-6.85 (dt, *J* = 8.8 Hz, 2H), 3.88-3.78 (s, 3H) ppm.

^{13}C{1H}-NMR ($CDCl_3$, 100 MHz): *δ* = 190.5, 161.0, 152.2, 143.3, 142.9, 136.1, 132.8, 132.0, 131.0, 127.6, 126.9, 123.7, 121.5, 120.0, 114.2, 109.8, 55.4 ppm.

LC-MS: t_R = 9.95 min, calculated for $C_{21}H_{16}N_2O_2Br$ $[M+H]^+$ 407.04: found 407.25.

2-bromo-4-(2`-(6``-methoxy-3``-pyridinecarboxaldehyde)-benzimidazole)-benzaldehyde (30)

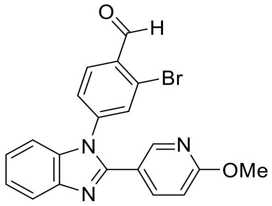

According to GP2, a mixture of 2-bromo-4-fluorobenzaldehyde (98.7 mg, 486 mmol), K_2CO_3 (67.1 mg, 486 mmol), 2-(6-methoxy-3-pyridinecarboxaldehyde)-benzimidazole (99.9 mg, 444 mmol) and DMSO (1 mL) was stirred for 60 minutes at 130 °C. Flash chromatography (24 g silica, heptane/EtOAc 12% → 100%) afforded benzimidazole **30** as a yellow solid (100 mg, 27 mmol, 61%).
^{1}H-NMR ($CDCl_3$, 400 MHz): δ = 10.42-10.39 (s, 1H), 8.26-8.22 (d, J = 2.8 Hz, 1H), 8.08-8.03 (d, J = 8.0 Hz, 1H), 7.90-7.83 (m, 2H), 7.74-7.70 (d, J = 2.0 Hz, 1H), 7.41-7.28 (m, 4H), 6.80-6.75 (d, J = 8.8 Hz, 1H), 3.99-3.91 (s, 3H) ppm.
^{13}C{1H}-NMR ($CDCl_3$, 100 MHz): δ = 190.3, 164.8, 147.8, 143.2, 142.4, 139.4, 136.2, 133.2, 132.0, 131.3, 127.8, 126.8, 124.1, 123.9, 120.2, 118.8, 111.27, 109.9, 53.9, 31.9 ppm.
LC-MS: t_R = 10.10 min, calculated for $C_{20}H_{15}N_3O_2Br$ $[M+H]^+$ 408.03: found 408.25.

2-bromo-4-(2`-[4``-(benzimidazol-2``-yl)phenoxy]ethanol)-benzaldehyde (31)

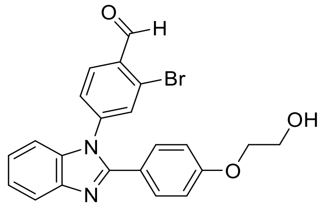

According to GP2, a mixture of 2-bromo-4-fluorobenzaldehyde (72.4 mg, 357 mmol), K_2CO_3 (49.2 mg, 356 mmol), 2-[4-(Benzimidazol-2-yl)phenoxy]ethanol (100 mg, 394 mmol) and DMSO (1 mL) was stirred for 60 minutes at 130 °C. Flash chromatography (24 g silica, heptane/EtOAc 12% → 100%) afforded benzimidazole **31** as a colorless foam (22.5 mg, 51 µmol, 14%).
^{1}H-NMR ($CDCl_3$, 400 MHz): δ = 10.43-10.35 (s, 1H), 8.05-7.97 (d, J = 8.0 Hz, 1H), 7.90-7.82 (d, J = 7.2 Hz, 1H), 7.72-7.64 (s, 1H), 7.51-7.42 (d, J = 9.2 Hz, 2H), 7.40-7.34 (m, 2H), 7.31-7.28 (m, 2H), 6.91-6.84 (d, J = 8.8 Hz, 2H), 4.09-4.06 (t, J = 4.8 Hz, 2H), 4.00-3.95 (t, J = 4.4 Hz, 2H), 2.66-2.51 (s, 1H), 1.88-1.64 (s, 1H) ppm.
^{13}C{1H}-NMR ($CDCl_3$, 100 MHz): δ = 190.5, 160.1, 152.0, 143.1, 142.8, 136.1, 132.9, 132.0, 131.1, 127.6, 126.8, 123.8, 121.8, 120.1, 114.7, 109.9, 69.4, 61.2 ppm.
LC-MS: t_R = 8.47 min, calculated for $C_{22}H_{18}N_2O_3Br$ $[M+H]^+$ 437.05: found 437.33.
HRMS (ESI-TOF) calculated for $C_{22}H_{18}N_2O_3Br$ $[M+H]^+$ 437.0501; found 437.0492.

2-bromo-4-(2-(piperidin-4-yl)-1H-imidazol-1-yl)benzaldehyde (93)

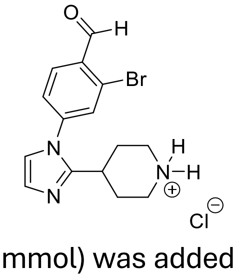

HCl in 1,4-dioxane (4M, 4.4 mL, 17.5 mmol) was added to Boc-protected amine (253 mg, 583 µmol) and the mixture was stirred 12 h at room temperature. The volatiles were evaporated to

obtain the ammonium chloride **93** that was used without further purification of characterization in the subsequent steps.

4-(2-(1-acetylpiperidin-4-yl)-1H-imidazol-1-yl)-2-bromobenzaldehyde (44)

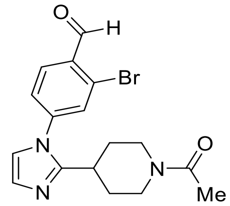

AcCl (5.08 µL, 71.8 µmol) and NEt_3 (16.7 µL, 120 µmol) were added to a solution of amine **93** (20.0 mg, 59.8 µmol) in THF (1 mL)and the mixture was stirred 2 h at room temperature. Water (5 mL) was added and the aqueous layer was extracted with EtOAc (4 x 10 mL). The combined organic extracts were treated with $MgSO_4$ and evaporated. Flash chromatography (4 g silica, CH_2Cl_2/MeOH 2% → 10%) afforded imidazole **44** (17.5 mg, 46.5 µmol, 78%).
^{1}H-NMR ($CDCl_3$, 400 MHz): δ = 10.41 (s, 1H), 8.08 (d, *J* = 8.3 Hz, 1H), 7.64 (d, *J* = 2.8 Hz, 1H), 7.40 (dd, *J* = 8.2, 2.3 Hz, 1H), 7.11 (d, *J* = 1.3 Hz, 1H), 7.00 (d, *J* = 1.3 Hz, 1H), 4.60 (d, *J* = 13.6 Hz, 1H), 3.90 (d, *J* = 13.0 Hz, 1H), 3.11 – 3.03 (m, 1H), 2.87 (ddt, *J* = 10.8, 8.0, 4.1 Hz, 1H), 2.60 (ddd, *J* = 14.4, 11.2, 4.3 Hz, 1H), 2.09 (s, 3H), 2.05 – 1.95 (m, 1H), 1.89 – 1.73 (m, 3H) ppm.
^{13}C{1H}-NMR ($CDCl_3$, 100 MHz): δ = 190.4, 168.9, 150.3, 143.1, 133.4, 131.2, 131.1, 129.0, 127.8, 125.4, 120.7, 46.2, 41.3, 33.9, 31.4 ppm.
LC-MS: t_R = 6.10 min, calc for $C_{17}H_{19}N_3O_2Br$ $[M+H]^+$ 376.07: found 376.17.

4-(1-(3-bromo-4-formylphenyl)-1H-imidazol-2-yl)piperidine-1-carboxamide (45)

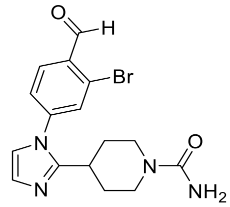

NaOCN (8.6 mg, 132 µmol) and Na_2CO_3 (6.3 mg, 59.8 µmol) were added to a solution of amine **93** (20.0 mg, 59.8 µmol) in THF/water (1 mL, 8:2 v/v)and the mixture was stirred 2 h at room temperature. Water (5 mL) was added and the aqueous layer was extracted with EtOAc (4 x 10 mL). The combined organic extracts were treated with $MgSO_4$ and evaporated. Flash chromatography (4 g silica, CH_2Cl_2/MeOH 2% → 15%) afforded imidazole **45** (10.4 mg, 27.5 µmol, 46%).
^{1}H-NMR ($CDCl_3$, 400 MHz): δ = 10.39 (s, 1H), 8.07 (d, *J* = 8.3 Hz, 1H), 7.63 (d, *J* = 2.1 Hz, 1H), 7.39 (dd, *J* = 8.2, 2.3 Hz, 1H), 7.10 (d, *J* = 1.5 Hz, 1H), 6.99 (d, *J* = 1.5 Hz, 1H), 4.51 (s, 2H), 3.98 (dt, *J* = 13.6, 4.0 Hz, 2H), 2.87– 2.78 (m, 3H), 1.99 – 1.87 (m, 2H), 1.83 – 1.74 (m, 2H) ppm.
^{13}C{1H}-NMR ($CDCl_3$, 100 MHz): δ = 190.4, 157.9, 150.4, 143.1, 133.4, 131.2, 131.0, 129.0, 127.7, 125.4, 120.7, 44.0, 33.8, 30.9 ppm.
LC-MS: t_R = 5.85 min, calc for $C_{16}H_{18}N_4O_2Br$ $[M+H]^+$ 377.06: found 377.08.

4-(1-(3-bromo-4-formylphenyl)-1*H*-imidazol-2-yl)-N-ethylpiperidine-1-carboxamide (46)

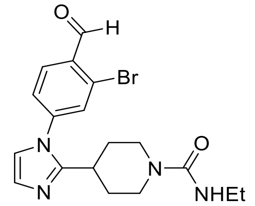

EtNCO (5.68 µL, 71.8 µmol) and Net_3 (16.7 µL, 120 µmol) were added to a solution of amine **93** (20.0 mg, 59.8 µmol) in THF (1 mL)and the mixture was stirred 2 h at room temperature. Water (5 mL) was added and the aqueous layer was extracted with EtOAc (4 x 10 mL). The combined organic extracts were treated with $MgSO_4$ and evaporated. Flash chromatography (4 g silica, CH_2Cl_2/MeOH 2% → 10%) afforded imidazole **46** (14.7 mg, 36.3 µmol, 61%).

^{1}H-NMR ($CDCl_3$, 399 MHz): δ = 10.40 (s, 1H), 8.07 (d, J = 8.2 Hz, 1H), 7.64 (d, J = 2.2 Hz, 1H), 7.40 (dd, J = 8.3, 2.3 Hz, 1H), 7.11 (d, J = 1.2 Hz, 1H), 7.01 – 6.99 (m, 1H), 4.43 (t, *J* = 5.6 Hz, 1H), 3.98 (dt, *J* = 13.5, 3.7 Hz, 2H), 3.31 – 3.17 (m, 2H), 2.86 – 2.74 (m, 3H), 1.97 – 1.85 (m, 2H), 1.78 (dd, *J* = 13.6, 3.7 Hz, 2H), 1.13 (t, *J* = 7.2 Hz, 3H) ppm.

^{13}C{1H}-NMR ($CDCl_3$, 100 MHz): δ = 190.4, 157.6, 150.6, 143.2, 133.3, 131.2, 131.0, 129.0, 127.7, 125.4, 120.6, 43.8, 35.9, 34.1, 31.0, 15.7 ppm.

LC-MS: t_R = 6.24 min, calc for $C_{18}H_{22}N_4O_2Br$ $[M+H]^+$ 405.09: found 405.25.

(R)-2-bromo-4-(2-(pyrrolidin-2-yl)-1H-imidazol-1-yl)benzaldehyde (94)

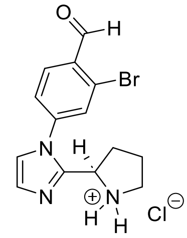

HCl in 1,4-dioxane (4M, 2.9 mL, 11.6 mmol) was added to Boc-protected amine (163 mg, 388 µmol) and the mixture was stirred 12 h at room temperature. The volatiles were evaporated to obtain the ammonium chloride **94** that was used without further purification of characterization in the subsequent steps.

(*R*)-4-(2-(1-acetylpyrrolidin-2-yl)-1H-imidazol-1-yl)-2-bromobenzaldehyde (42)

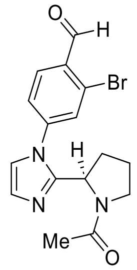

AcCl (7.9 mg, 101 µmol) and NEt_3 (35.2 µL, 252 µmol) were added to a solution of amine **94** (30.0 mg, 84.1 µmol) in THF (1 mL)and the mixture was stirred 2 h at room temperature. Water

(5 mL) was added and the aqueous layer was extracted with EtOAc (4 x 10 mL). The combined organic extracts were treated with $MgSO_4$ and evaporated. Flash chromatography (4 g silica, CH_2Cl_2/MeOH 2% → 10%) afforded imidazole **42** (17.2 mg, 47.5 µmol, 56%).
^{1}H-NMR ($CDCl_3$, 400 MHz): δ = 10.37 (d, *J* = 0.9 Hz, 1H), 8.03 (d, J = 8.2 Hz, 1H), 7.95 (d, *J* = 2.1 Hz, 1H), 7.72 (ddd, *J* = 8.3, 2.1, 1.0 Hz, 1H), 7.09 (d, *J* = 1.5 Hz, 1H), 6.95 (d, *J* = 1.5 Hz, 1H), 5.01 (dd, *J* = 8.3, 3.7 Hz, 1H), 3.86 (ddd, *J* = 9.9, 5.0, 3.1 Hz, 1H), 3.53 (dt, *J* = 9.5, 7.1 Hz, 1H), 2.54 – 2.44 (m, 1H), 2.13 – 2.06 (m, 1H), 2.05 (s, 3H), 2.01 – 1.94 (m, 2H) ppm.
^{13}C{1H}-NMR ($CDCl_3$, 100 MHz): δ = 190.7, 169.5, 149.4, 143.3, 133.1, 131.4, 130.9, 129.0, 127.5, 126.0, 120.2, 52.5, 48.1, 32.31, 24.7, 22.7 ppm.
LC-MS: t_R = 6.10 min, calc for $C_{16}H_{17}N_3O_2Br$ $[M+H]^+$ 362.05: found 362.25.

(R)-2-(1-(3-bromo-4-formylphenyl)-1H-imidazol-2-yl)-N-ethylpyrrolidine-1-carboxamide (43)

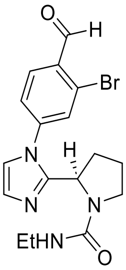

EtNCO (7.99 µL, 101 µmol) and NEt_3 (35.2 µL, 252 µmol) were added to a solution of amine **94** (30.0 mg, 84.1 µmol) in THF (1 mL)and the mixture was stirred 2 h at room temperature. Water (5 mL) was added and the aqueous layer was extracted with EtOAc (4 x 10 mL). The combined organic extracts were treated with $MgSO_4$ and evaporated. Flash chromatography (4 g silica, CH_2Cl_2/MeOH 2% → 10%) afforded imidazole **43** (22.2 mg, 56.7 µmol, 67%).
^{1}H-NMR ($CDCl_3$, 400 MHz): δ = 10.38 (s, 1H), 8.04 (d, *J* = 8.3 Hz, 1H), 7.90 (d, *J* = 2.1 Hz, 1H), 7.77 (ddd, *J* = 8.3, 2.0, 0.8 Hz, 1H), 7.10 (d, *J* = 1.5 Hz, 1H), 6.97 (d, *J* = 1.5 Hz, 1H), 5.07 (dd, *J* = 7.8, 3.8 Hz, 1H), 4.44 (dt, *J* = 38.3, 5.5 Hz, 1H), 3.79 – 3.70 (m, 1H), 3.37 (q, *J* = 7.2 Hz, 1H), 3.28 – 3.16 (m, 2H), 2.43 – 2.30 (m, 1H), 2.14 – 2.04 (m, 1H), 1.99 (ddd, *J* = 11.9, 5.9, 3.8 Hz, 1H), 1.92 – 1.85 (m, 1H), 1.11 (t, *J* = 7.3 Hz, 3H) ppm.
^{13}C{1H}-NMR ($CDCl_3$, 100 MHz): δ = 190.6, 156.6, 150.0, 143.1, 132.9, 131.1, 130.9, 128.8, 128.8, 127.4, 125.9, 120.4, 53.1, 46.0, 35.4, 32.5, 24.5, 15.6 ppm.
LC-MS: t_R = 6.25 min, calc for $C_{17}H_{20}N_4O_2Br$ $[M+H]^+$ 391.08: found 391.08.

Supporting Tables

SI Table S1. Focused library profiling potency and stabilization factors for the stabilization of the 14-3-3:tau_pS214_pS324 interaction.

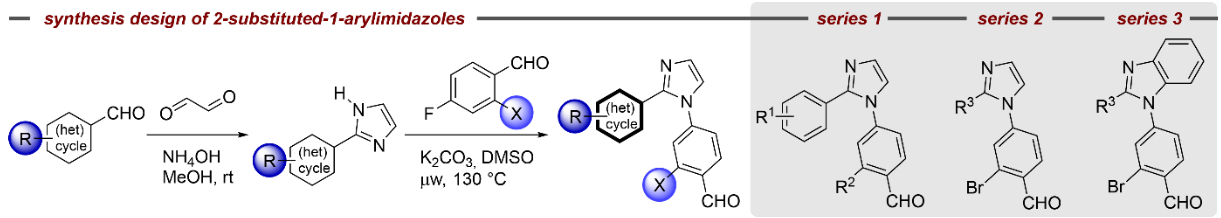

series 1

compound	R_1	R_2	CC_{50} (µM)	apparent K_D (nM)	SF
**1**	H	Br	122 ± 57	40 ± 6	23
**3**	*o*-Me	Br	149 ± 64	83 ± 4	11
**4**	*o*-OMe	Br	-	-	-
**5**	*o*-Cl	Br	105 ± 5	141 ± 47	7.5
**6**	*m*-Me	Br	160 ± 110	94 ± 12	10
**7**	*m*-OMe	Br	-	-	-
**8**	*m*-Cl	Br	-	-	-
**9**	*m*-OH	Br	-	-	-
**10**	*m*-C(=O)NH_2	Br	-	-	-
**11**	*p*-Me	Br	135 ± 78	32 ± 4	29
**12**	*p*-OMe	Br	-	-	-
**13**	*p*-F	Br	71 ± 19	22 ± 2	33
**14**	*p*-Cl	Br	69 ± 14	84 ± 20	8.9
**15**	*p*-I	Br	23 ± 5	245 ± 65	3.1
**16**	*p*-OH	Br	-	-	-
**17**	*p*-C(=O)NH_2	Br	-	-	-
**18**	*p*-Et	Br	15 ± 5	31 ± 8	37
**19**	*p*-*i*Pr	Br	-	-	-
**20**	*p*-CF_3	Br	65 ± 5	86 ± 4	13
**21**	*p*-OCF_3	Br	75 ± 7	103 ± 3	11
**22**	*p*-CN	Br	46 ± 39	44 ± 24	34
**23**	*p*-morpholino	Br	68 ± 25	70 ± 0.1	16
**24**	p-$O(CH_2)_2OH$	Br	-	-	-
**25**	H	Cl	31 ± 49	69 ± 6	15
**26**	H	I	n.d.	594 ± 120	1.8
**27**	H	CF_3	n.d.	175 ± 57	6.1

series 2

compound	R_3	CC_{50} (µM)	apparent K_D (nM)	SF
**32**	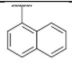	-	-	-
**33**	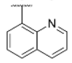	187 ± 42	124 ± 48	4.3
**34**	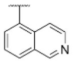	46 ± 14	92 ± 40	5.8
**35**	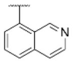	-	-	-
**36**	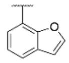	-	-	-
**37**	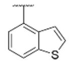	-	-	-
**38**	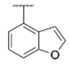	-	-	-
**39**	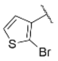	-	-	-
**40**	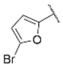	-	-	-
**41**	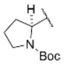	-	-	-
**42**	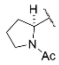	-	-	-
**43**	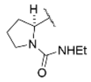	-	-	-
**44**	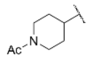	-	-	-
**45**	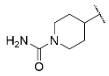	-	-	-
**46**	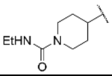	-	-	-

series 3

compound	R_3	CC_{50} (µM)	apparent K_D (nM)	SF
**2**	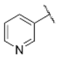	85 ± 36	45 ± 11	20
**28**	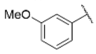	-	-	-
**29**	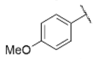	26 ± 3	63 ± 21	8.3
**30**	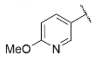	-	-	-
**31**	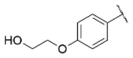	24 ± 9	27 ± 16	19

SI Table S2 Data collection and refinement statistics of crystal structures.

PDB	***9FVH***	***9FVI***	***9FVP***	***9FVG***
Protein	14-3-3σΔc	14-3-3σΔc	14-3-3σΔc	14-3-3σΔc
Peptide	Tau pS214	Tau pS214	Tau pS214	Tau pS214
Compound	**3**	6	**11**	**18**
Beam	ID30B	ID30A-3	ID23-1	ID30B
Data collection				
Wavelength (Å)	0.8731	0.9677	0.8856	0.8731
Space group	C 2 2 21	C 2 2 21	C 2 2 21	C 2 2 21
Cell dimensions				
a, b, c (Å)	82.1, 112.4, 62.4	82.1, 112.5, 62.4	82.3, 112.5, 62.6	82.1, 112.1, 62.6
α, β, γ (°)	90, 90, 90	90, 90, 90	90, 90, 90	90, 90, 90
Resolution (Å)	66.30-1.45 (1.47-1.45)	62.35-1.55 (1.58-1.55)	66.42-1.58 (1.61-1.58)	66.22-1.45 (1.47-1.45)
I / σ(I)	24.9 (4.4)	25.2 (4.3)	21.2 (4.8)	22.6 (3.8)
Completeness (%)	100.0 (100.0)	99.8 (99.8)	94.3 (100.0)	100.0 (100.0)
Redundancy	14.0 (12.9)	13.7 (14.2)	12.9 (13.6)	14.1 (12.9)
$CC_{1/2}$	0.999 (0.947)	0.999 (0.943)	0.999 (0.946)	1.000 (0.936)
Refinement				
No. reflections	51458	42025	37872	51426
R_{work}/R_{free}	0.148 / 0.176	0.172 / 0.204	0.183 / 0.206	0.162 / 0.192
No. atoms				
Overall	2205	2143	2065	2218
Solvent	310	256	233	354
B-factors				
Overall	21.10	23.67	24.46	19.67
R.m.s. deviations				
Bond lengths (Å)	0.0125	0.0122	0.0114	0.0122
Bond angles (°)	1.923	1.745	1.713	1.800
Ramachandran				
favored (%)	96.96	97.35	98.23	97.39
outliers (%)	0.43	0.44	0.44	0.43

SI Table S3 Data collection and refinement statistics of crystal structures.

PDB	***9FVN***	***9FS4***	***9GFA***
Protein	14-3-3σΔc	14-3-3σΔc	14-3-3σΔc
Peptide	Tau pS214	Tau pS214	Tau pS214
Compound	**31**	1	**2**
Beam	ID30B	ID23-1	ID30B
Data collection			
Wavelength (Å)	0.8731	0.972425	0.885601
Space group	C 2 2 21	C 2 2 21	C 2 2 21
Cell dimensions			
a, b, c (Å)	82.2, 112.0, 62.3	82.3, 112.2, 62.4	82.4, 112.6, 62.5
α, β, γ (°)	90, 90, 90	90.0, 90.0, 90.0	90.0, 90.0, 90.0
Resolution (Å)	66.27-1.50 (1.53-1.50)	66.39-1.6 (1.63-1.6)	66.48-1.6 (1.63-1.6)
I / σ(I)	20.6 (4.2)	27.4 (5)	22.6 (5.4)
Completeness (%)	100.0 (100.0)	91.1 (100)	100 (100)
Redundancy	7.8 (7.6)	13.1 (13.5)	11.7 (11.5)
$CC_{1/2}$	0.999 (0.940)	0.999 (0.965)	0.999 (0.957)
Refinement			
No. reflections	46289	35107	38668
R_{work}/R_{free}	0.184 / 0.206	0.178 / 0.200	0.176 / 0.191
No. atoms			
Overall	2096	2105	2087
Solvent	302	203	181
B-factors			
Overall	15.21	20.27	17.16
R.m.s. deviations			
Bond lengths (Å)	0.0128	0.0142	0.0151
Bond angles (°)	1.823	2.05	1.860
Ramachandran			
favored (%)	97.35	95.98	97.77
outliers (%)	0.44	0.89	0.45

Supporting Figures

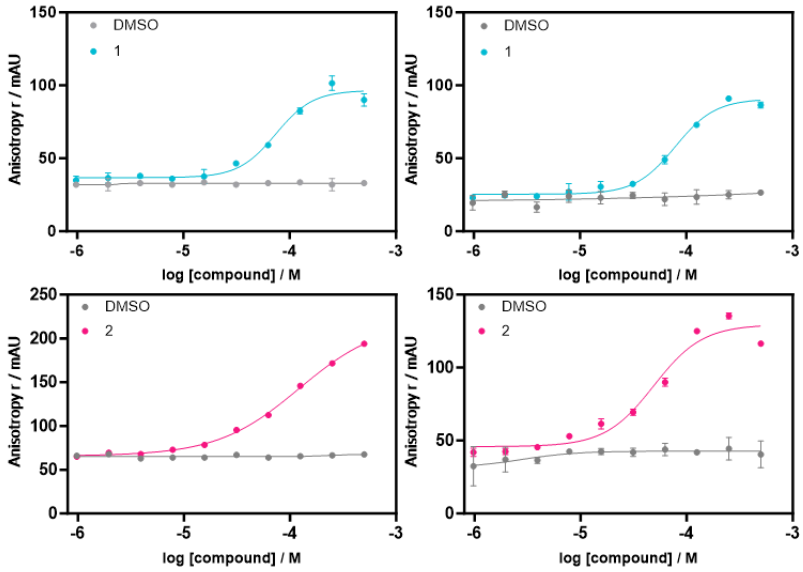

SI Figure S1 Dose-response fluorescence anisotropy replicate data of compound **1** & **2** titrations and DMSO as negative control to 10 nM FITC-labeled Tau pS2 peptide and 100 nM 14-3-3ζ. Background (without 14-3-3ζ) subtracted, overnight measurement, mean and SD of technical duplicate

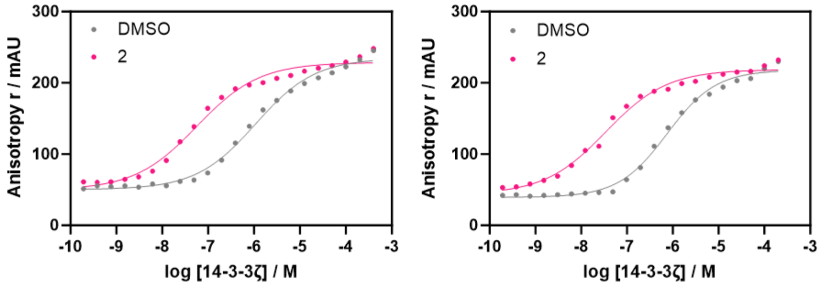

SI Figure S2 Dose-response fluorescence anisotropy replicate data of 14-3-3ζ titrations to 10 nM FITC-labeled Tau pS2 peptide and 250 µM compound **2**, data of technical singlets.

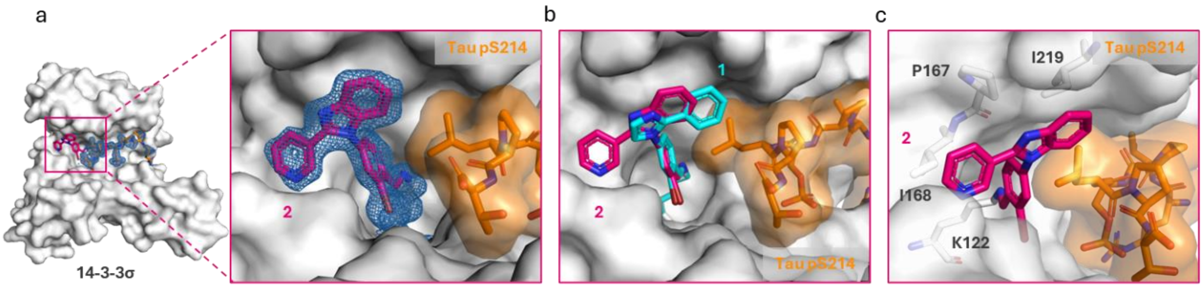

SI Figure S3 a, Ternary structure of 14-3-3σΔC (white surface), Tau pS214 (orange sticks) and **2** (hotpink sticks) complex. Zoom-in of the crystallized ternary complex **2** (hotpink sticks)/14-3-3σΔC (white surface)/Tau pS214 (orange sticks). 2Fo – Fc electron density map (blue mesh) is contoured at 1σ. **b,** Overlay of the ternary structure of 14-3-3σΔC/Tau pS214/**2** complex with compound **1** (PDB ID: 9FS4). **c**, The occupation of the hydrophobic entrance of the binding groove of 14-3-3 (white sticks) by compound **2**. PDB ID: 9GFA.

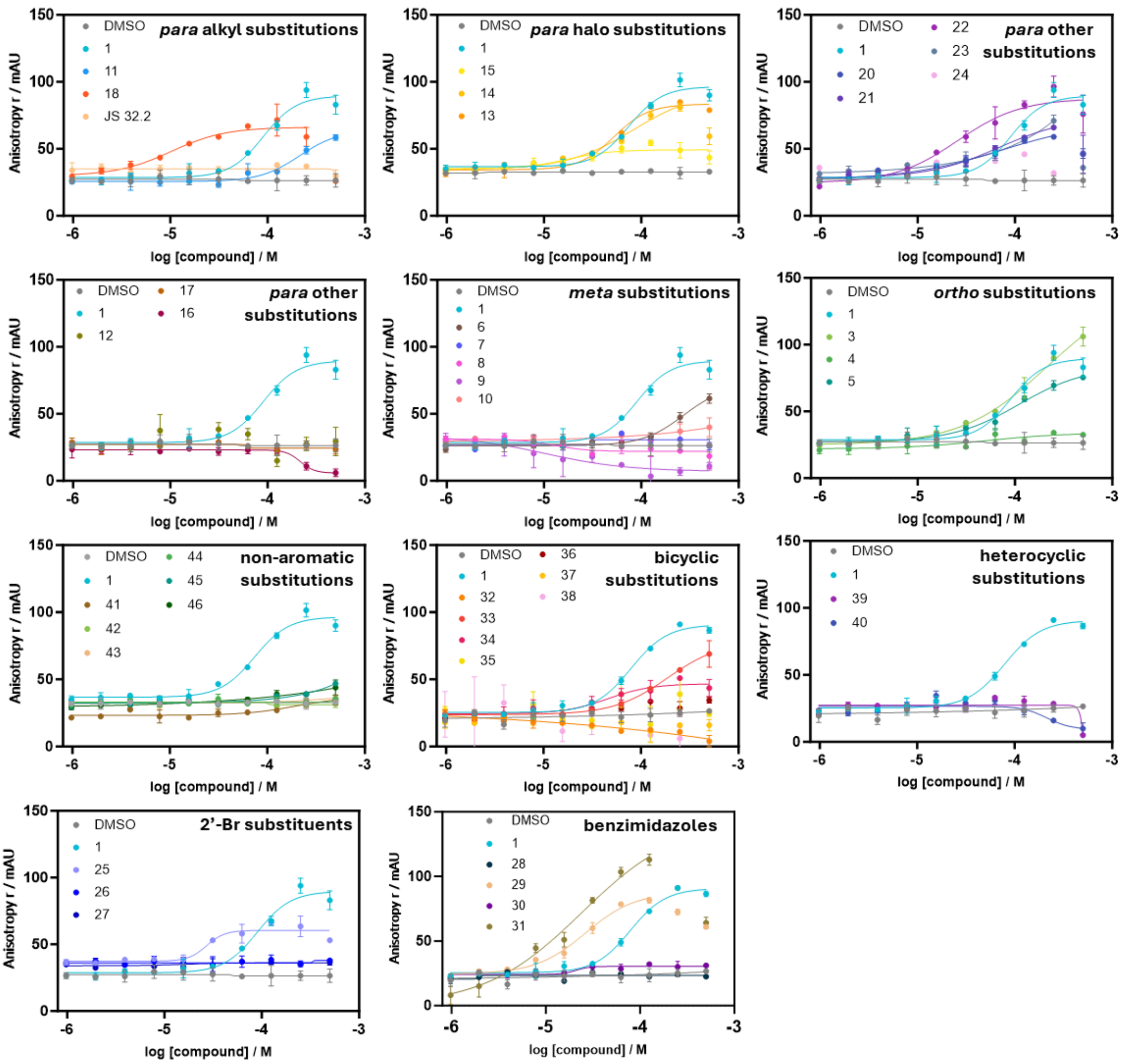

SI Figure S4 Initial compound screening. Dose-response fluorescence anisotropy data of compound titrations and DMSO as negative control to 10 nM FITC-labeled Tau pS2 peptide and 100 nM 14-3-3ζ. Background (without 14-3-3ζ) subtracted, overnight measurement, mean and SD of technical duplicate

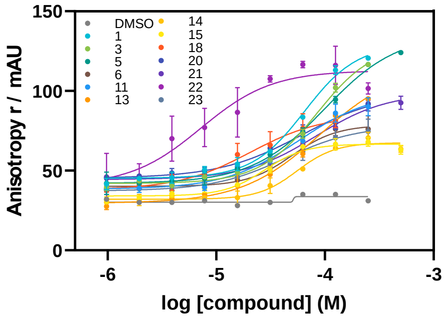

SI Figure S5 Dose-response fluorescence anisotropy replicate data of hit compound titrations and DMSO as negative control to 10 nM FITC-labeled Tau pS2 peptide and 100 nM 14-3-3ζ. Background (without 14-3-3ζ) subtracted, overnight measurement, mean and SD of technical duplicate

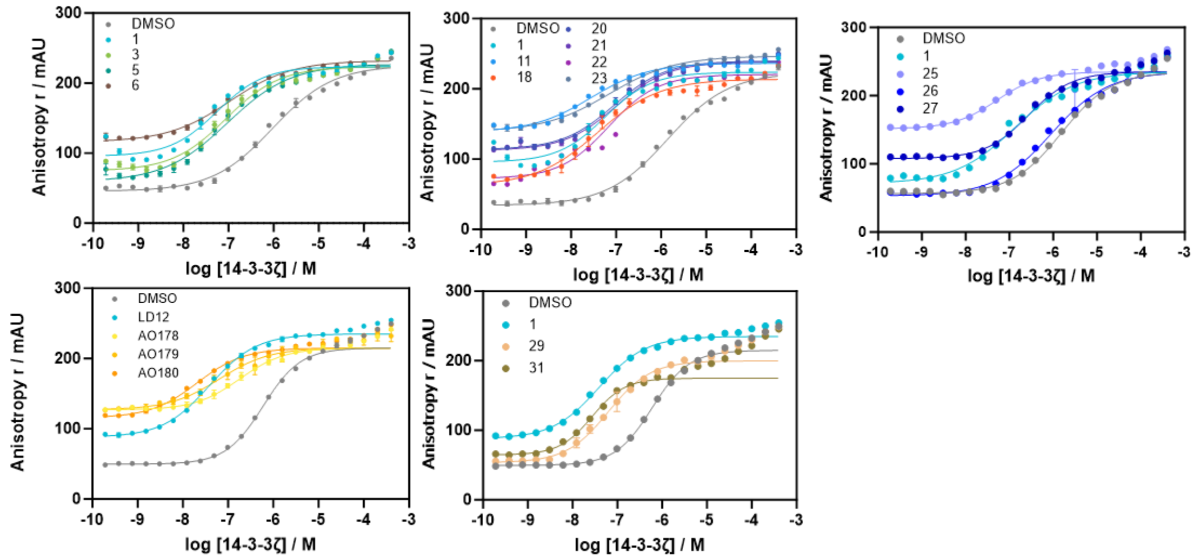

SI Figure S6 Dose-response fluorescence anisotropy replicate data of 14-3-3ζ titrations to 10 nM FITC-labeled Tau pS2 peptide and 250 µM compound, mean and SD of technical duplicate.

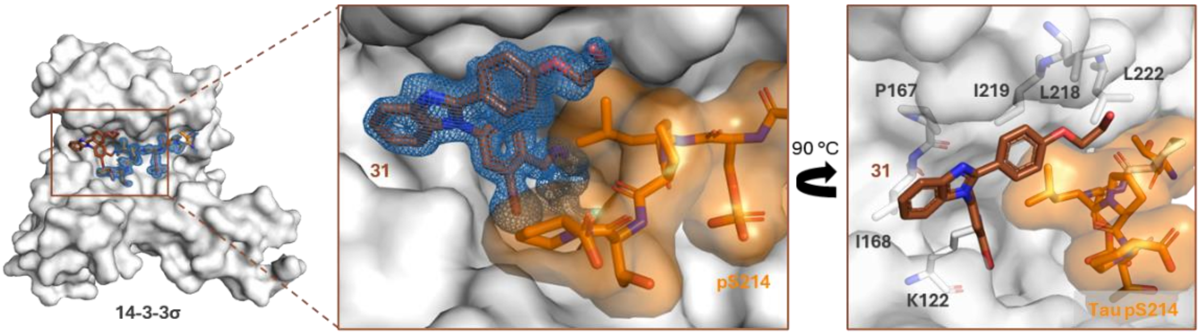

SI Figure S7 a, Ternary structure of 14-3-3σΔC (white surface), Tau pS214 (orange sticks) and **31** (brown sticks) complex. Zoom-in of the crystallized ternary complex **31** (brown sticks)/14-3-3σΔC (white surface)/Tau pS214 (orange sticks). 2Fo – Fc electron density map (blue mesh) is contoured at 1σ. **b,** The occupation of the hydrophobic groove of 14-3-3 (white sticks) by compound **31**. PDB ID: 9FVN.

C-Raf 233

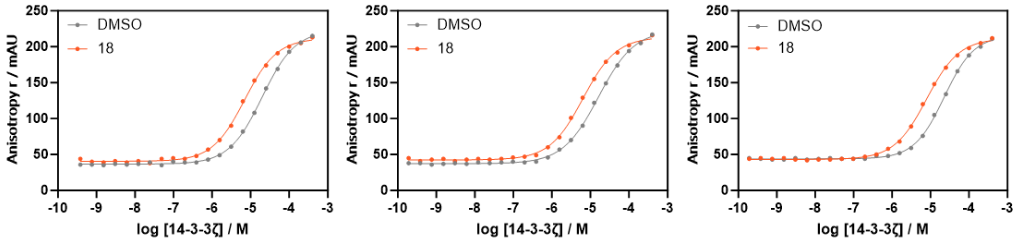

C-Raf 259

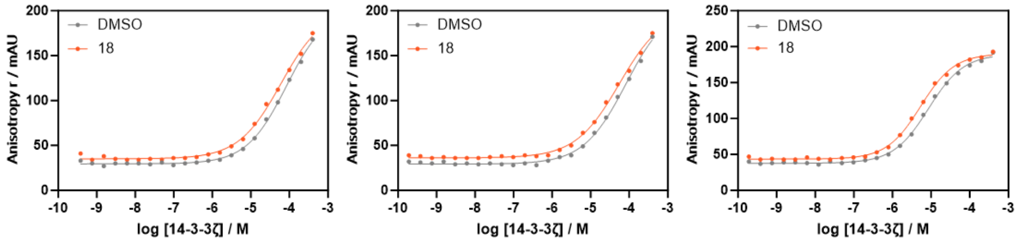

CFTR

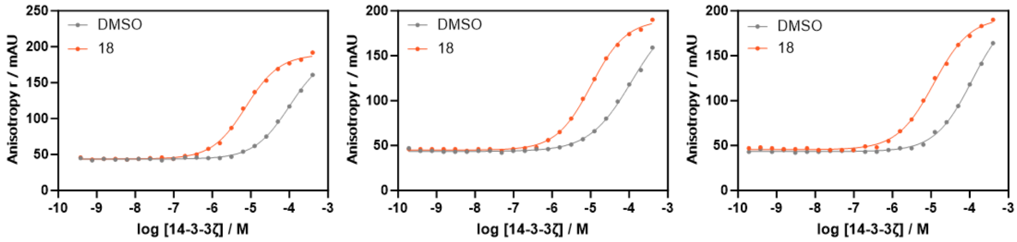

Pin1

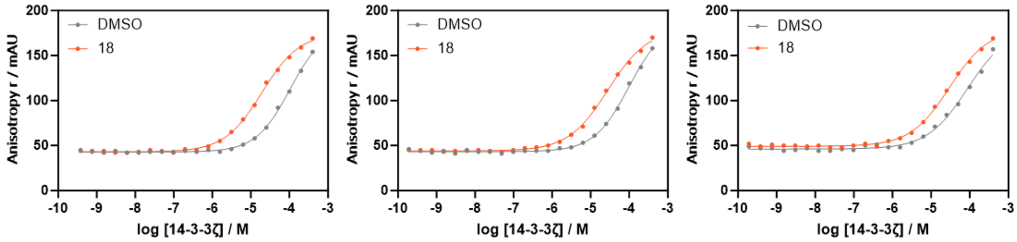

Rptor

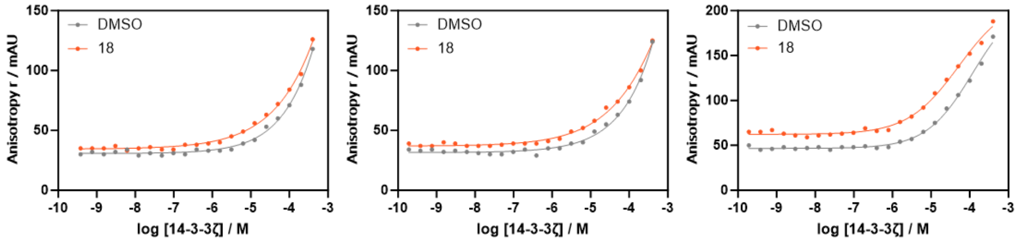

ERα

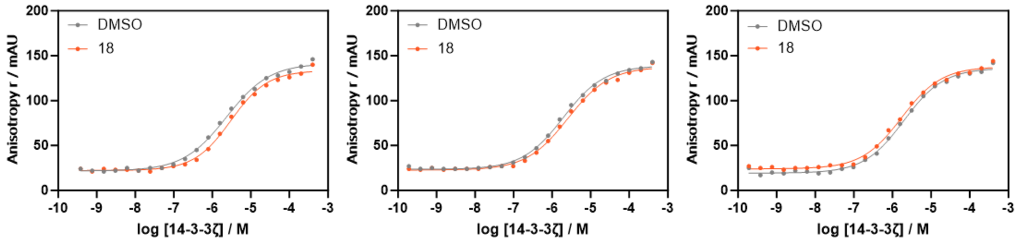

Rubic

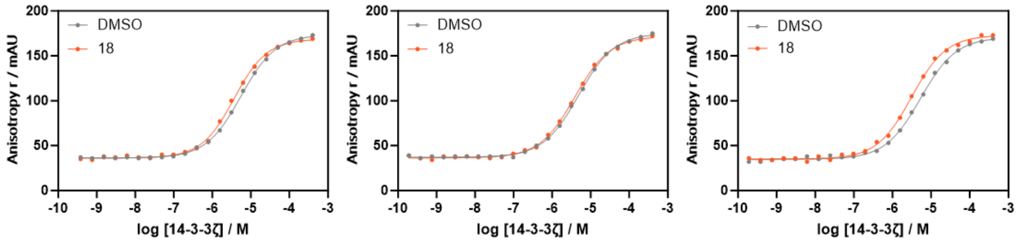

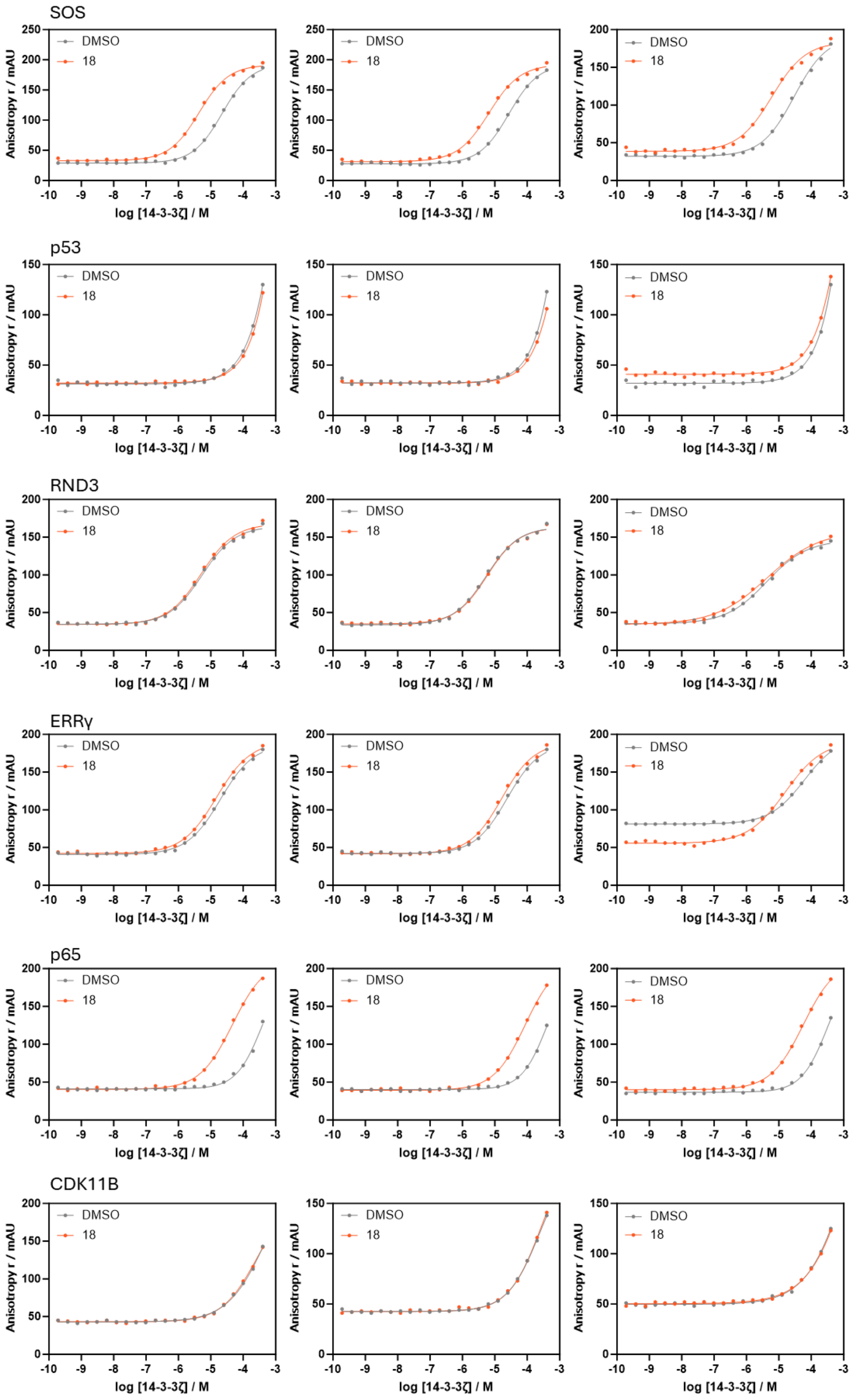

SI Figure S8 Dose-response fluorescence anisotropy data of 14-3-3ζ titrations to 10 nM FITC-labeled Tau pS2 peptide and 250 μM compound, data shown of three separately performed experiments.

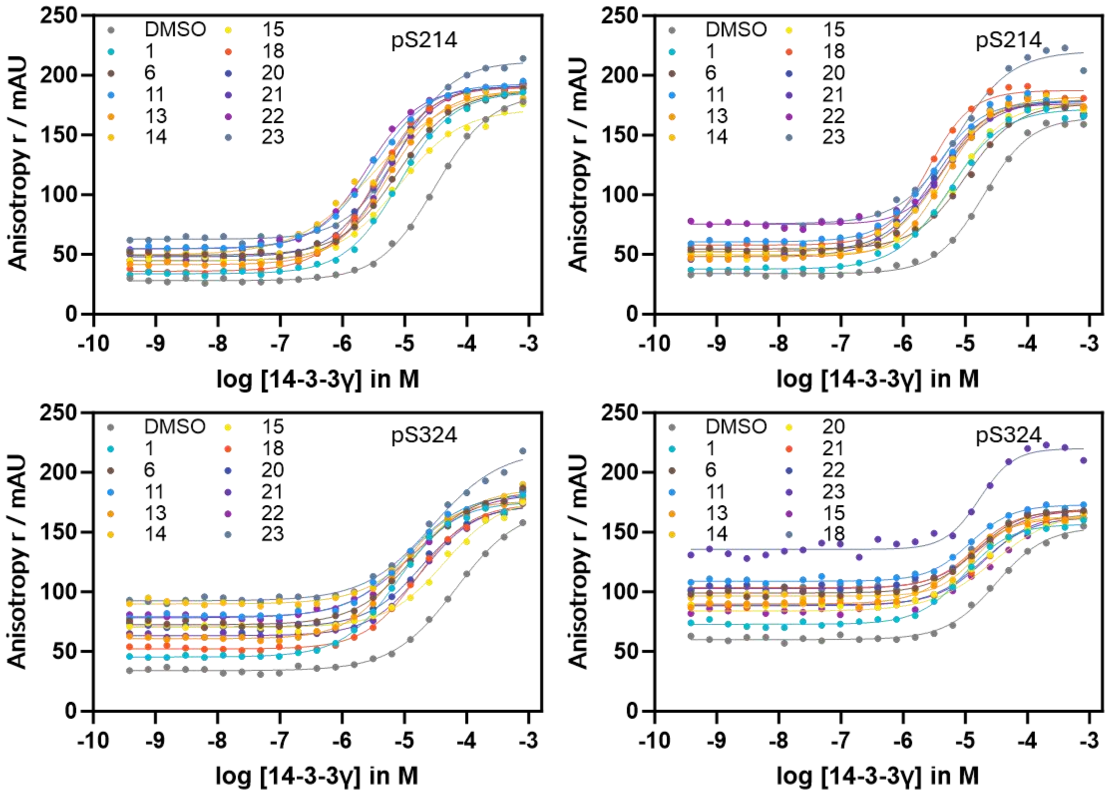

SI Figure S9 Dose-response fluorescence anisotropy data of 14-3-3γ titrations to 10 nM FITC-labeled Tau pS214 or pS324 peptide and 250 μM compound, data shown of two separately performed experiments.

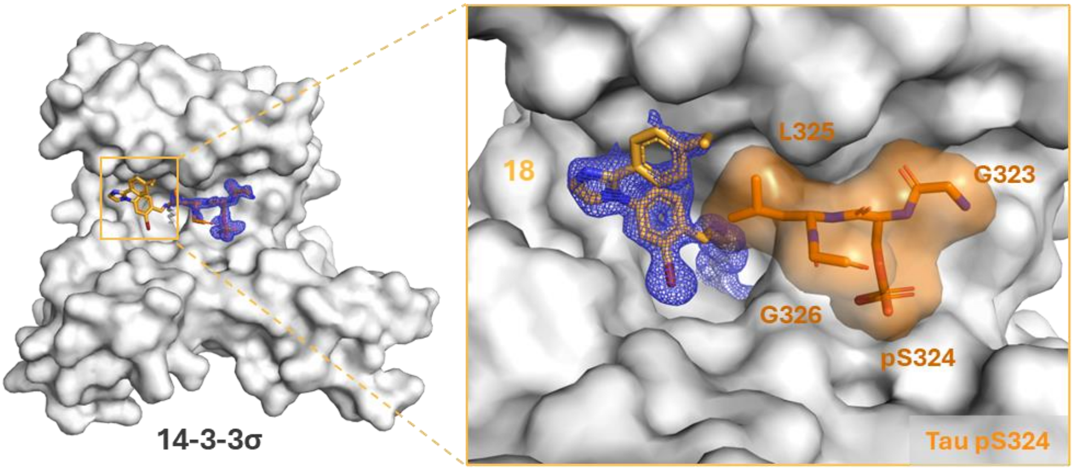

SI Figure S10 Ternary structure of 14-3-3σΔC (white surface), Tau pS324 (orange sticks) and **18** (light orange sticks) complex. Zoom-in of the crystallized ternary complex **18** (light orange sticks)/14-3-3σΔC (white surface)/Tau pS324 (orange sticks). 2Fo – Fc electron density map (blue mesh) is contoured at 1σ.

NMR Spectra

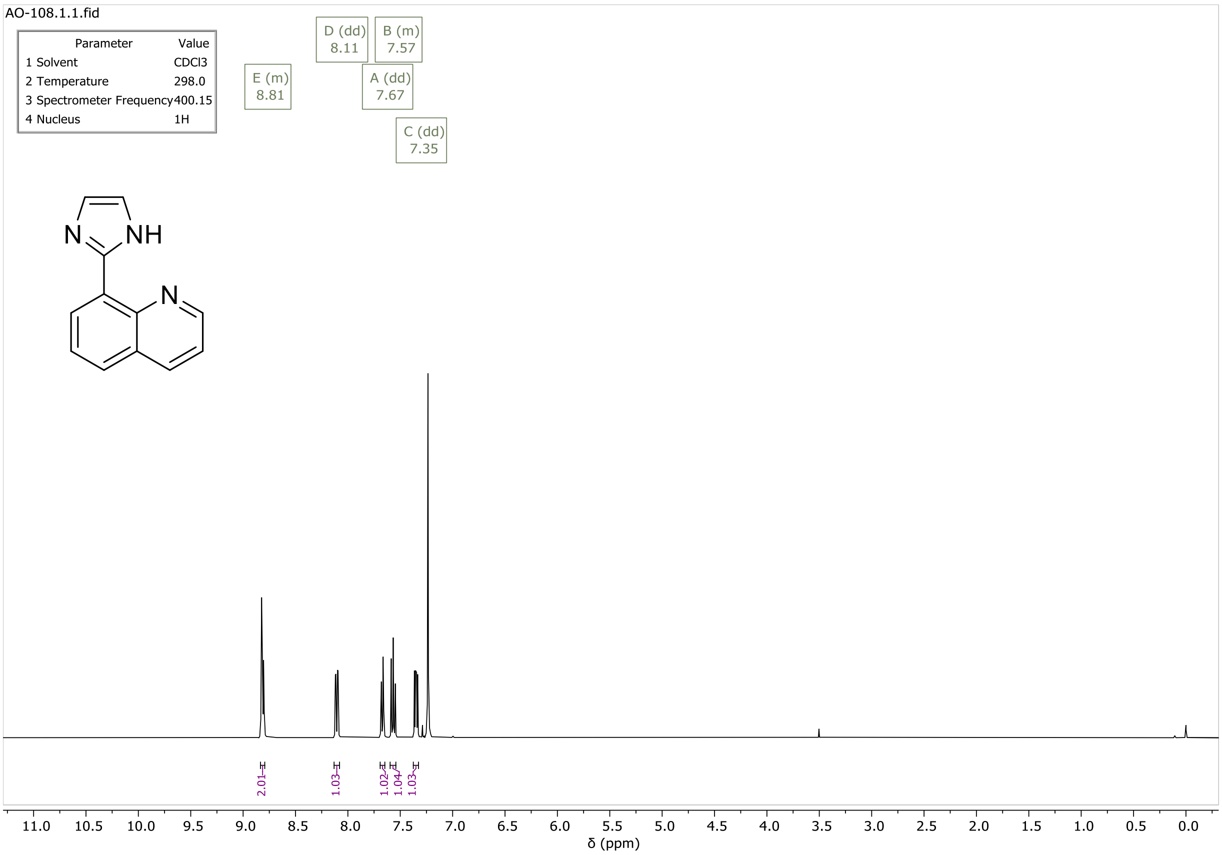

SI Figure S11: ^{1}H-NMR spectrum of imidazole **51**.

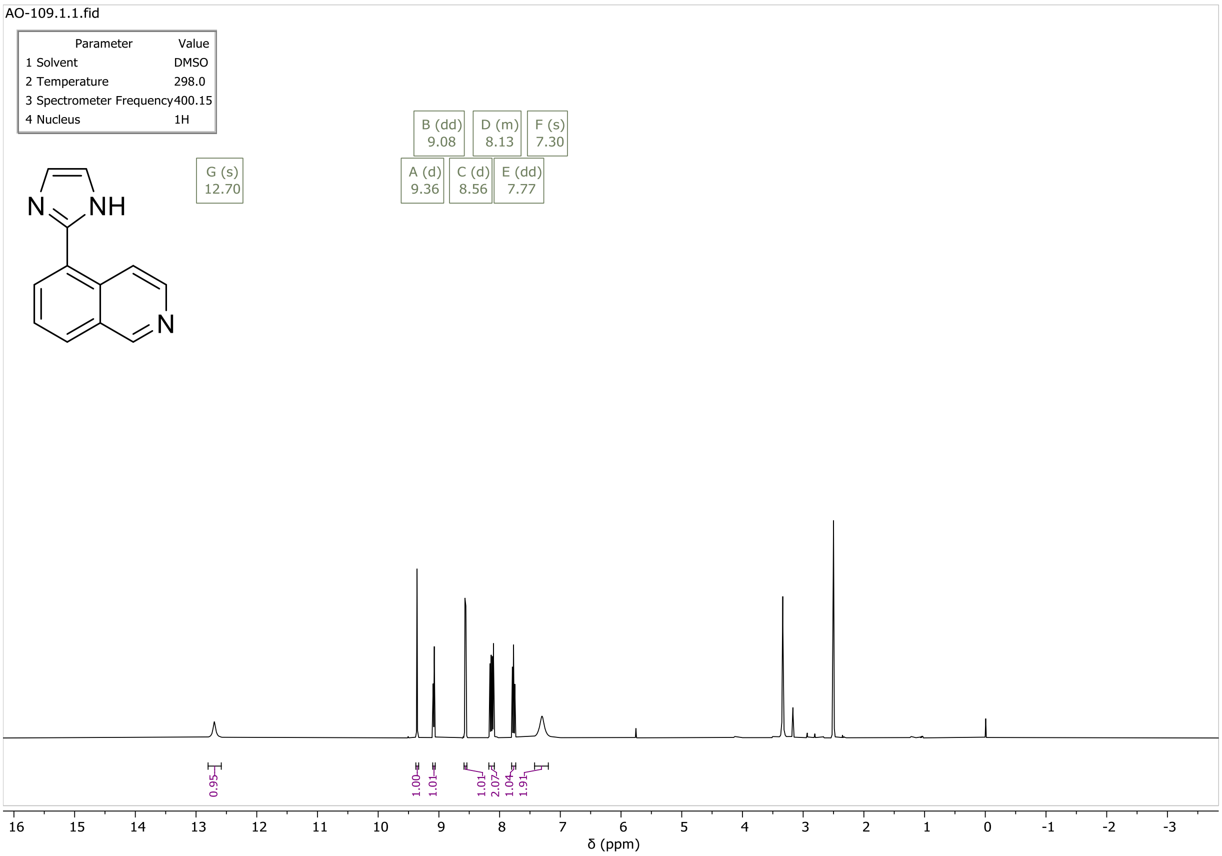

SI Figure S12. ^{1}H-NMR spectrum of imidazole **52**.

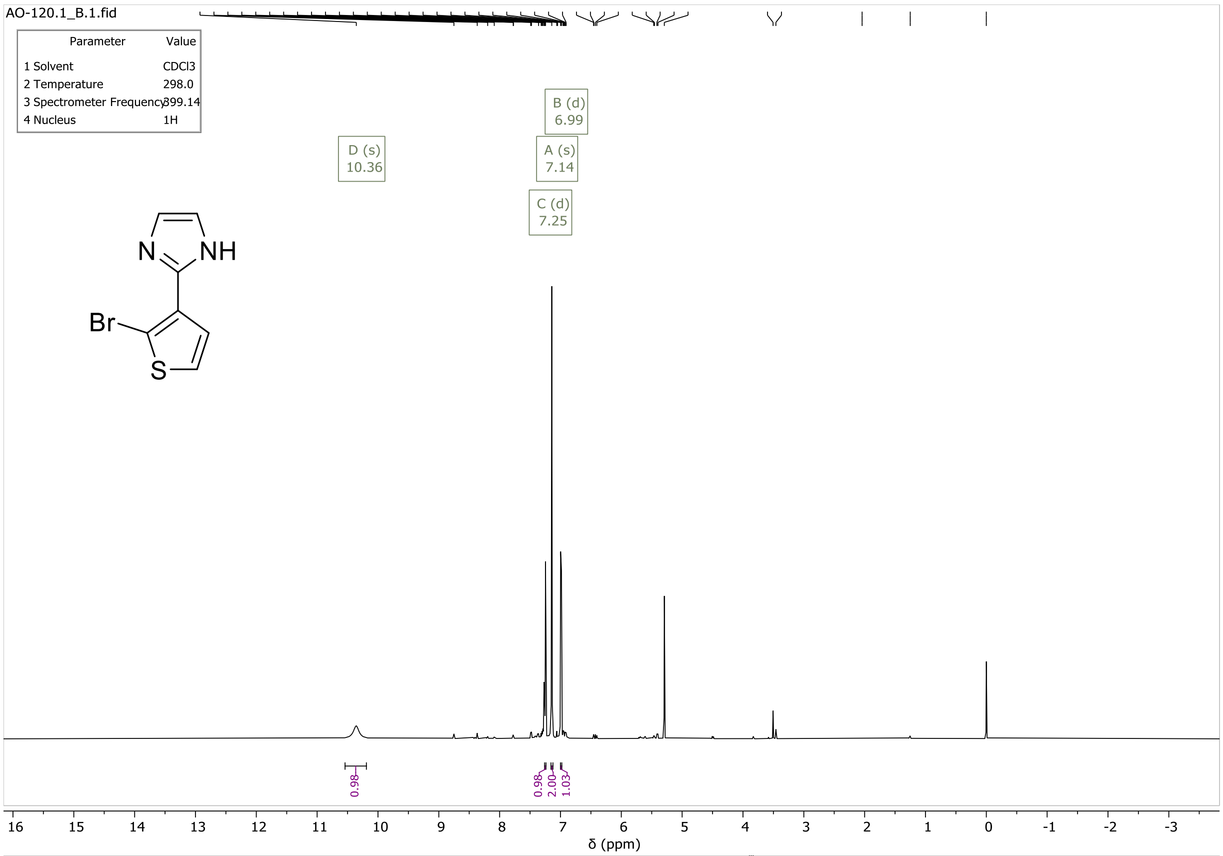

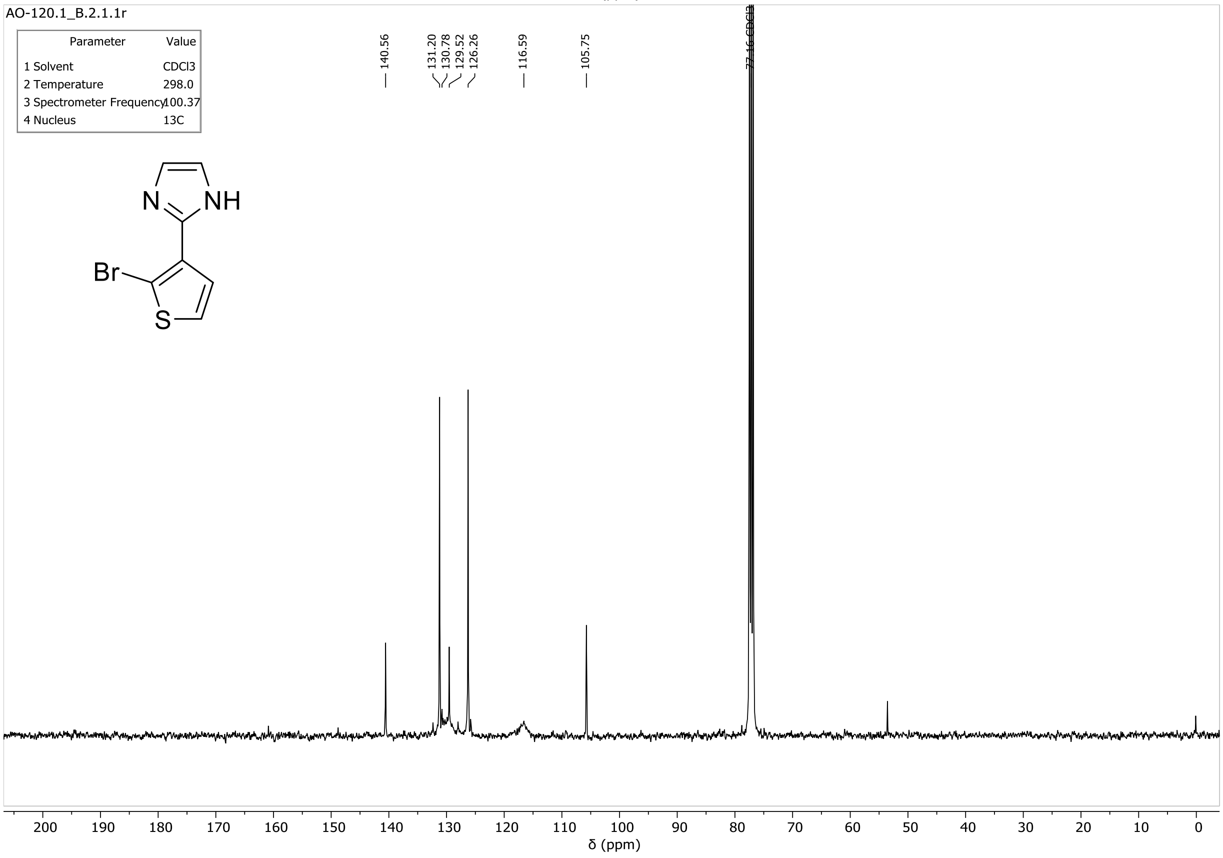

SI Figure S13: ^{1}H- and ^{13}C{^{1}H}-NMR spectra of imidazole **59**.

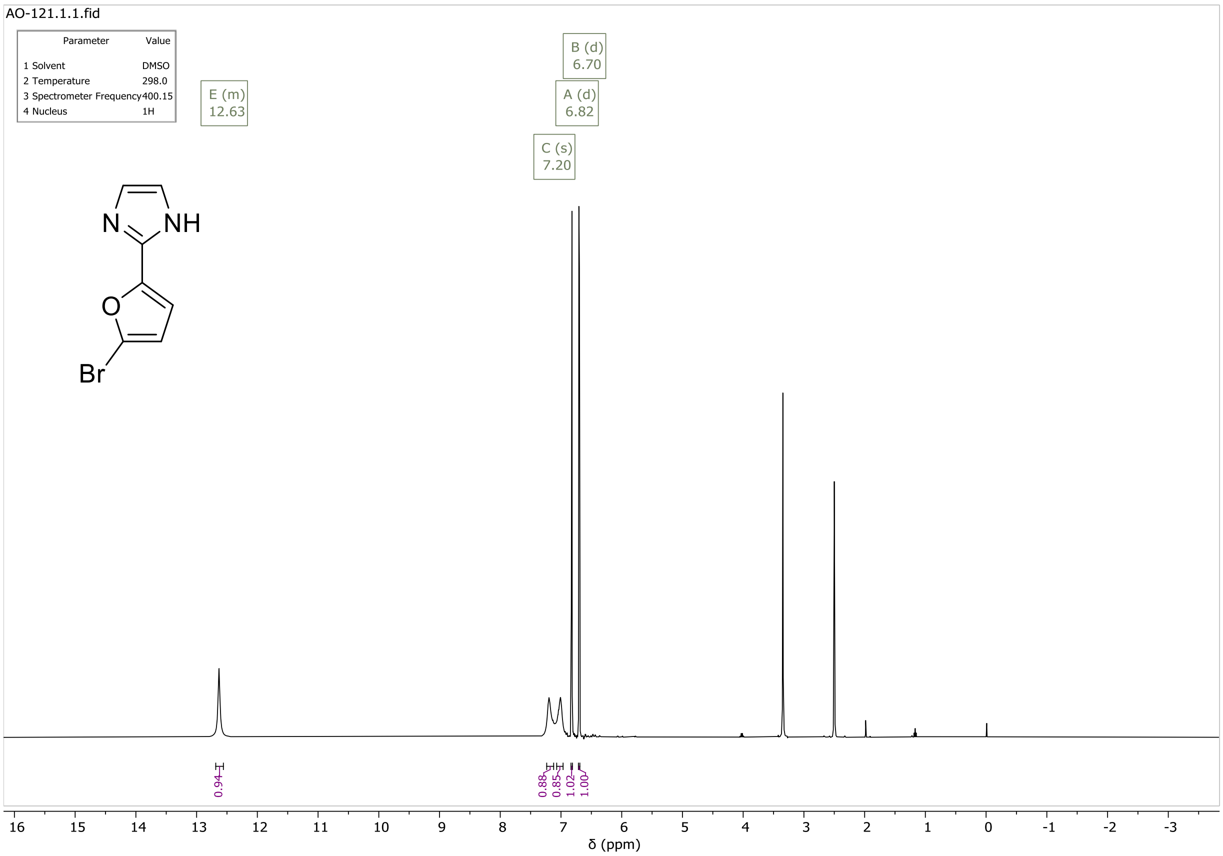

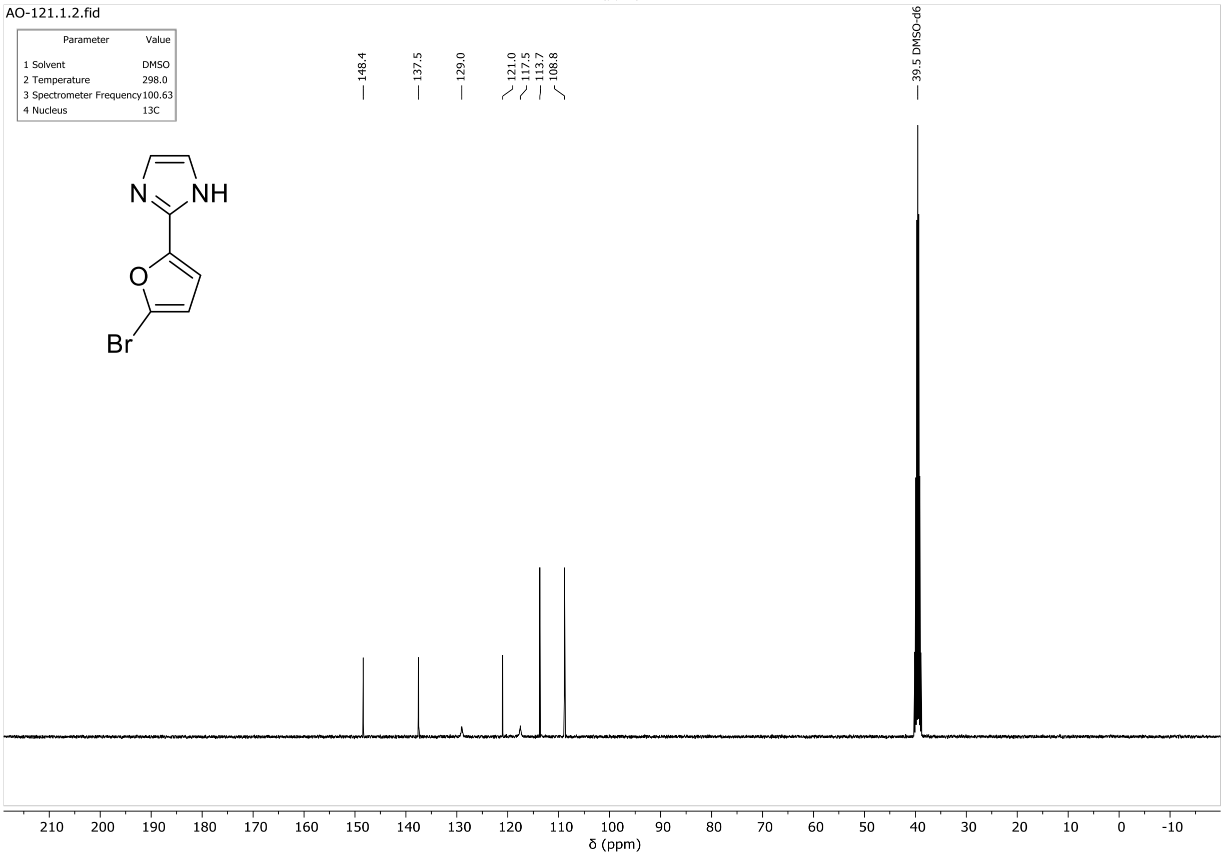

SI Figure 14: ^{1}H- and $^{13}C\{^{1}H\}$-NMR spectra of imidazole **60**.

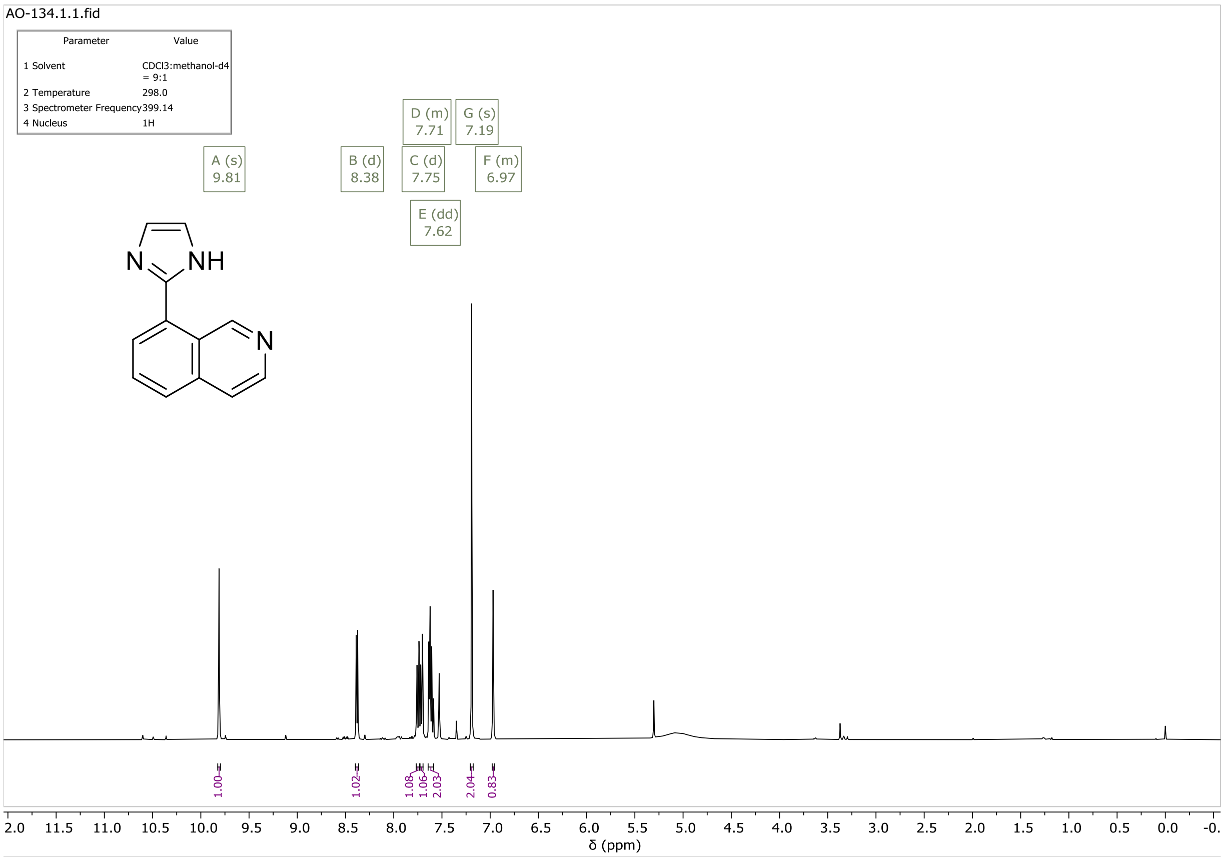

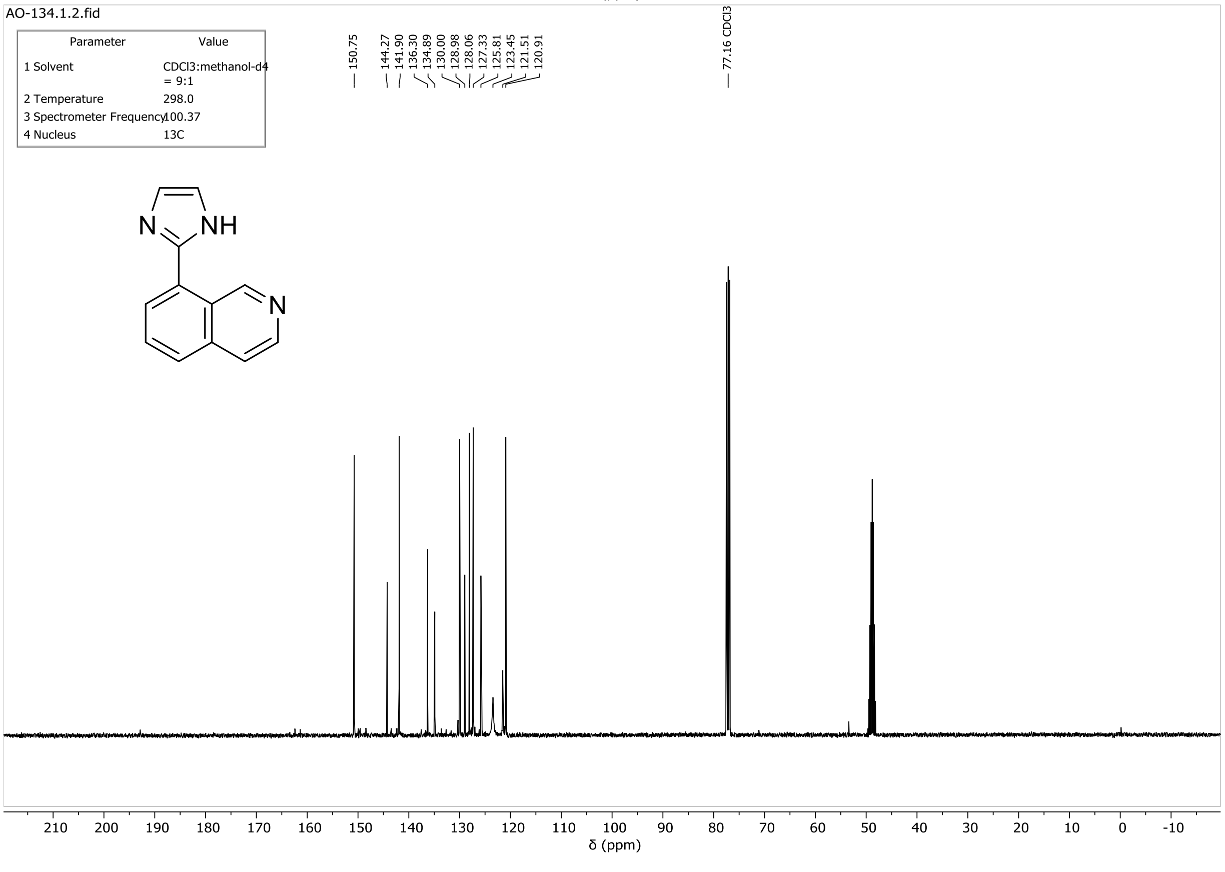

SI Figure S15: ^{1}H- and ^{13}C{^{1}H}-NMR spectra of imidazole **61**.

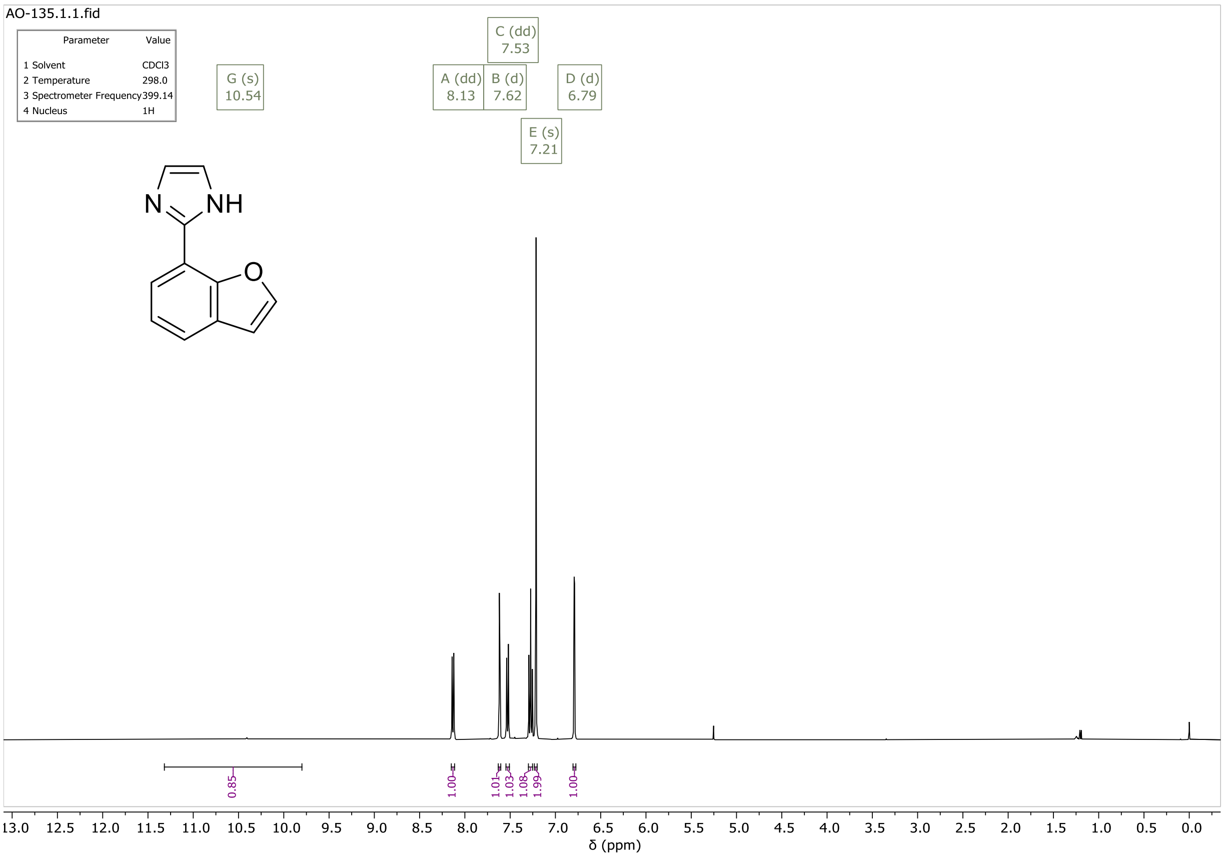

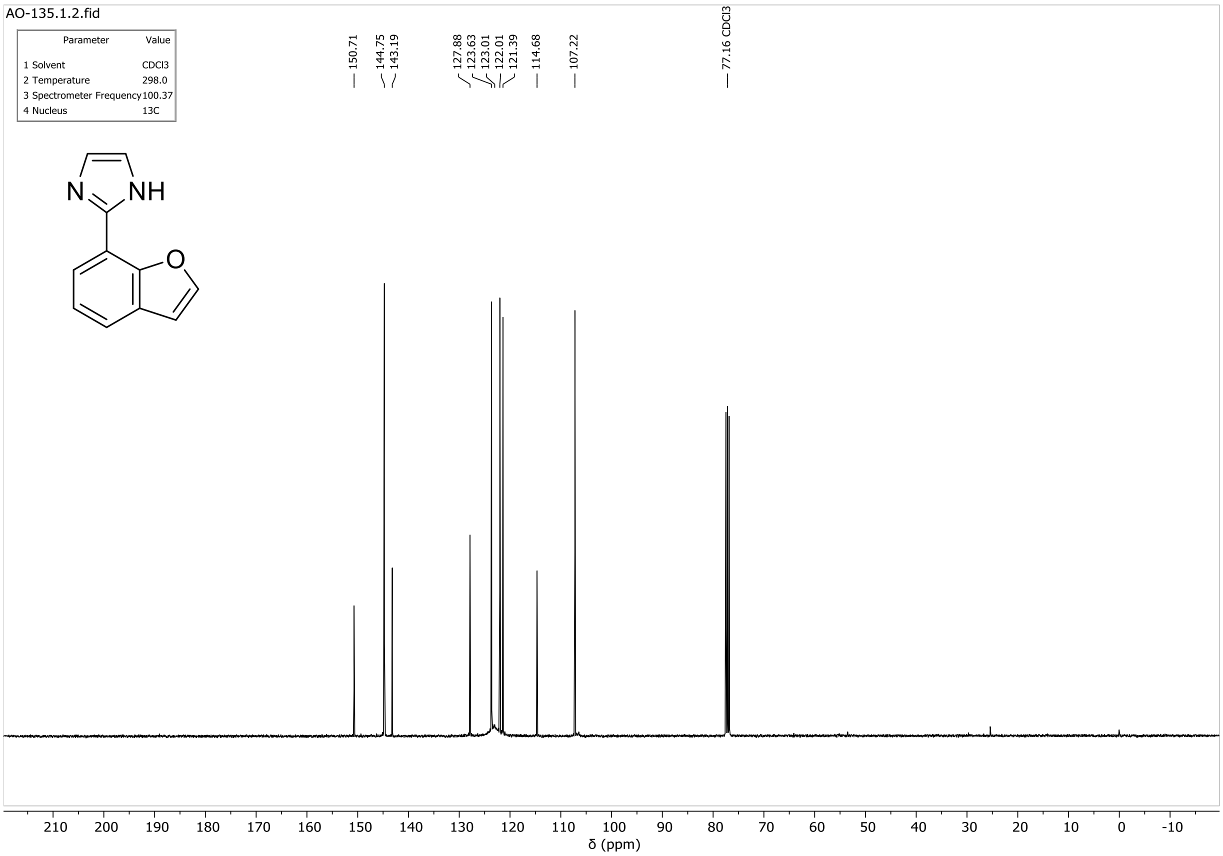

SI Figure S16: ^{1}H- and ^{13}C{^{1}H}-NMR spectra of imidazole **62.**

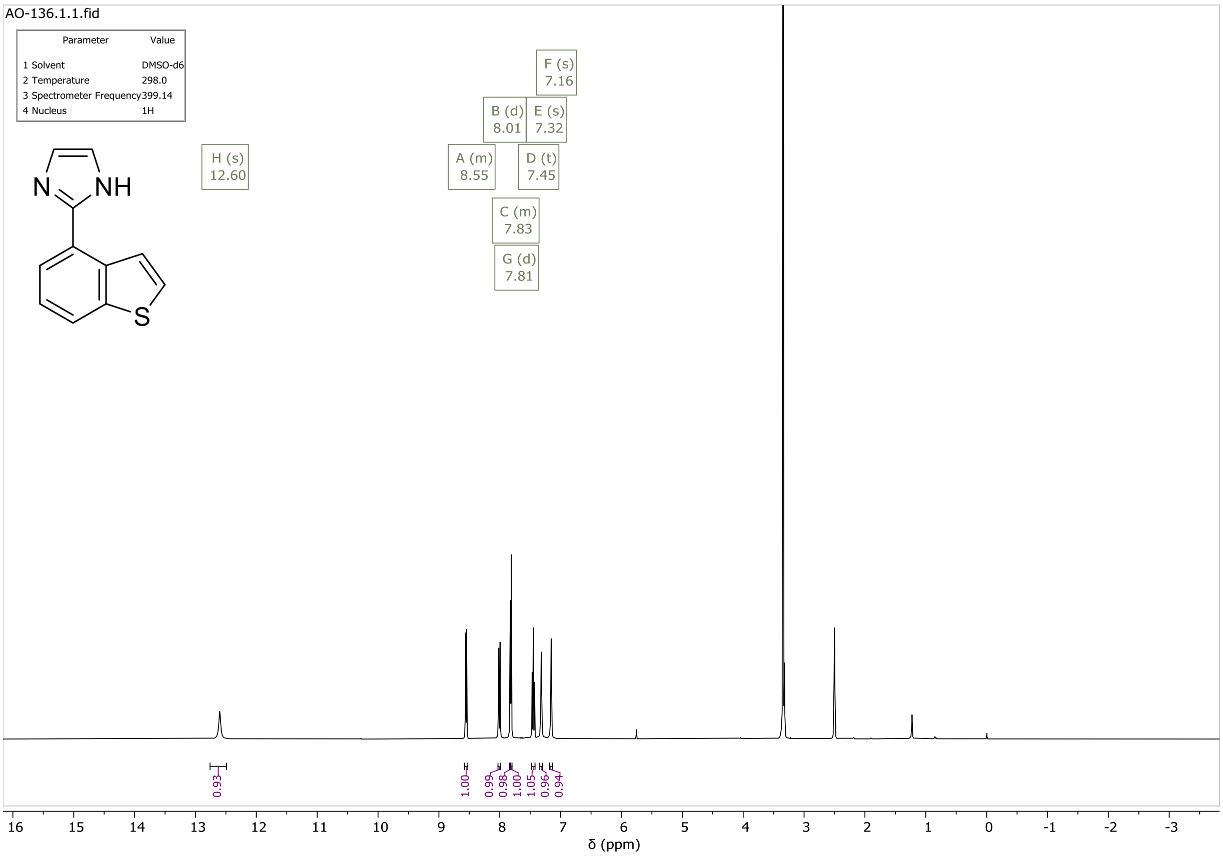

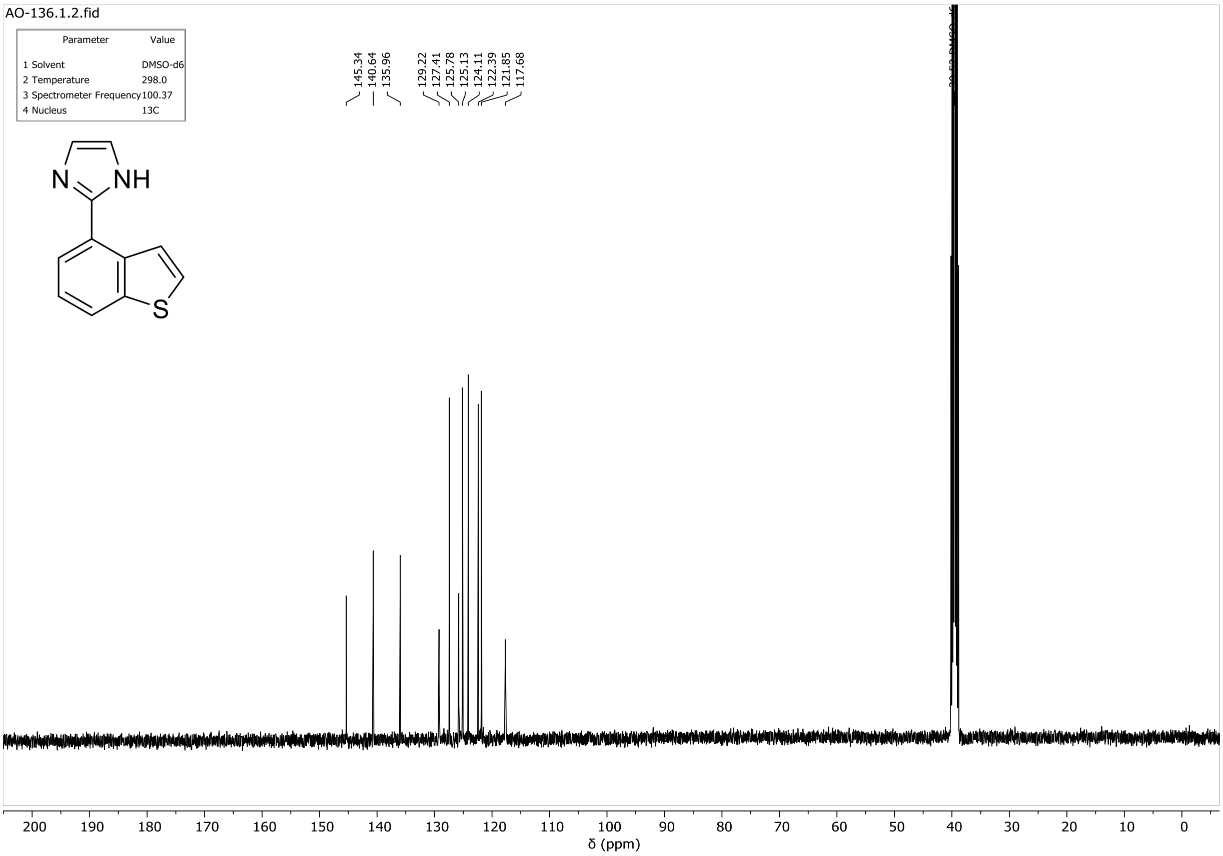

SI Figure S17: ^{1}H- and $^{13}C\{^{1}H\}$-NMR spectra of imidazole **63**.

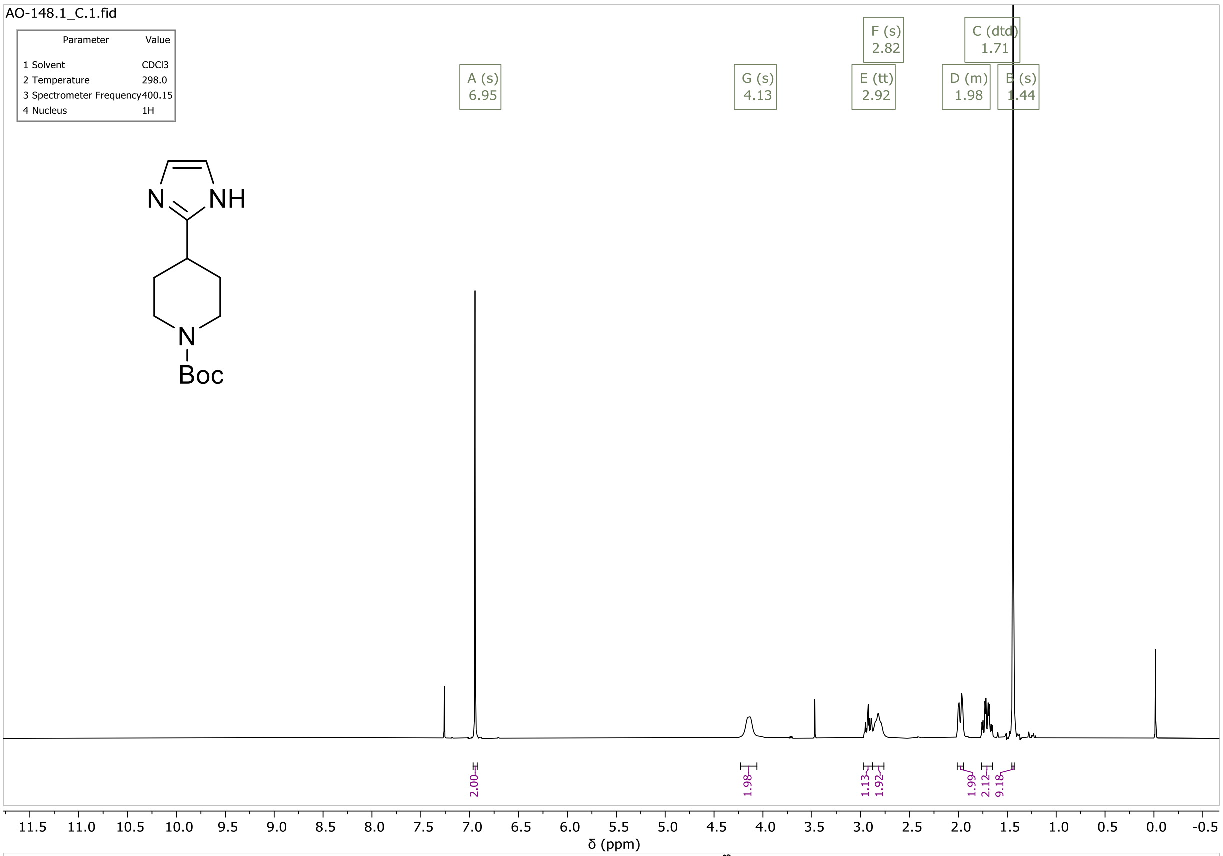

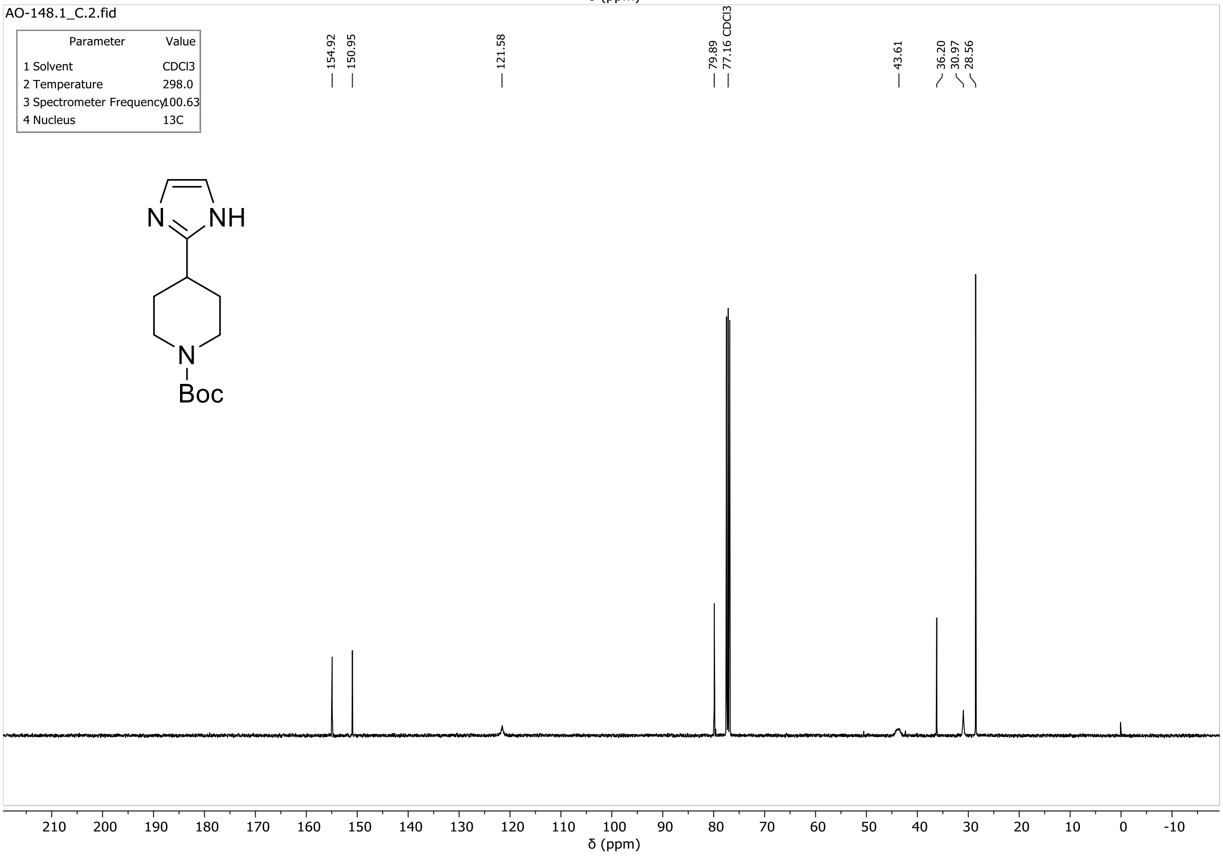

SI Figure S18: ^{1}H- and ^{13}C{^{1}H}-NMR spectra of imidazole **64**.

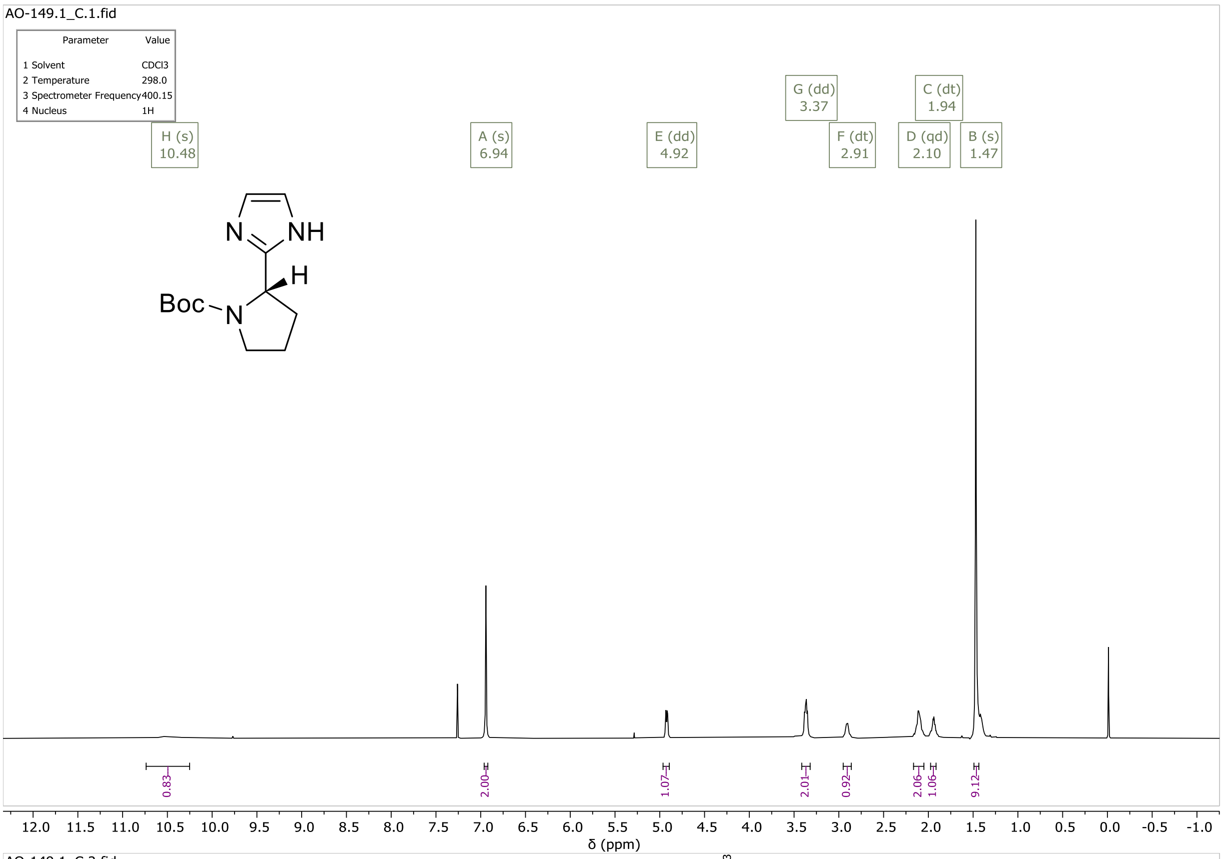

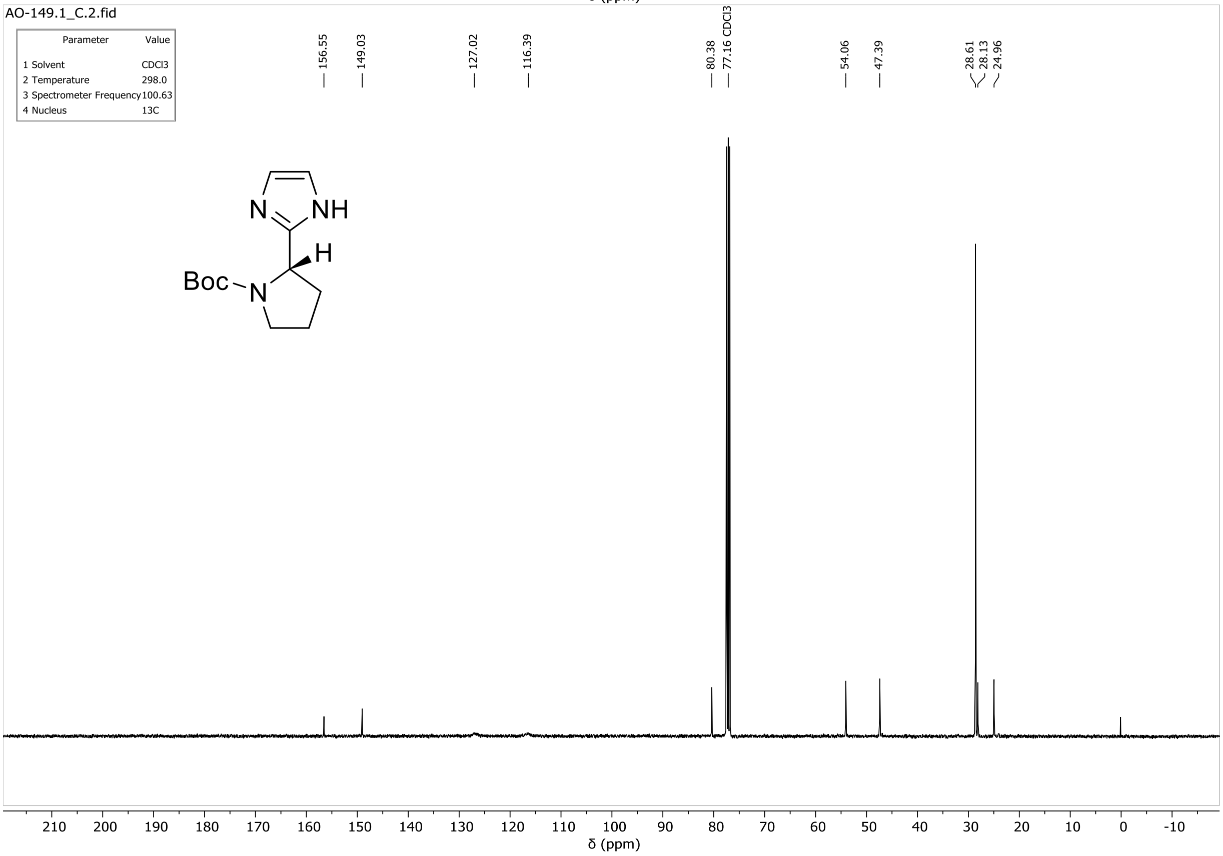

SI Figure S19: ^{1}H- and ^{13}C{^{1}H}-NMR spectra of imidazole **65.**

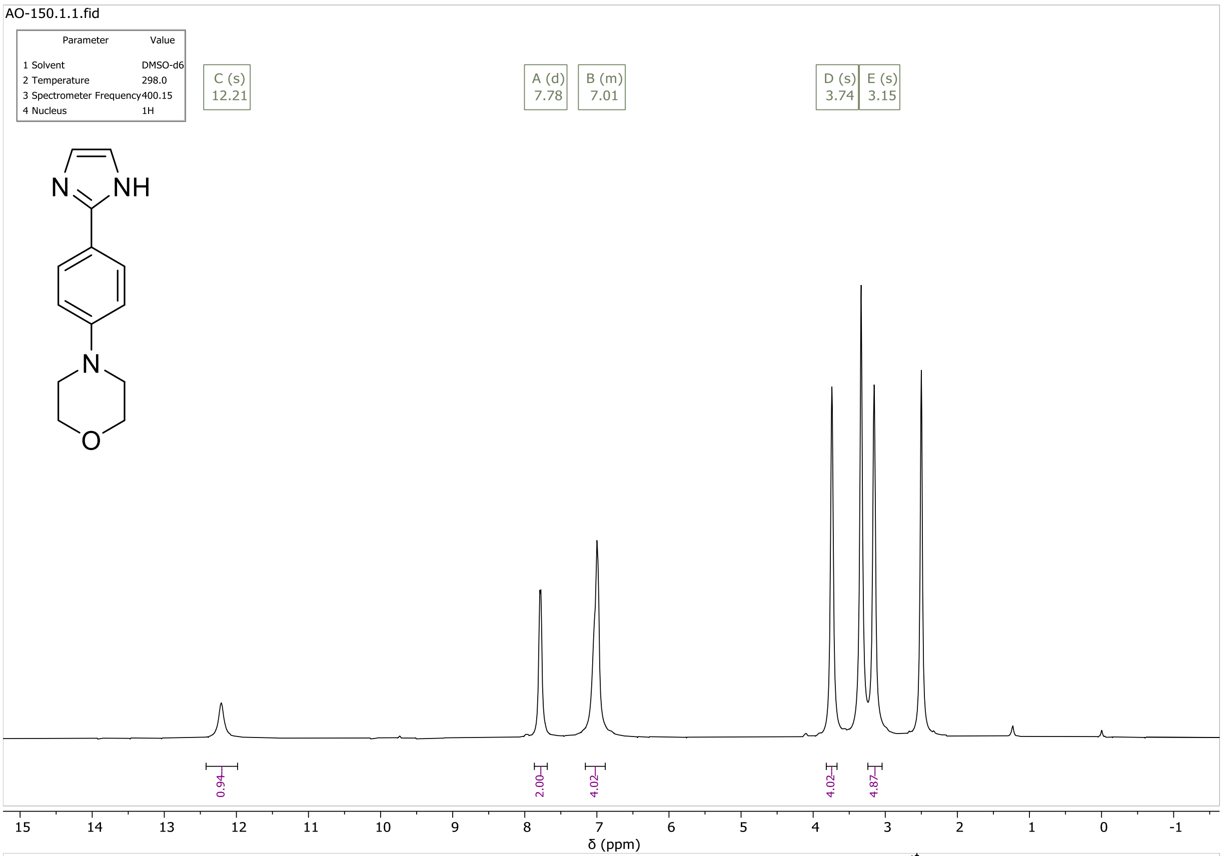

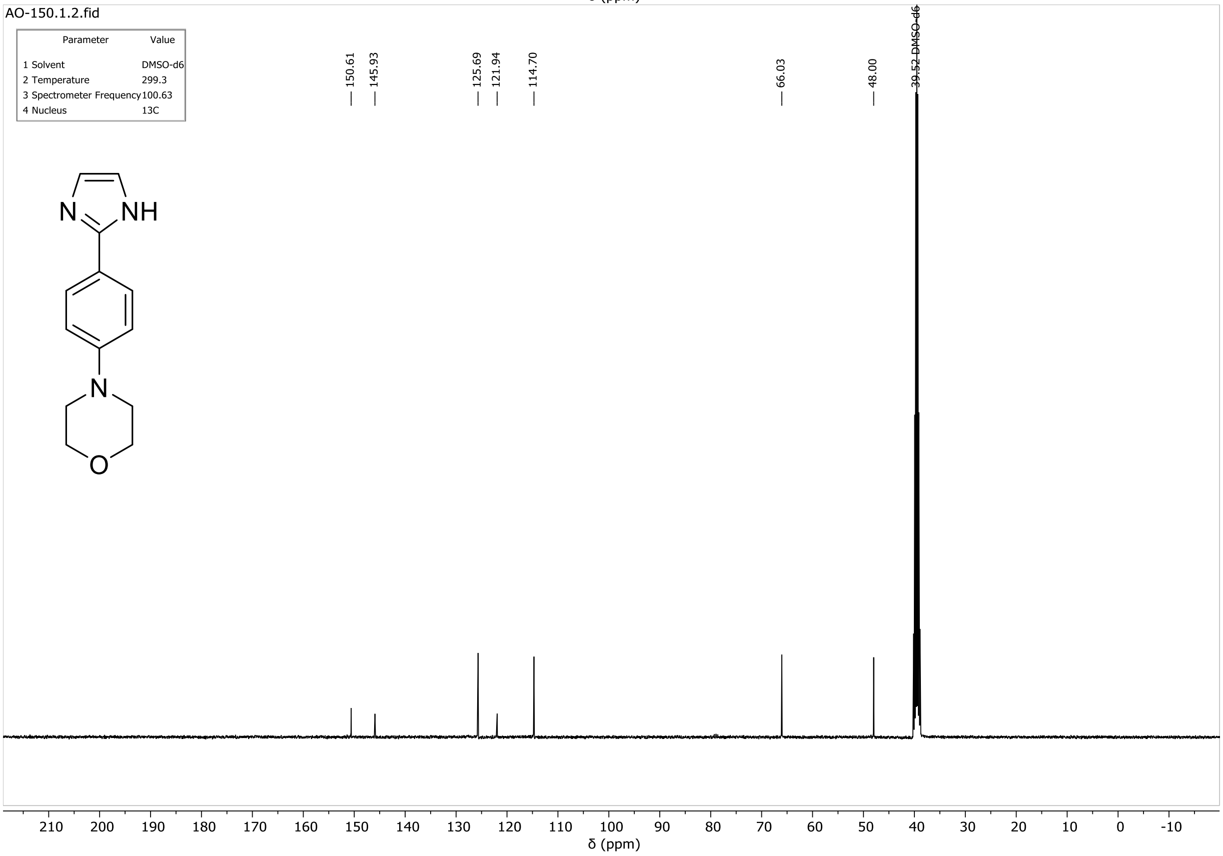

SI Figure S20: ^{1}H- and ^{13}C{^{1}H}-NMR spectra of imidazole **66.**

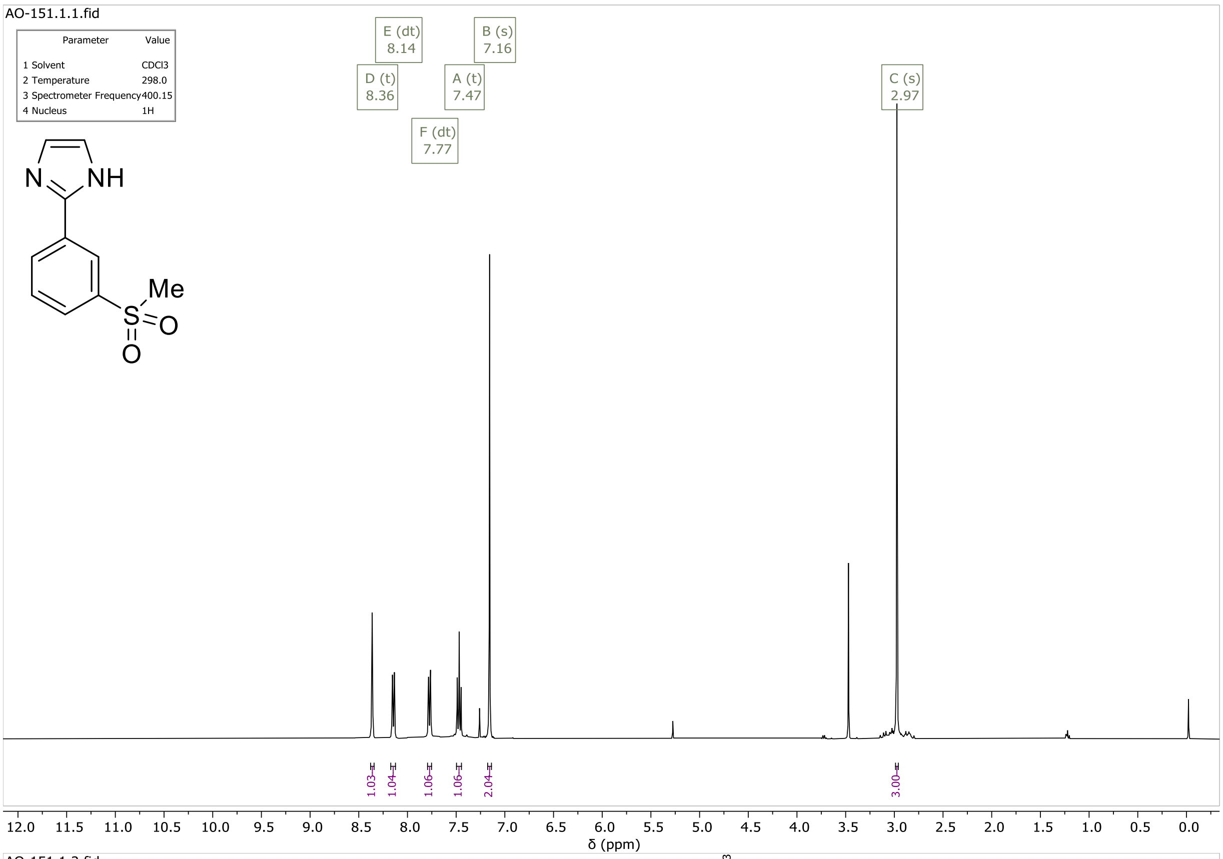

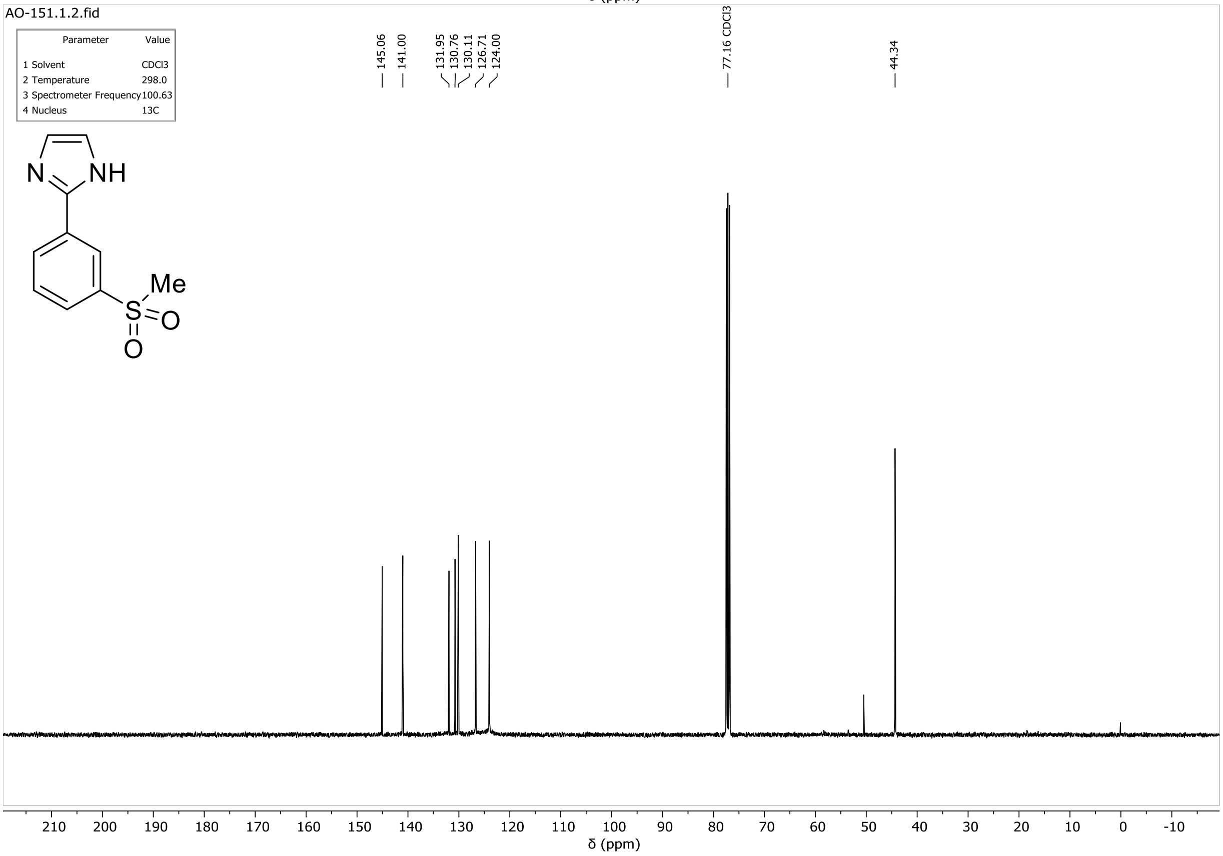

SI Figure S21: ^{1}H- and ^{13}C{^{1}H}-NMR spectra of imidazole **67**.

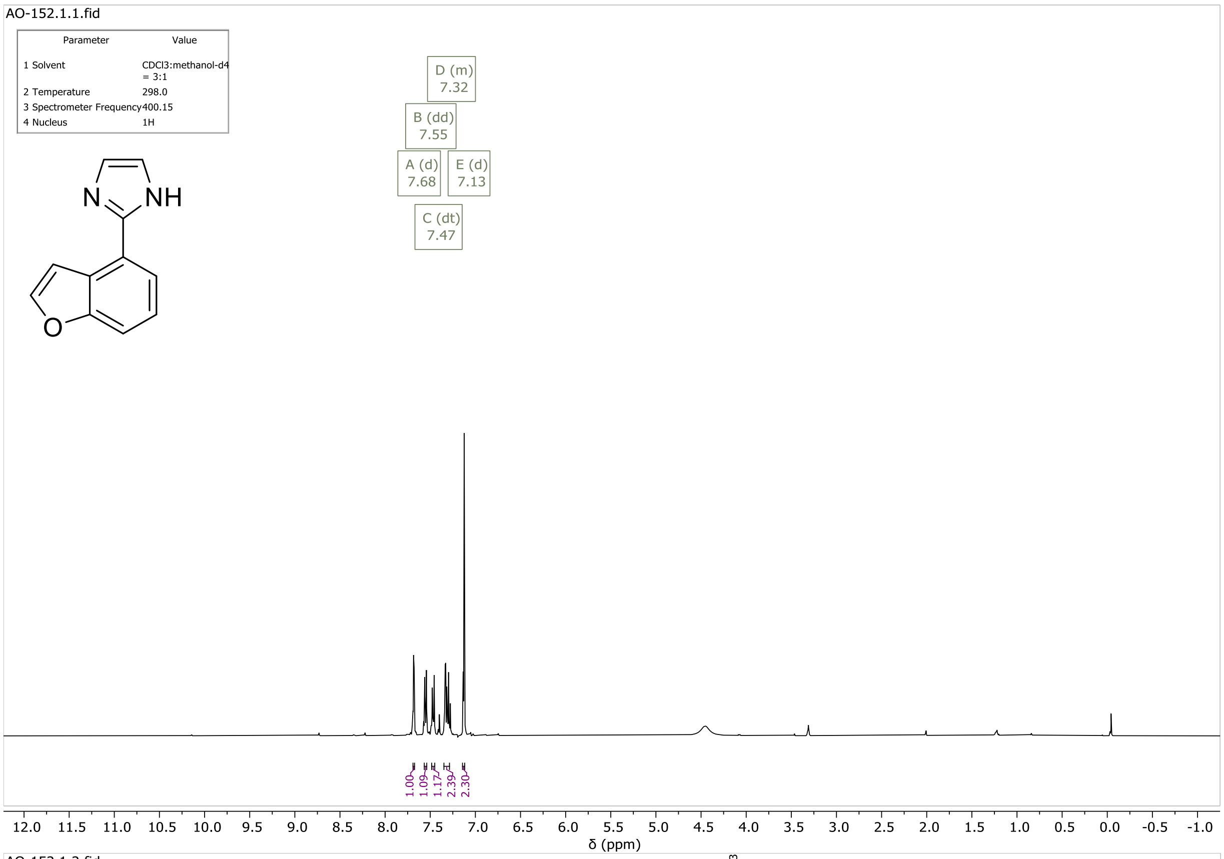

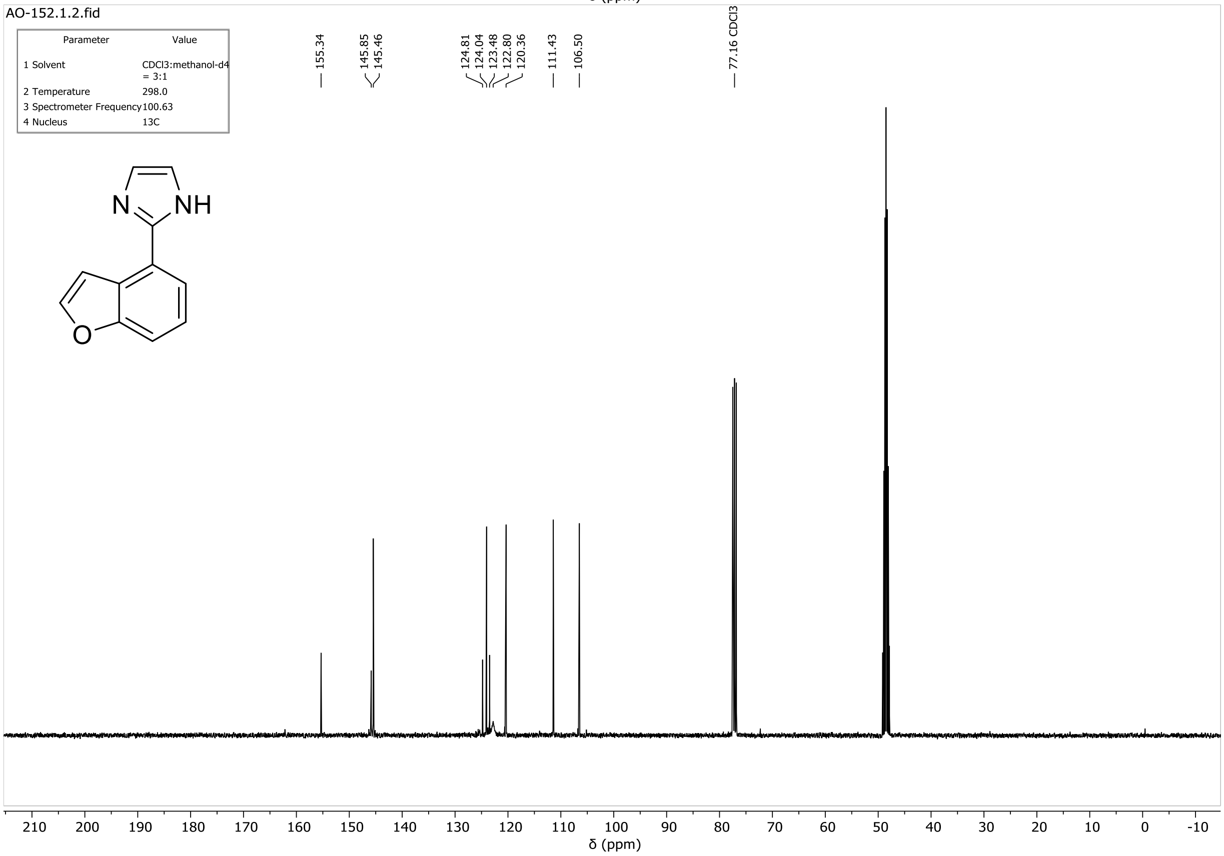

SI Figure S22: ^{1}H- and ^{13}C{^{1}H}-NMR spectra of imidazole **68.**

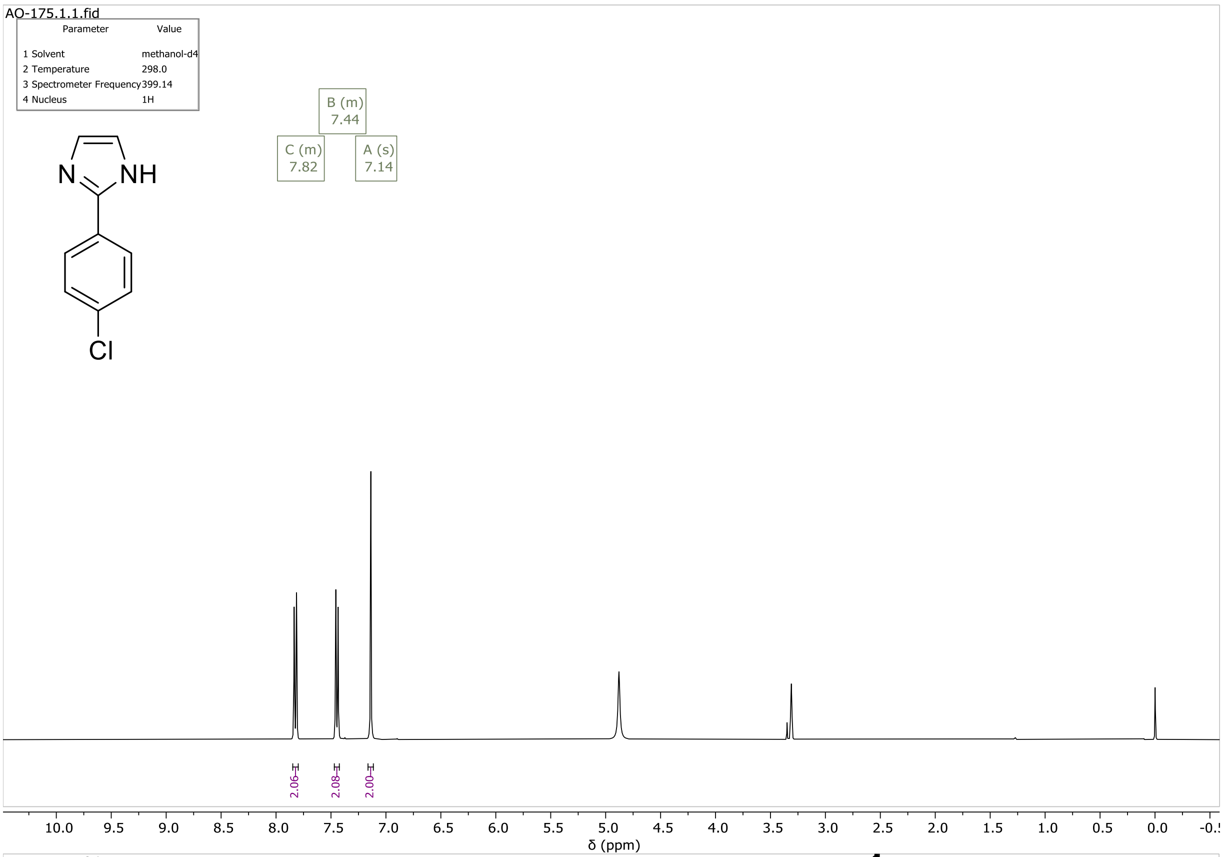

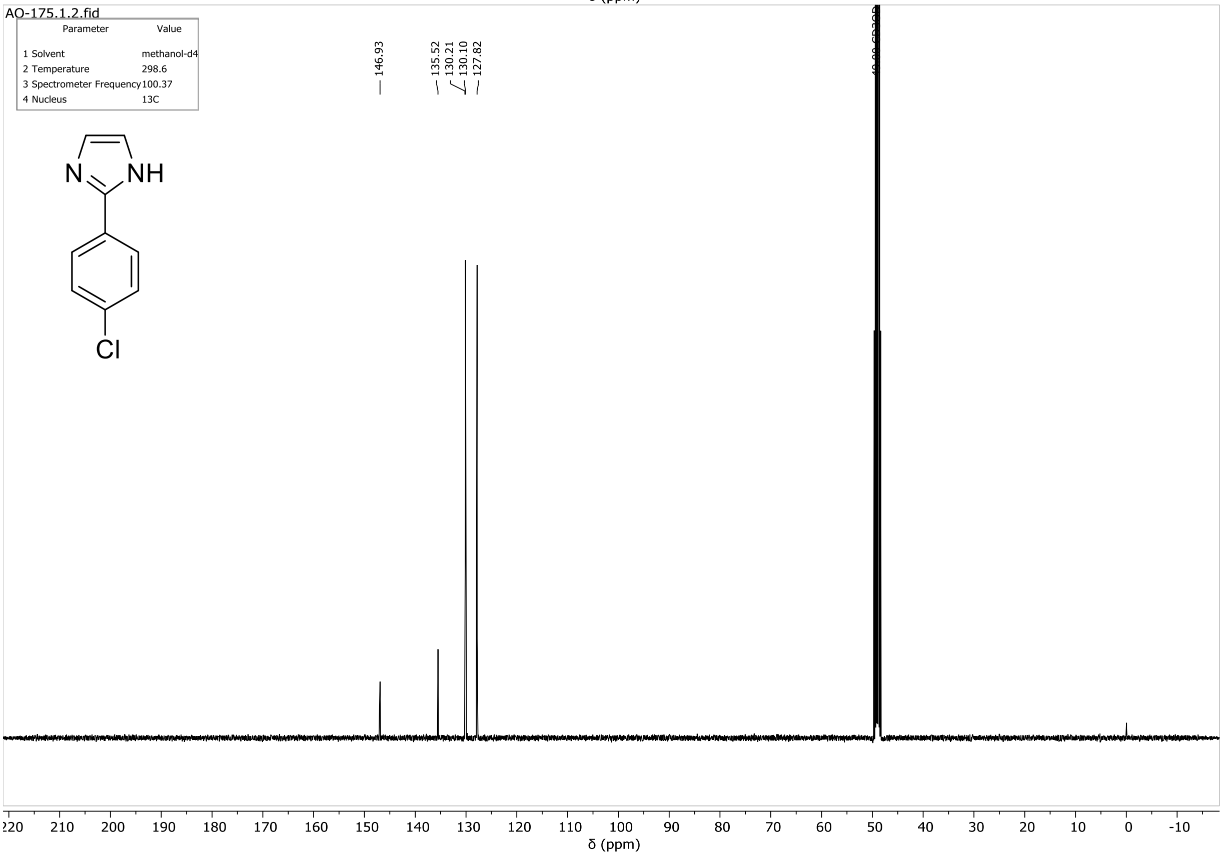

SI Figure S23: ^{1}H- and ^{13}C{^{1}H}-NMR spectra of imidazole **69**.

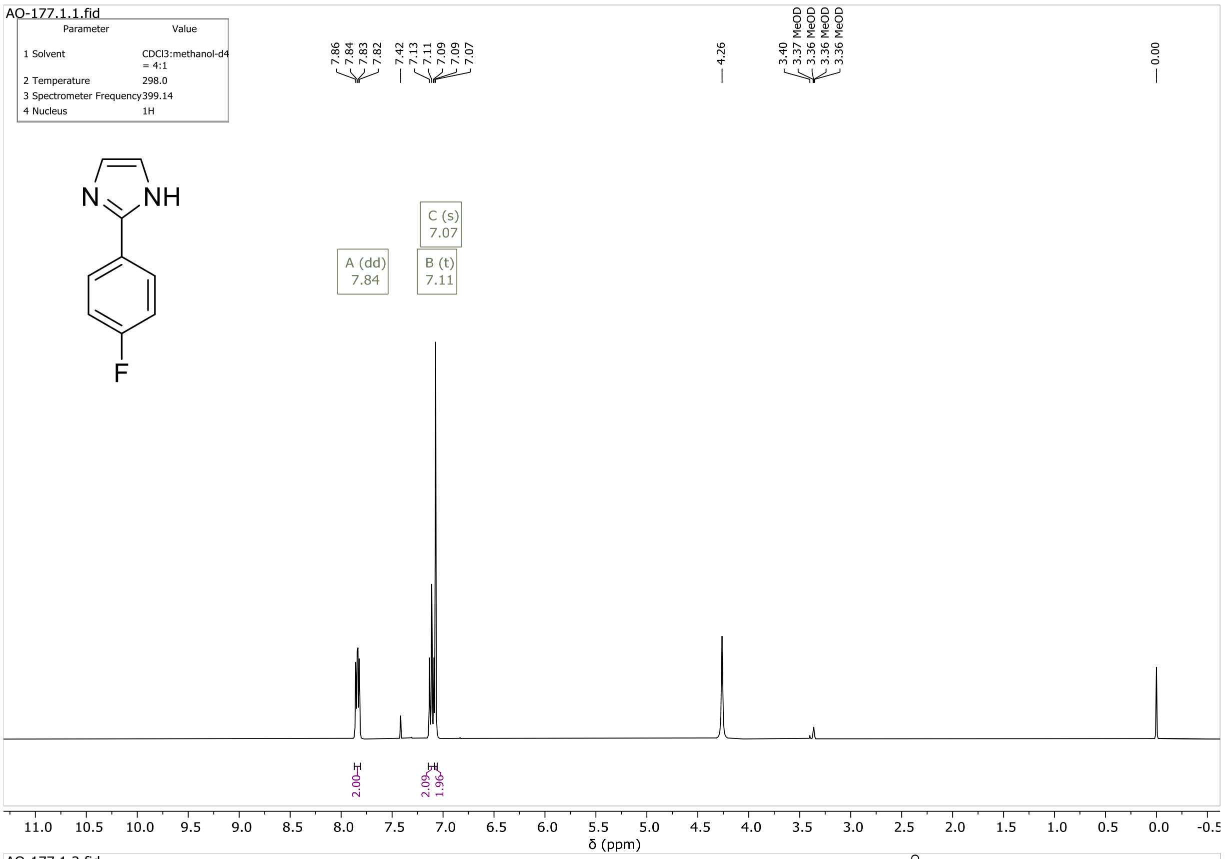

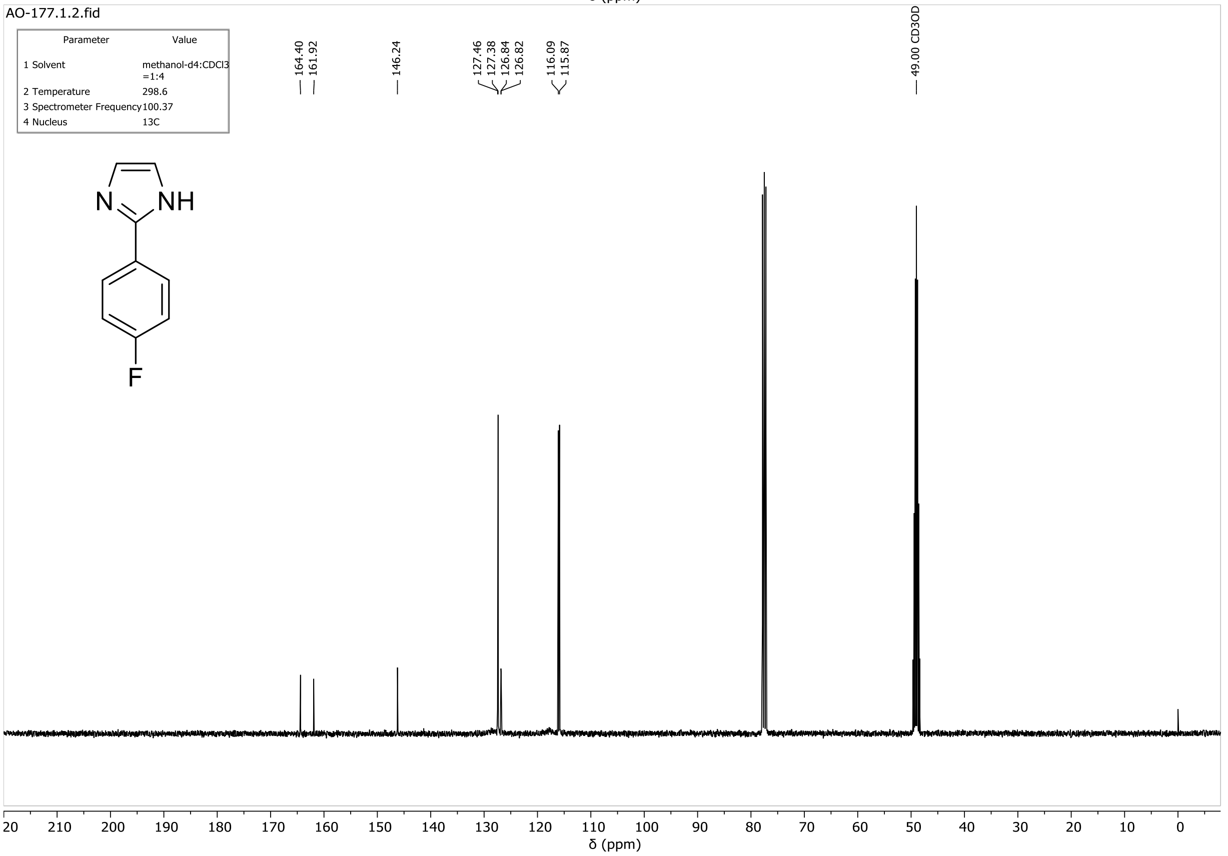

SI Figure S24: ^{1}H- and $^{13}C\{^{1}H\}$-NMR spectra of imidazole **71**.

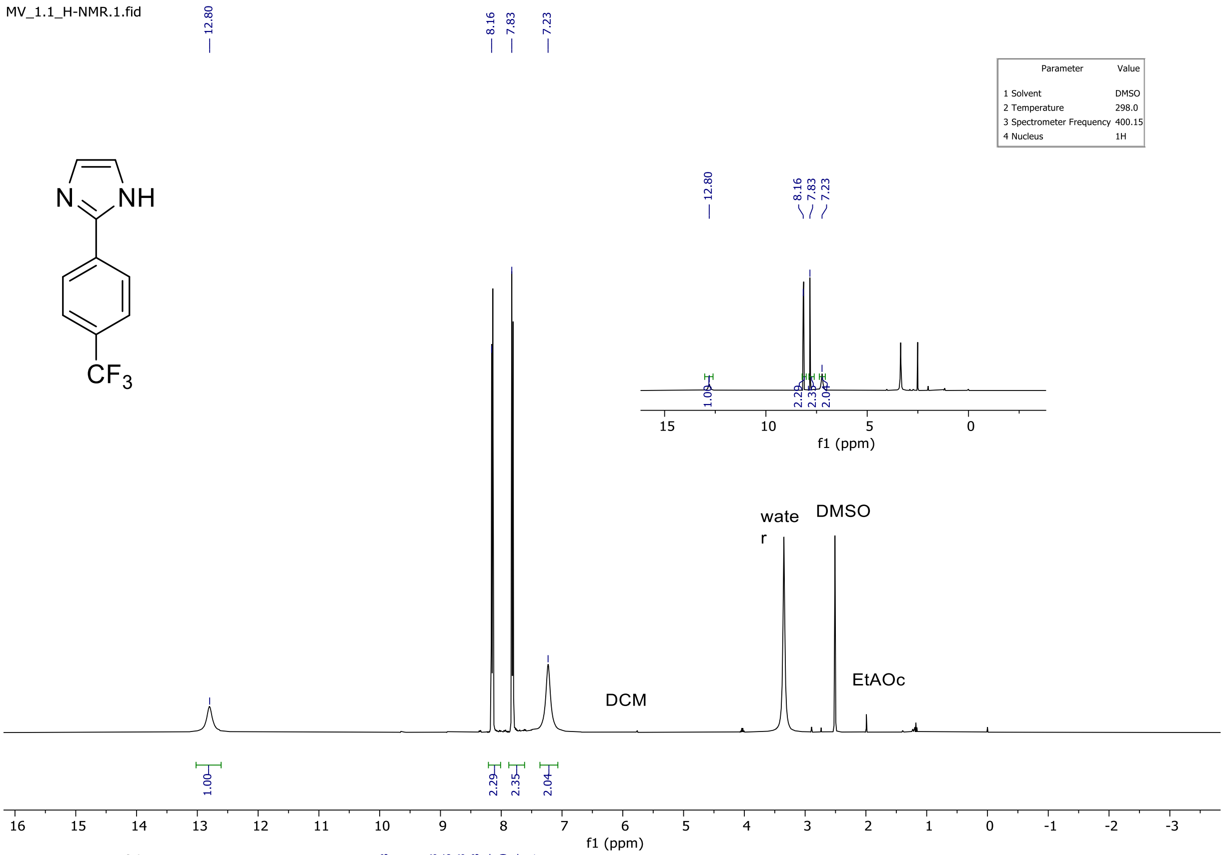

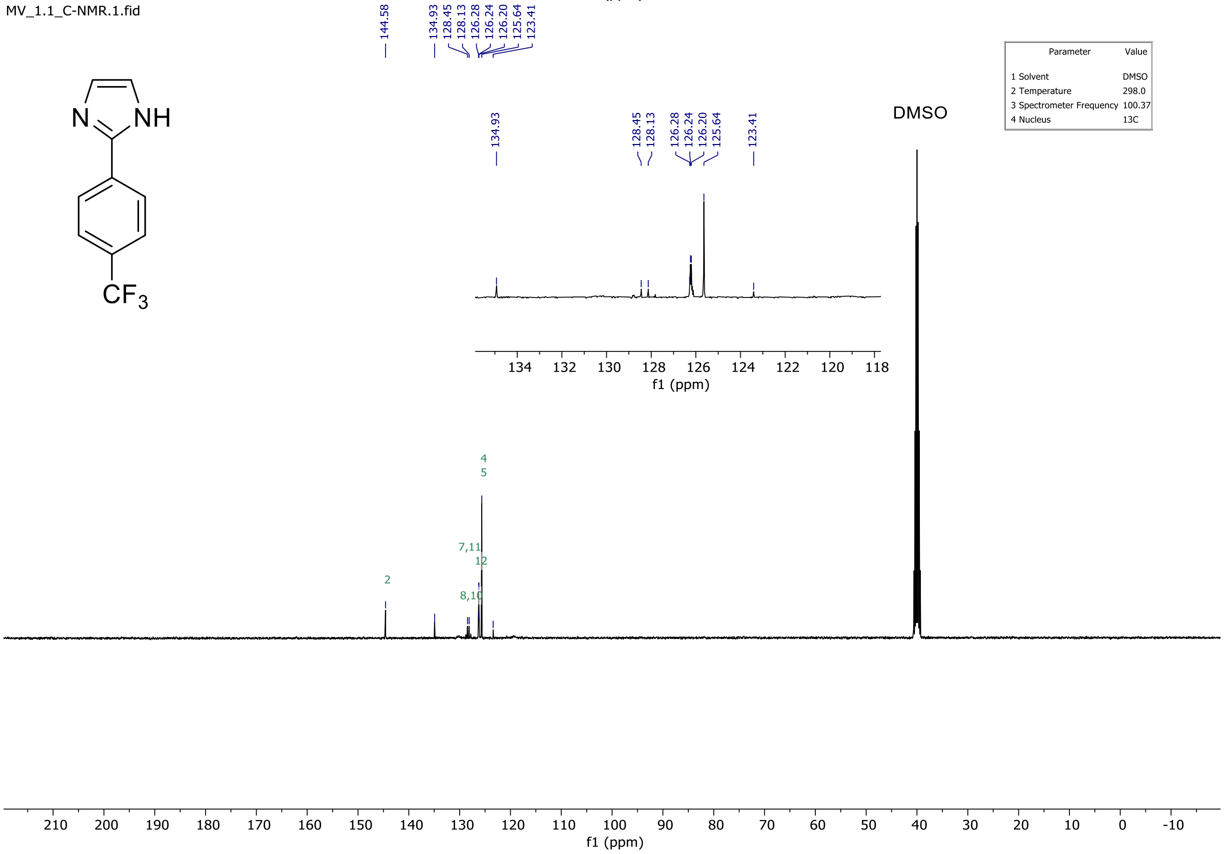

SI Figure S25: ^{1}H- and $^{13}C\{^{1}H\}$-NMR spectra of imidazole **81**.

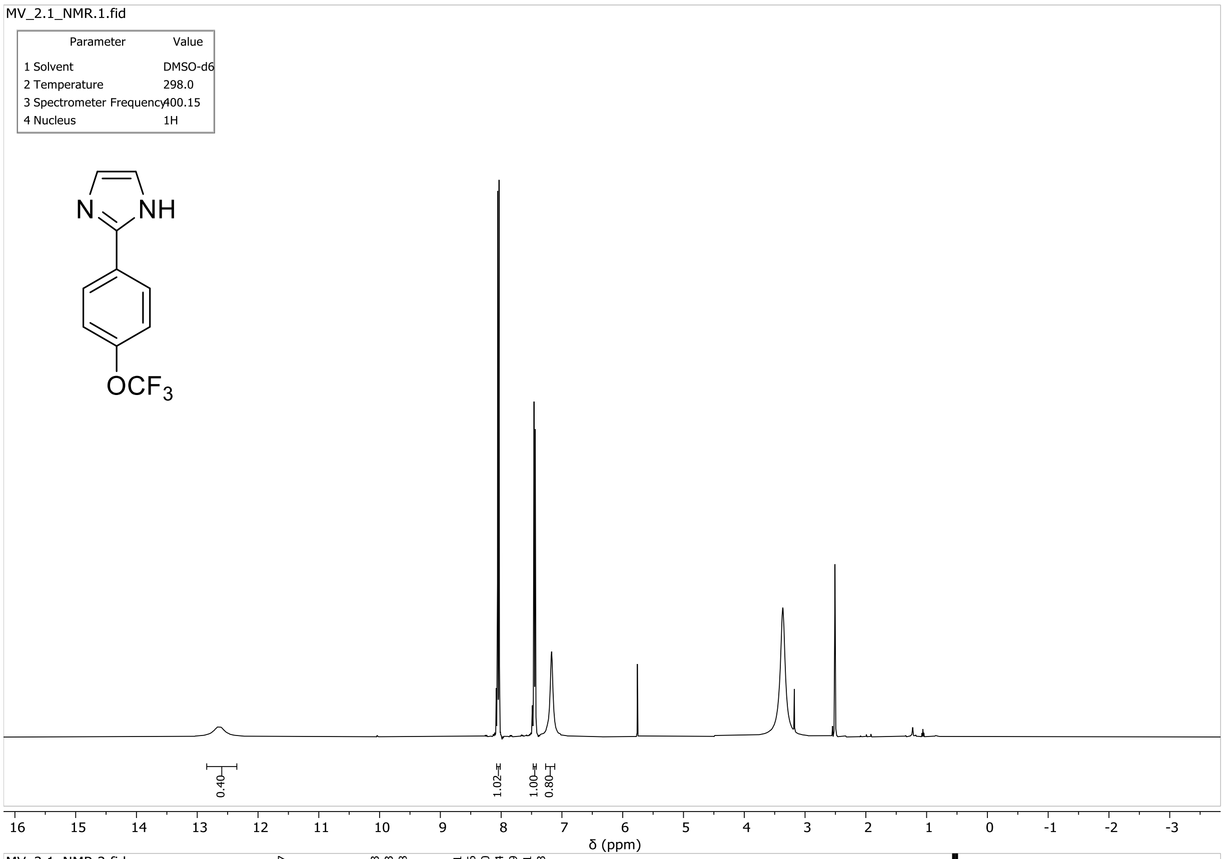

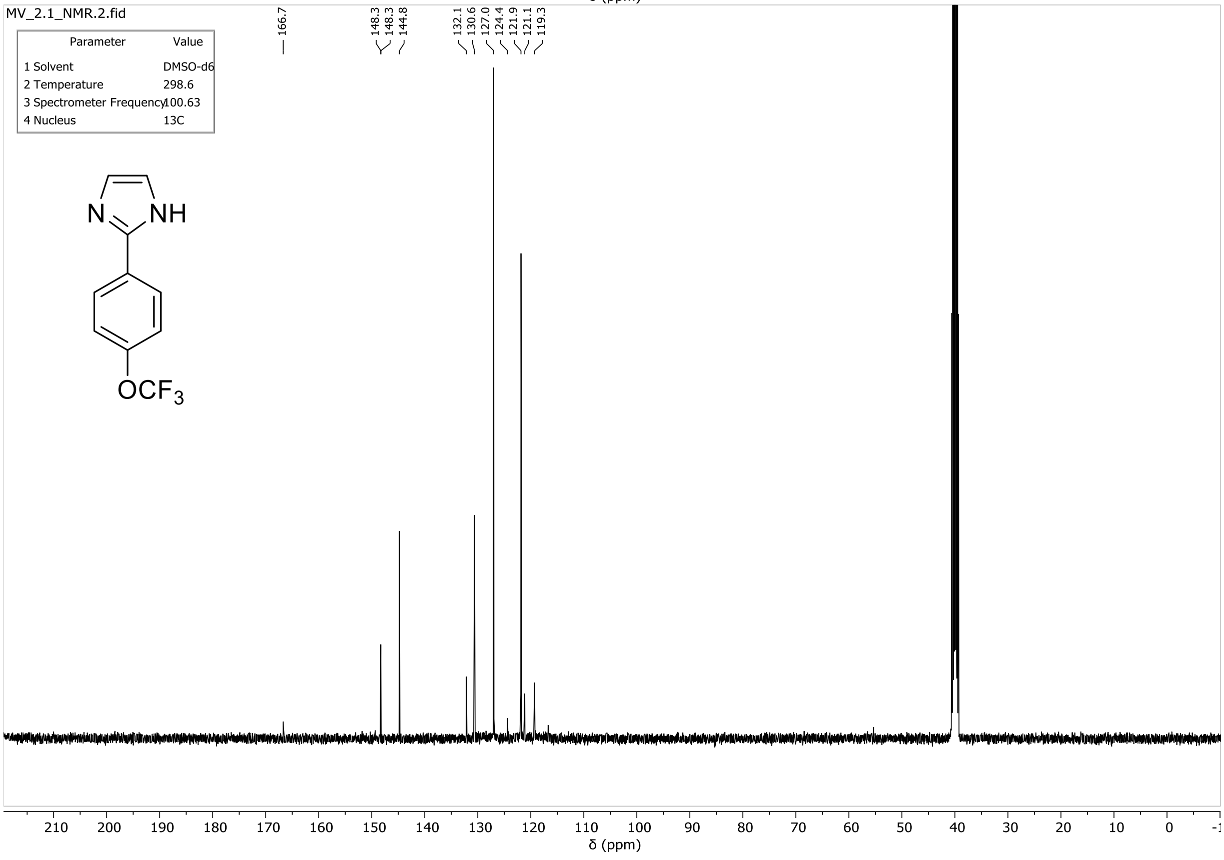

SI Figure S26: ^{1}H- and $^{13}C\{^{1}H\}$-NMR spectra of imidazole **82.**

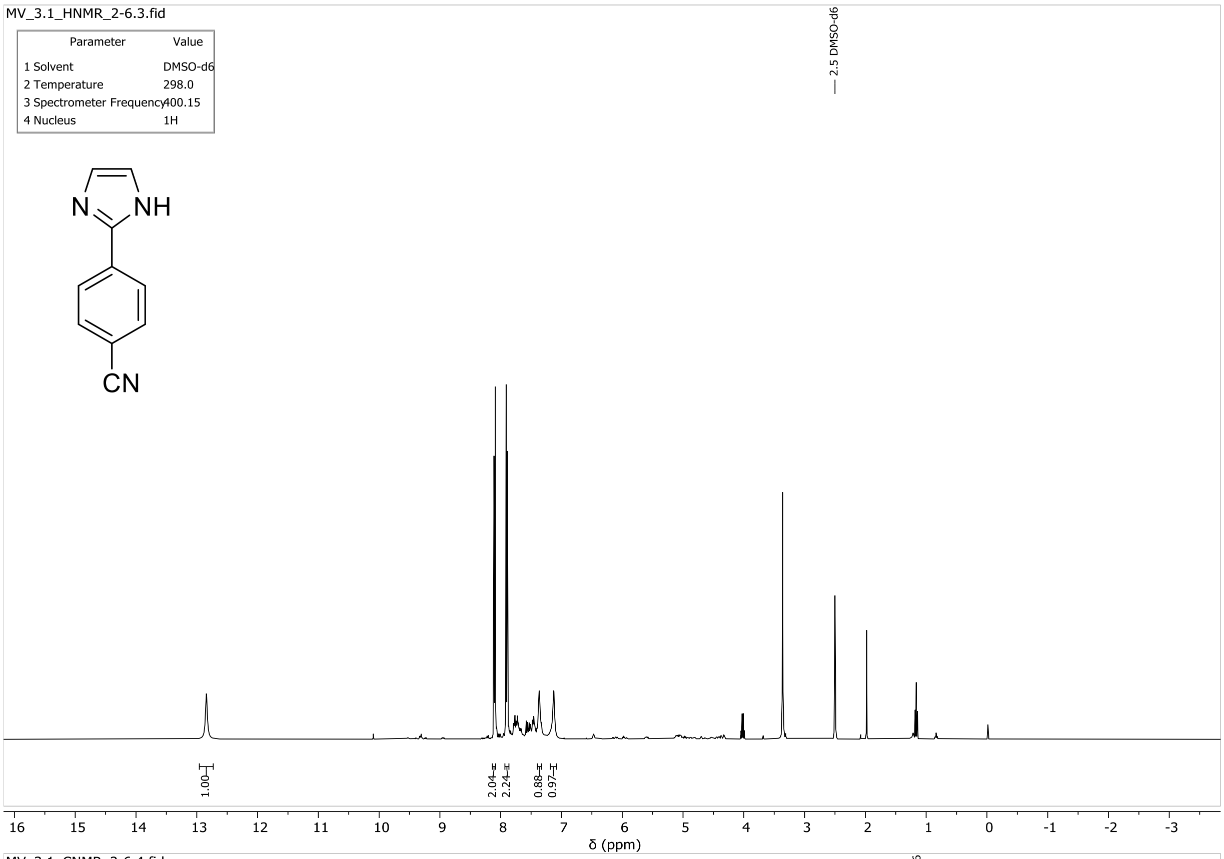

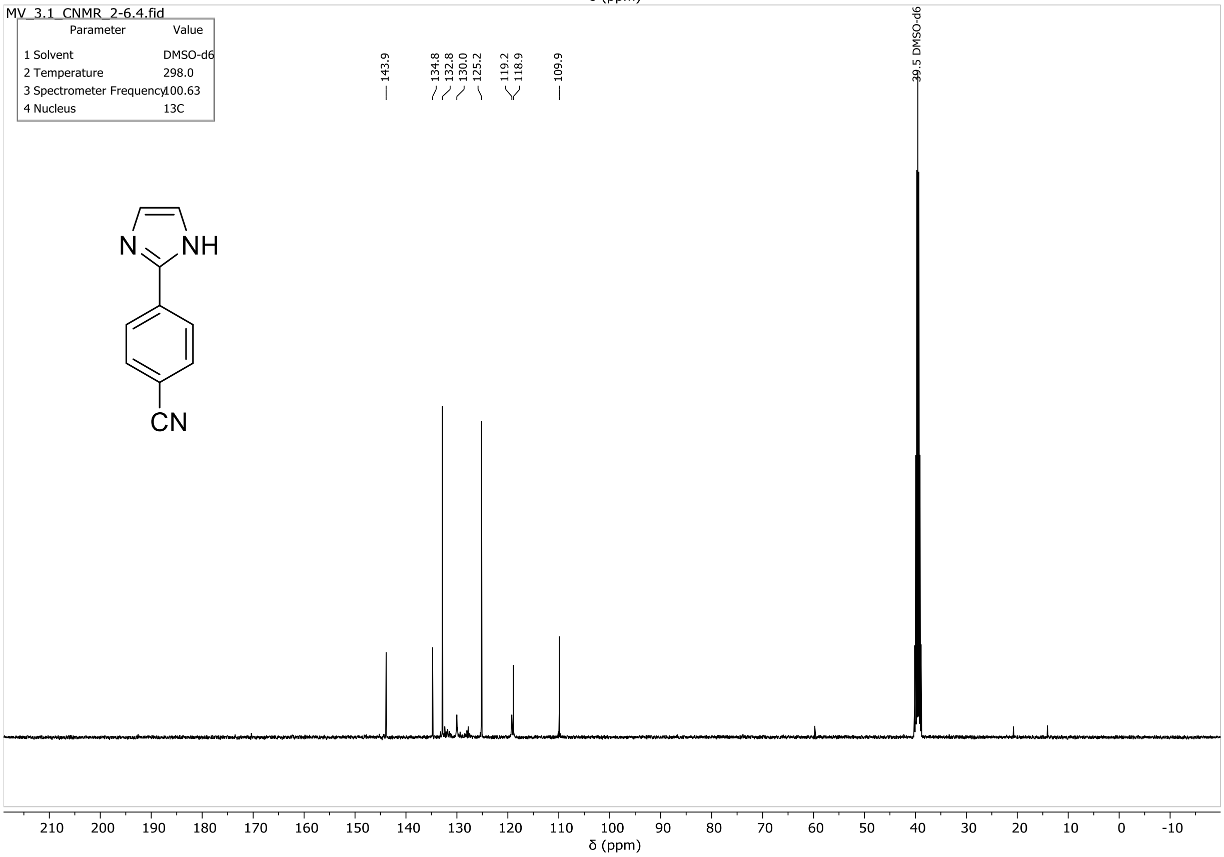

SI Figure S27: ^{1}H- and ^{13}C{^{1}H}-NMR spectra of imidazole **83.**

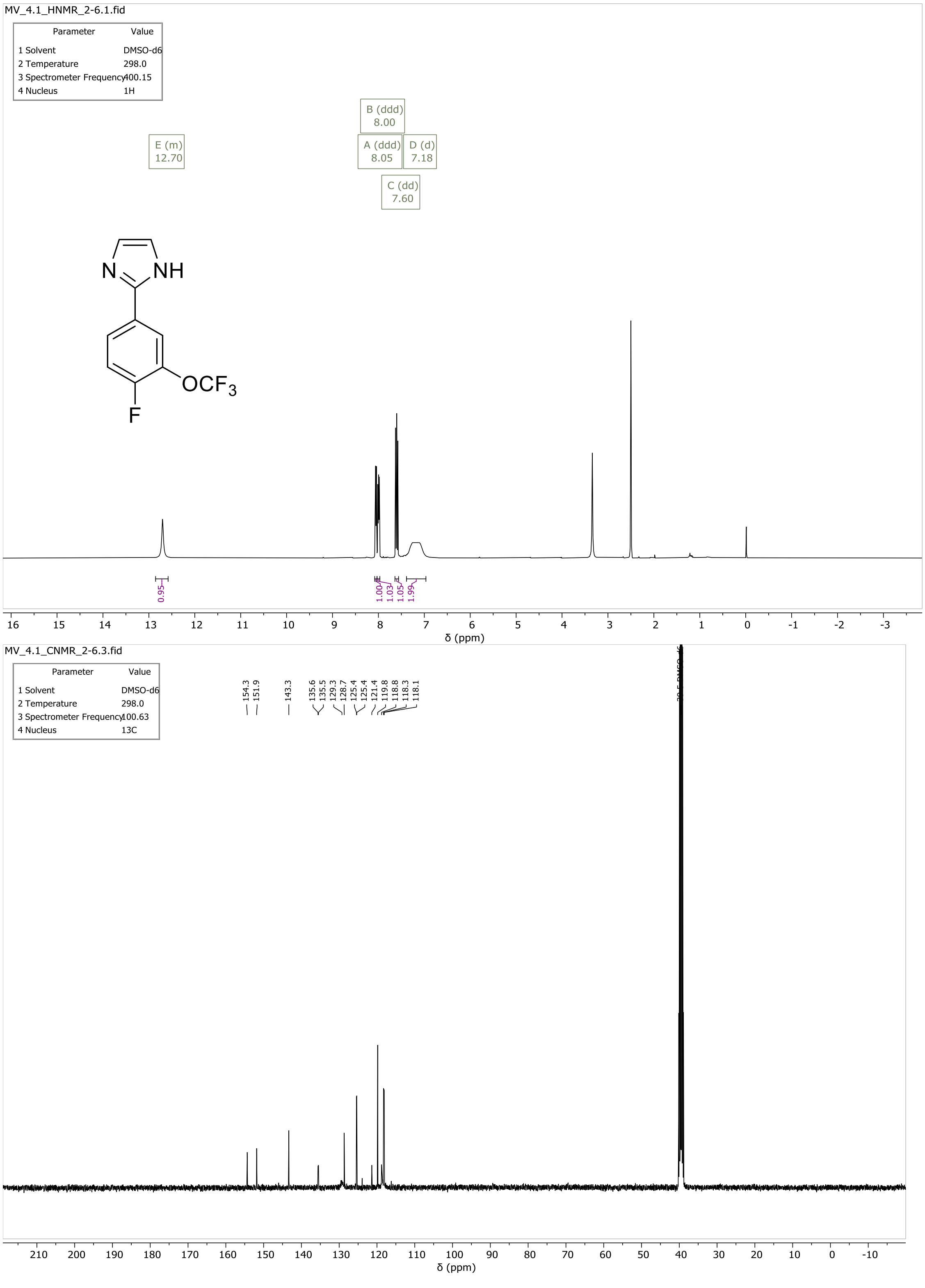

SI Figure S28: ^{1}H- and $^{13}C\{^{1}H\}$-NMR spectra of imidazole **84**.

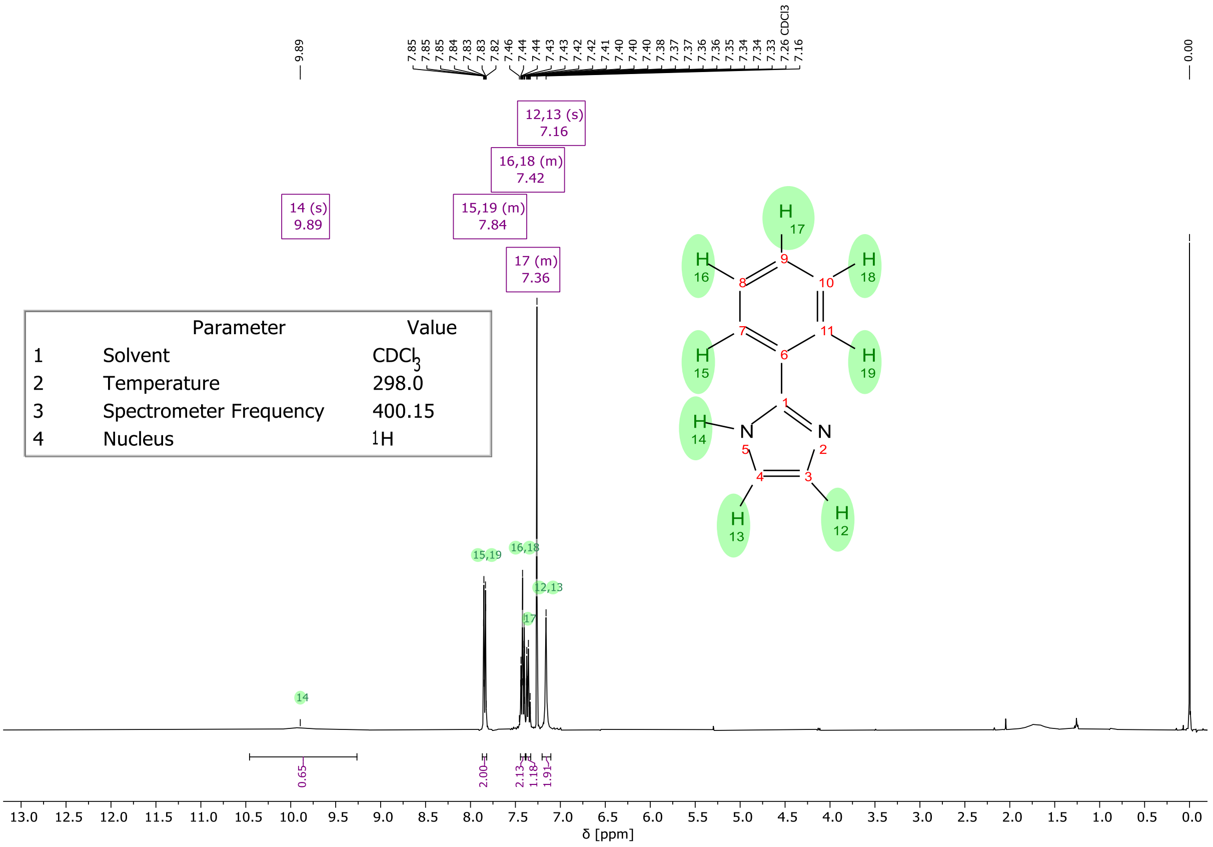

SI Figure S29: ^{1}H-NMR spectrum of imidazole **47**.

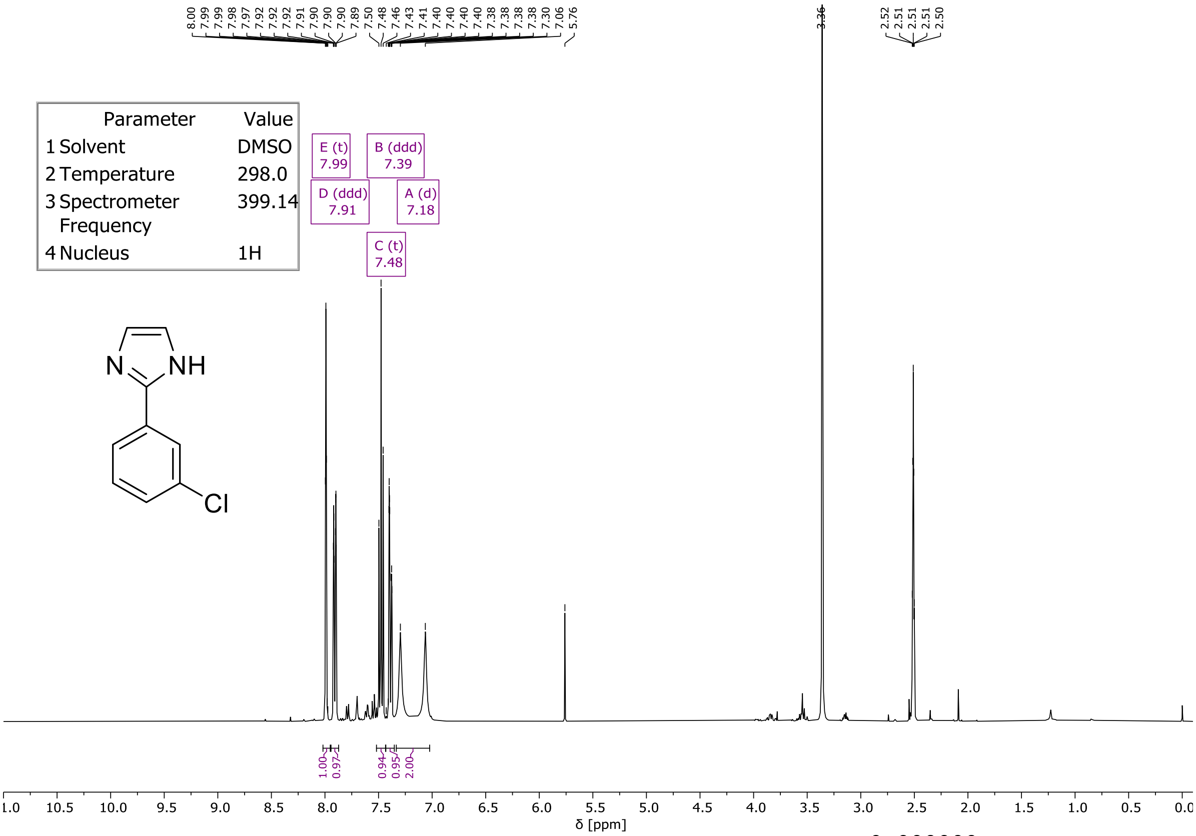

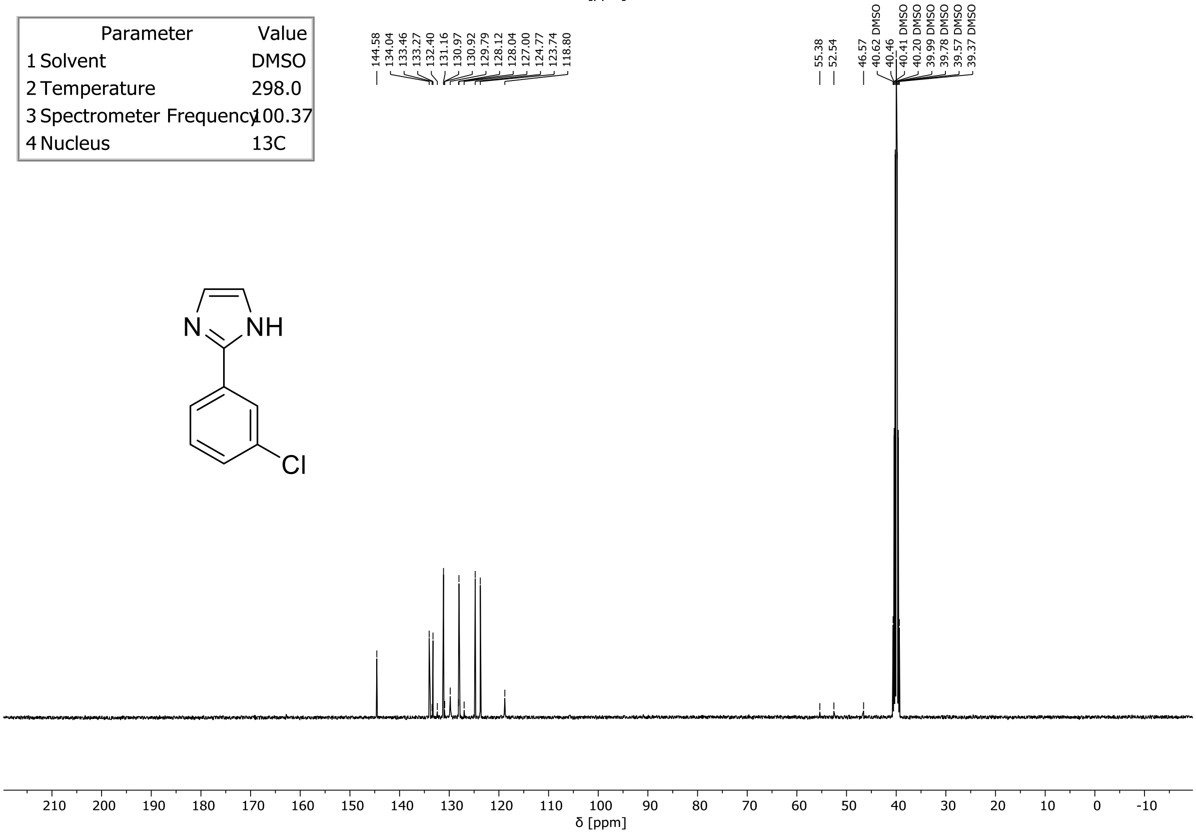

SI Figure S30: ^{1}H- and $^{13}C\{^{1}H\}$-NMR spectra of imidazole **50**.

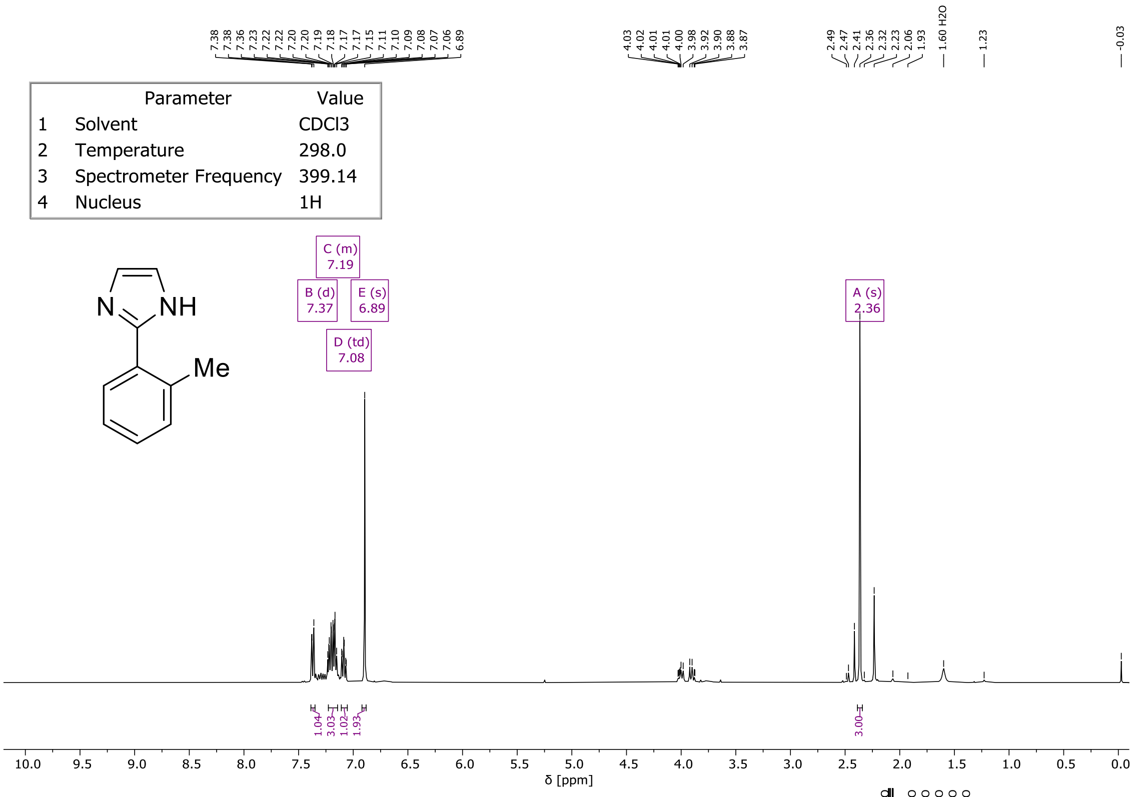

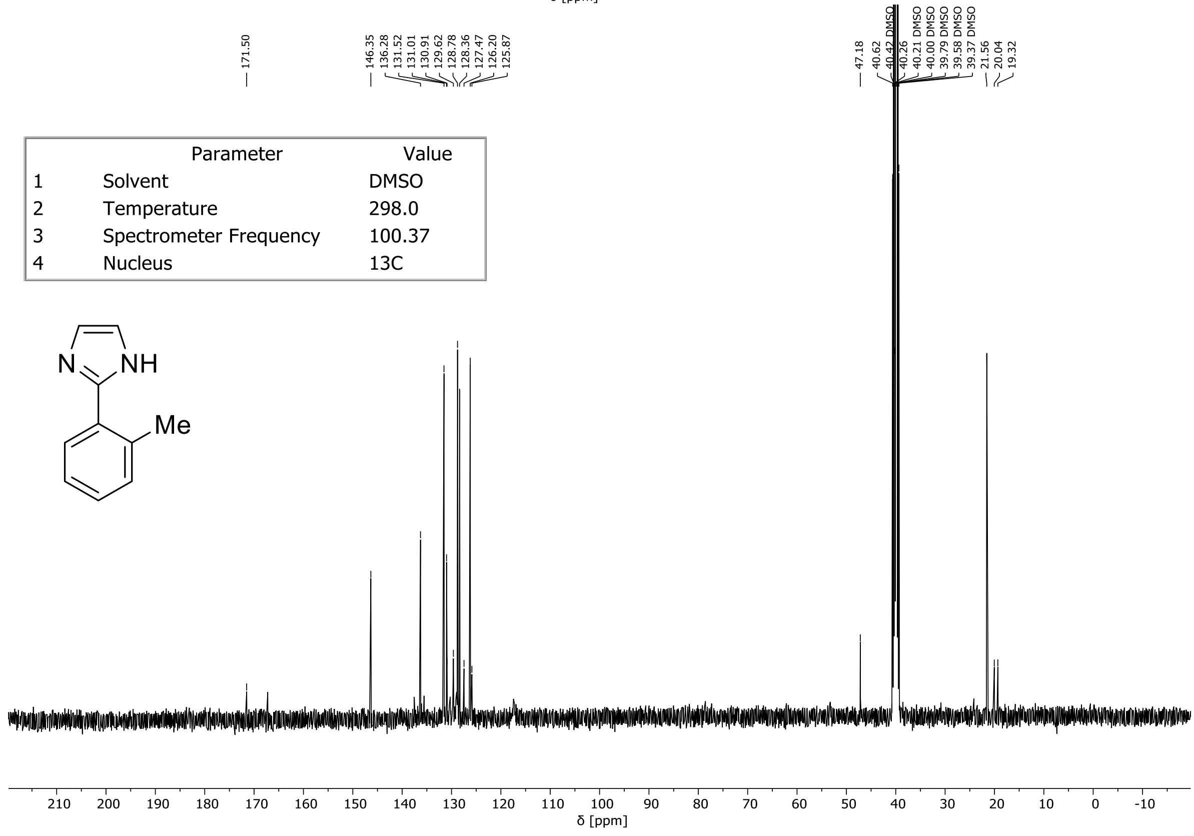

SI Figure S31: ^{1}H- and ^{13}C{^{1}H}-NMR spectra of imidazole **54**.

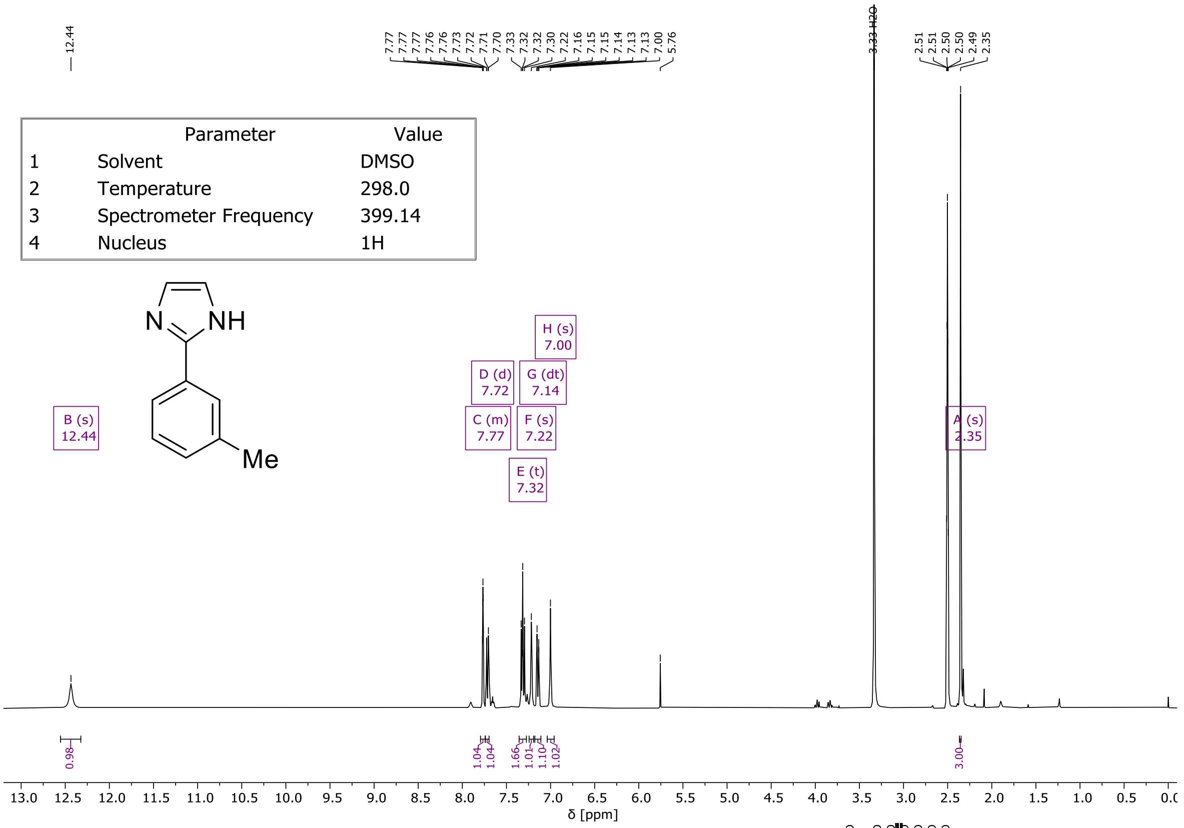

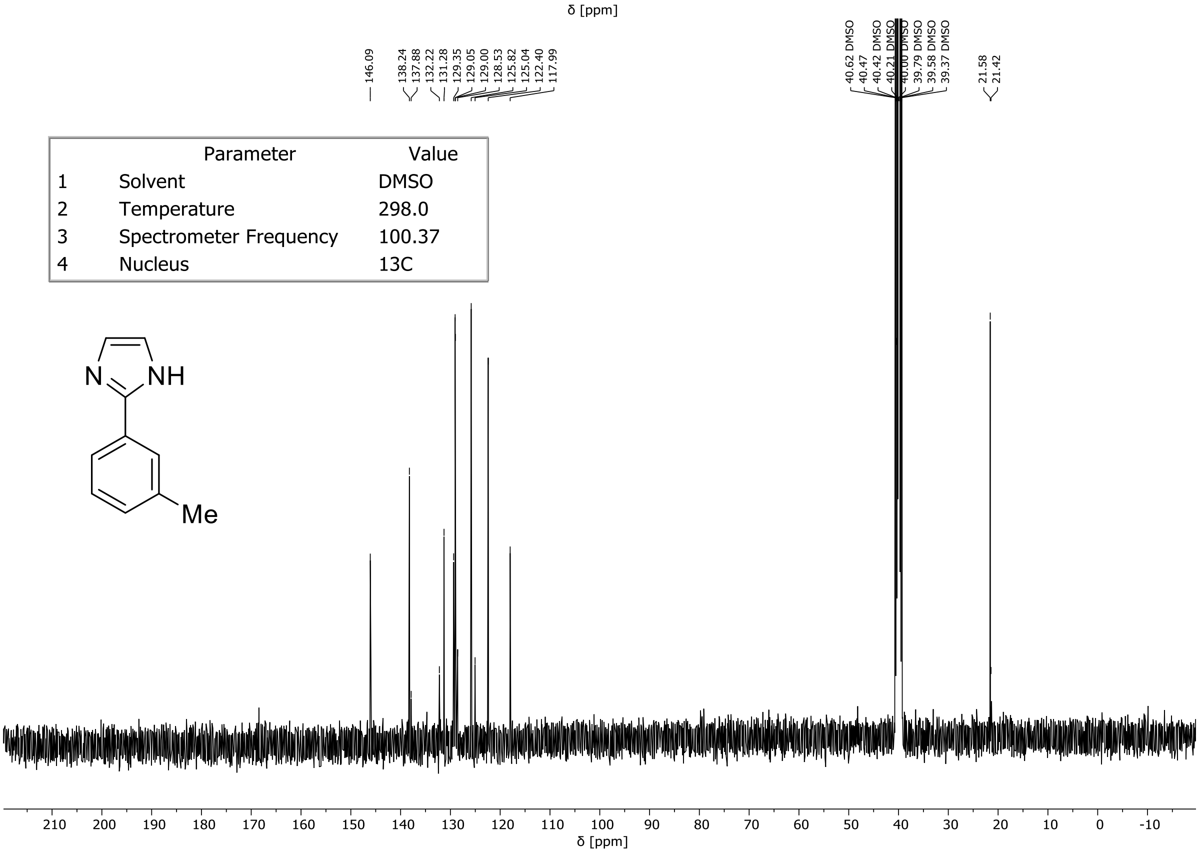

SI Figure S32: ^{1}H- and ^{13}C{^{1}H}-NMR spectra of imidazole **56**.

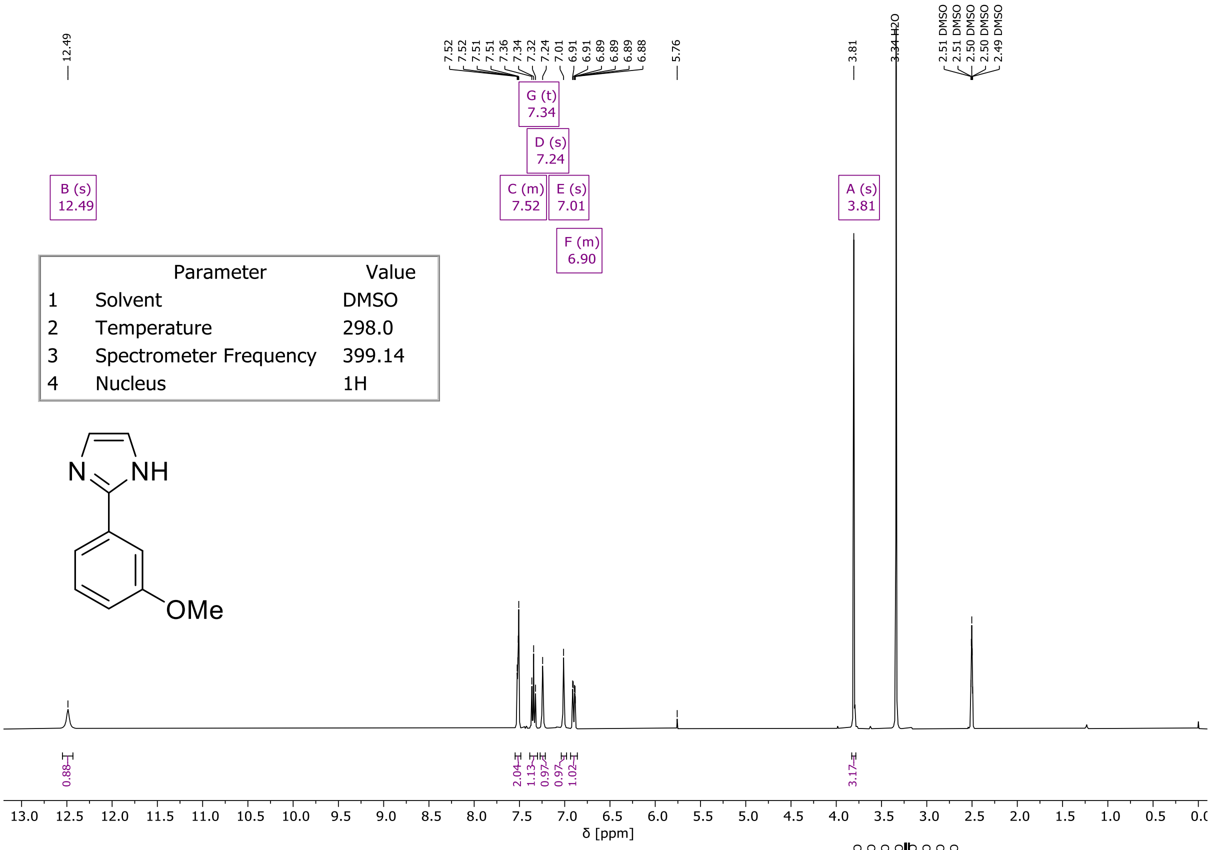

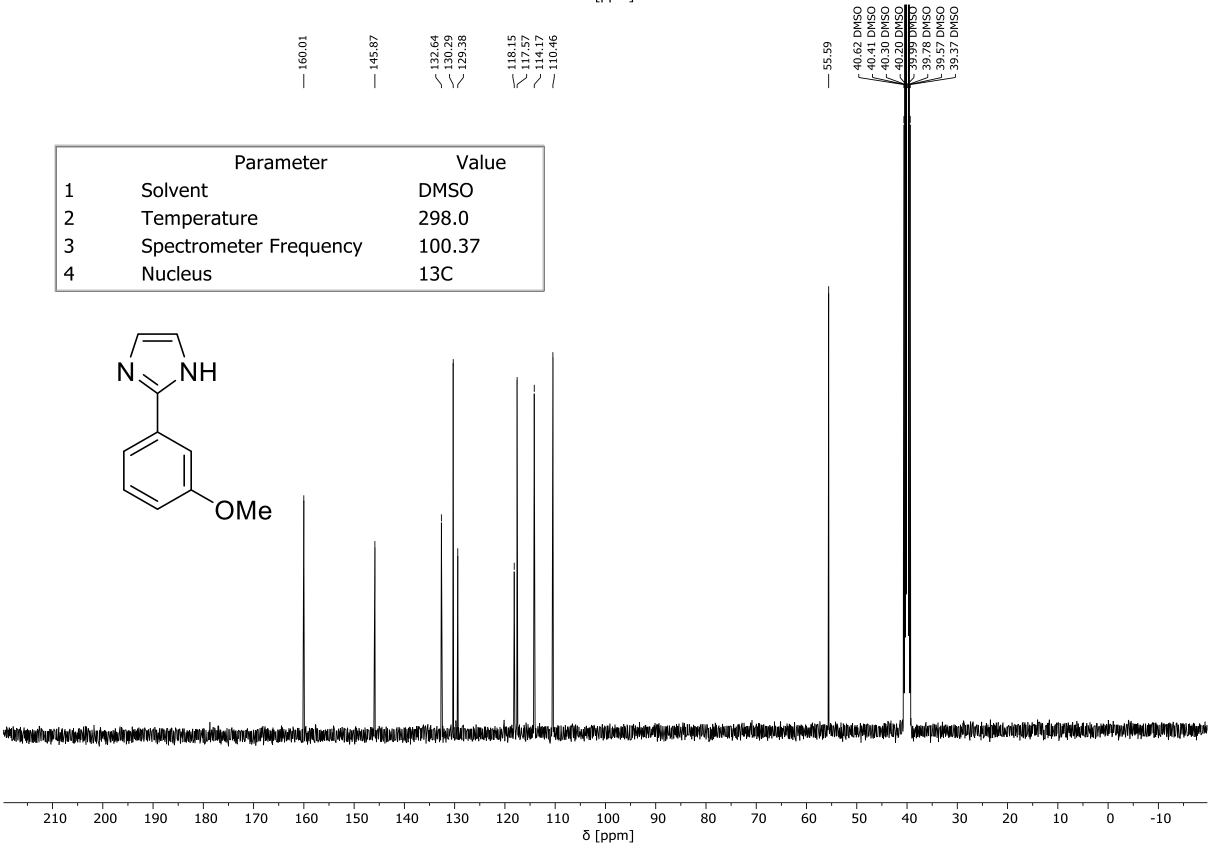

SI Figure S33: ^{1}H- and ^{13}C{^{1}H}-NMR spectra of imidazole **58**.

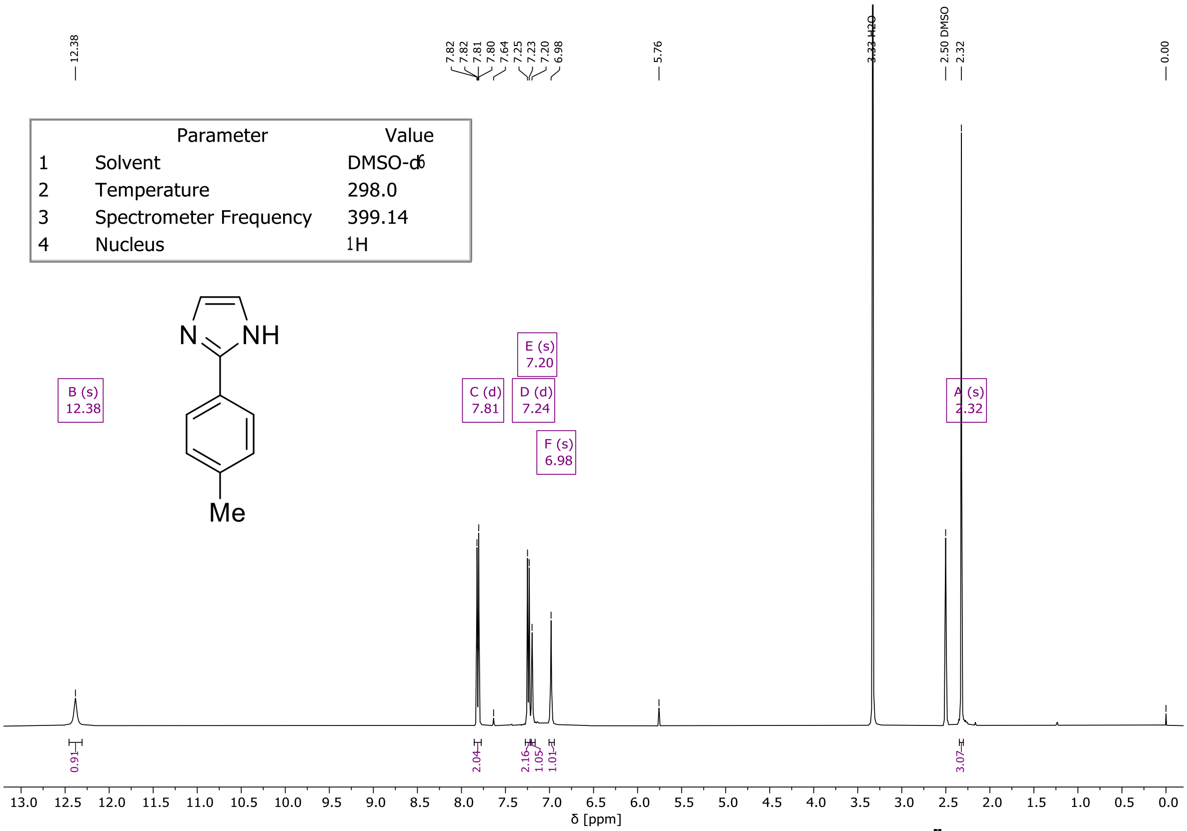

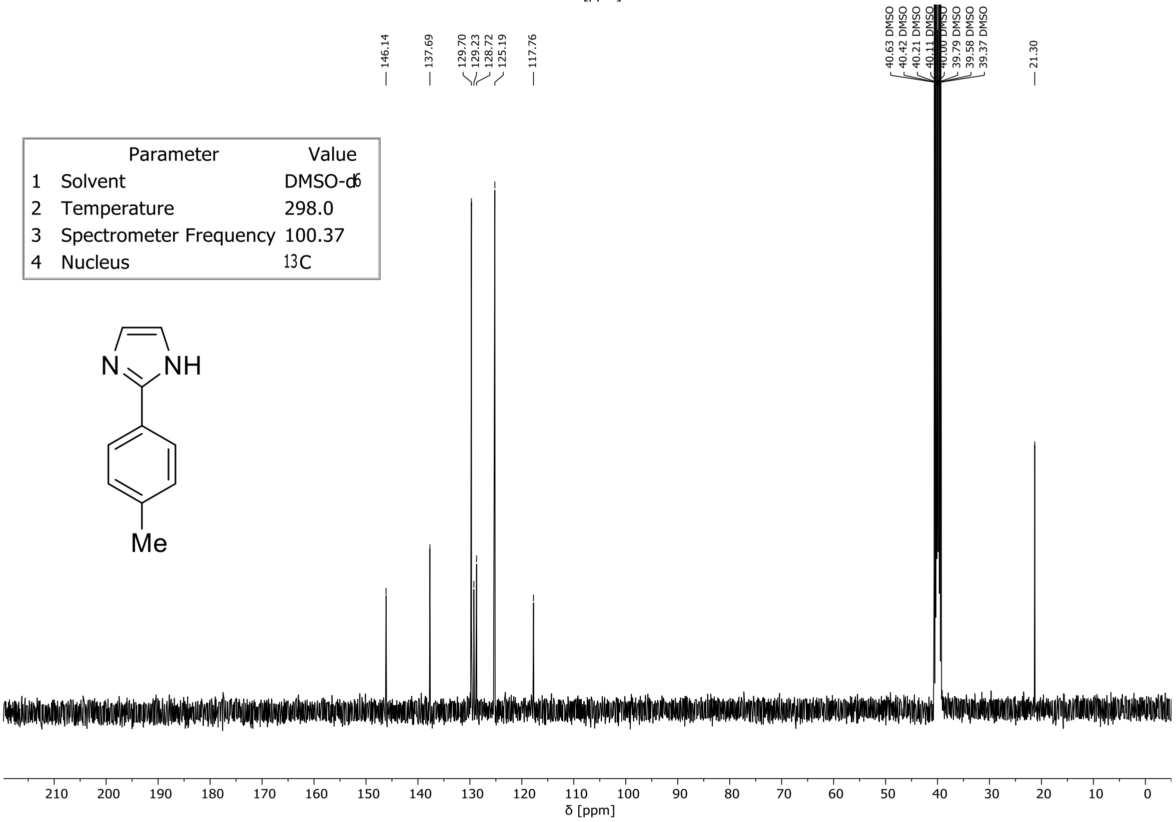

SI Figure34: ^{1}H- and ^{13}C{^{1}H}-NMR spectra of imidazole **73**.

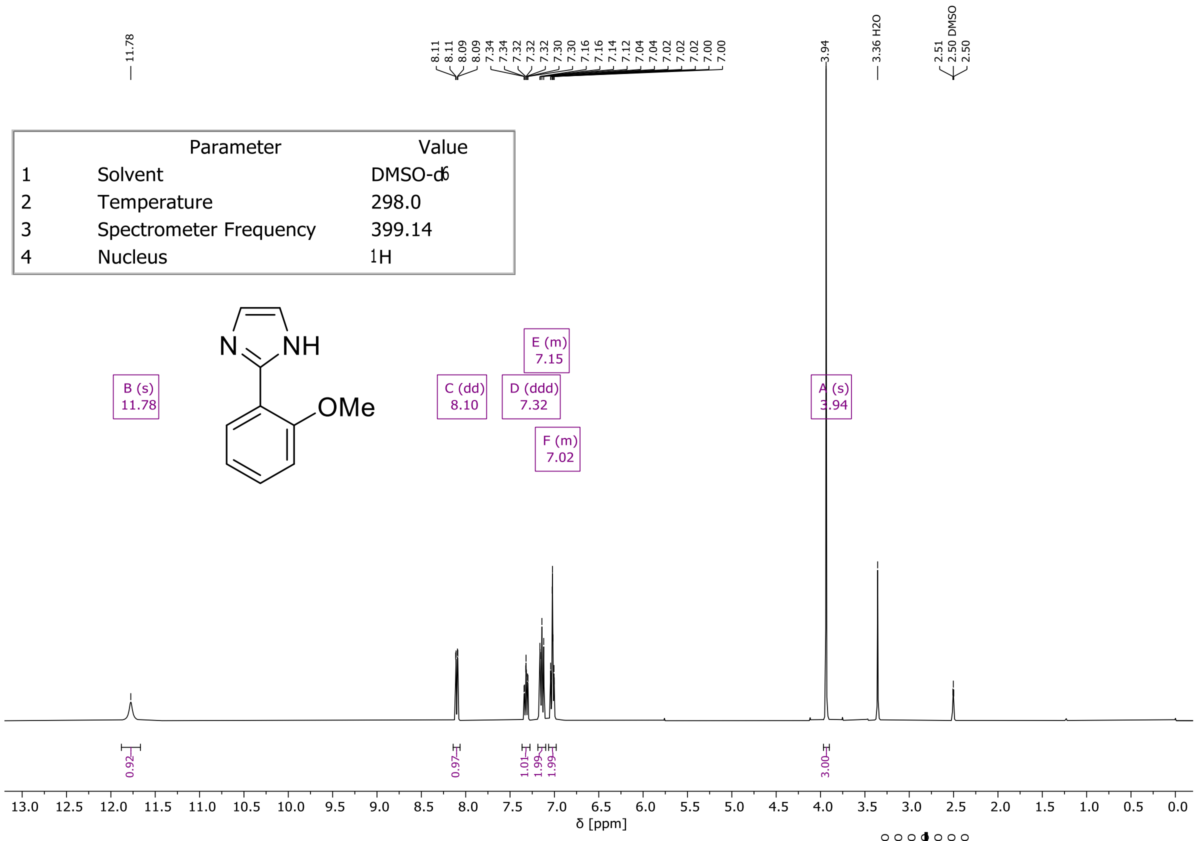

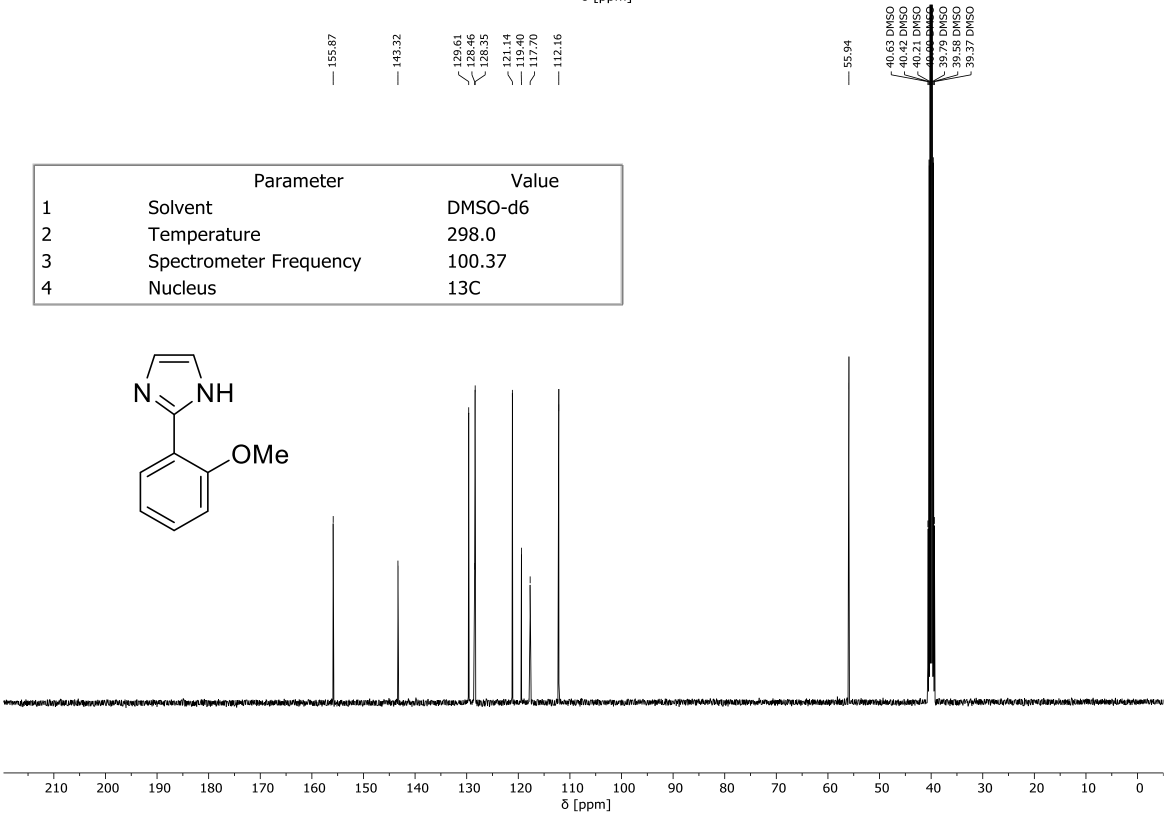

SI Figure S35: ^{1}H- and ^{13}C{^{1}H}-NMR spectra of imidazole **74**.

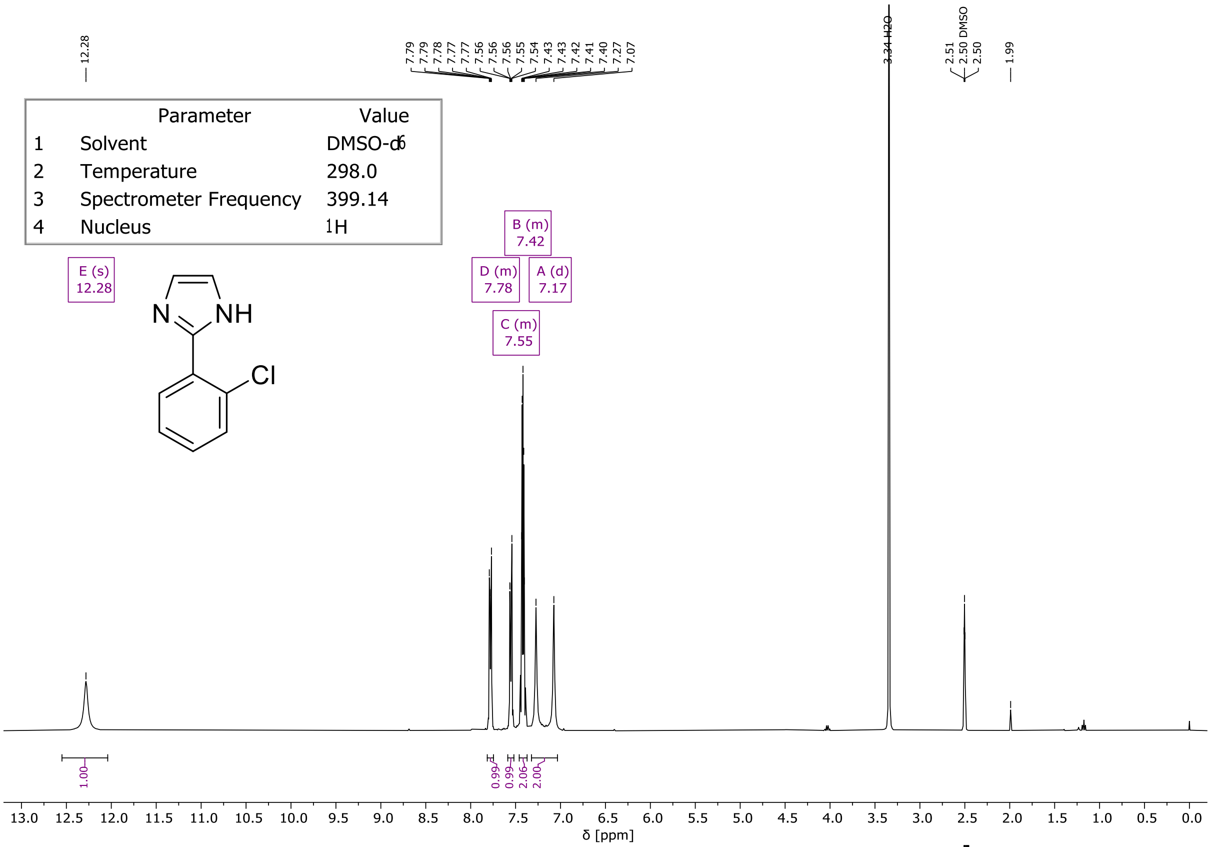

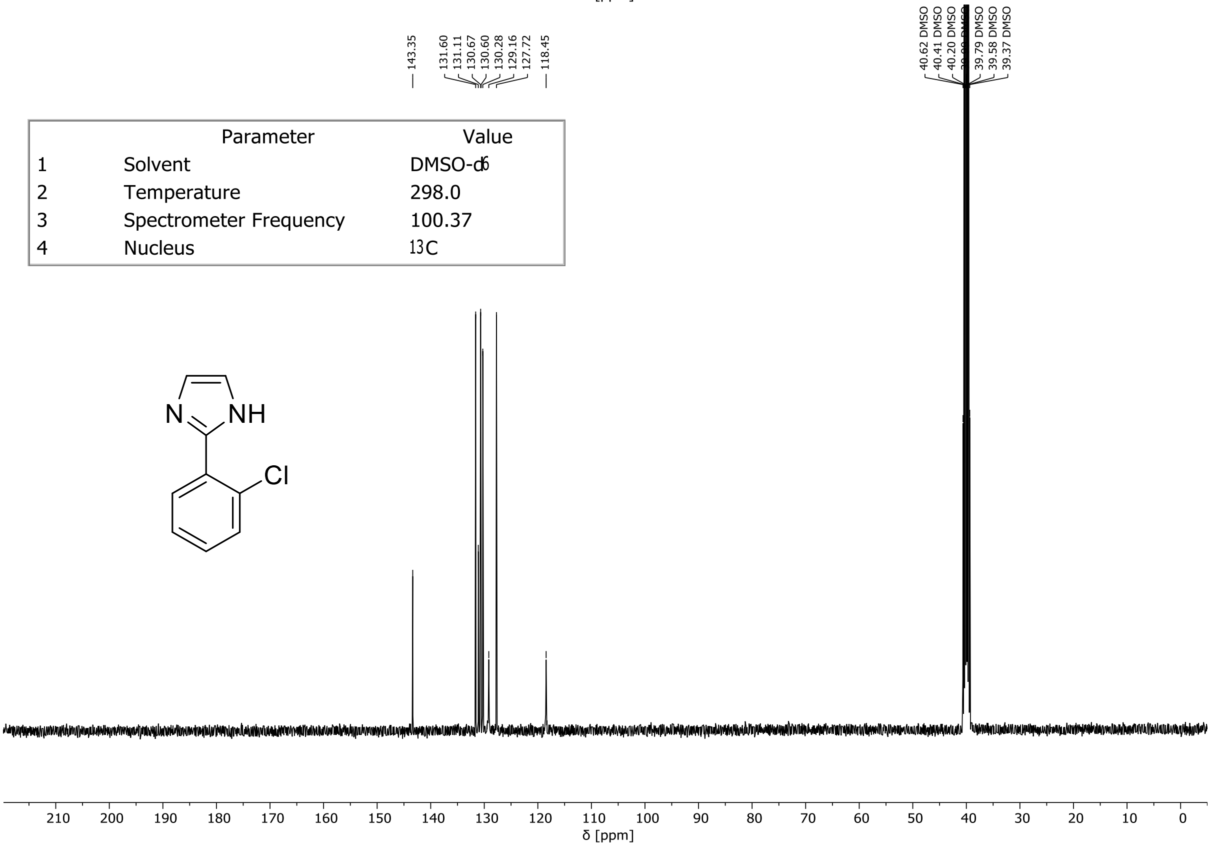

SI Figure S36: ^{1}H- and $^{13}C\{^{1}H\}$-NMR spectra of imidazole **75**.

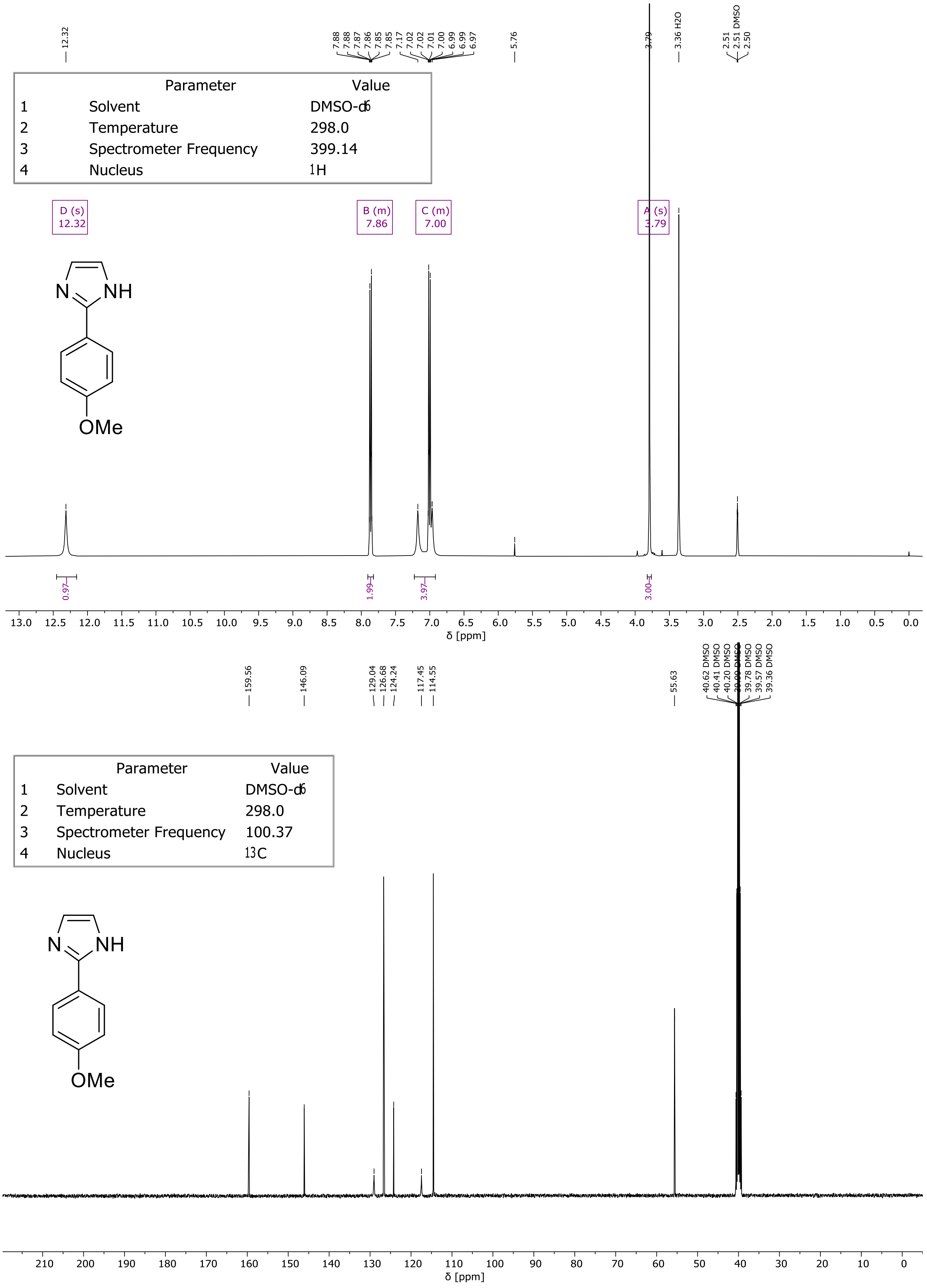

SI Figure S37: ^{1}H- and ^{13}C{^{1}H}-NMR spectra of imidazole **76**.

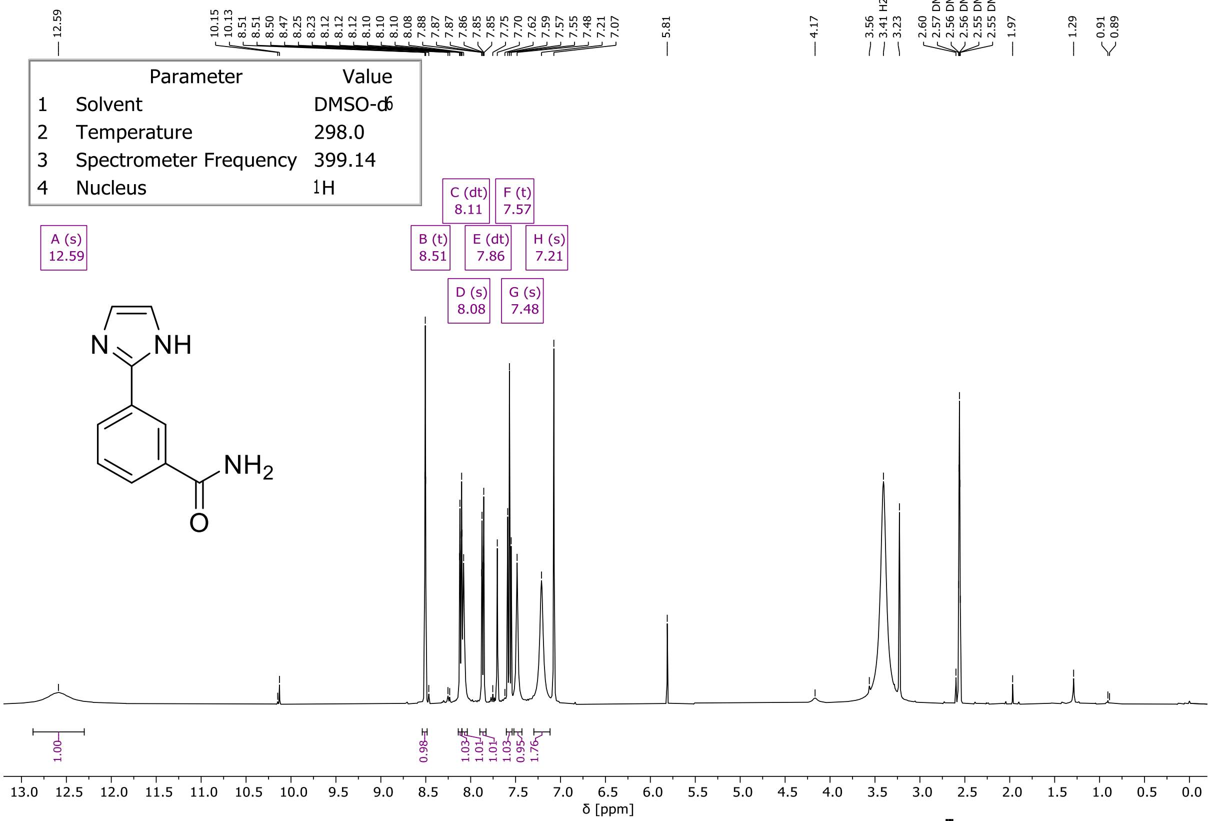

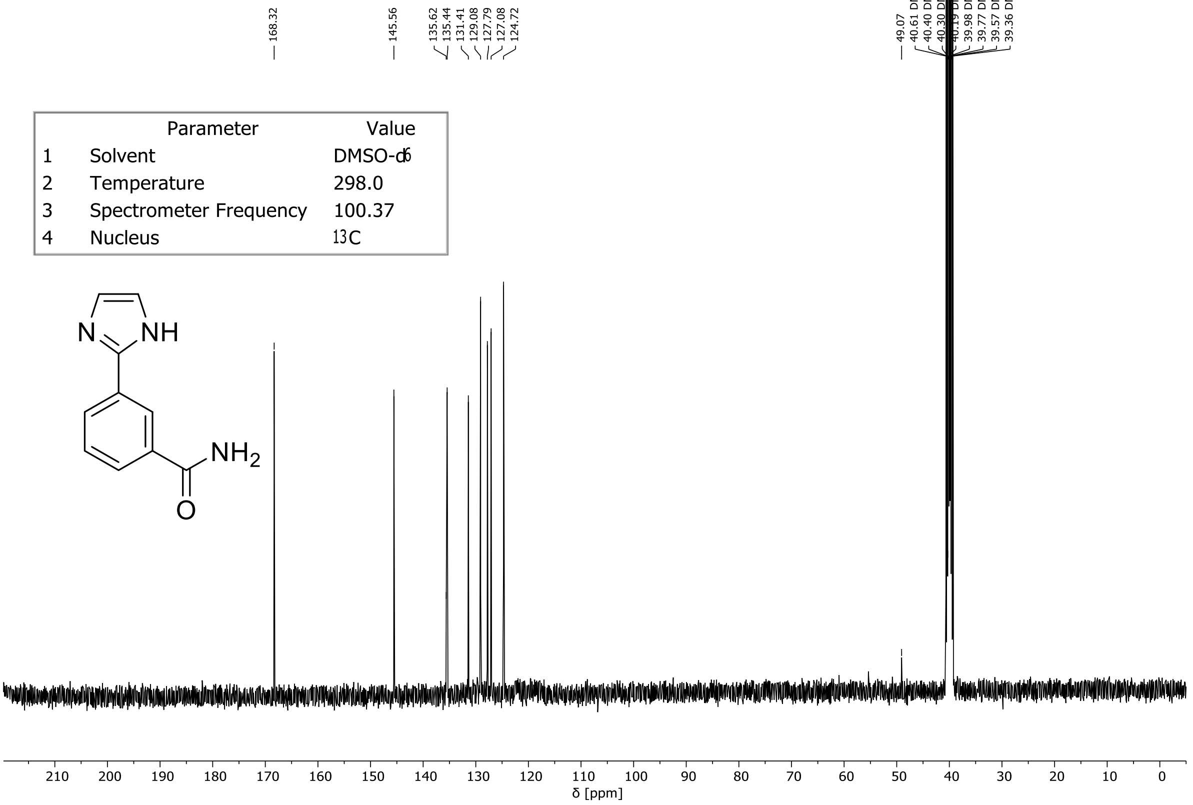

SI Figure S38: ^{1}H- and ^{13}C{^{1}H}-NMR spectra of imidazole **77**.

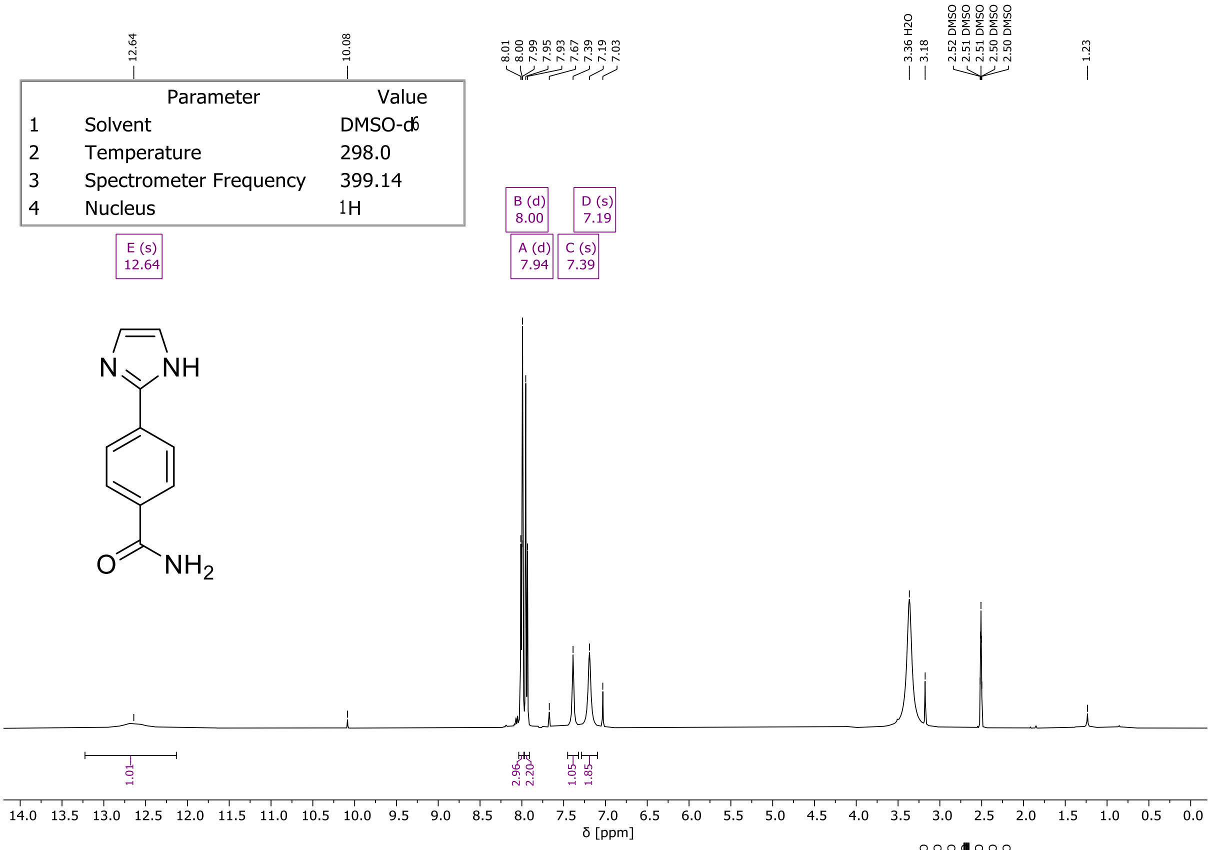

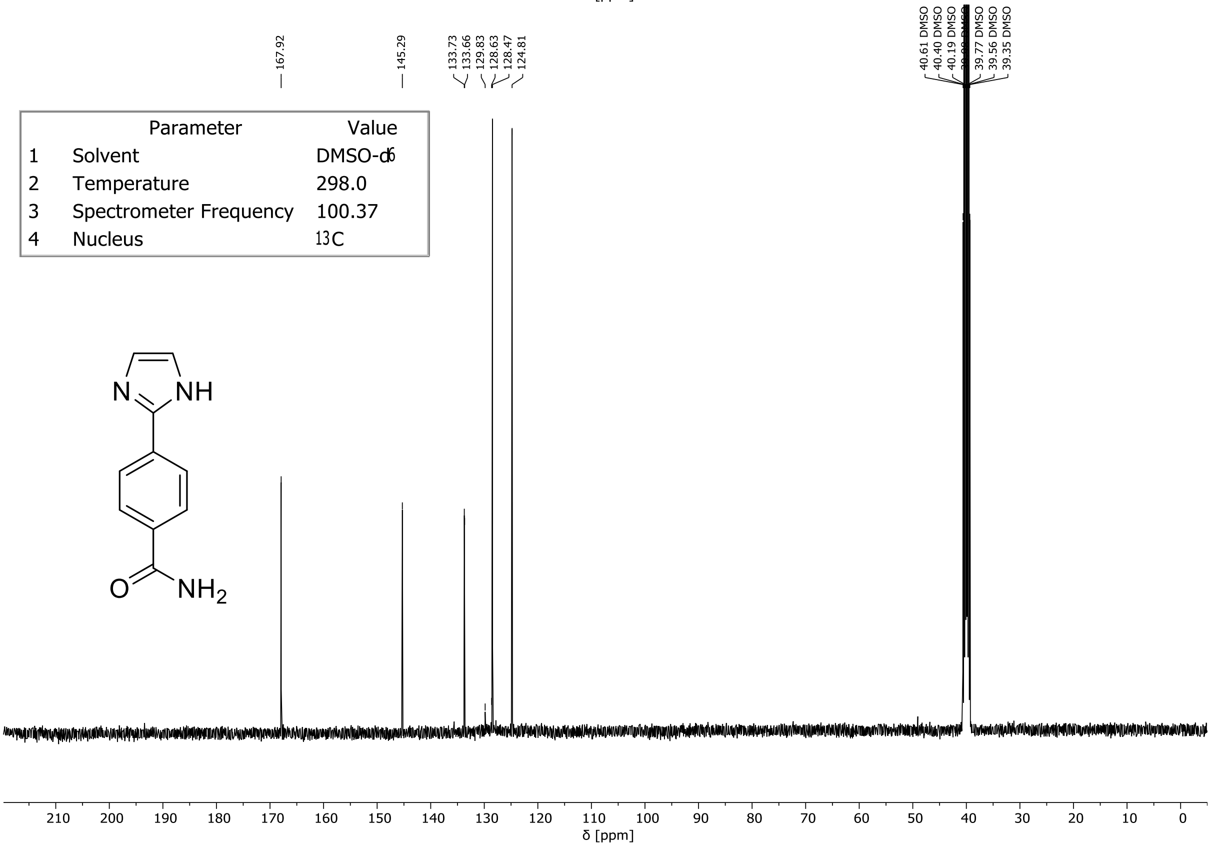

SI Figure S39: ^{1}H- and ^{13}C{^{1}H}-NMR spectra of imidazole **78**.

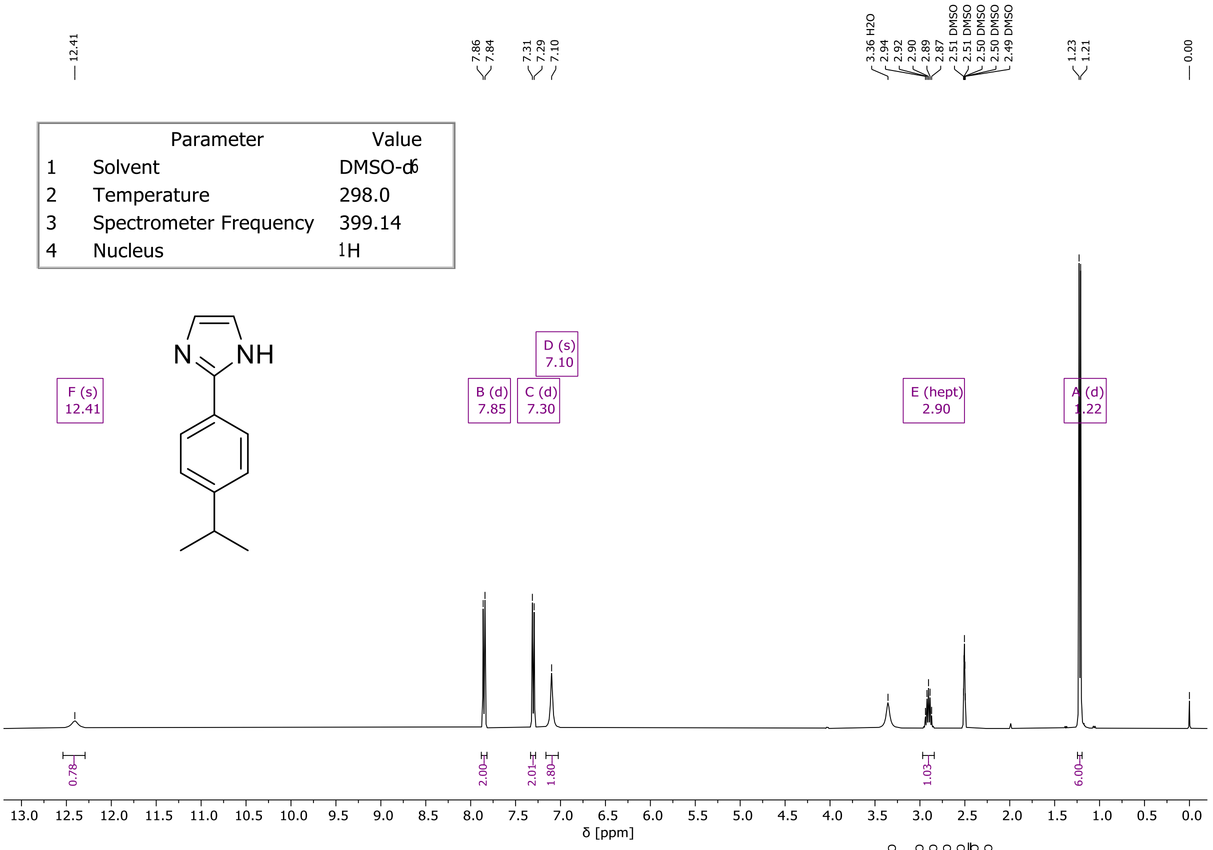

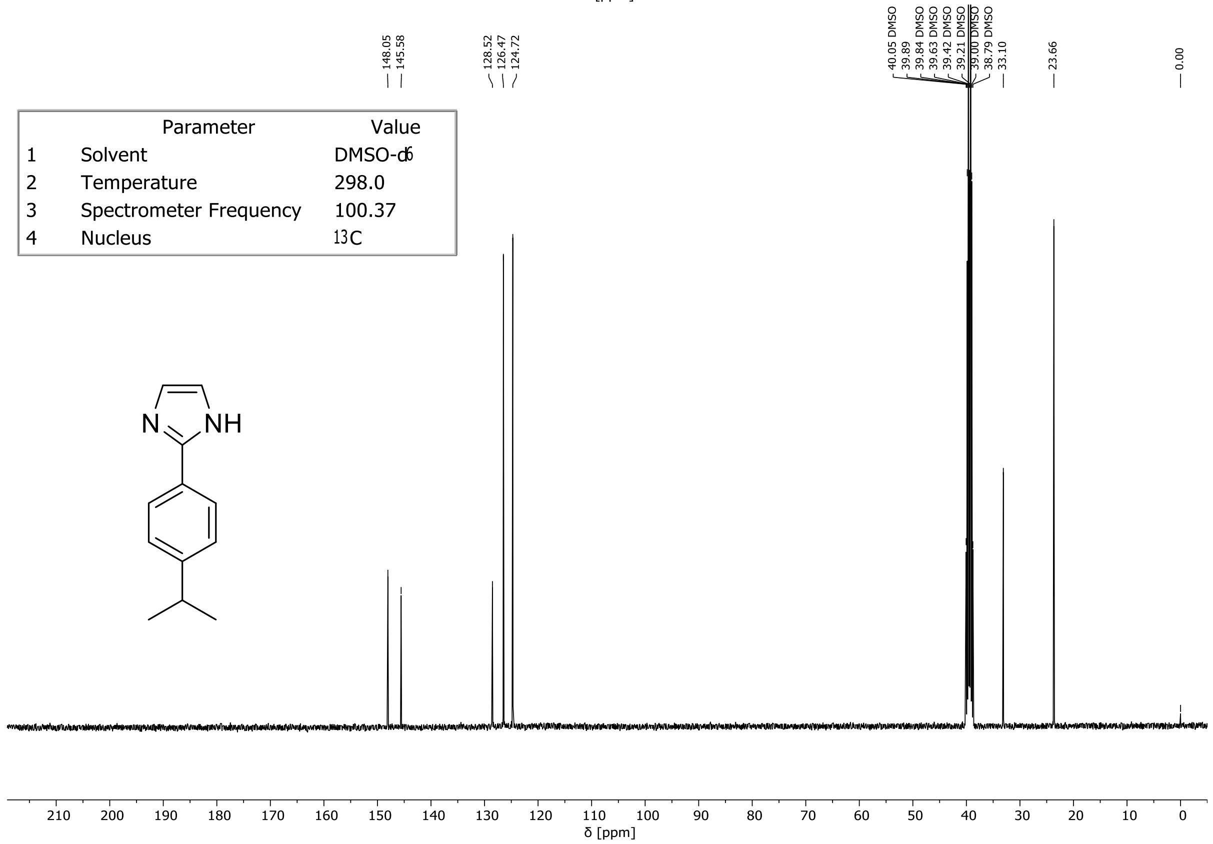

SI Figure S40: ^{1}H- and ^{13}C{^{1}H}-NMR spectra of imidazole **79a**.

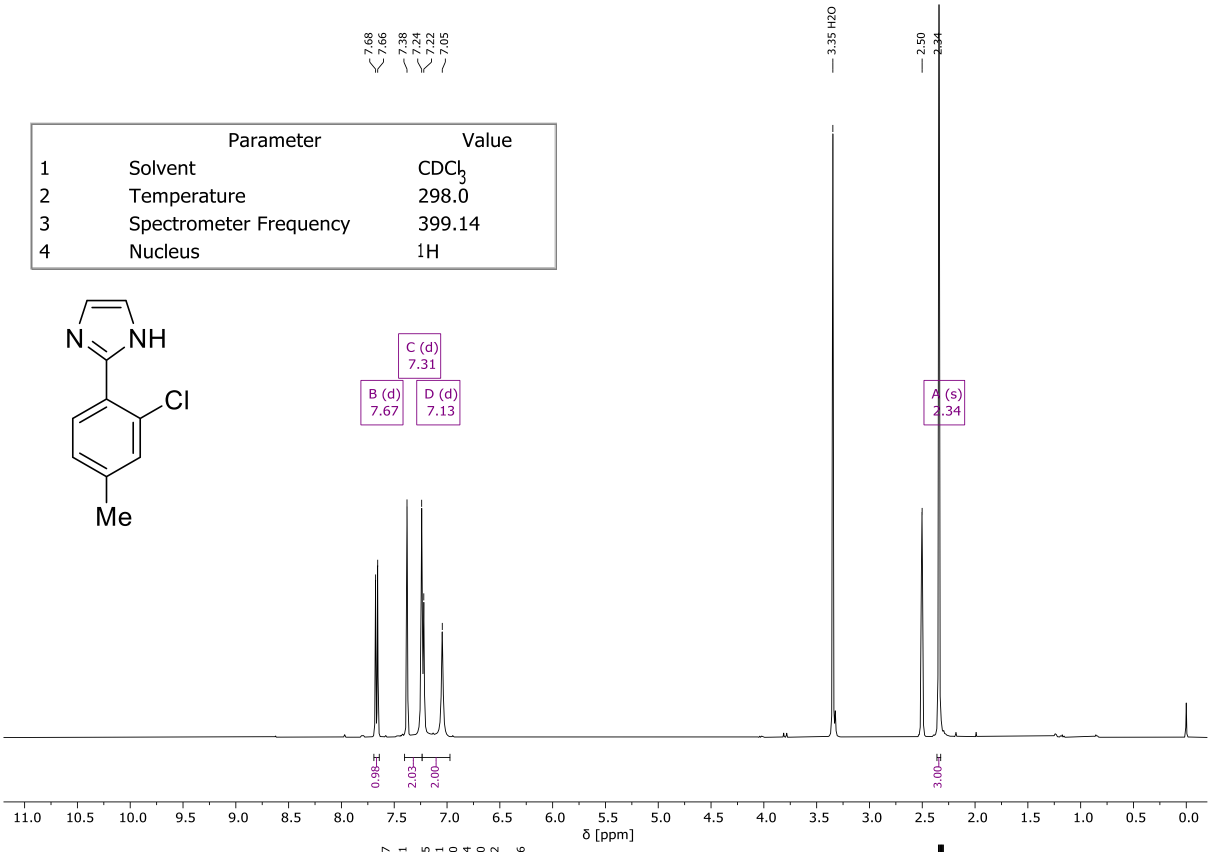

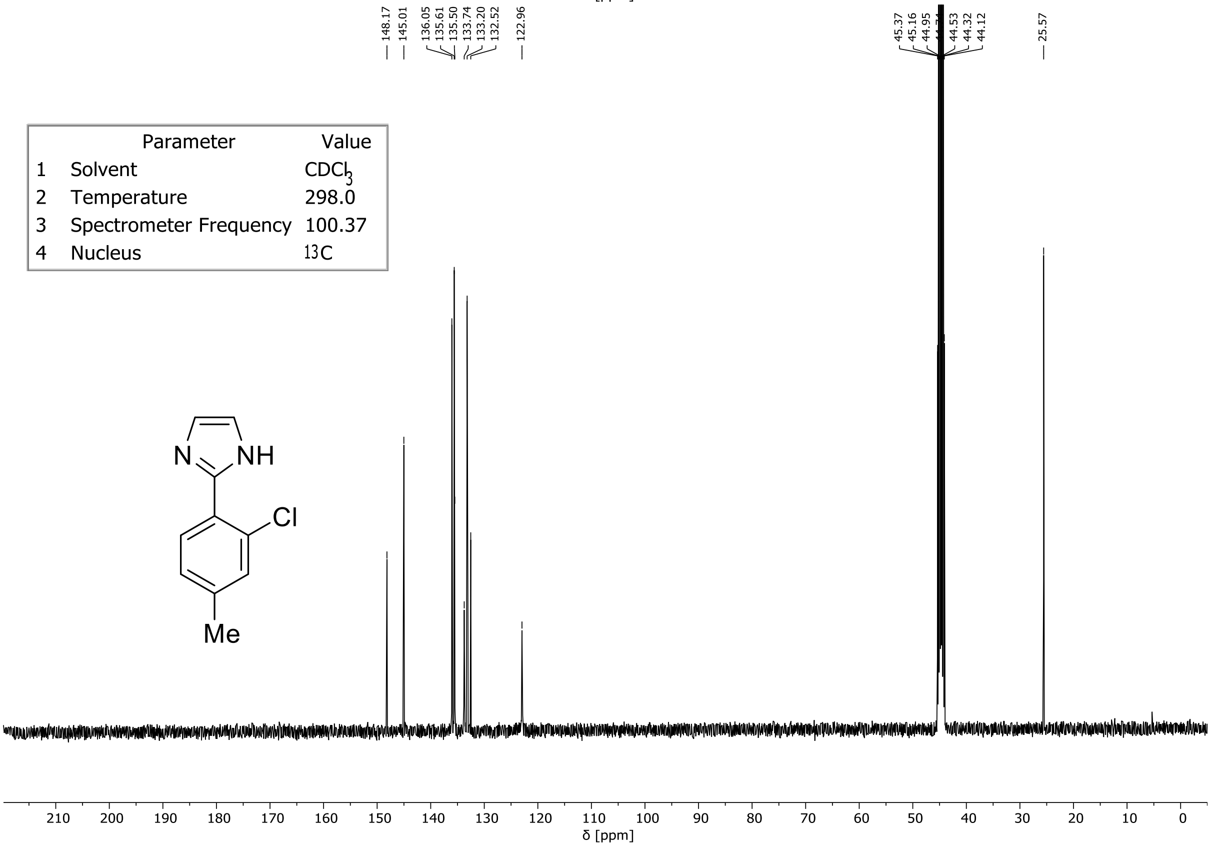

SI Figure S41: ^{1}H- and ^{13}C{^{1}H}-NMR spectra of imidazole **79b**.

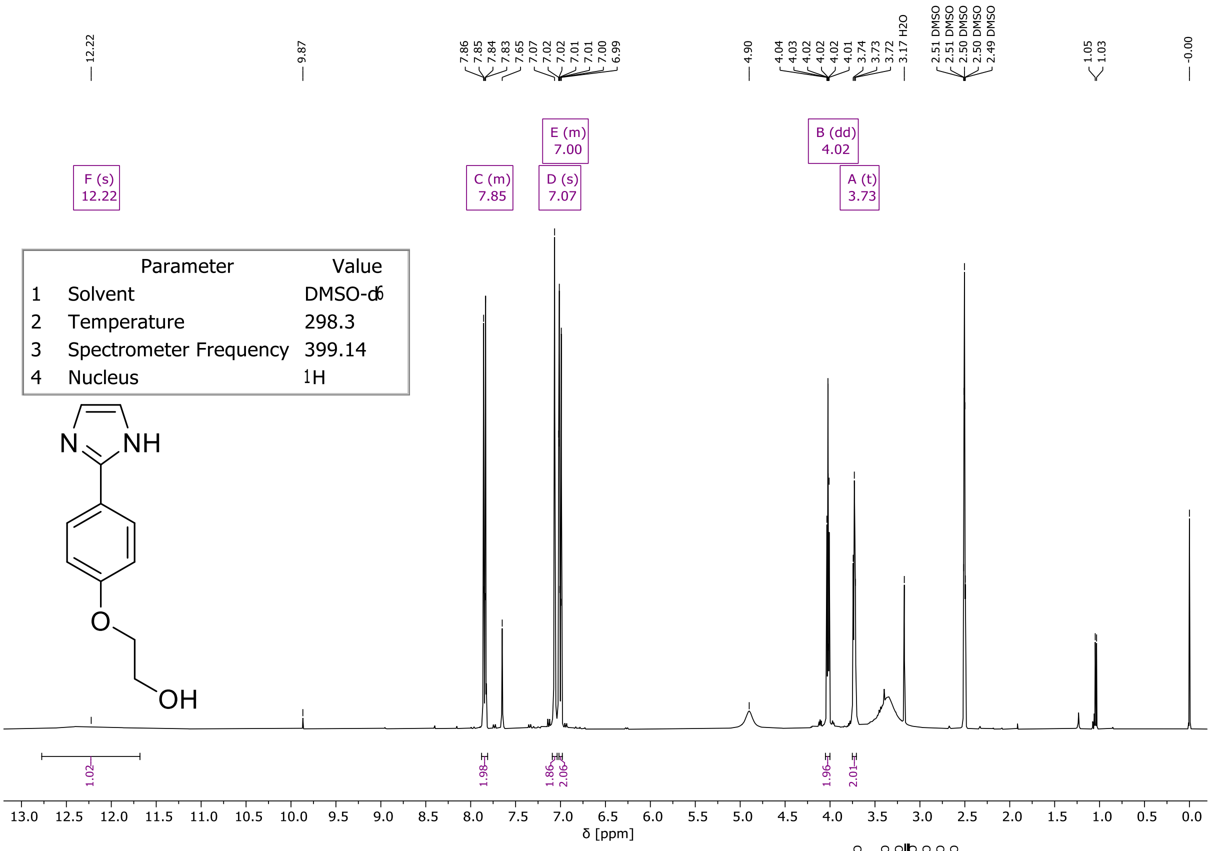

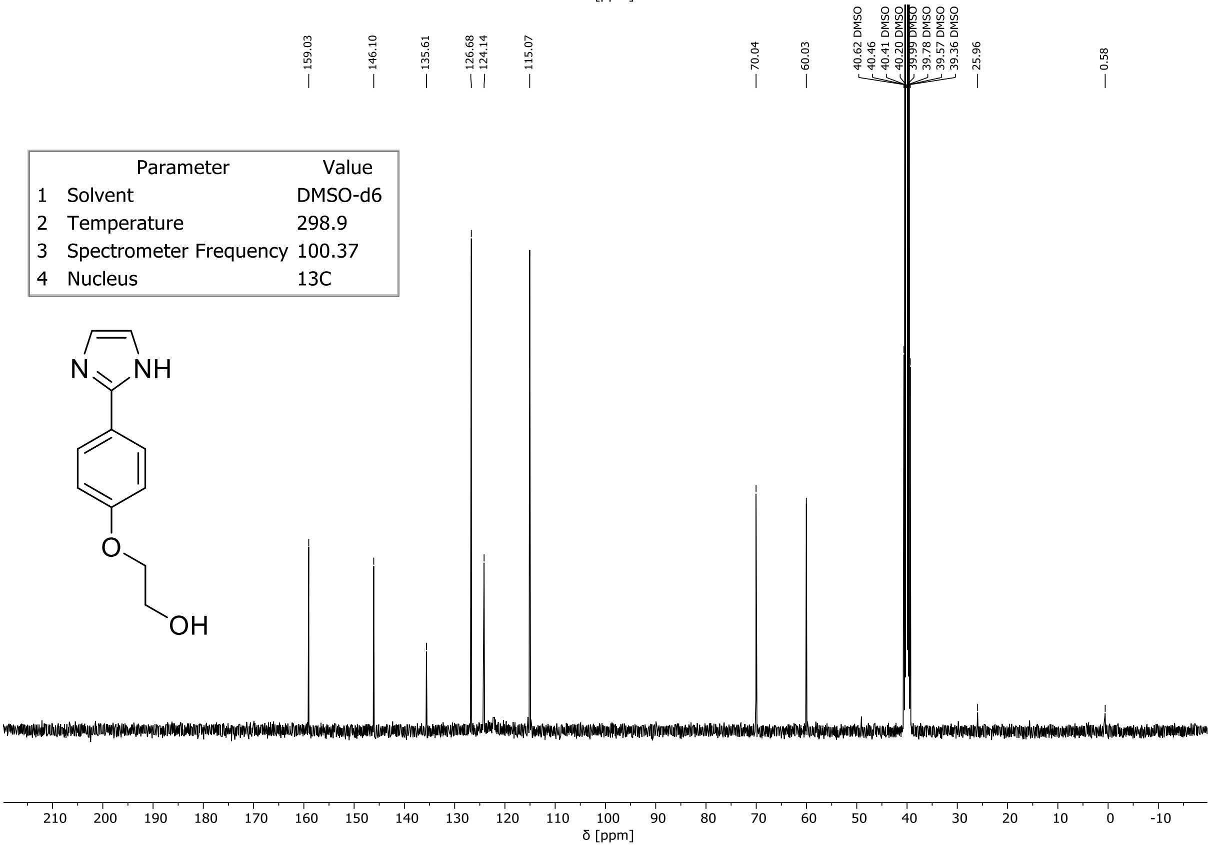

SI Figure S42: ^{1}H- and $^{13}C\{^{1}H\}$-NMR spectra of imidazole **80**.

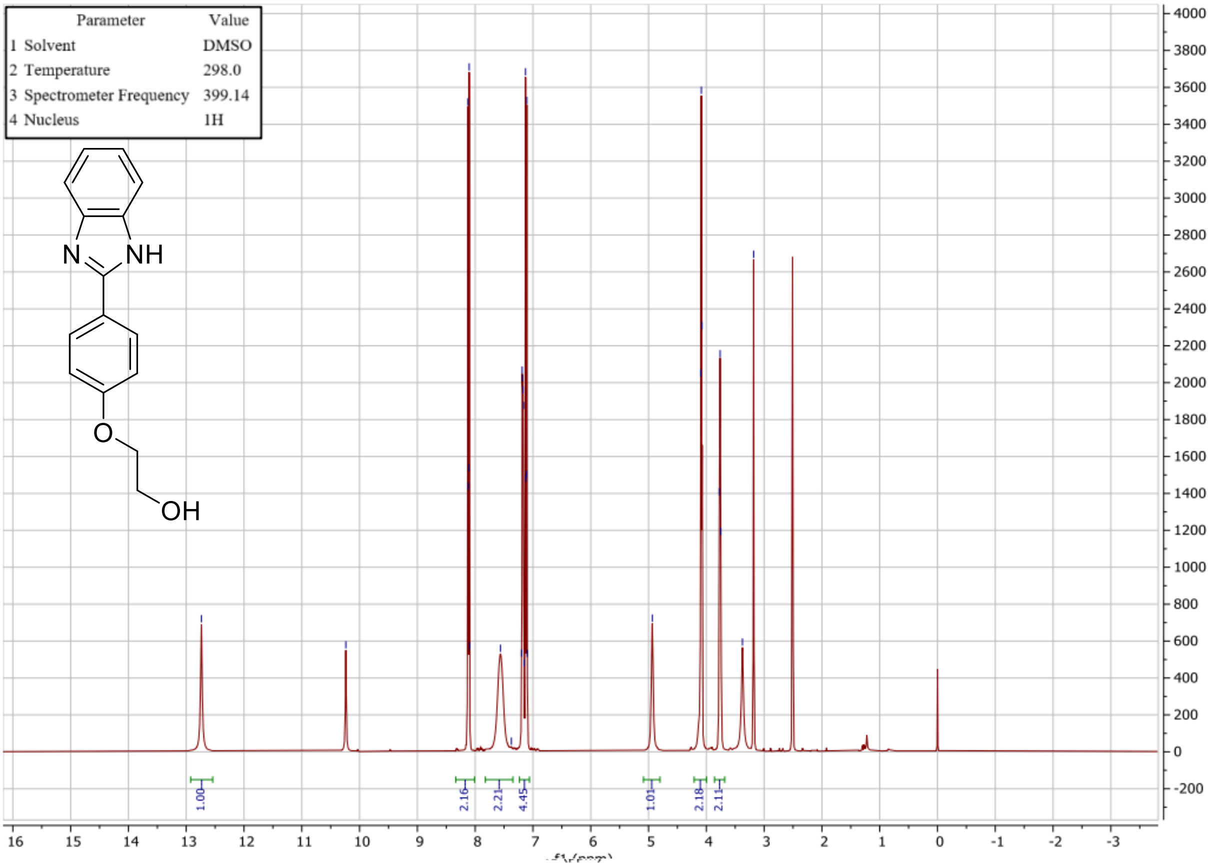

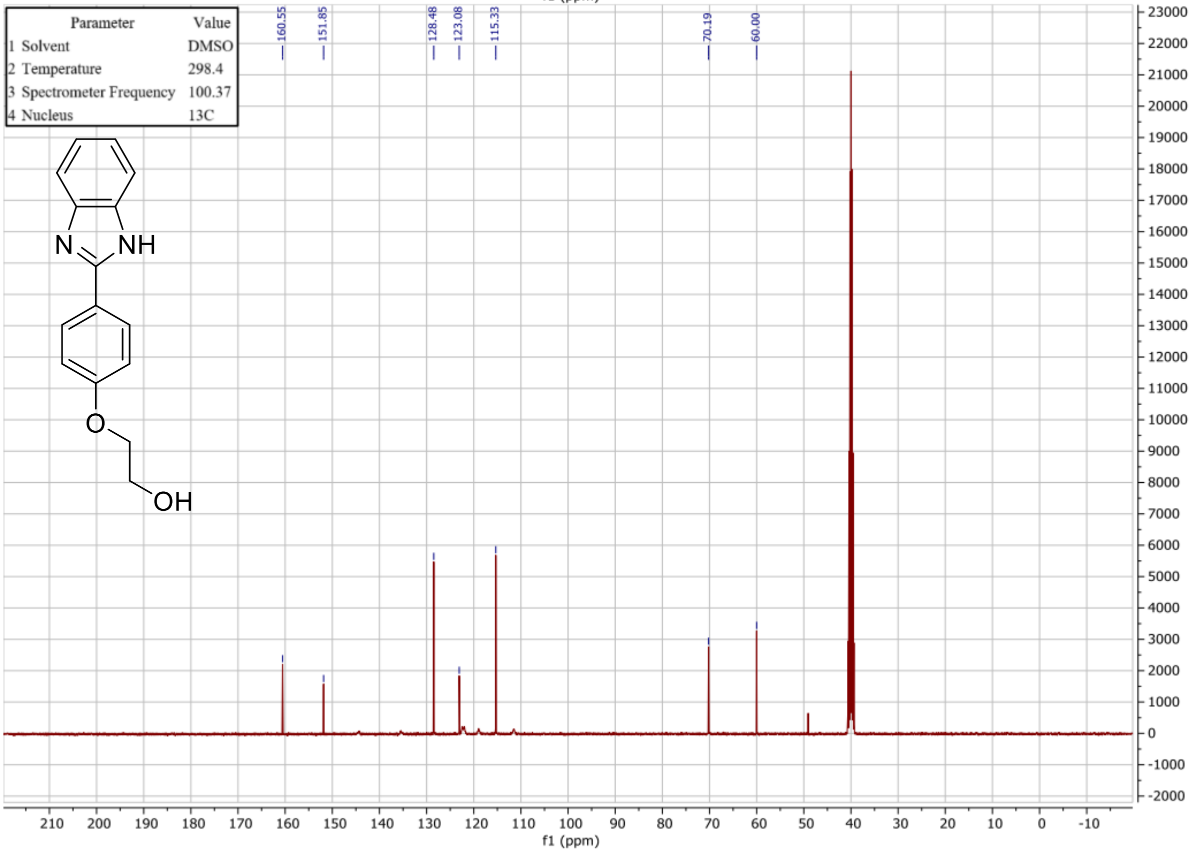

SI Figure S43: ^{1}H- and ^{13}C{^{1}H}-NMR spectra of imidazole **92**.

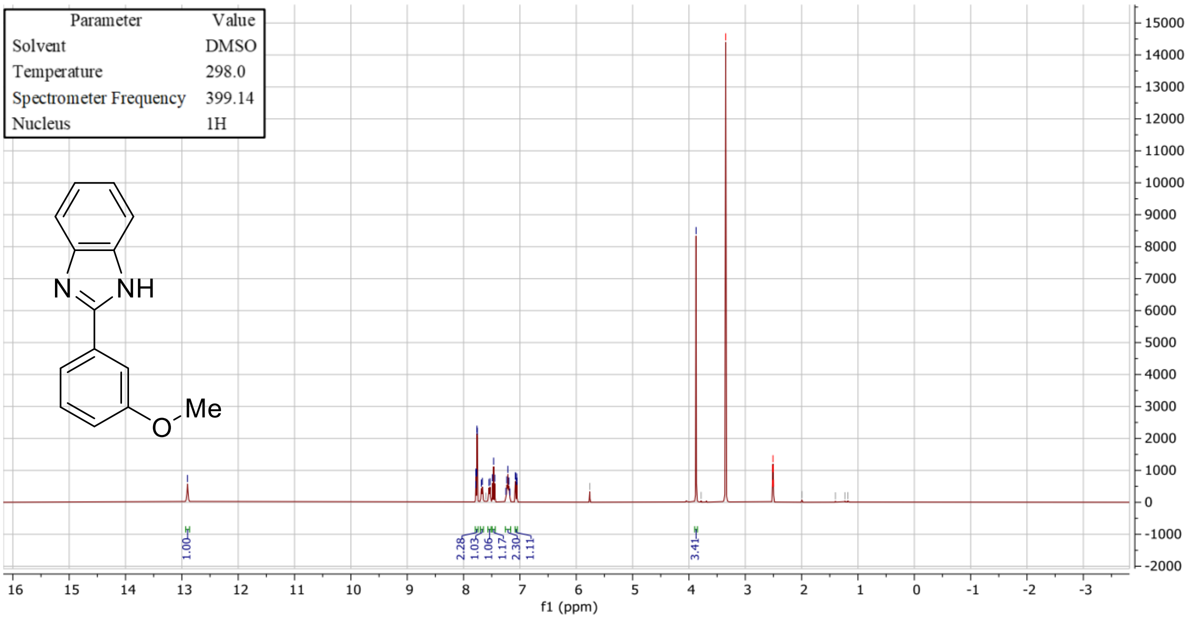

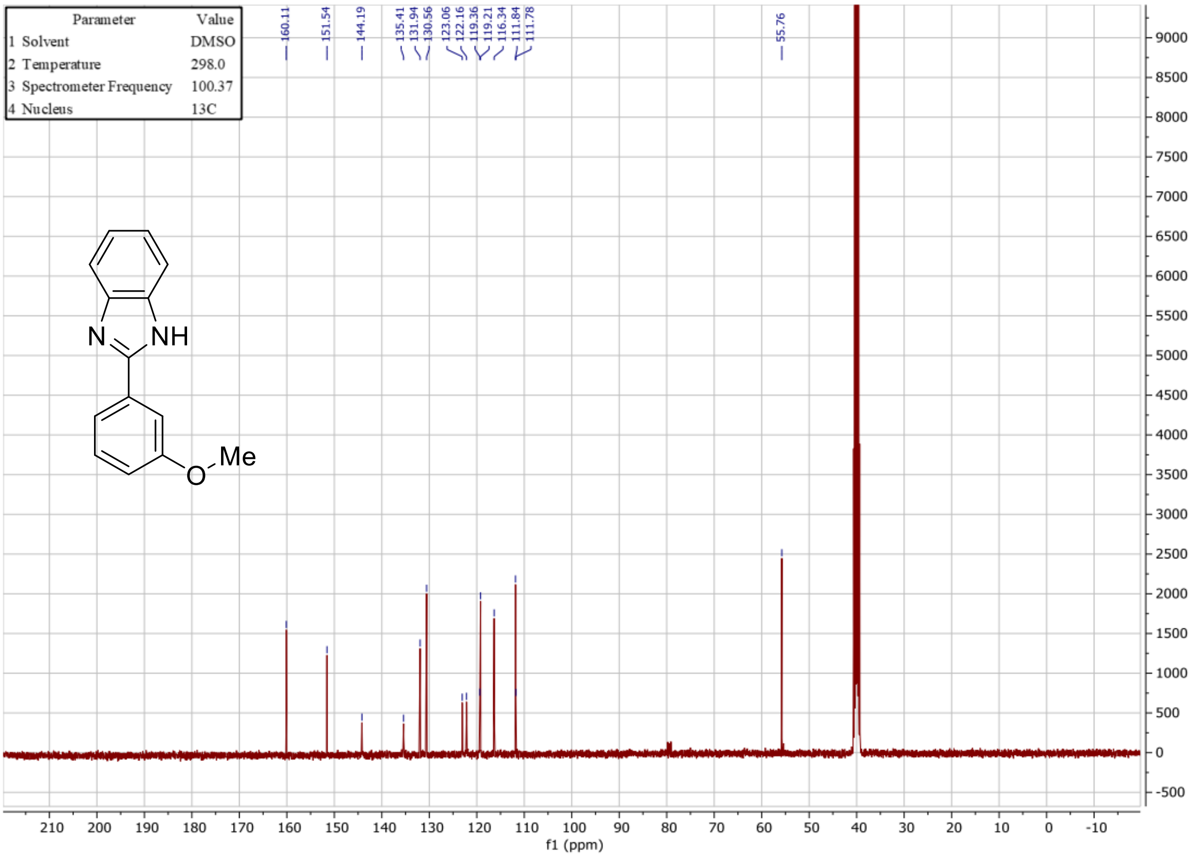

SI Figure S44: ^{1}H- and ^{13}C{^{1}H}-NMR spectra of imidazole **89**.

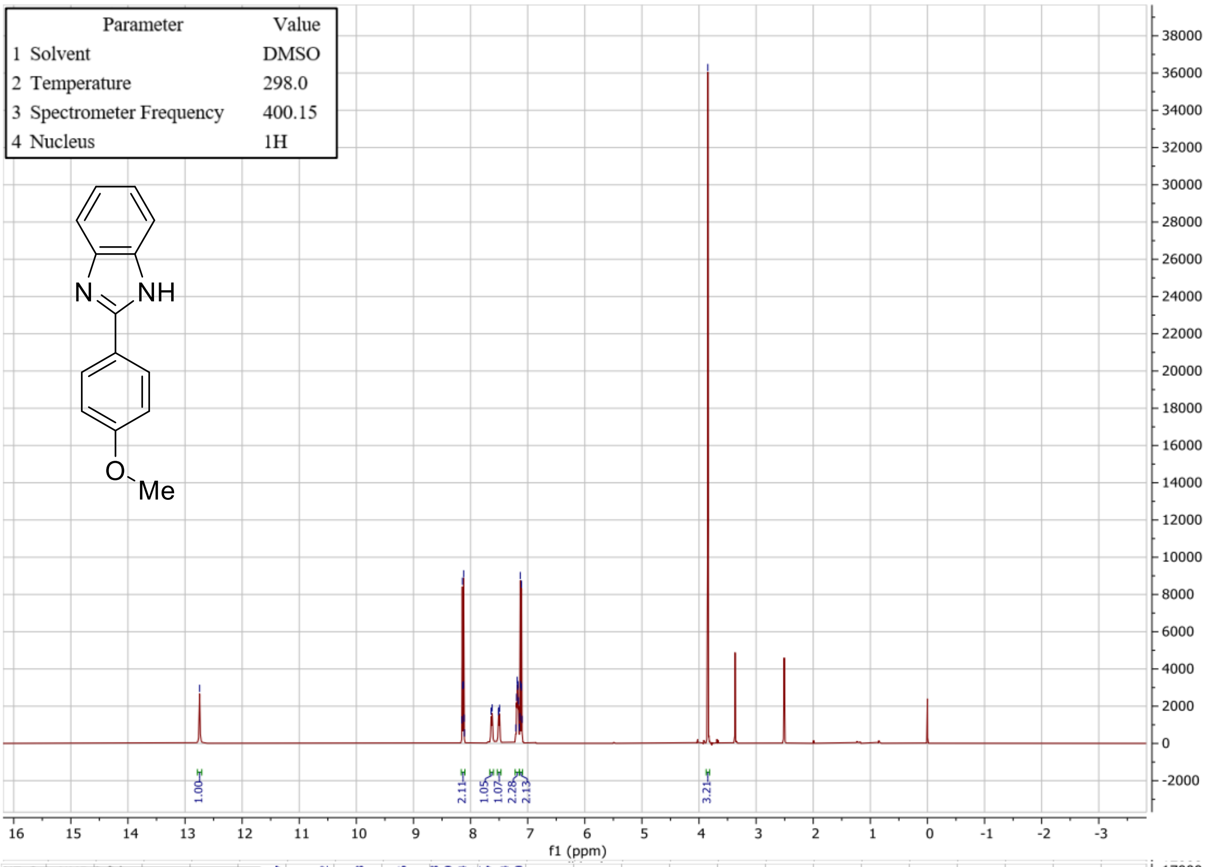

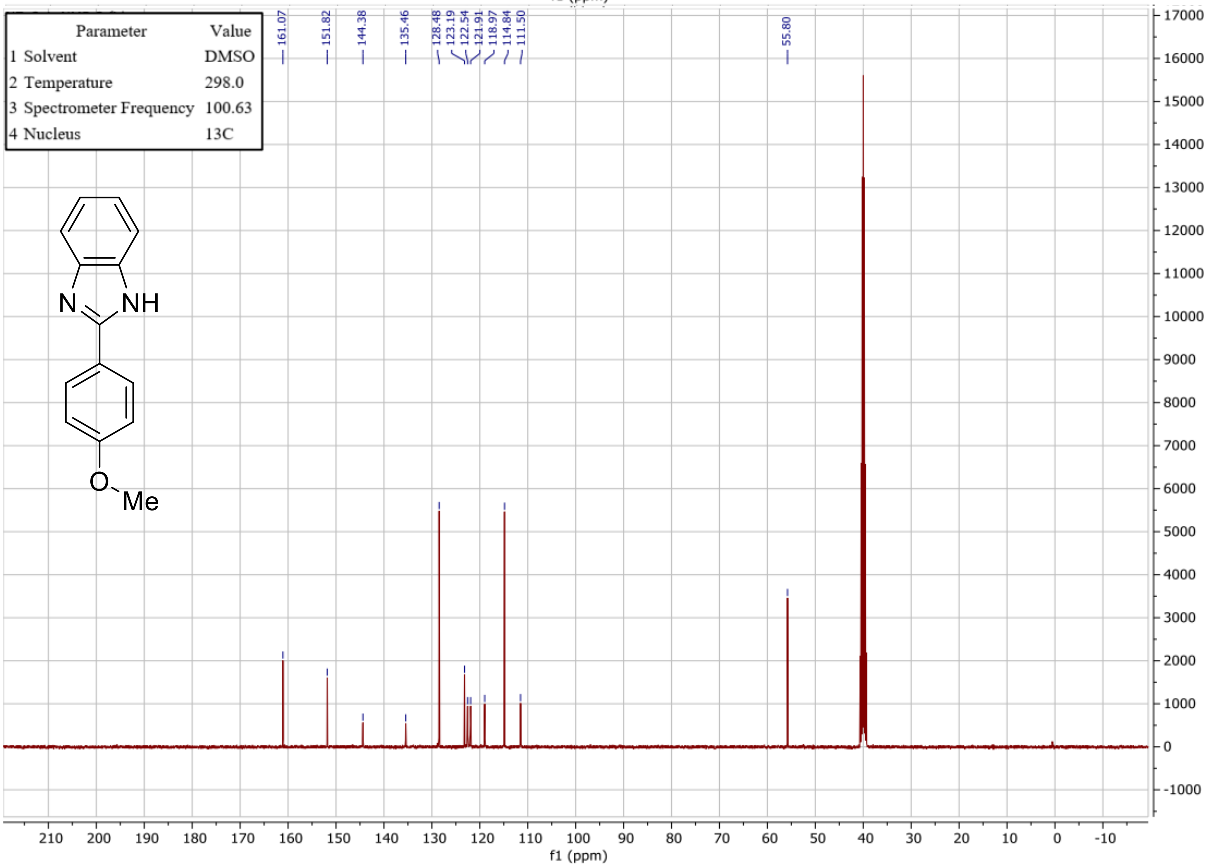

SI Figure S45: ^{1}H- and ^{13}C{^{1}H}-NMR spectra of imidazole **90**.

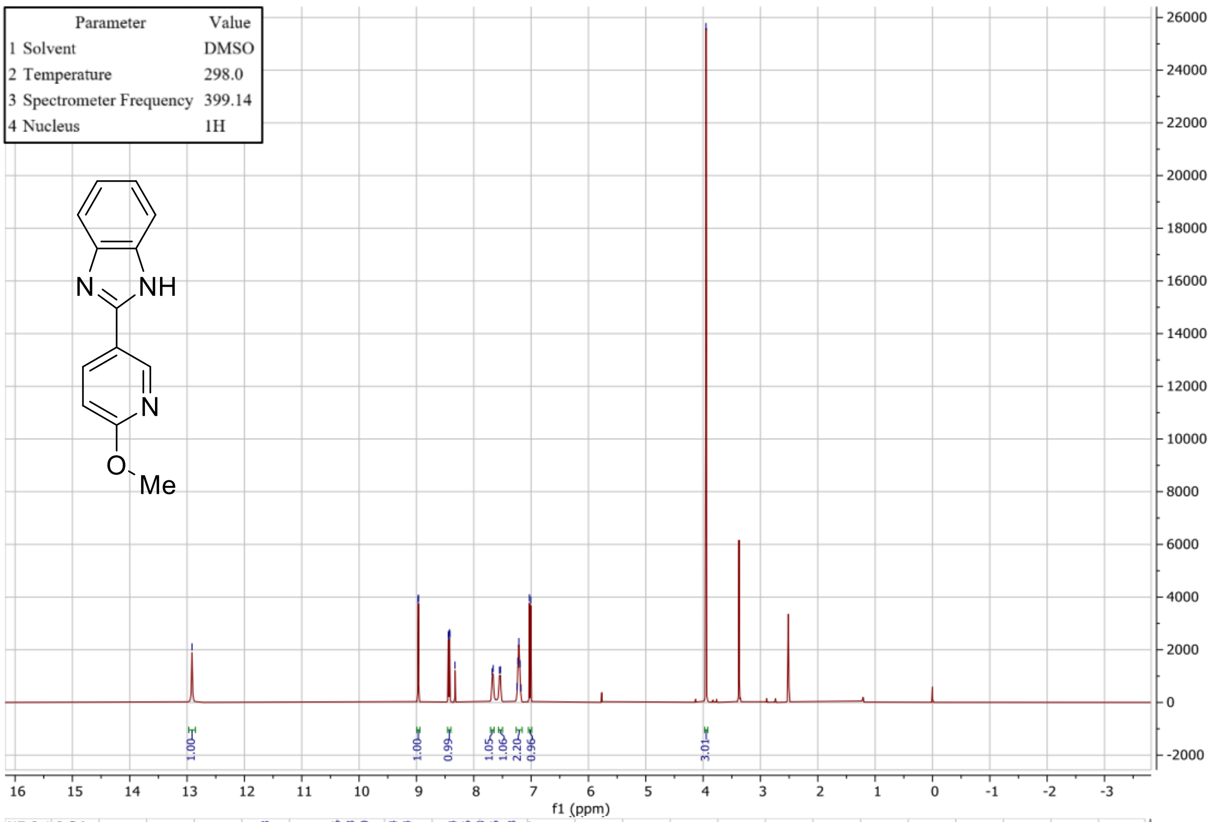

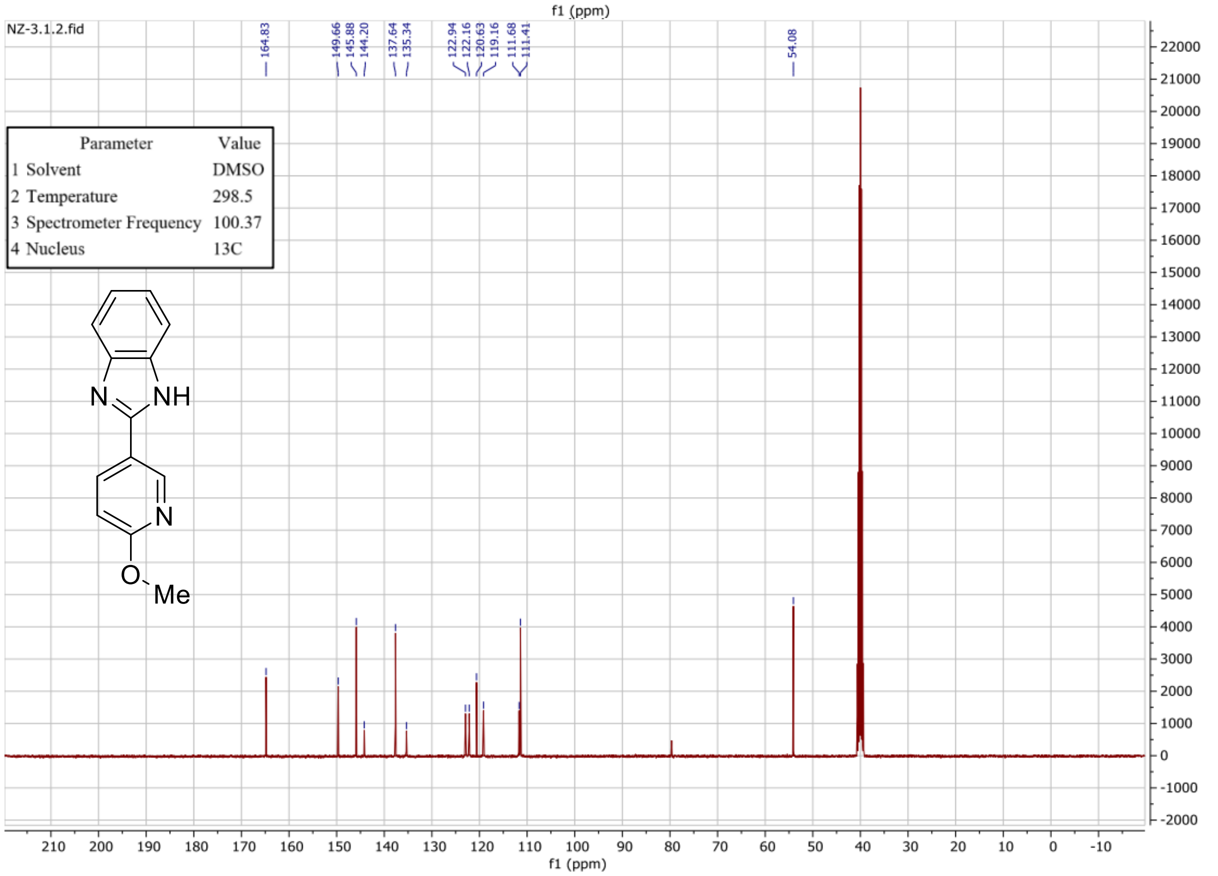

SI Figure S46: ^{1}H- and ^{13}C{^{1}H}-NMR spectra of imidazole **91**.

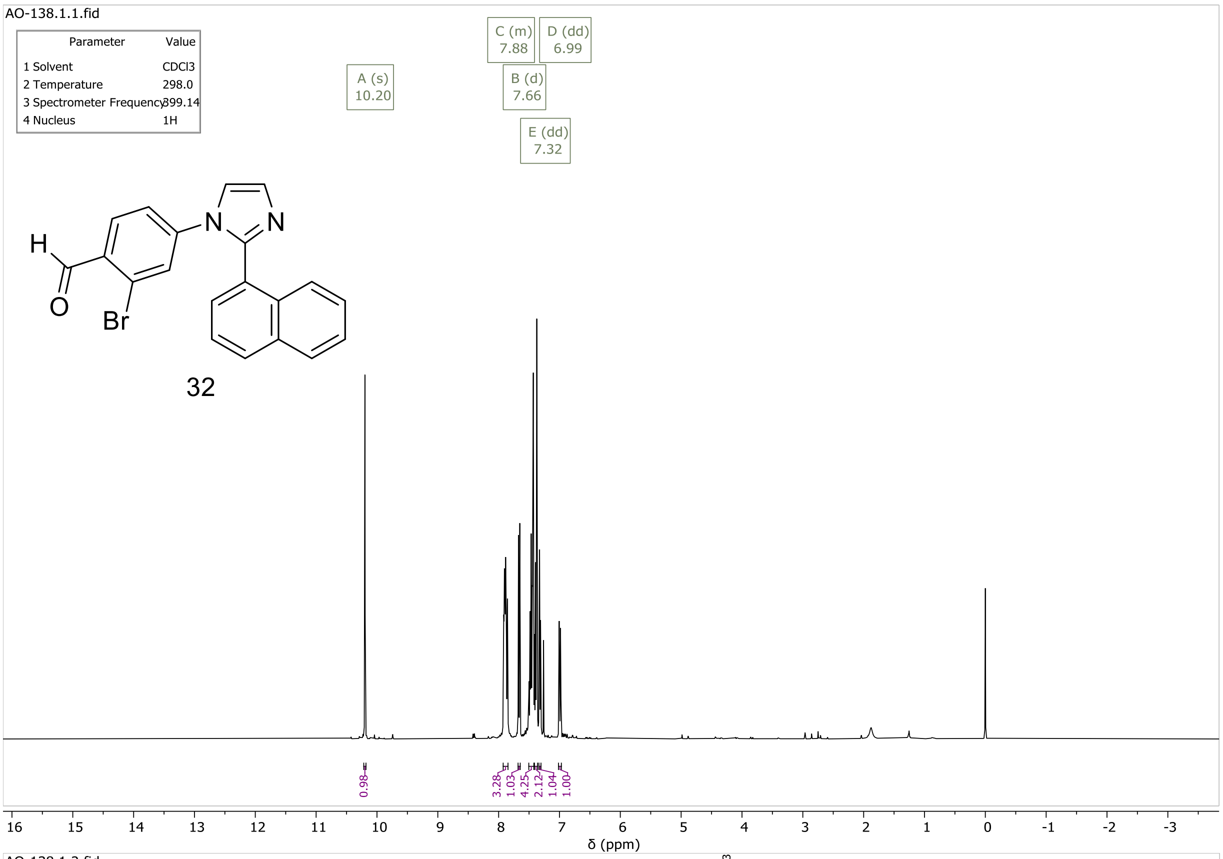

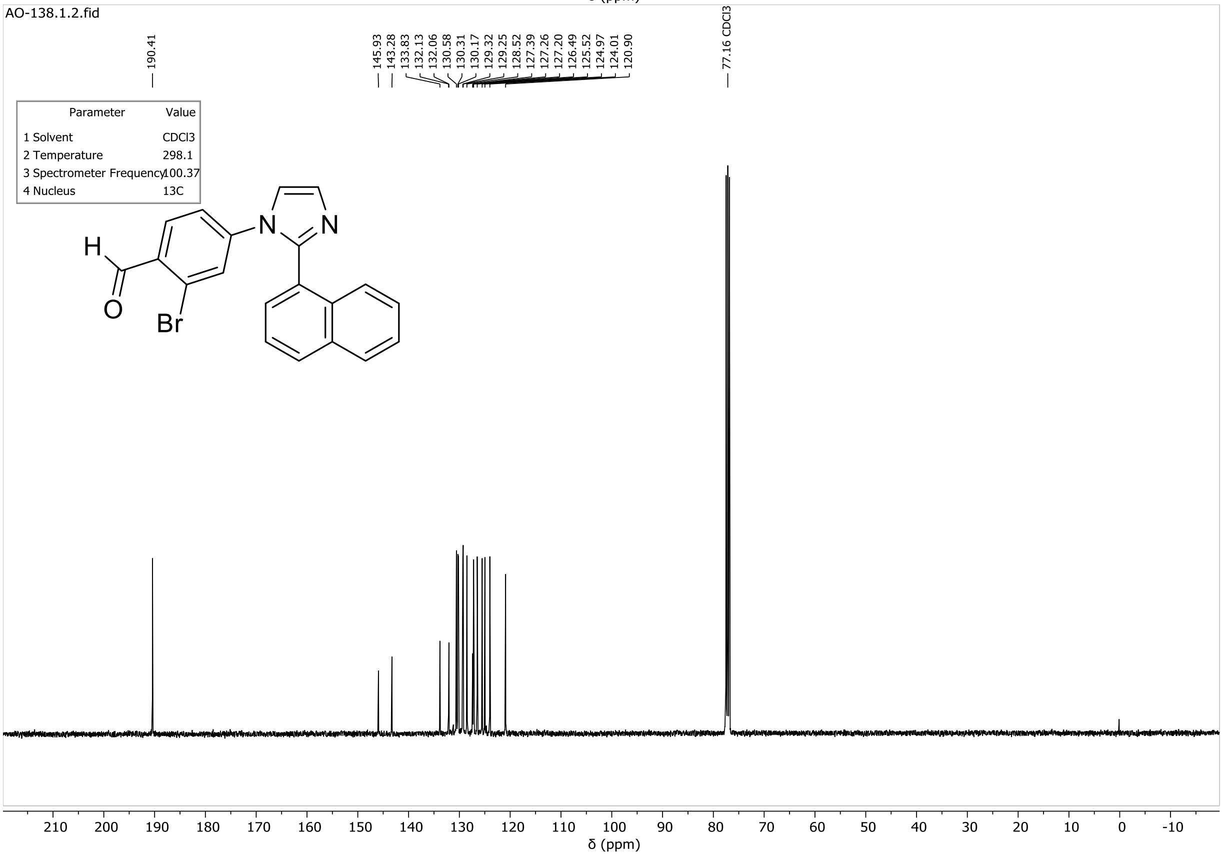

SI Figure S47: ^{1}H- and $^{13}C\{^{1}H\}$-NMR spectra of imidazole **32**.

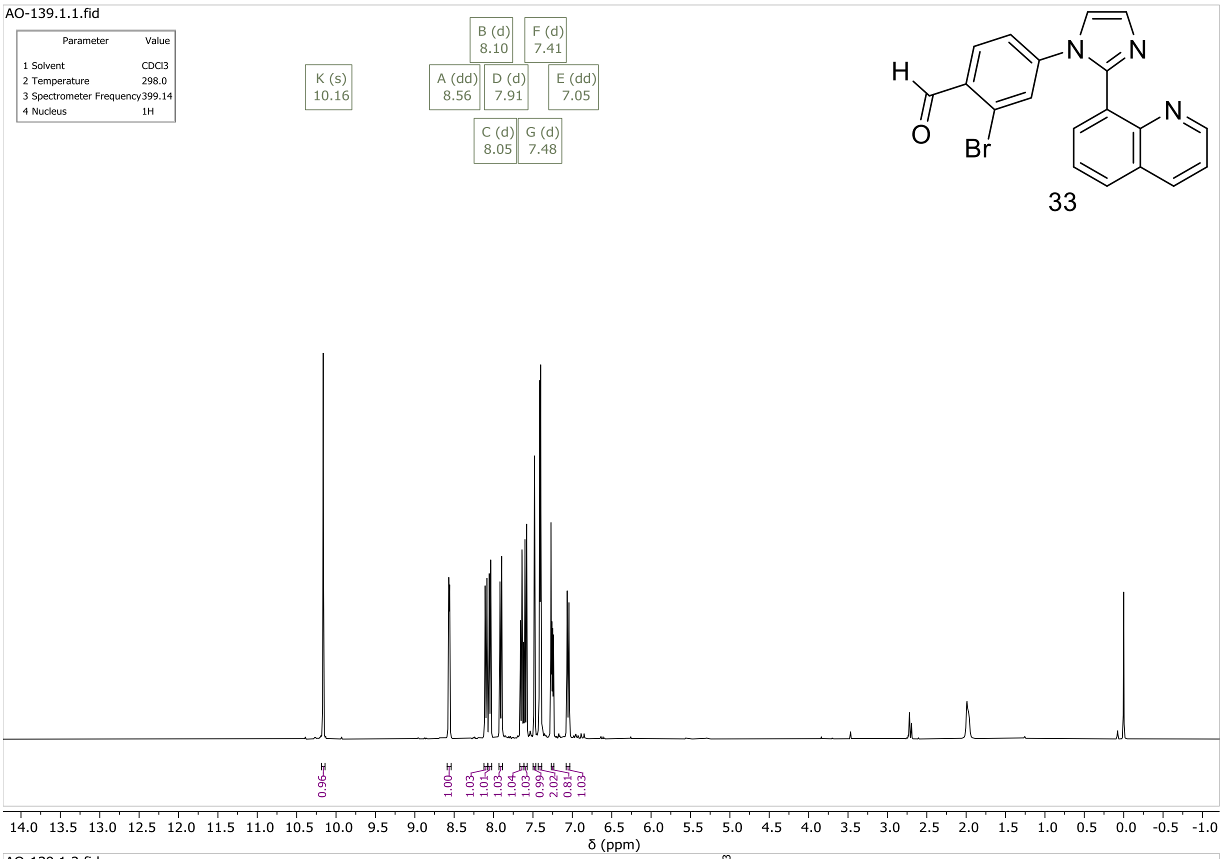

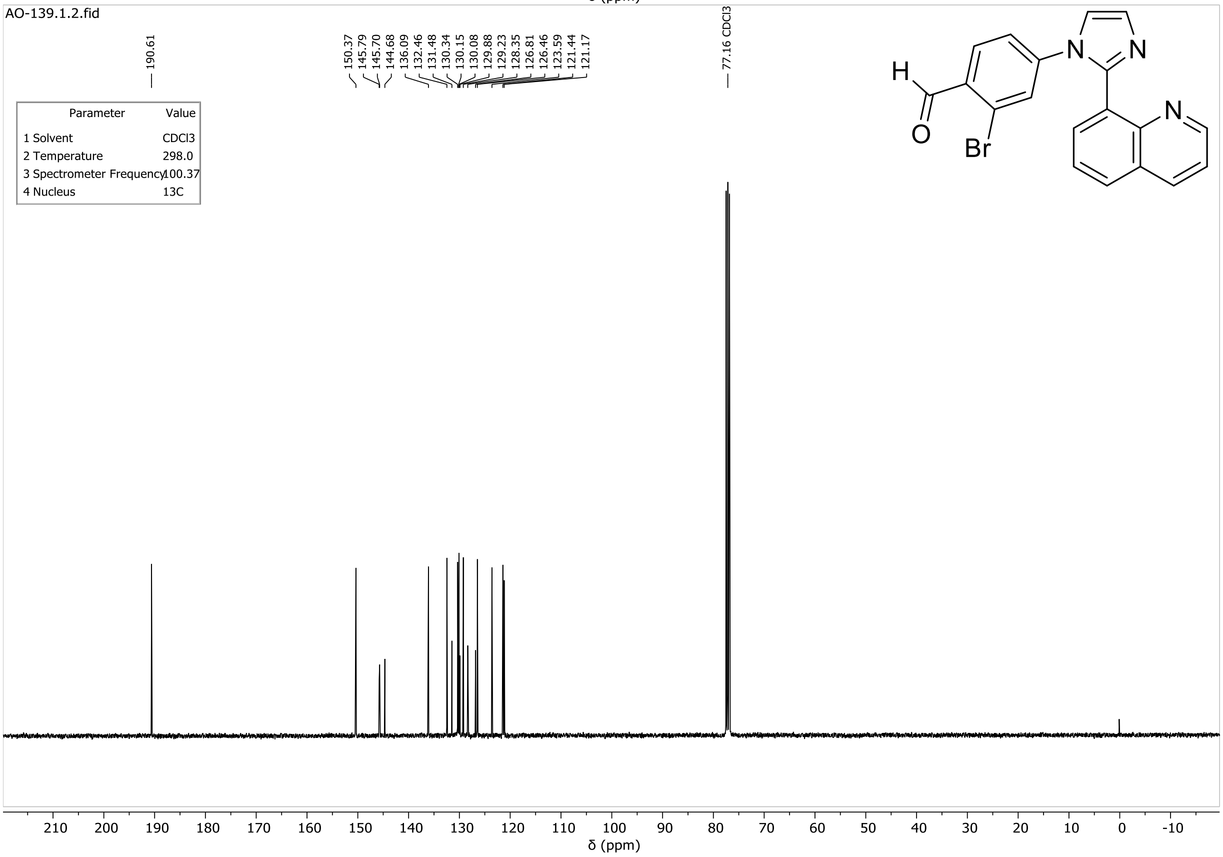

SI Figure S48: ^{1}H- and ^{13}C{^{1}H}-NMR spectra of imidazole **33.**

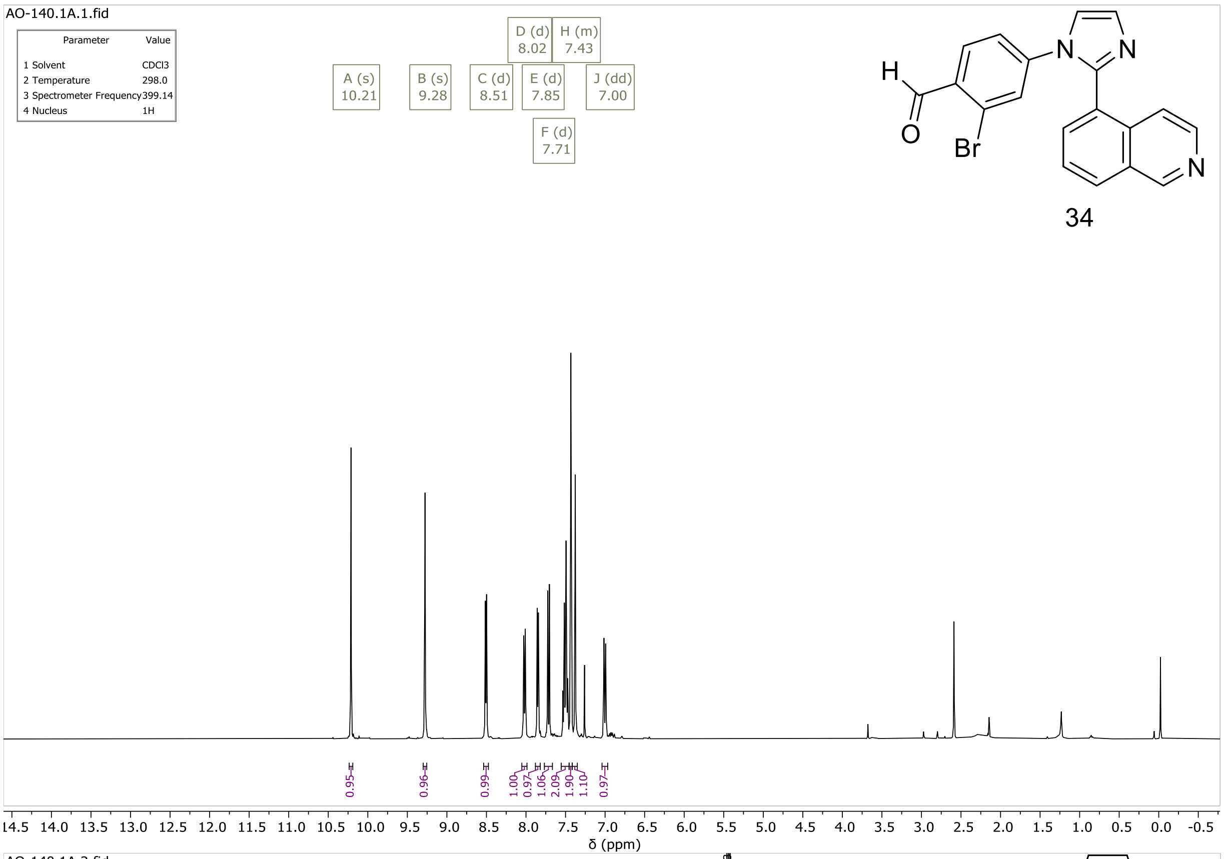

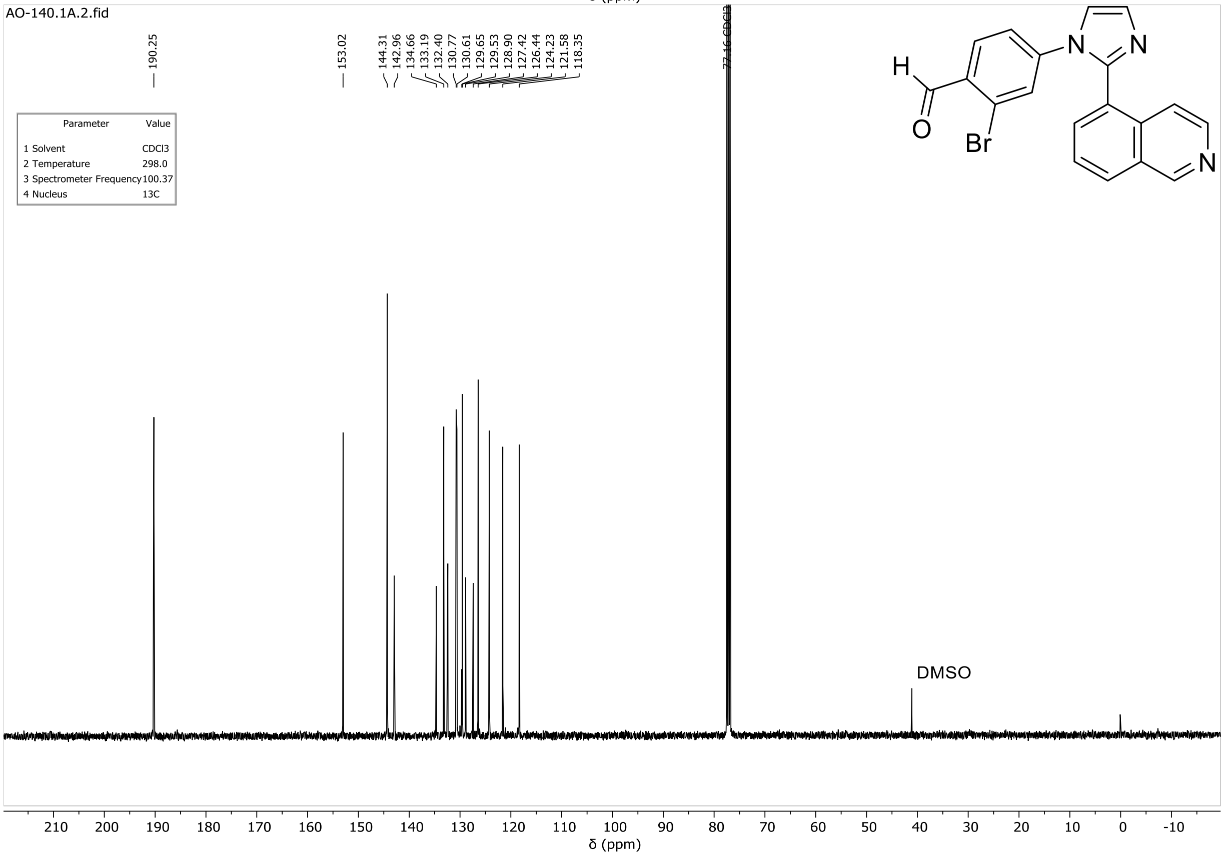

SI Figure S49: ^{1}H- and ^{13}C{^{1}H}-NMR spectra of imidazole **34**.

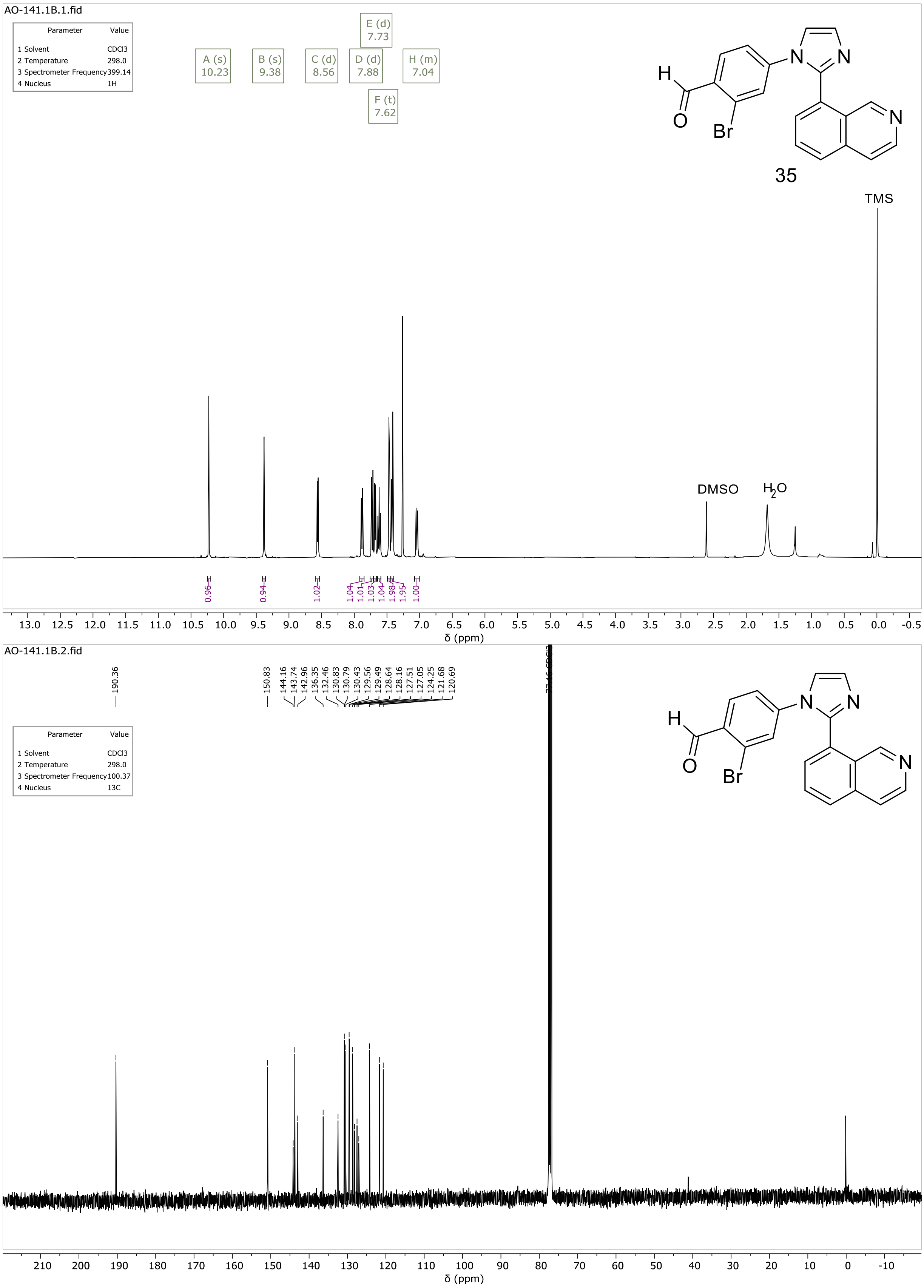

SI Figure S50: ^{1}H- and ^{13}C{^{1}H}-NMR spectra of imidazole **35.**

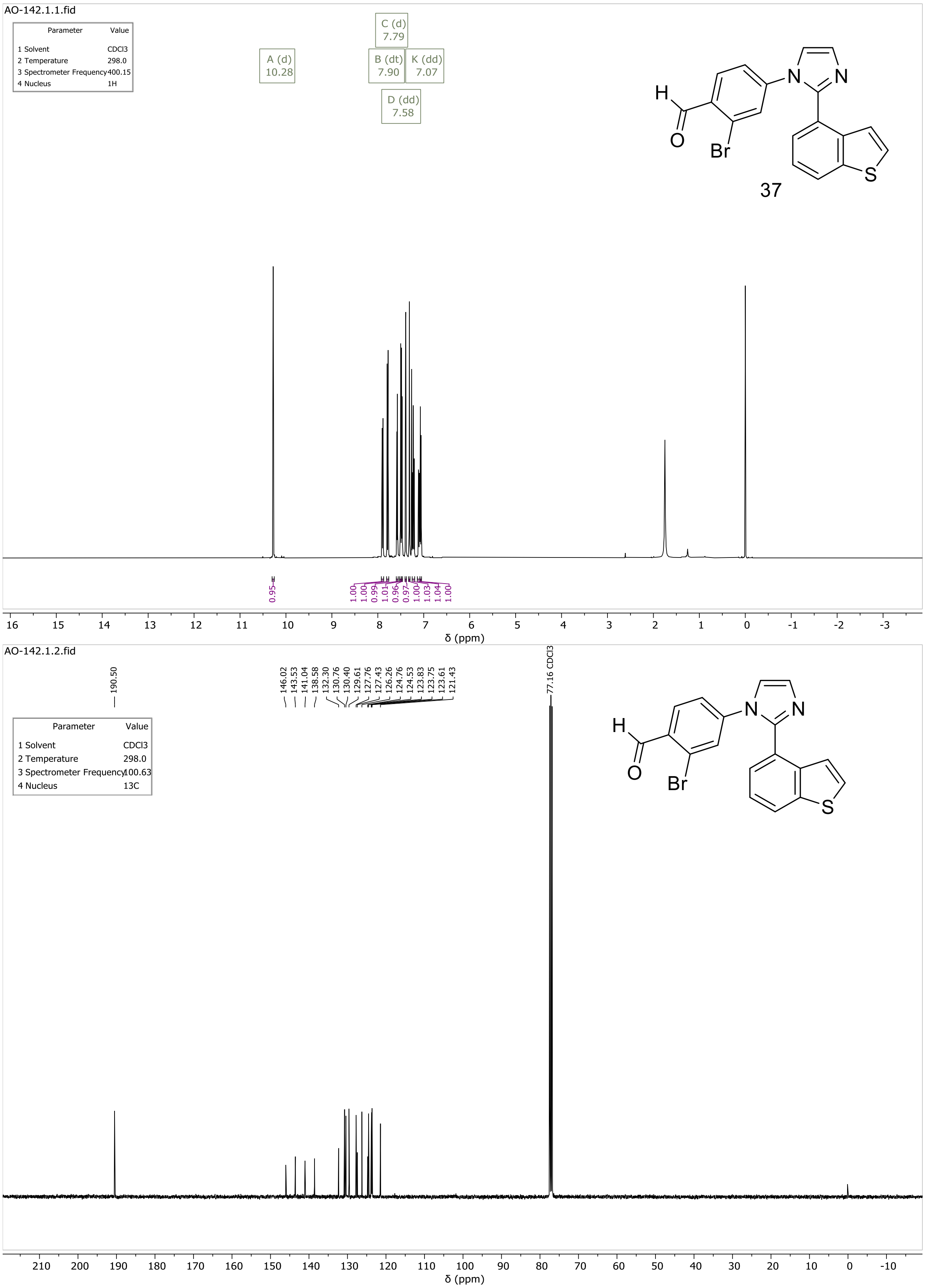

SI Figure S51: ^{1}H- and ^{13}C{^{1}H}-NMR spectra of imidazole **37**.

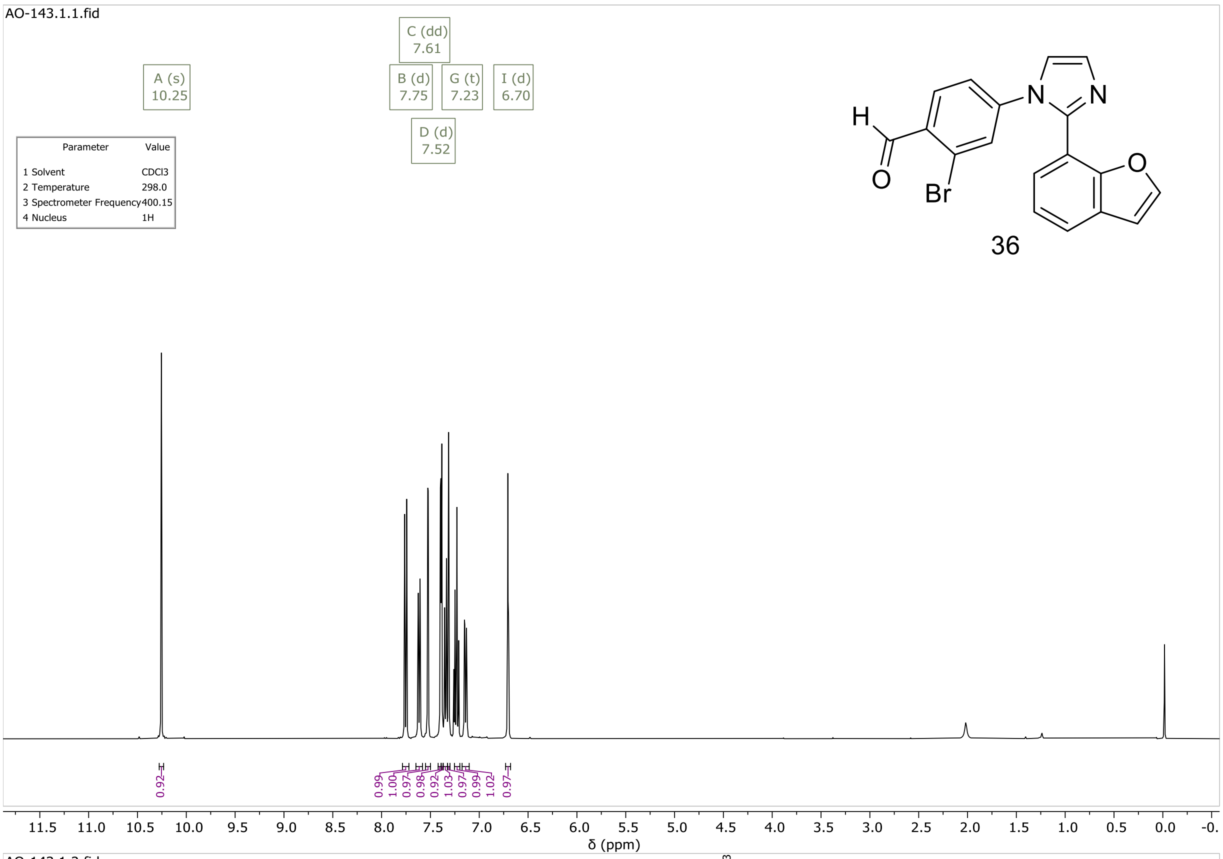

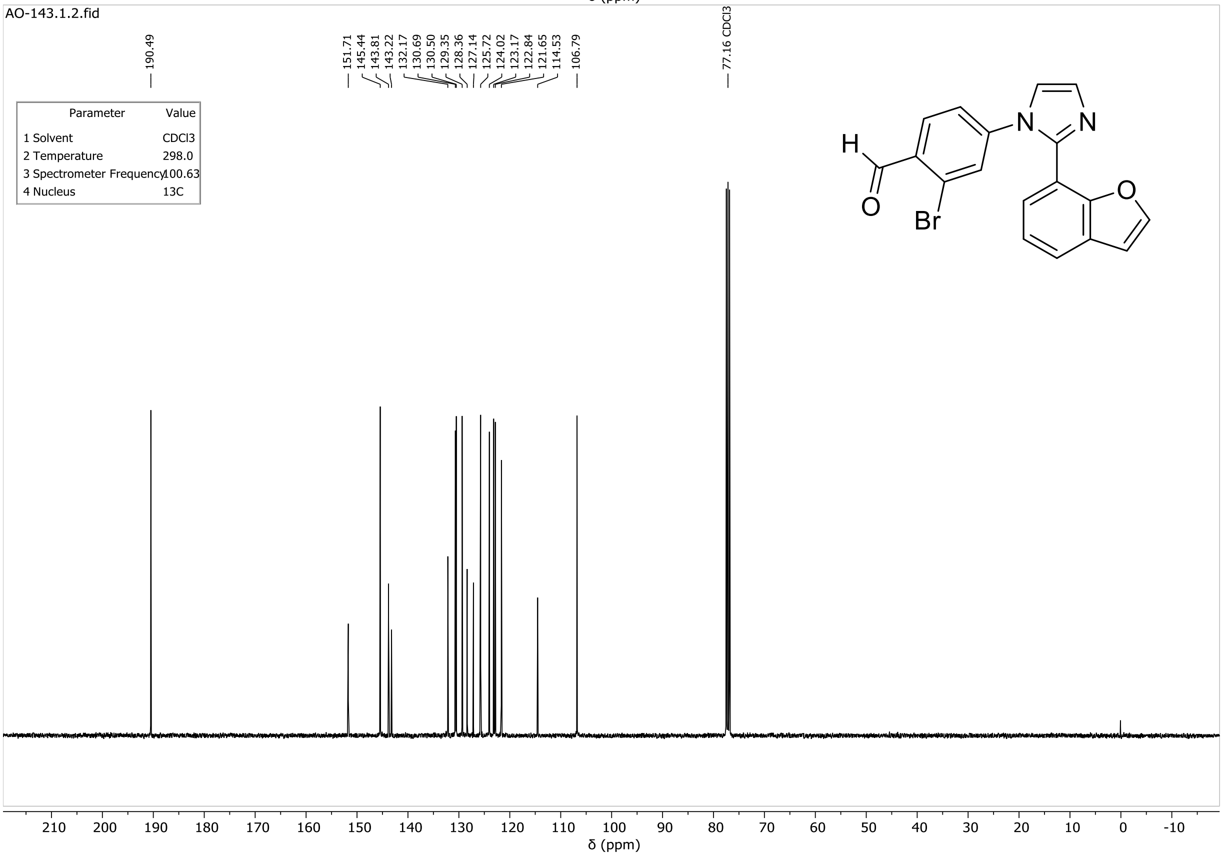

SI Figure S52: ^{1}H- and $^{13}C\{^{1}H\}$-NMR spectra of imidazole **36**.

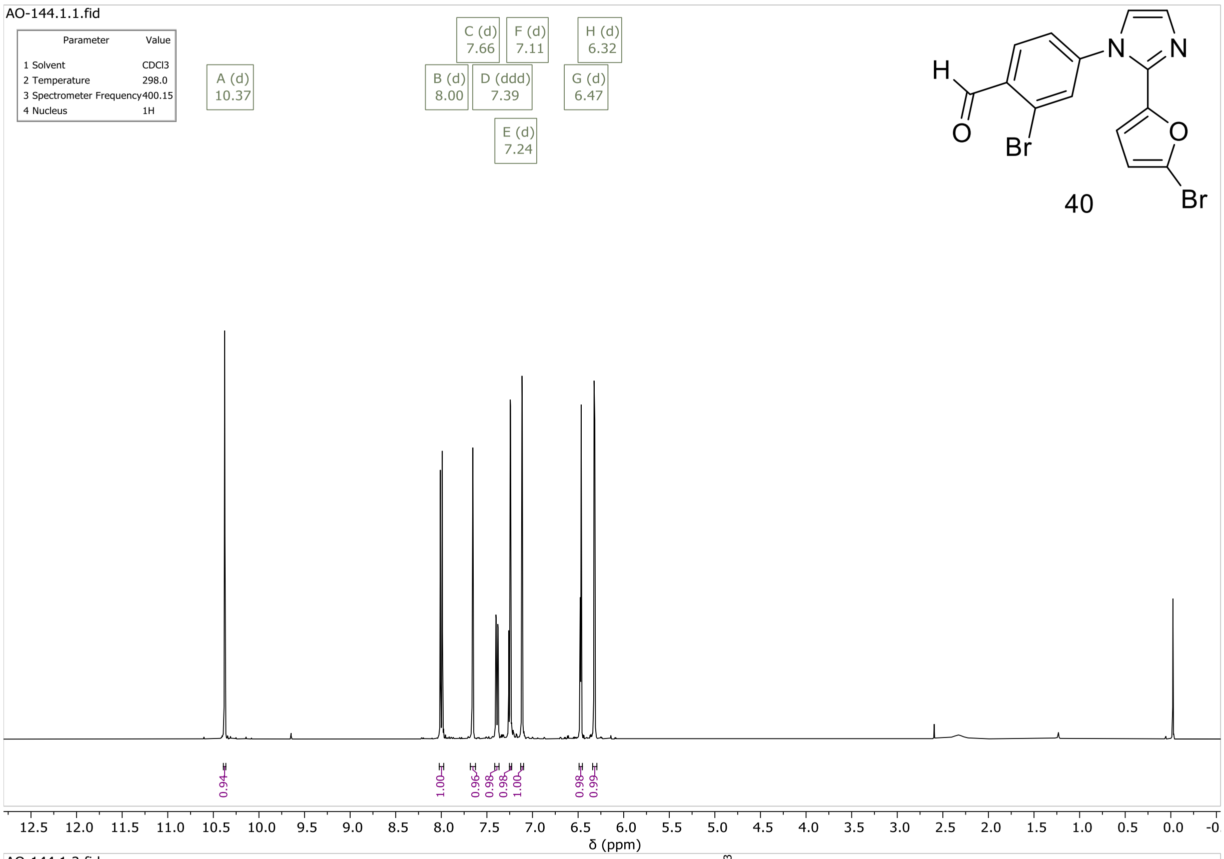

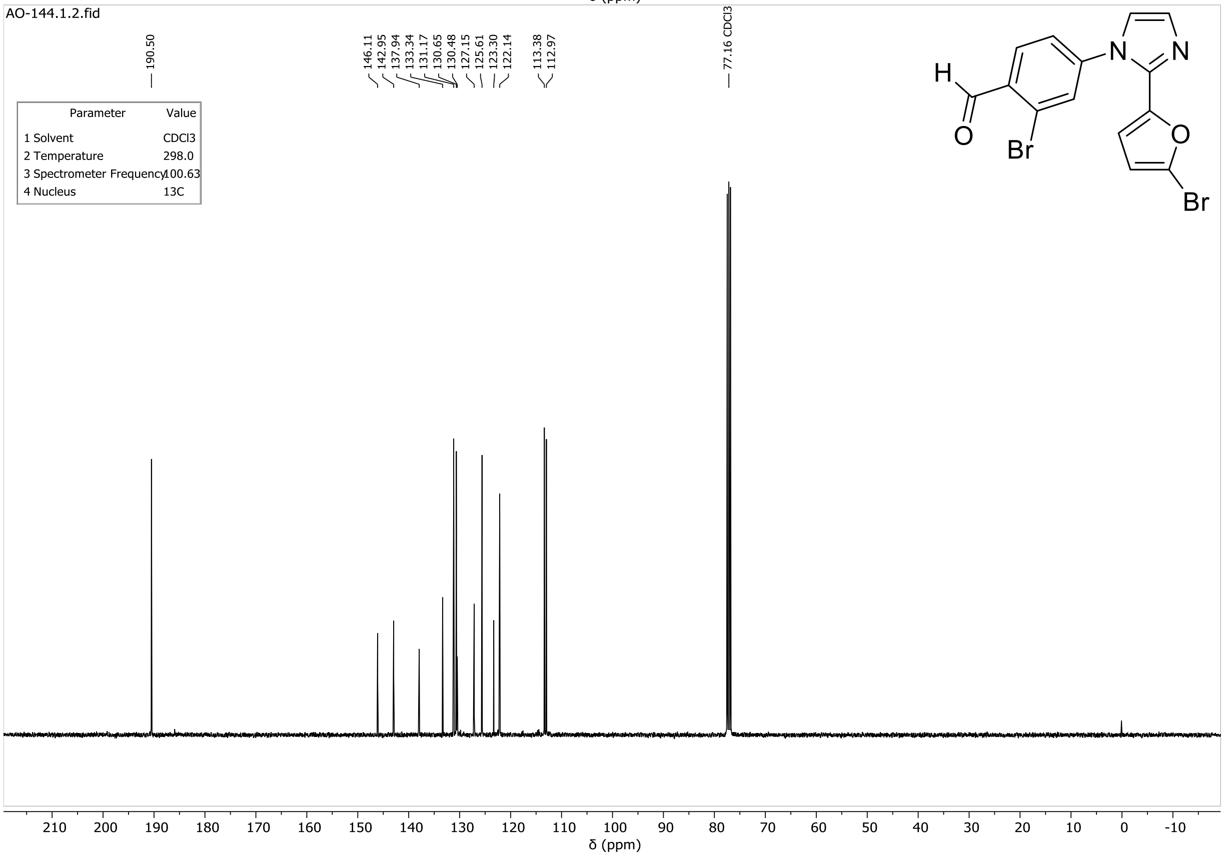

SI Figure S53: ^{1}H- and ^{13}C{^{1}H}-NMR spectra of imidazole **40**.

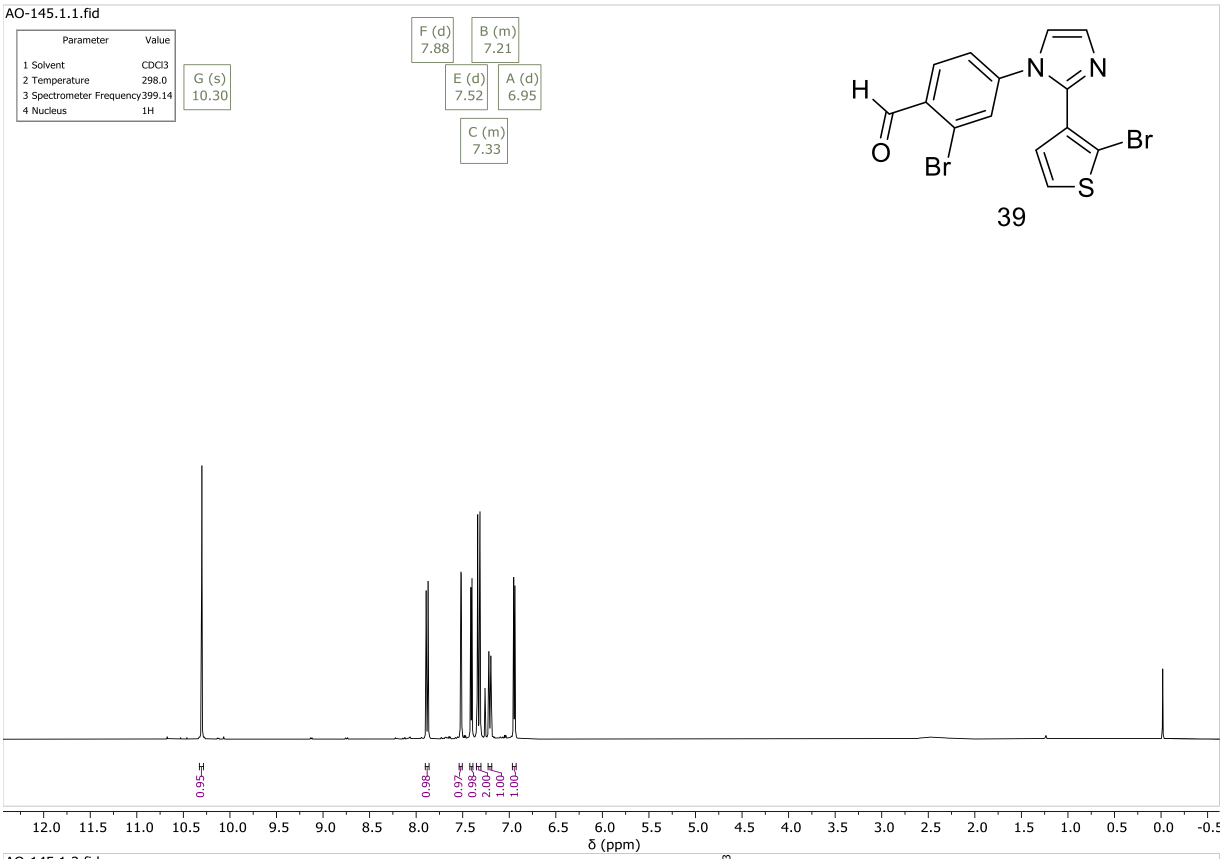

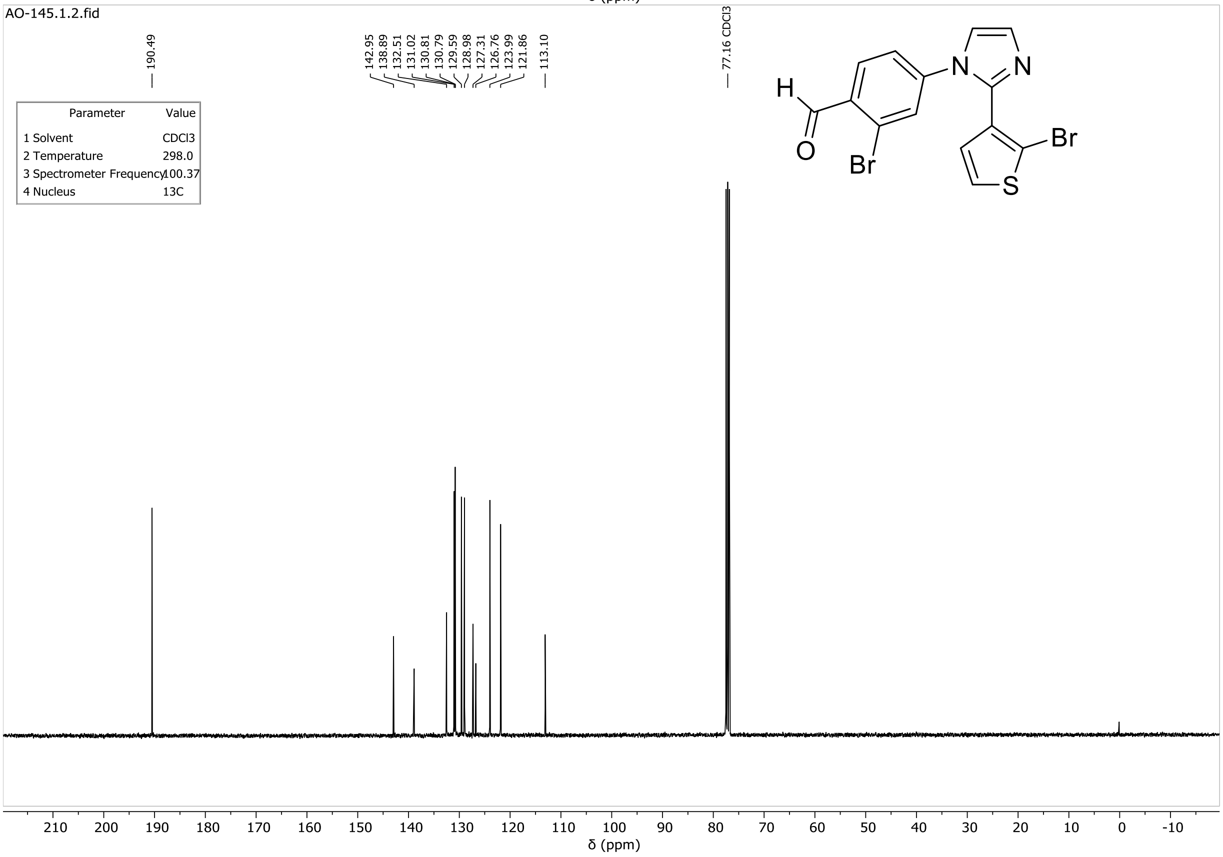

SI Figure S54: ^{1}H- and ^{13}C{^{1}H}-NMR spectra of imidazole **39**.

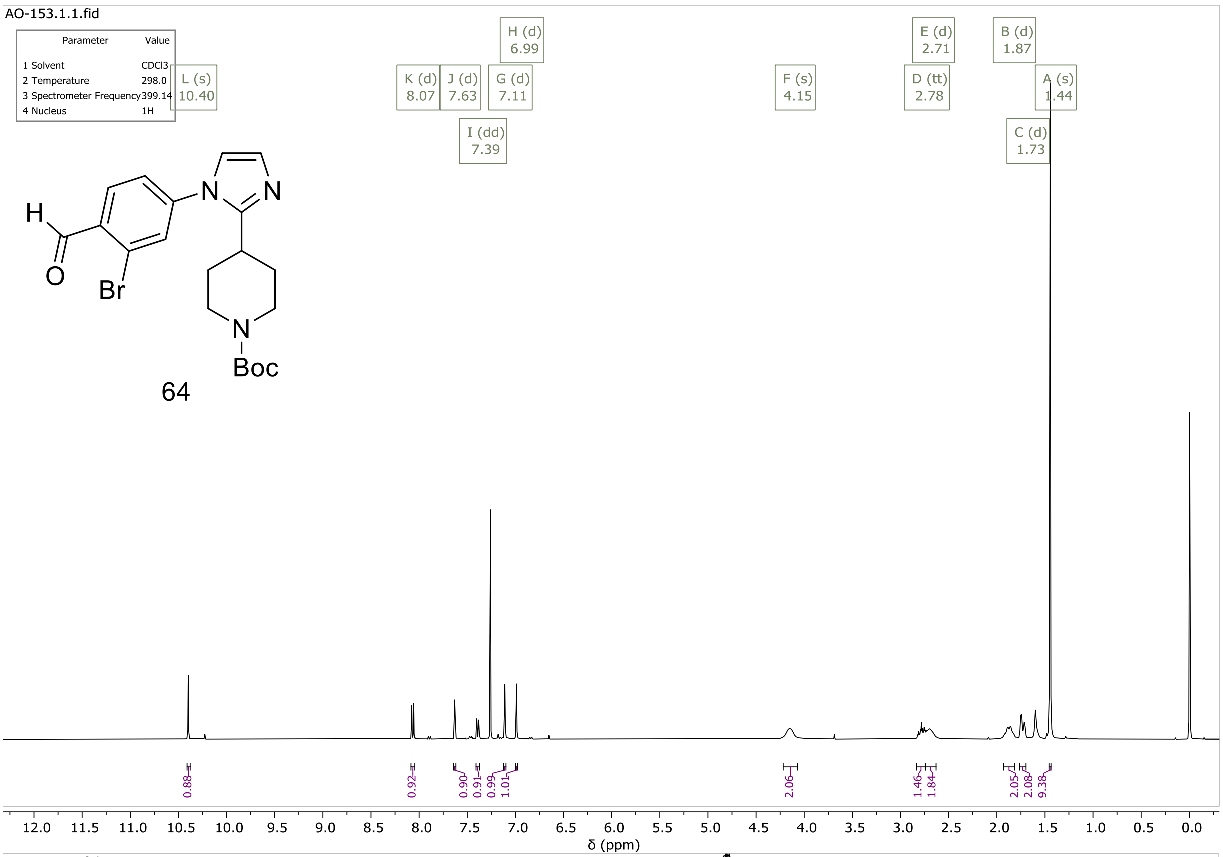

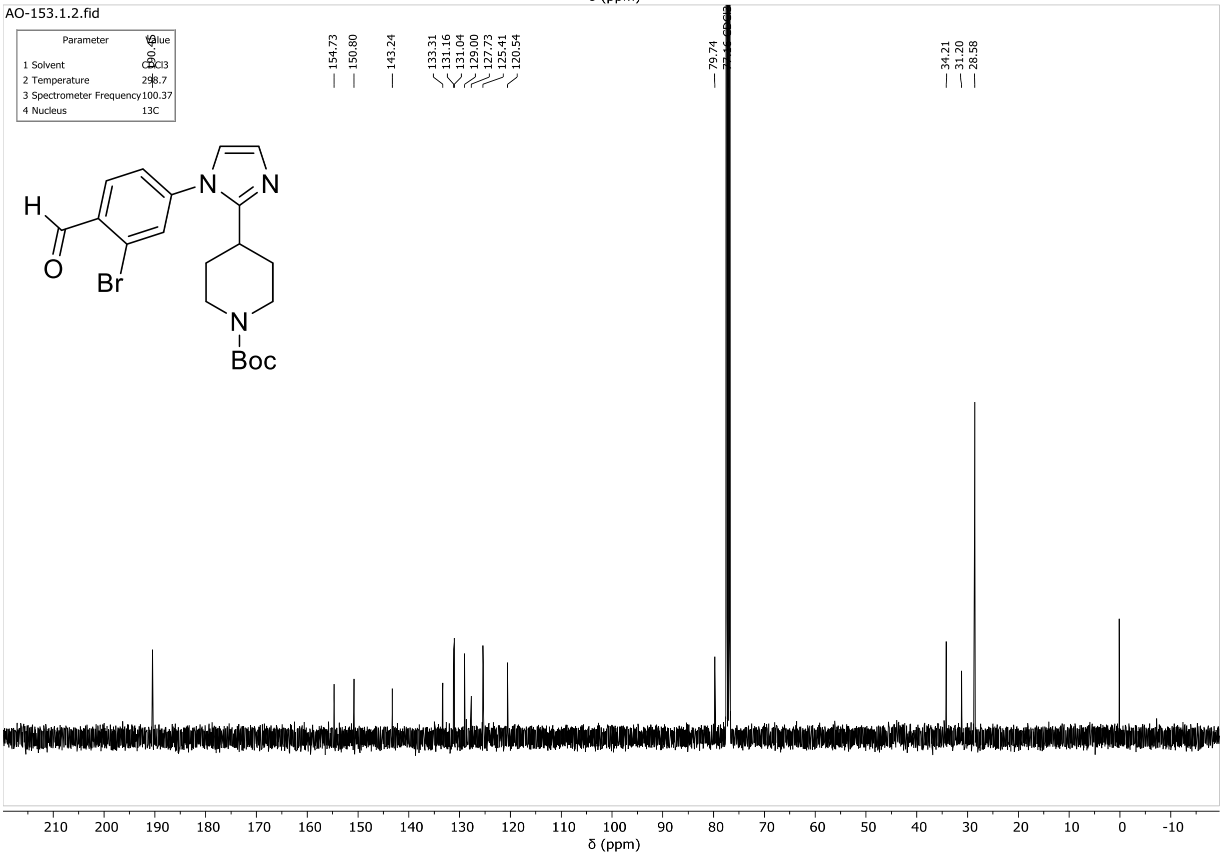

SI Figure S55: ^{1}H- and ^{13}C{^{1}H}-NMR spectra of imidazole **64**.

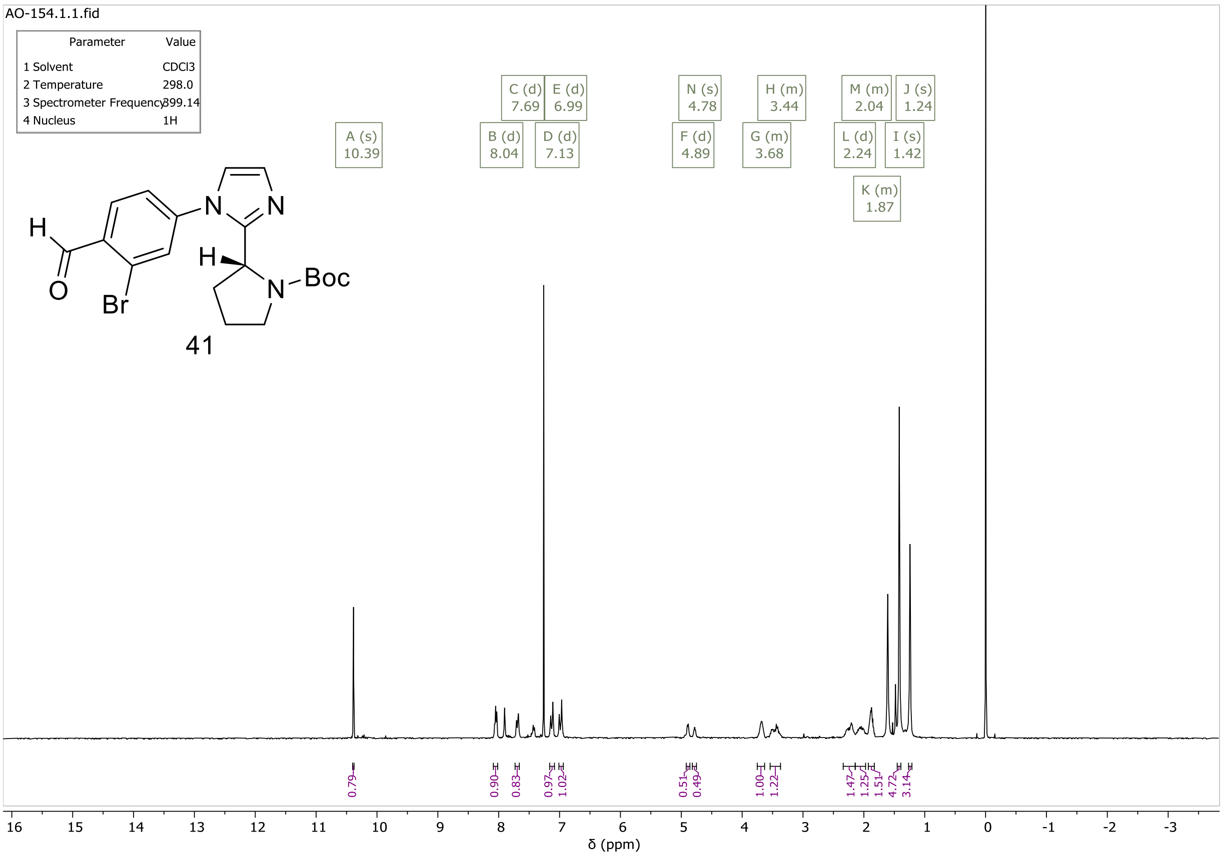

SI Figure S56: ^{1}H-NMR spectrum of imidazole **41**.

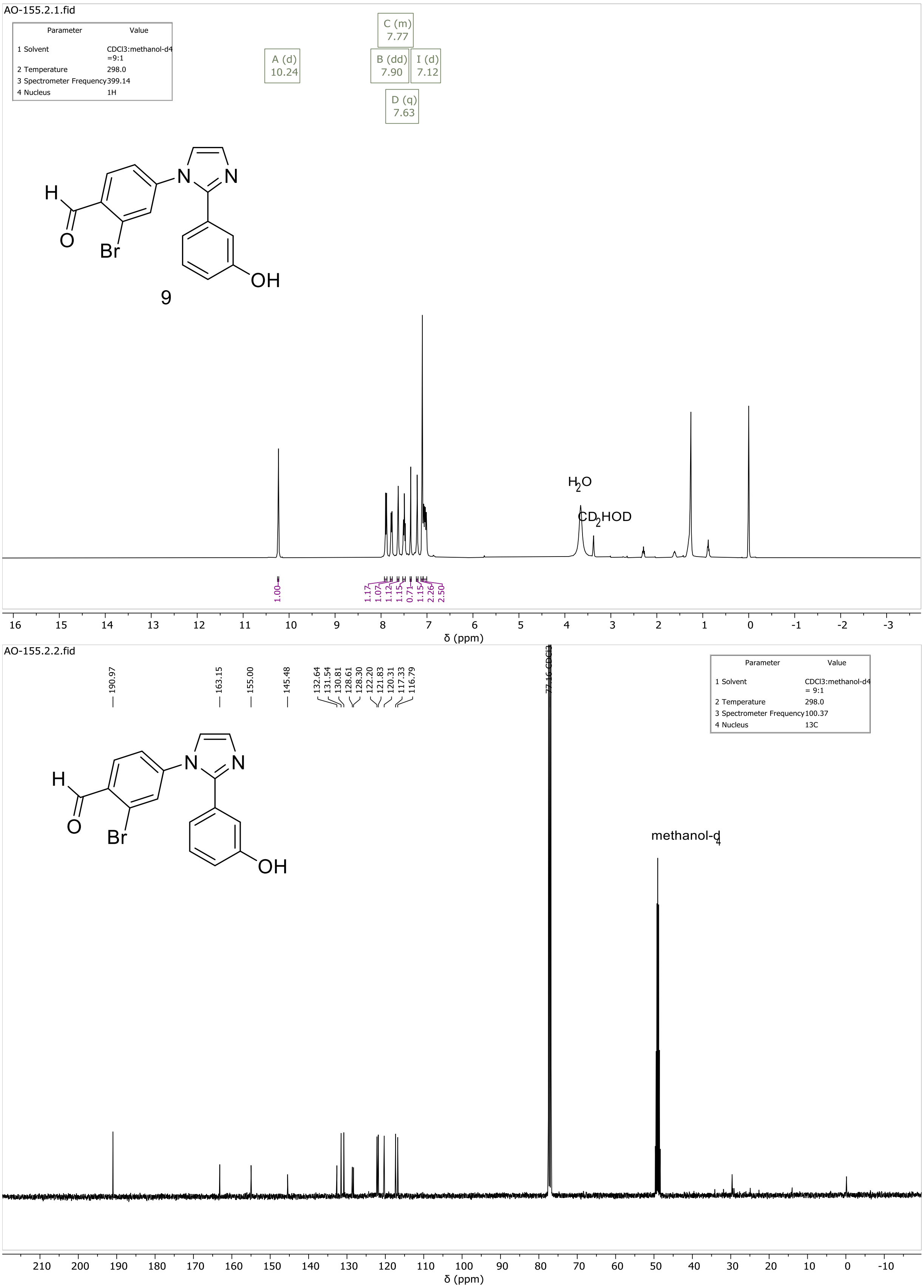

SI Figure S57: ^{1}H- and ^{13}C{^{1}H}-NMR spectra of imidazole **9.**

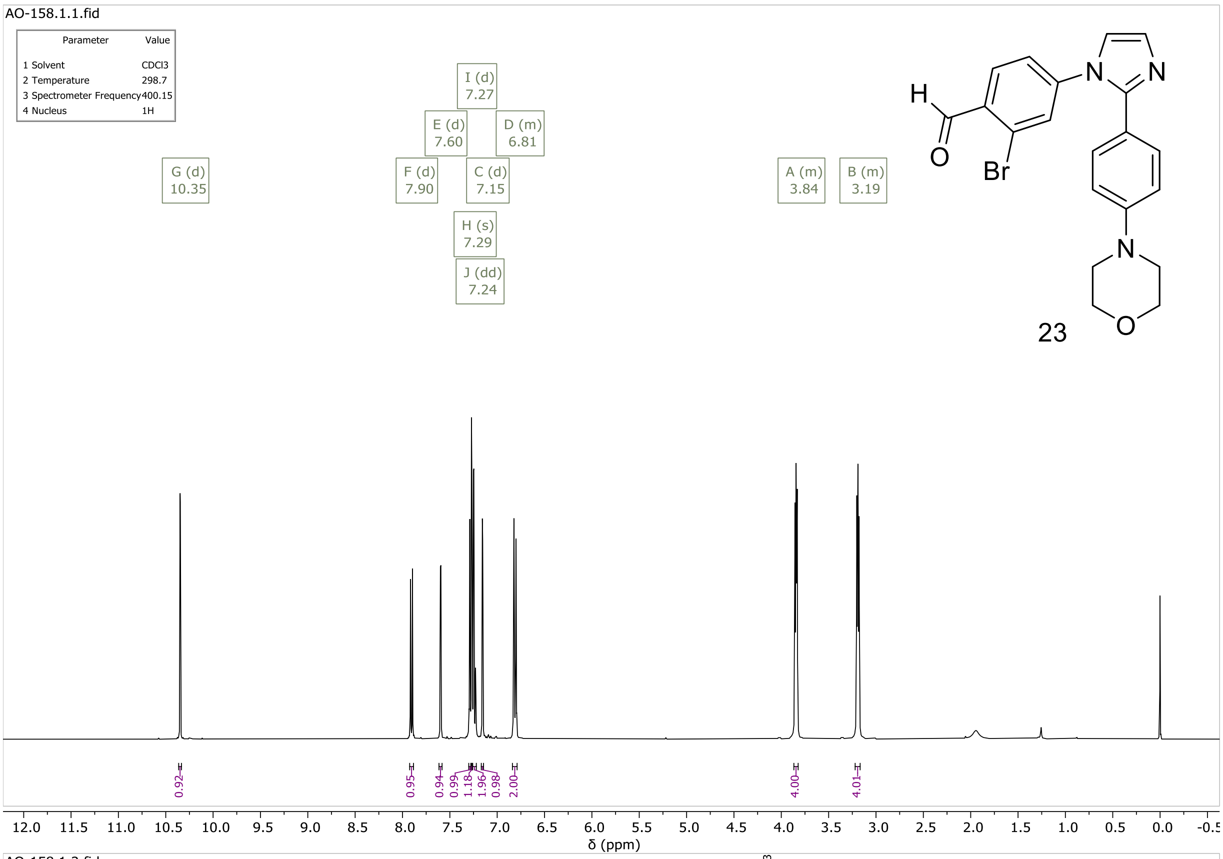

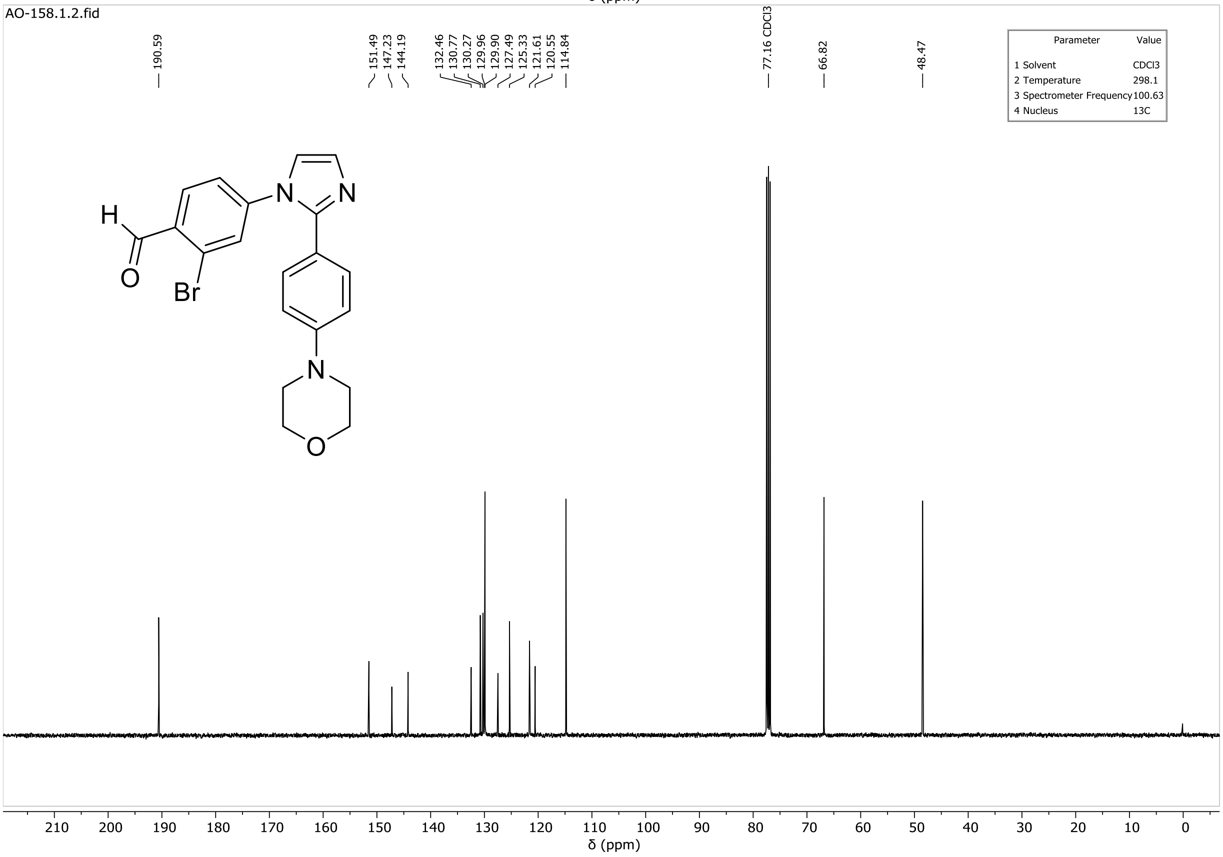

SI Figure S58: ^{1}H- and ^{13}C{^{1}H}-NMR spectra of imidazole **23.**

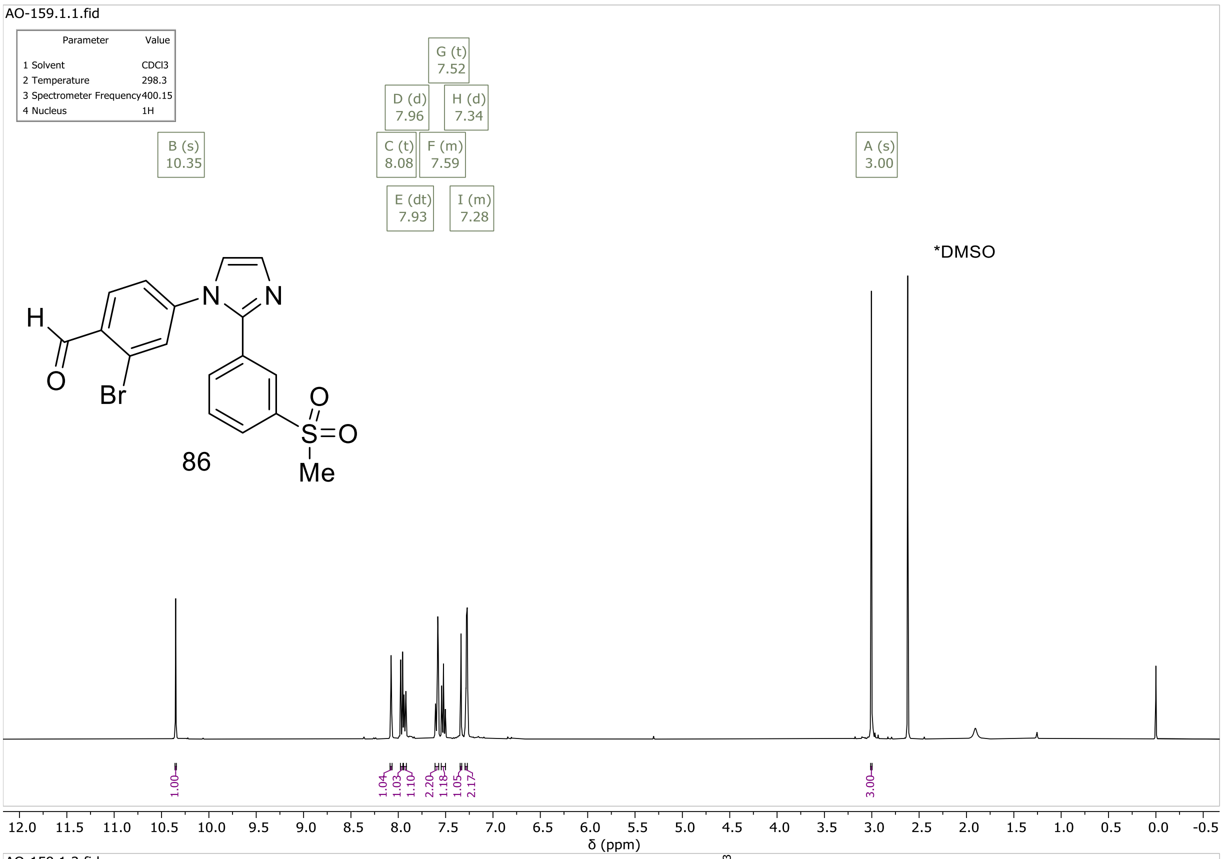

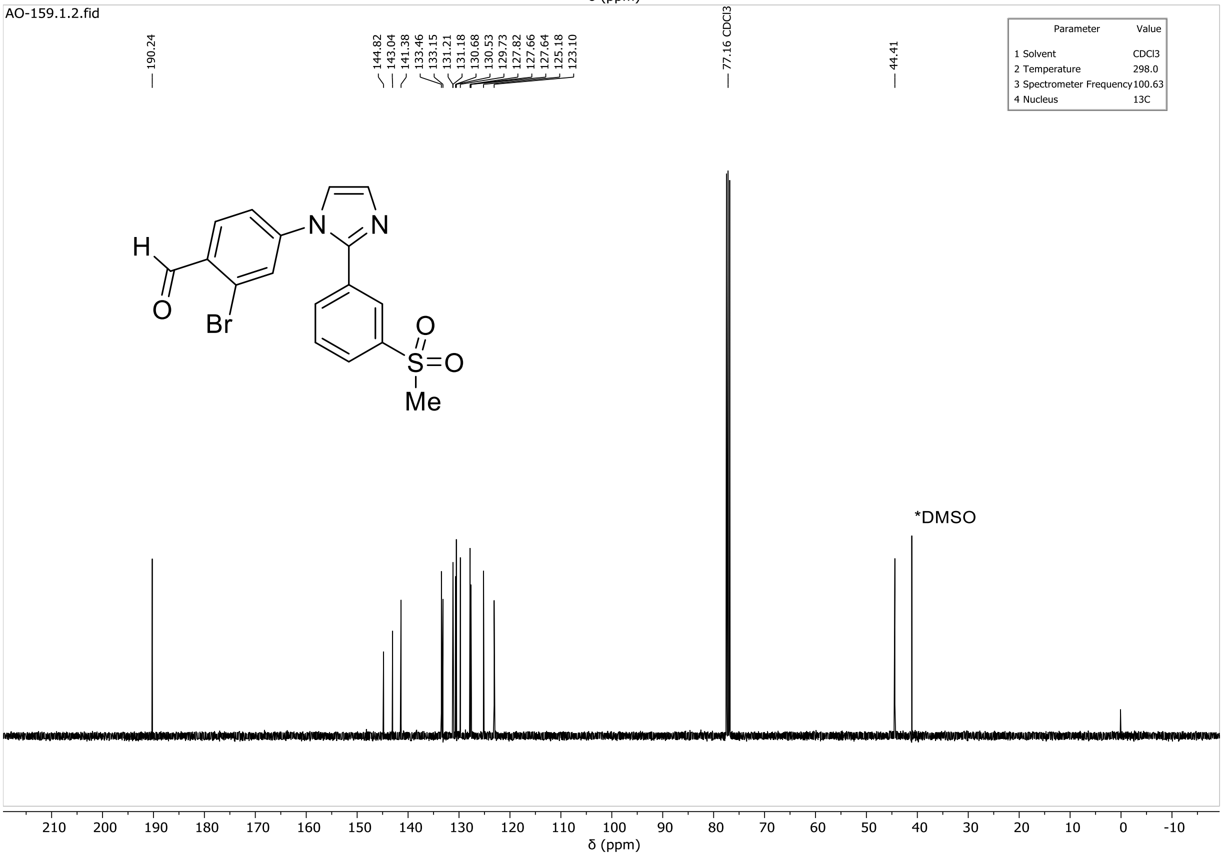

SI Figure S59: ^{1}H- and ^{13}C{^{1}H}-NMR spectra of imidazole **86.**

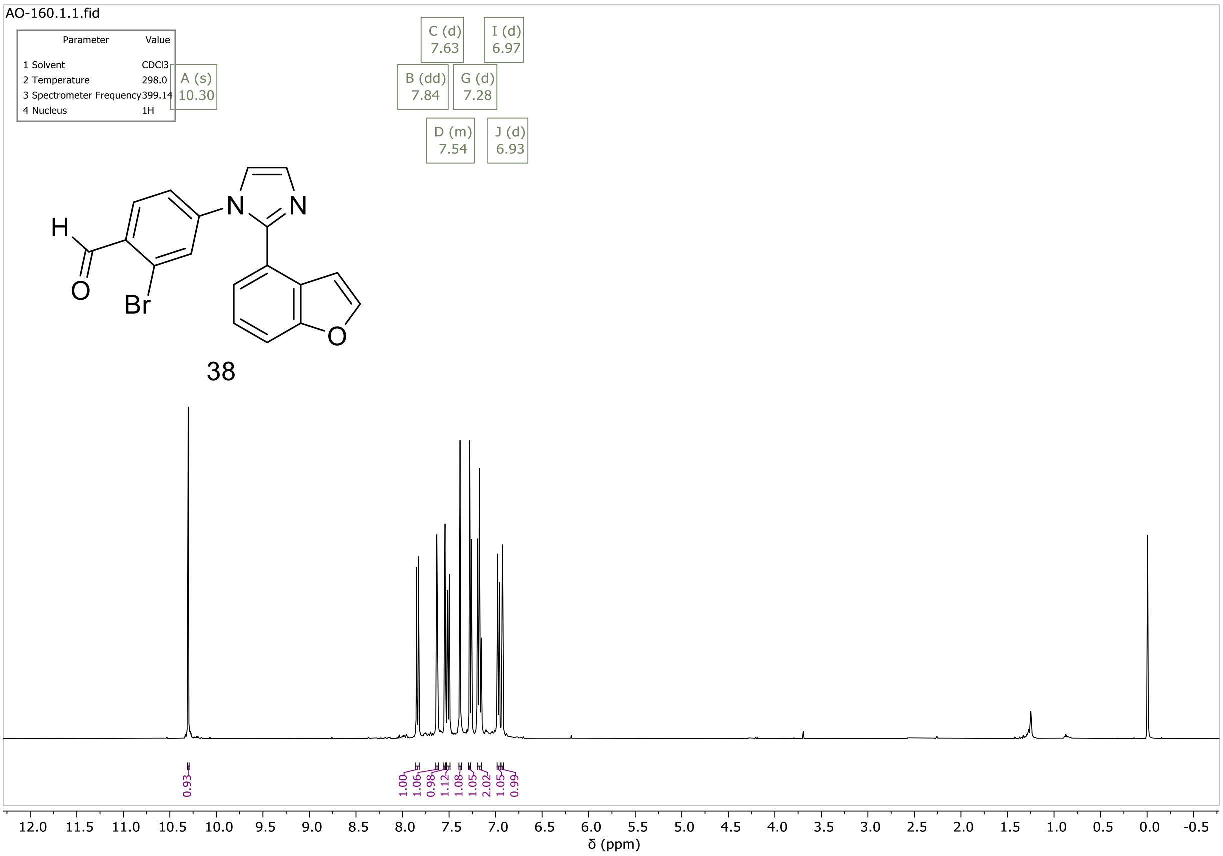

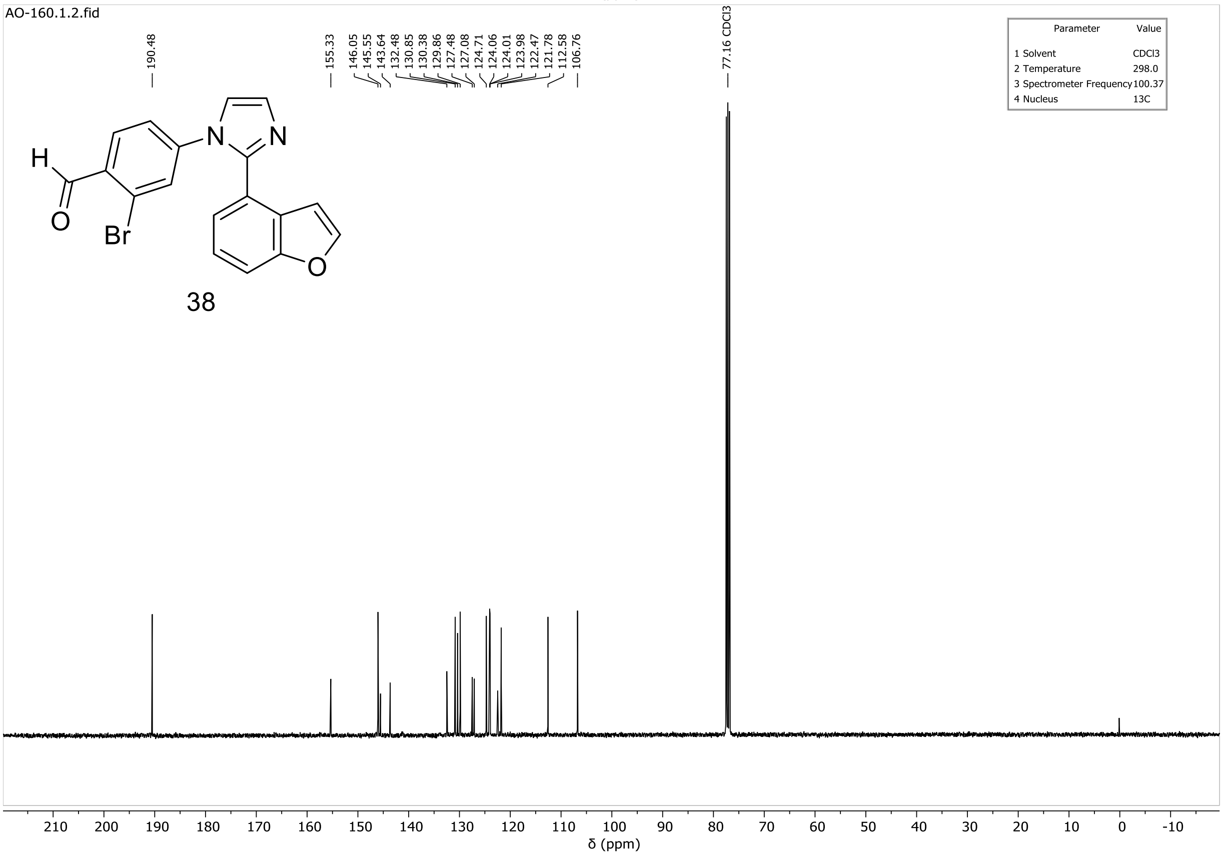

SI Figure S60: ^{1}H- and ^{13}C{^{1}H}-NMR spectra of imidazole **38**.

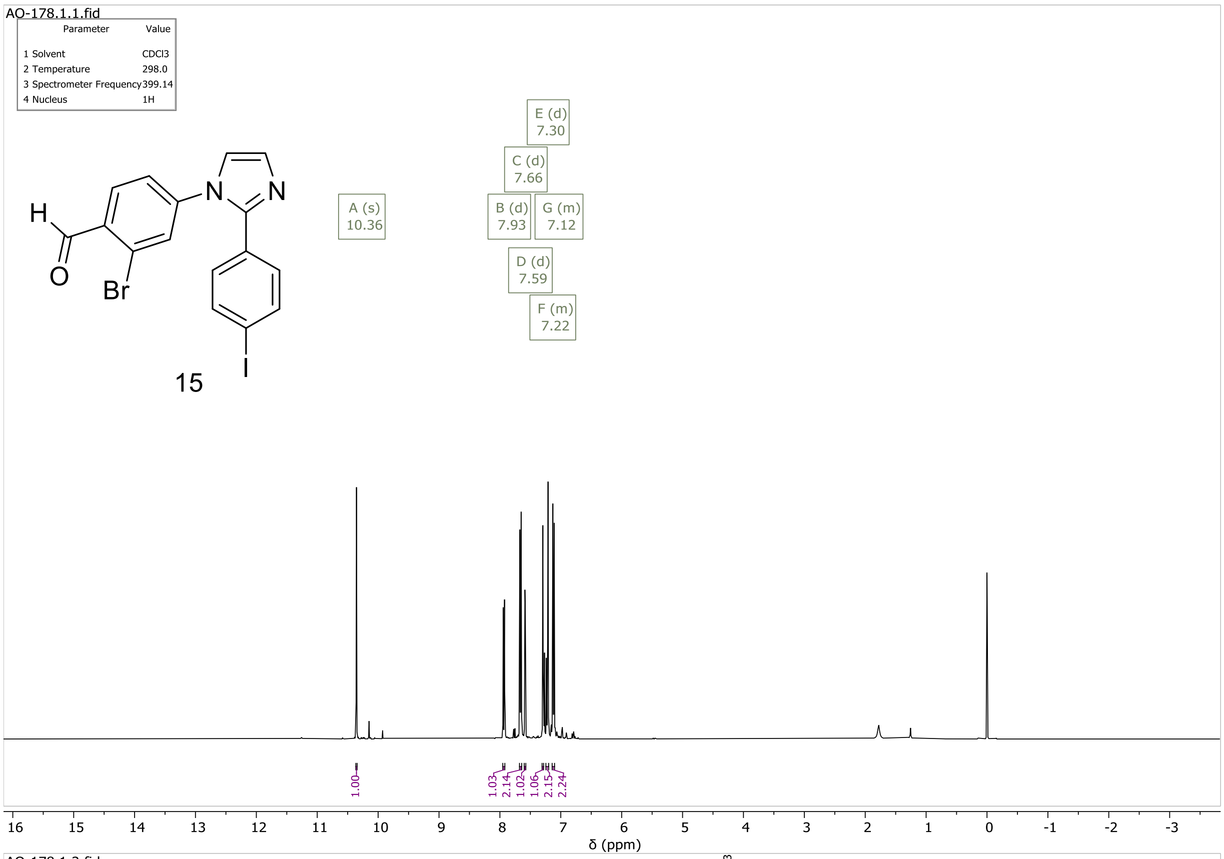

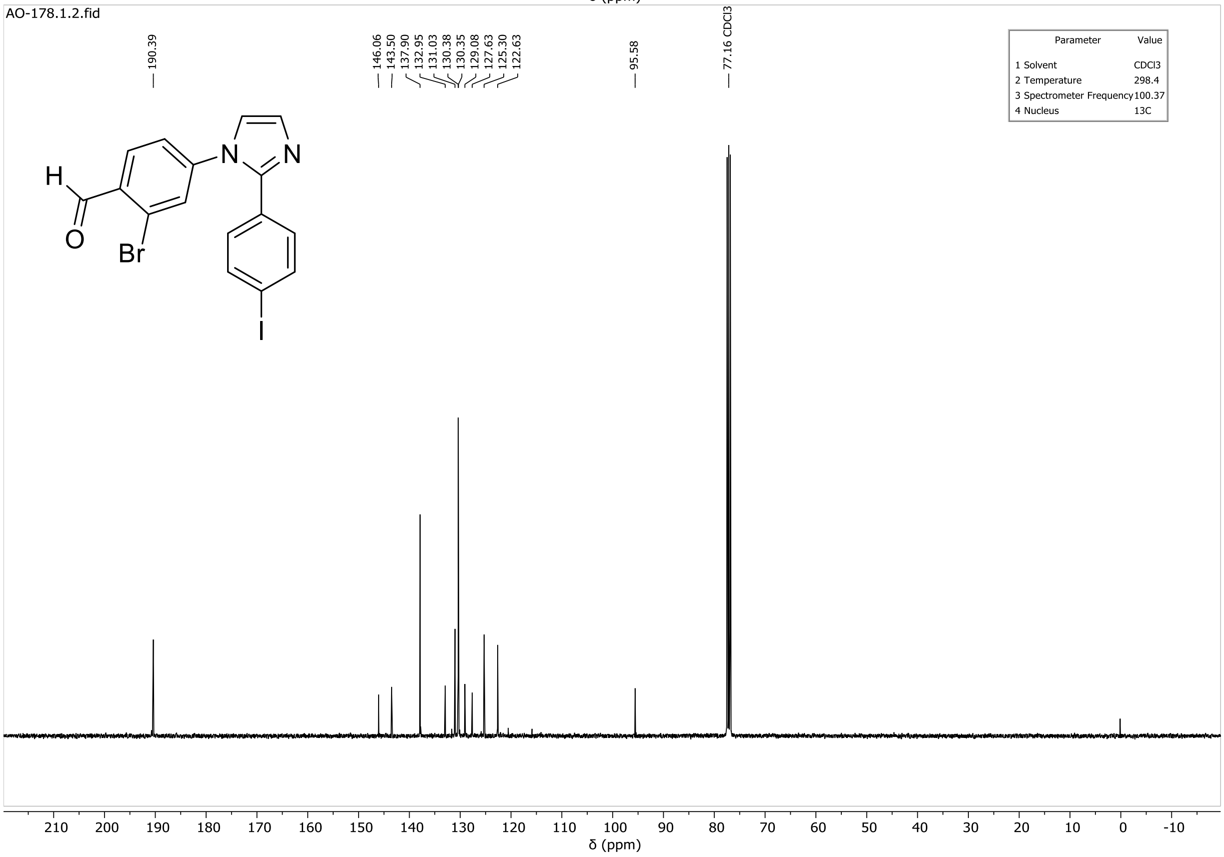

SI Figure S61: ^{1}H- and ^{13}C{^{1}H}-NMR spectra of imidazole **15.**

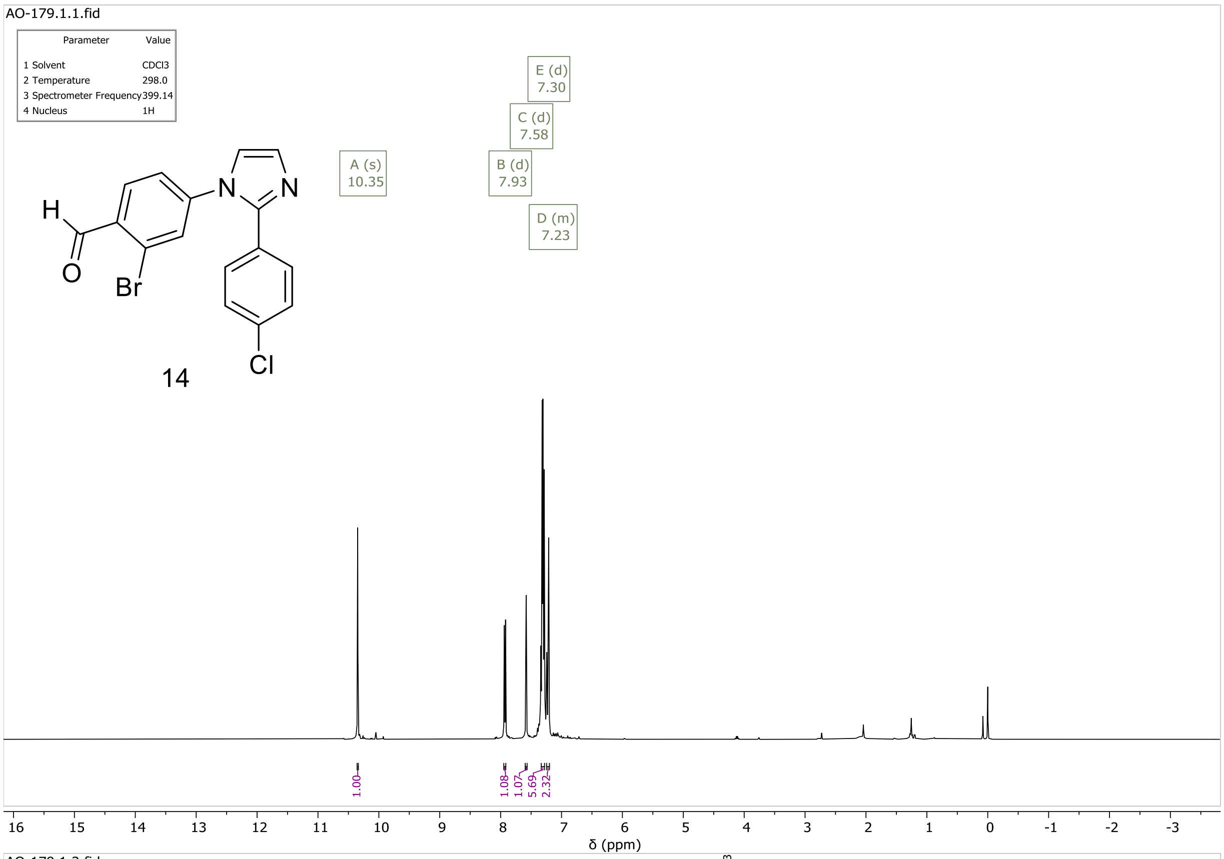

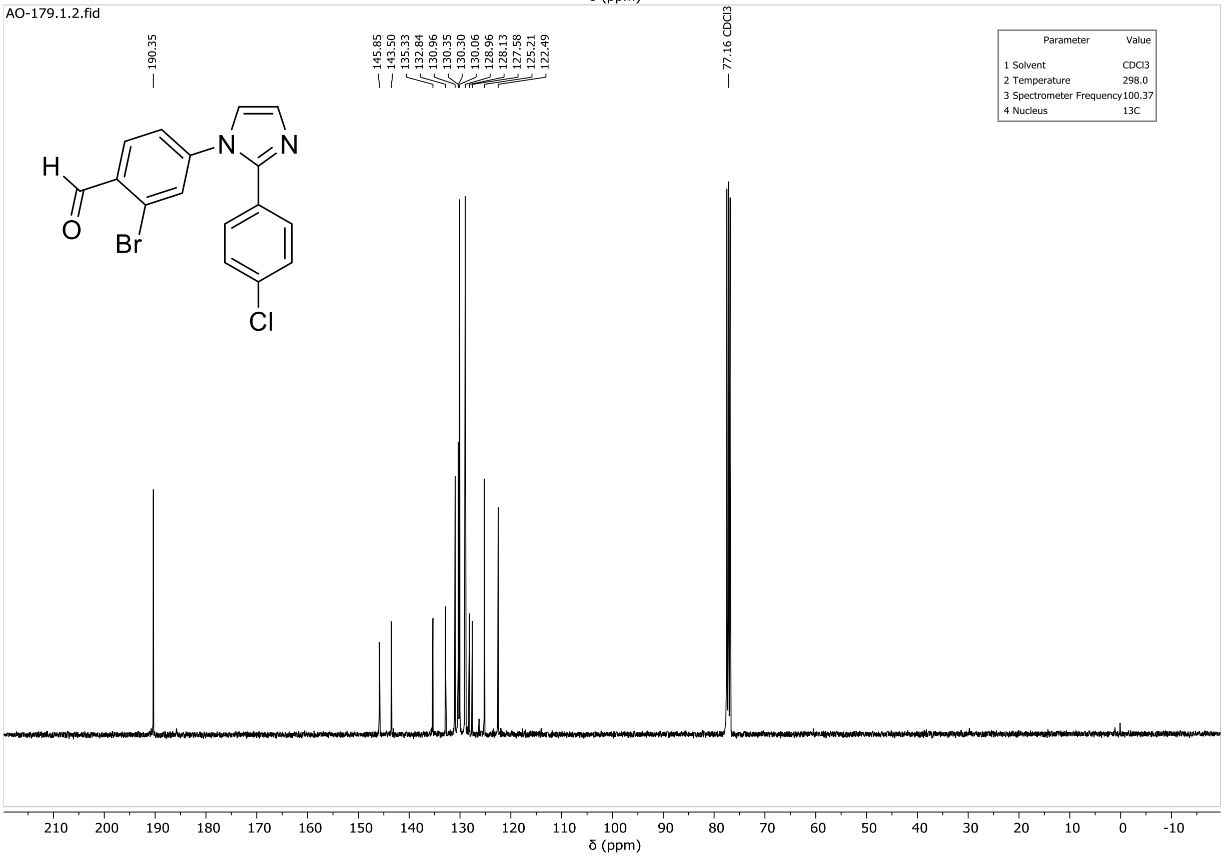

SI Figure S62: ^{1}H- and ^{13}C{^{1}H}-NMR spectra of imidazole **14.**

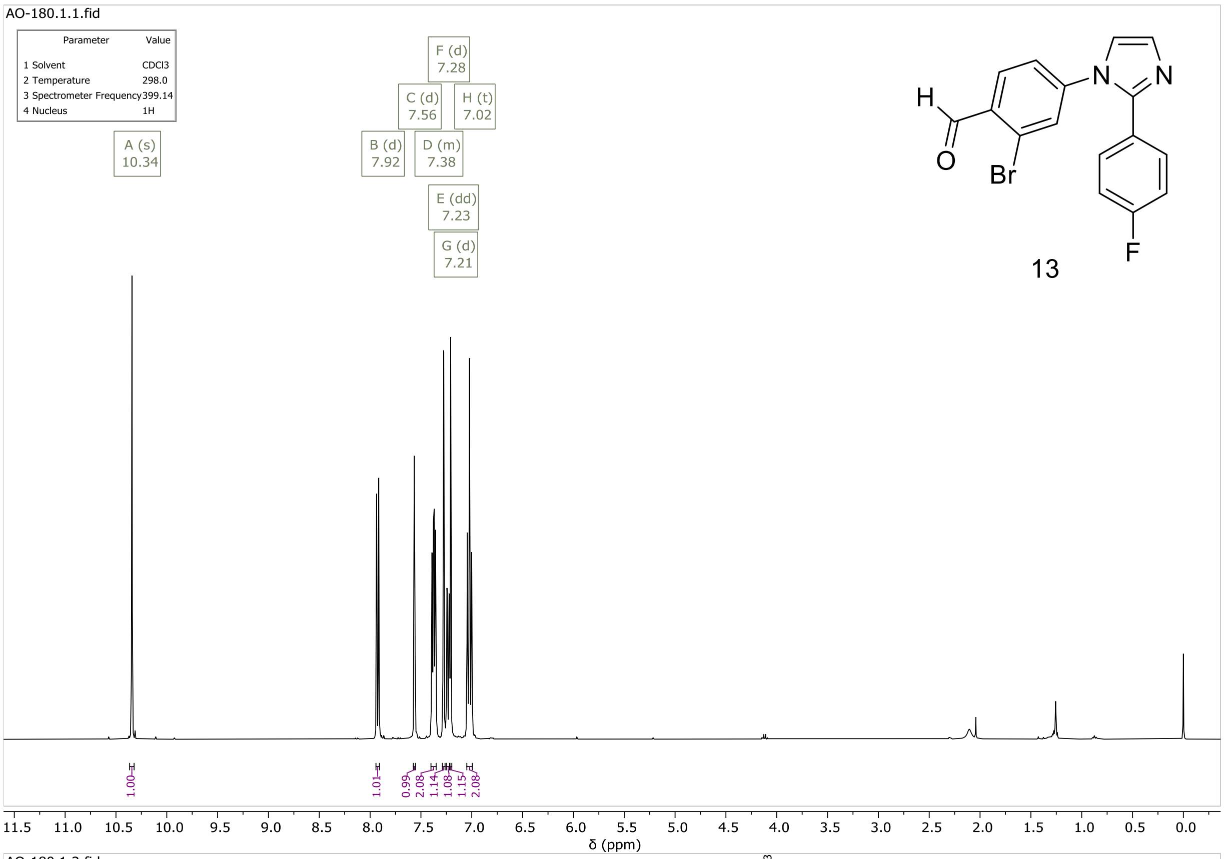

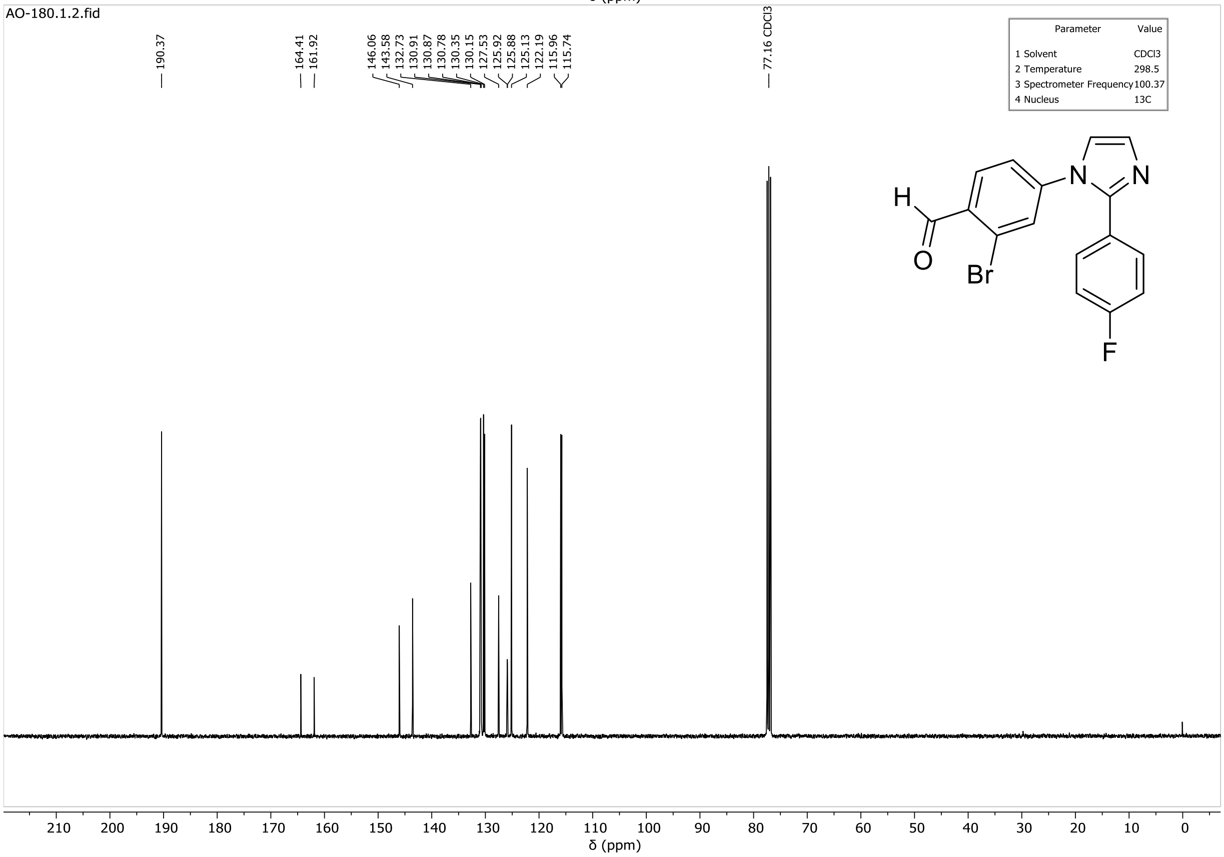

SI Figure S63: ^{1}H- and $^{13}C\{^{1}H\}$-NMR spectra of imidazole **13.**

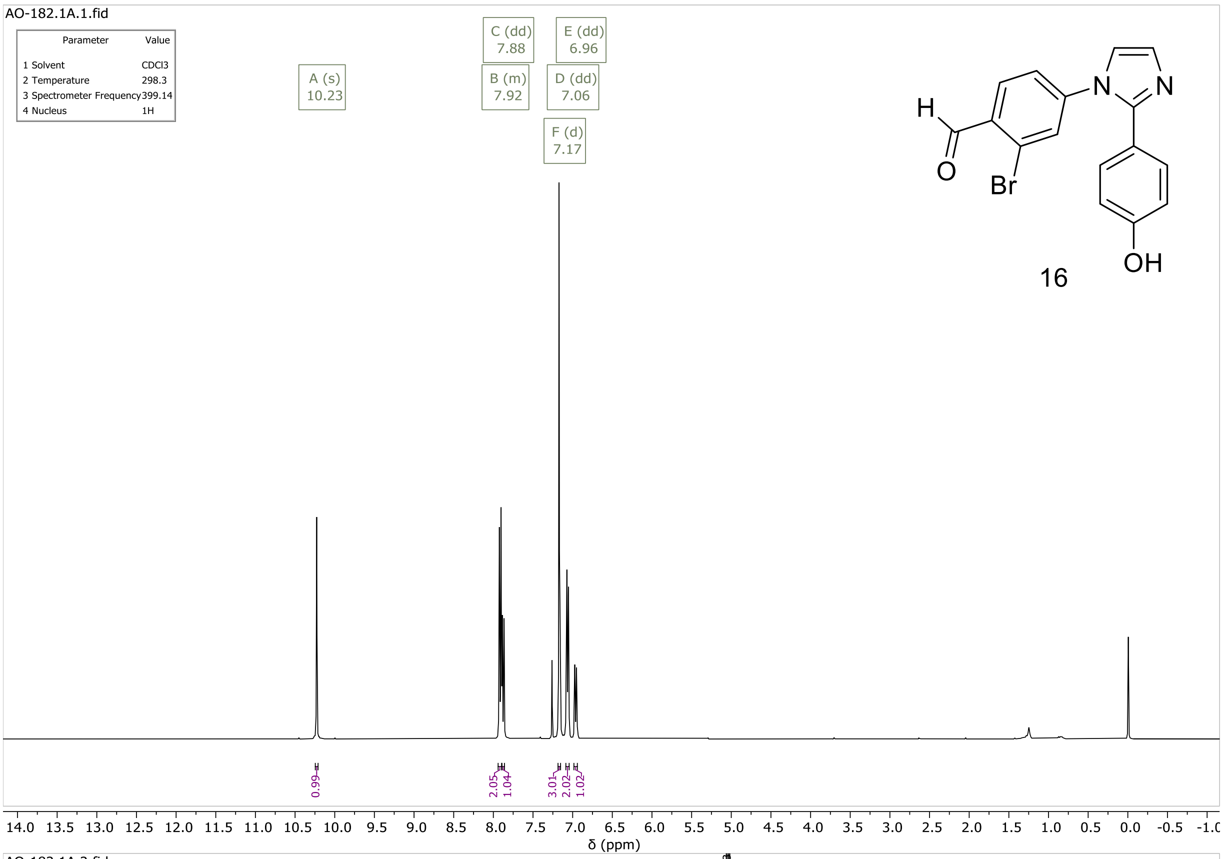

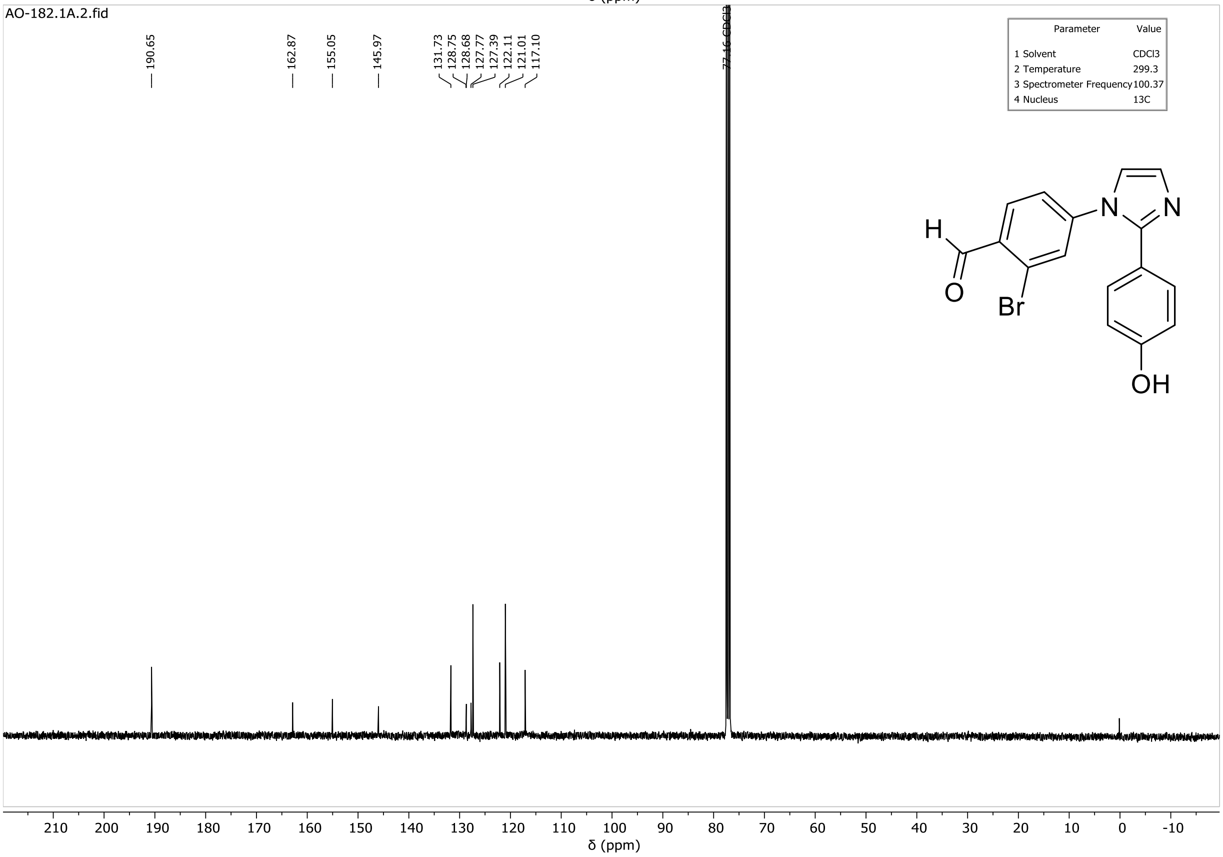

SI Figure S64: ^{1}H- and $^{13}C\{^{1}H\}$-NMR spectra of imidazole **16.**

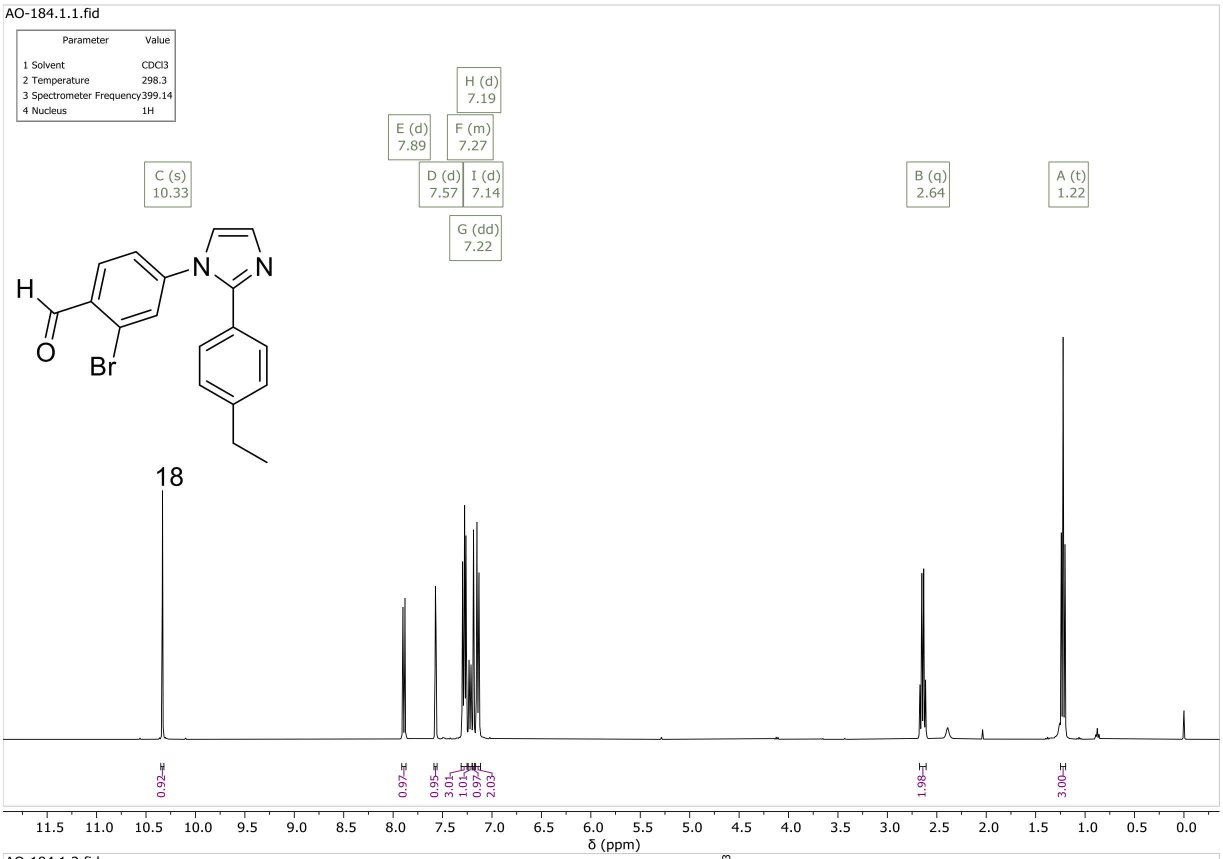

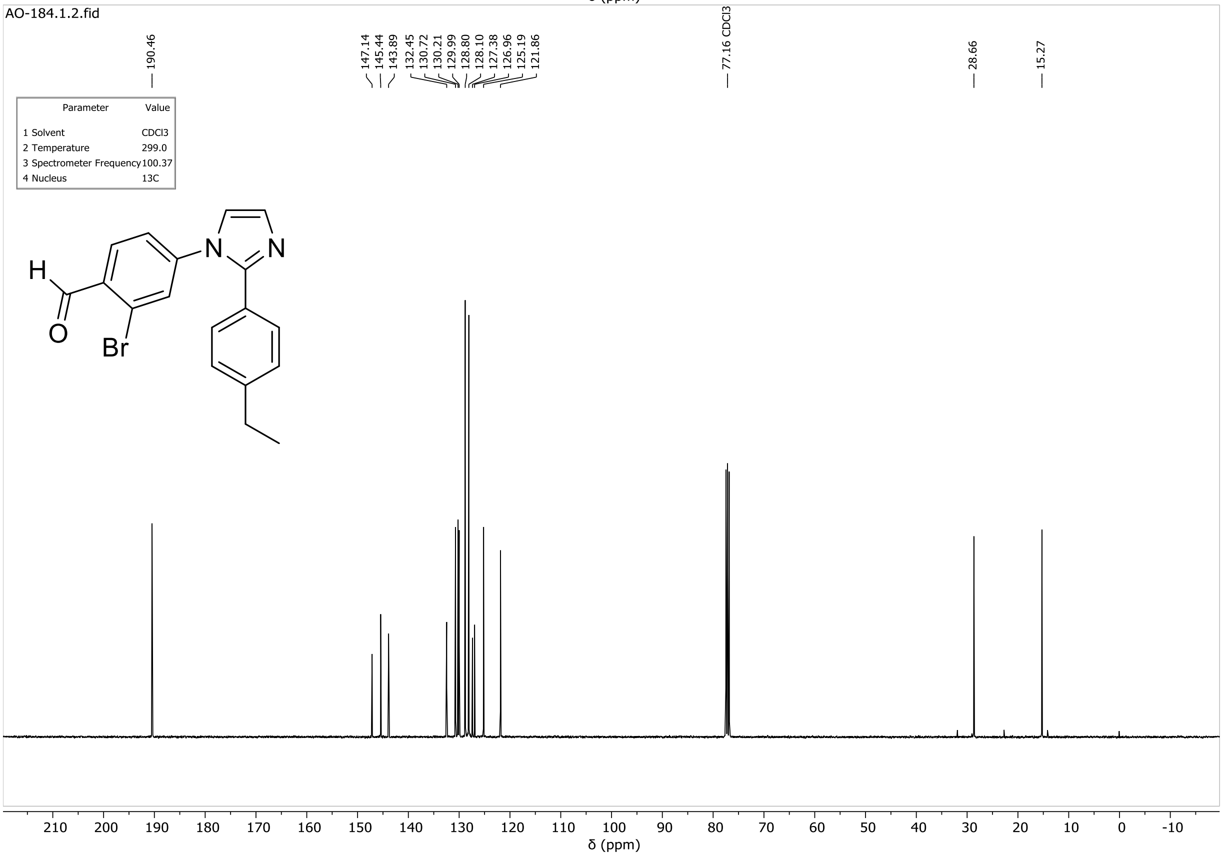

SI Figure S65: ^{1}H- and ^{13}C{^{1}H}-NMR spectra of imidazole **18.**

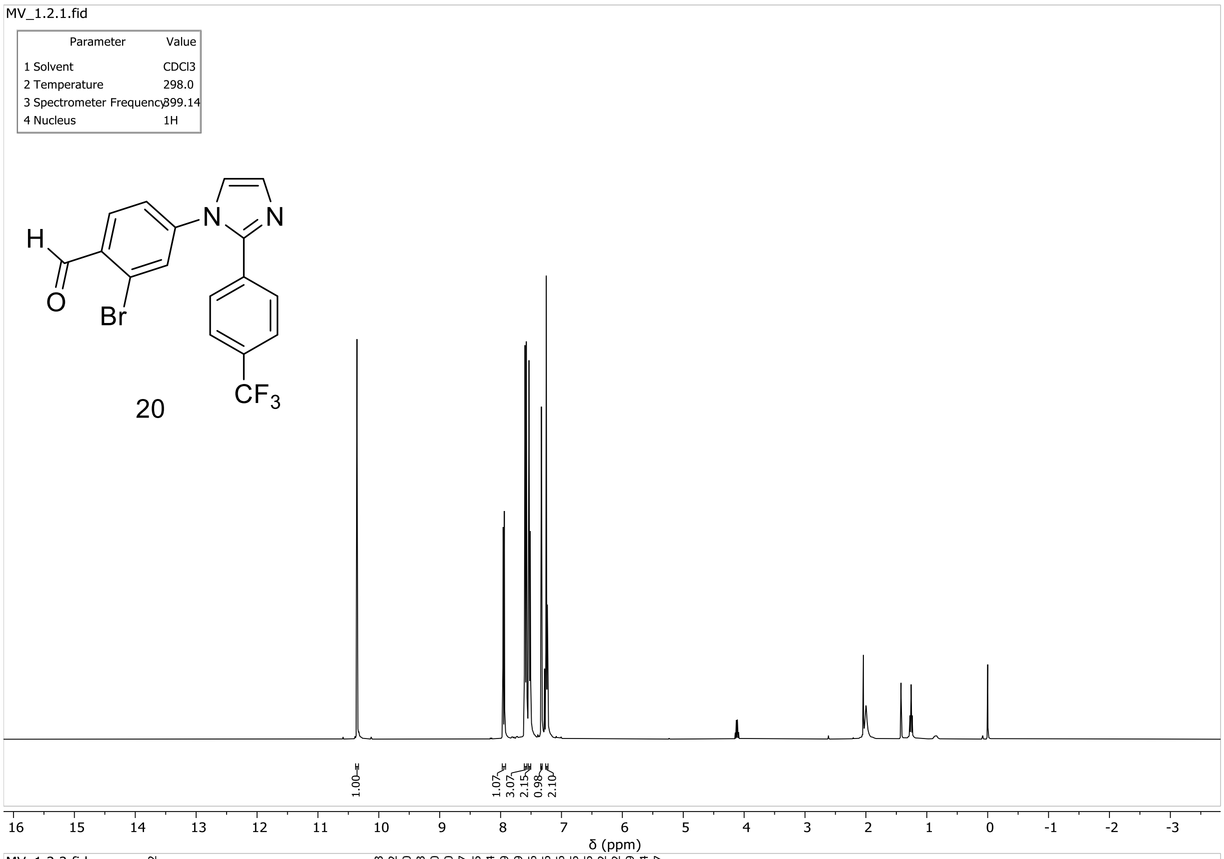

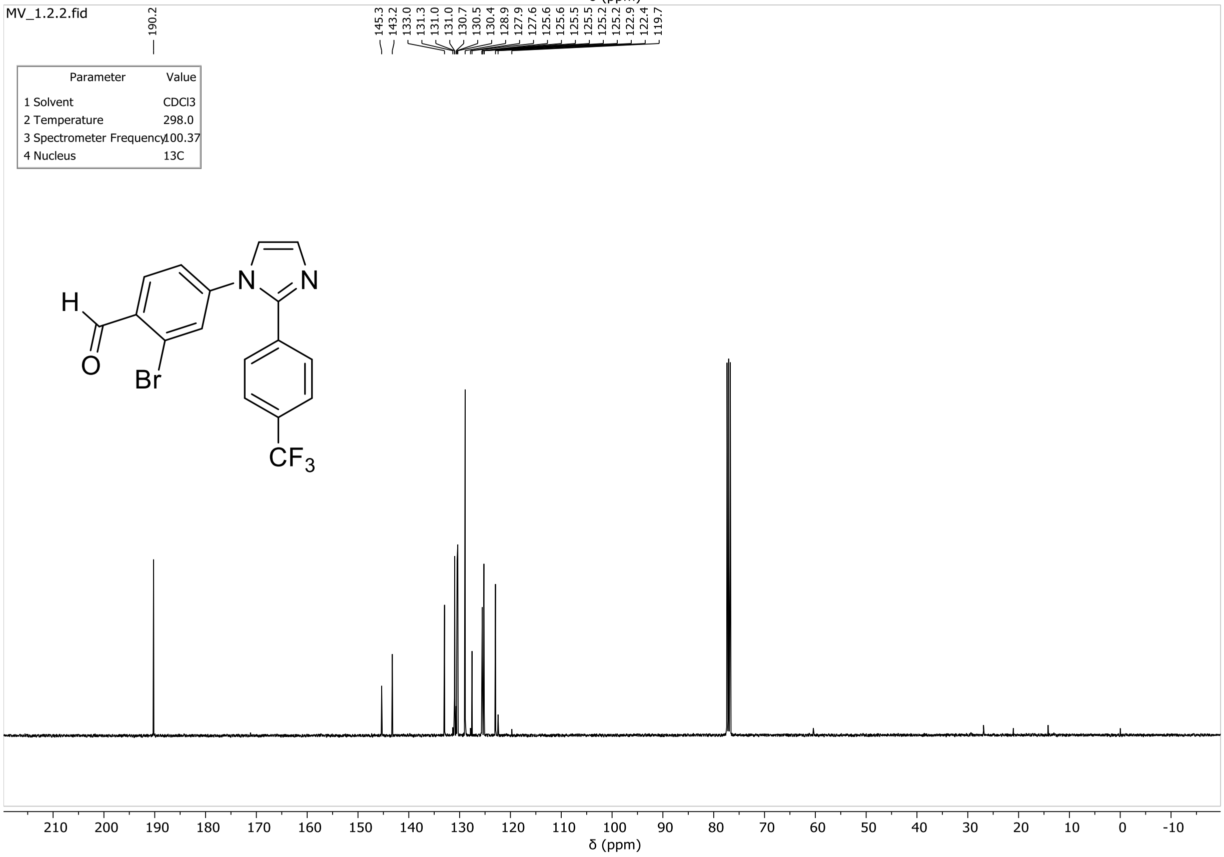

SI Figure S66: ^{1}H- and ^{13}C{^{1}H}-NMR spectra of imidazole **20.**

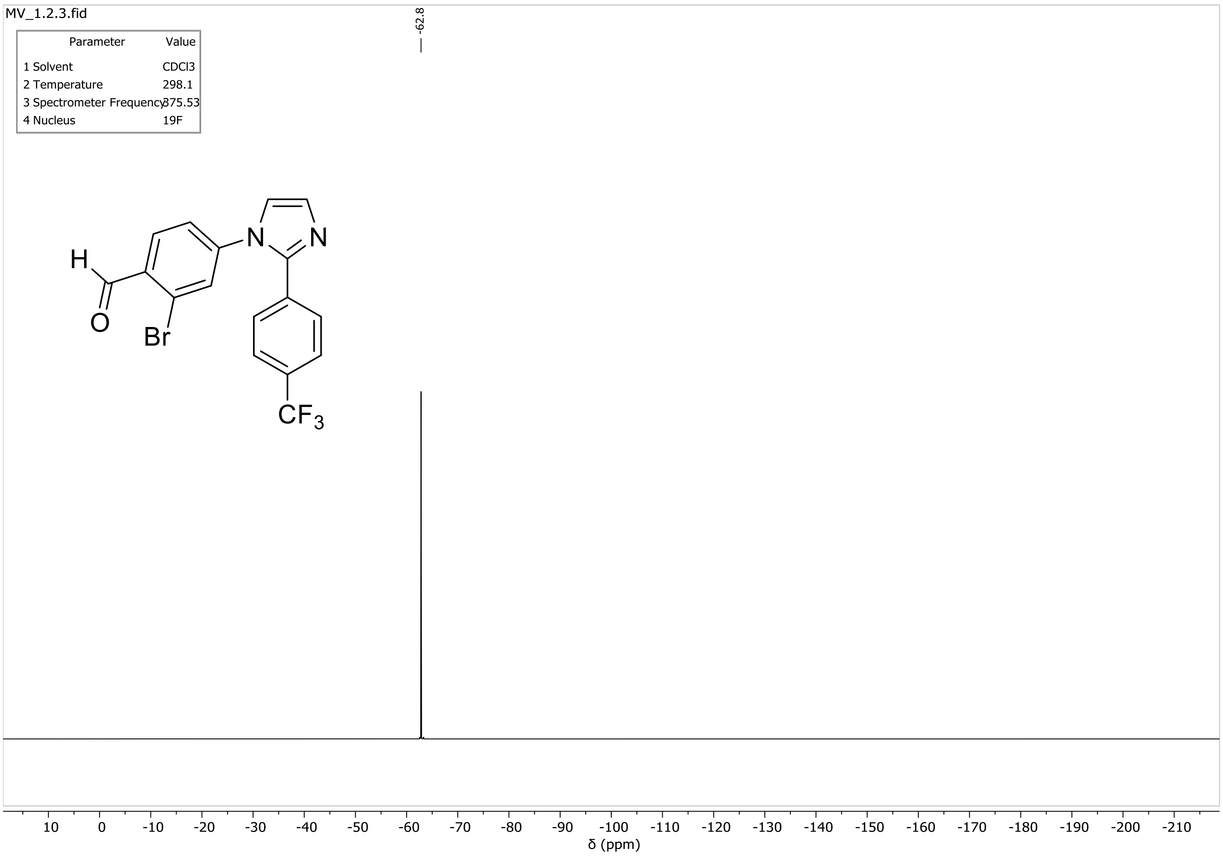

SI Figure S67: ^{19}F-NMR spectrum of imidazole **20**.

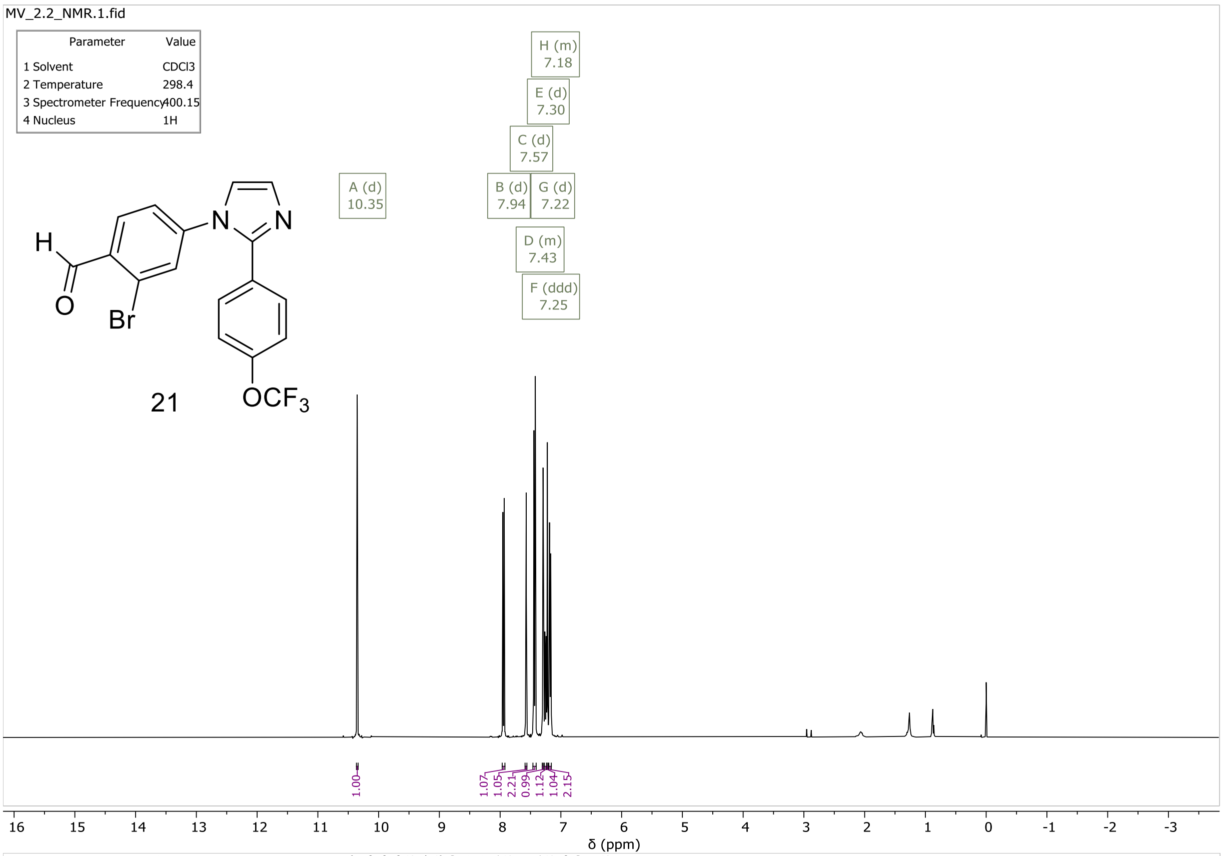

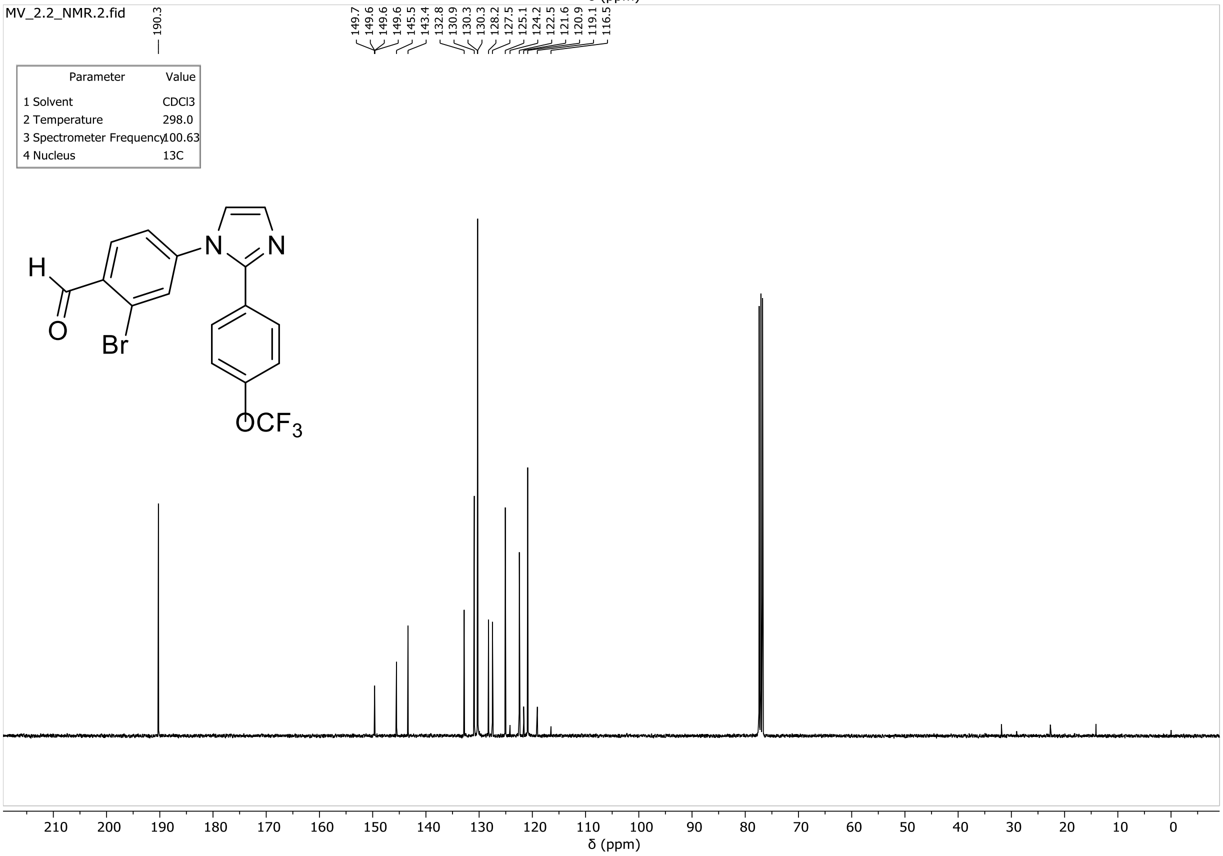

SI Figure S68: ^{1}H- and $^{13}C\{^{1}H\}$-NMR spectra of imidazole **21**.

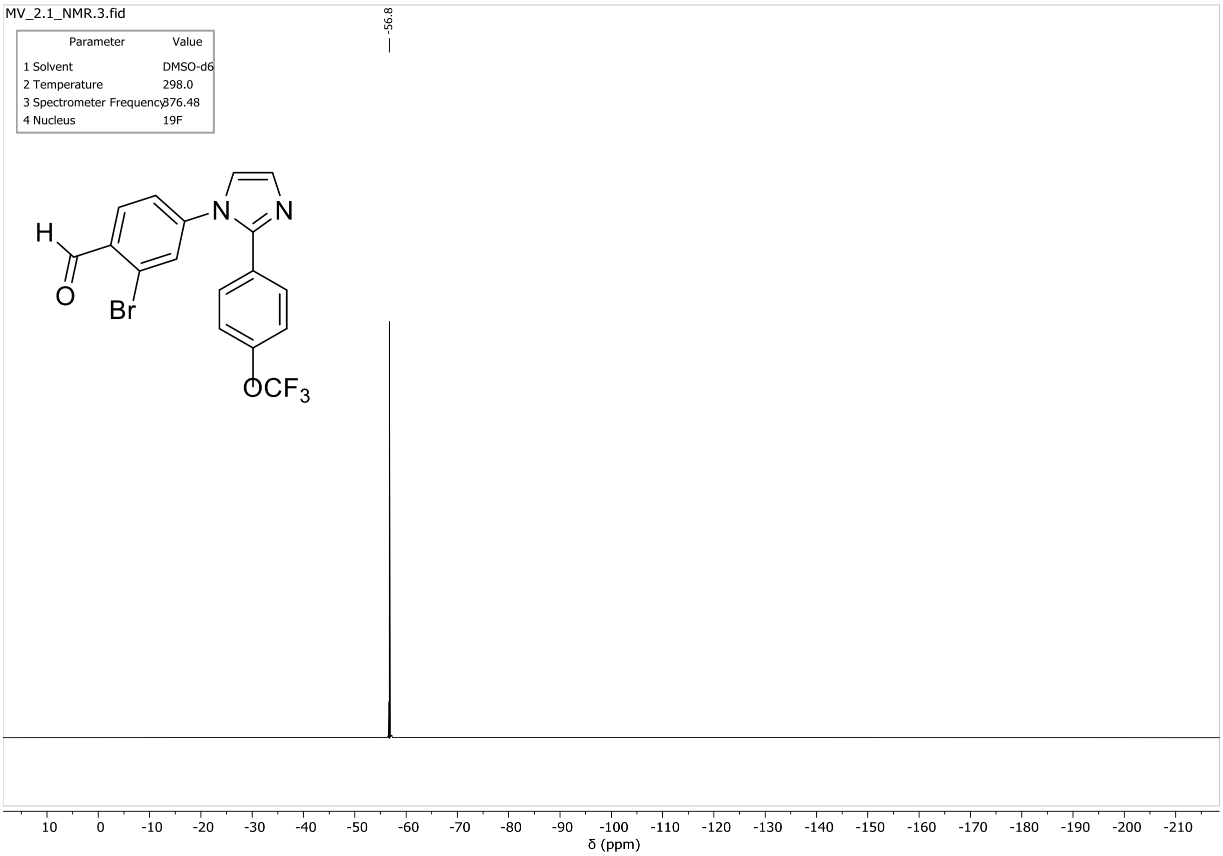

SI Figure S69: ^{19}F-NMR spectrum of imidazole **21**.

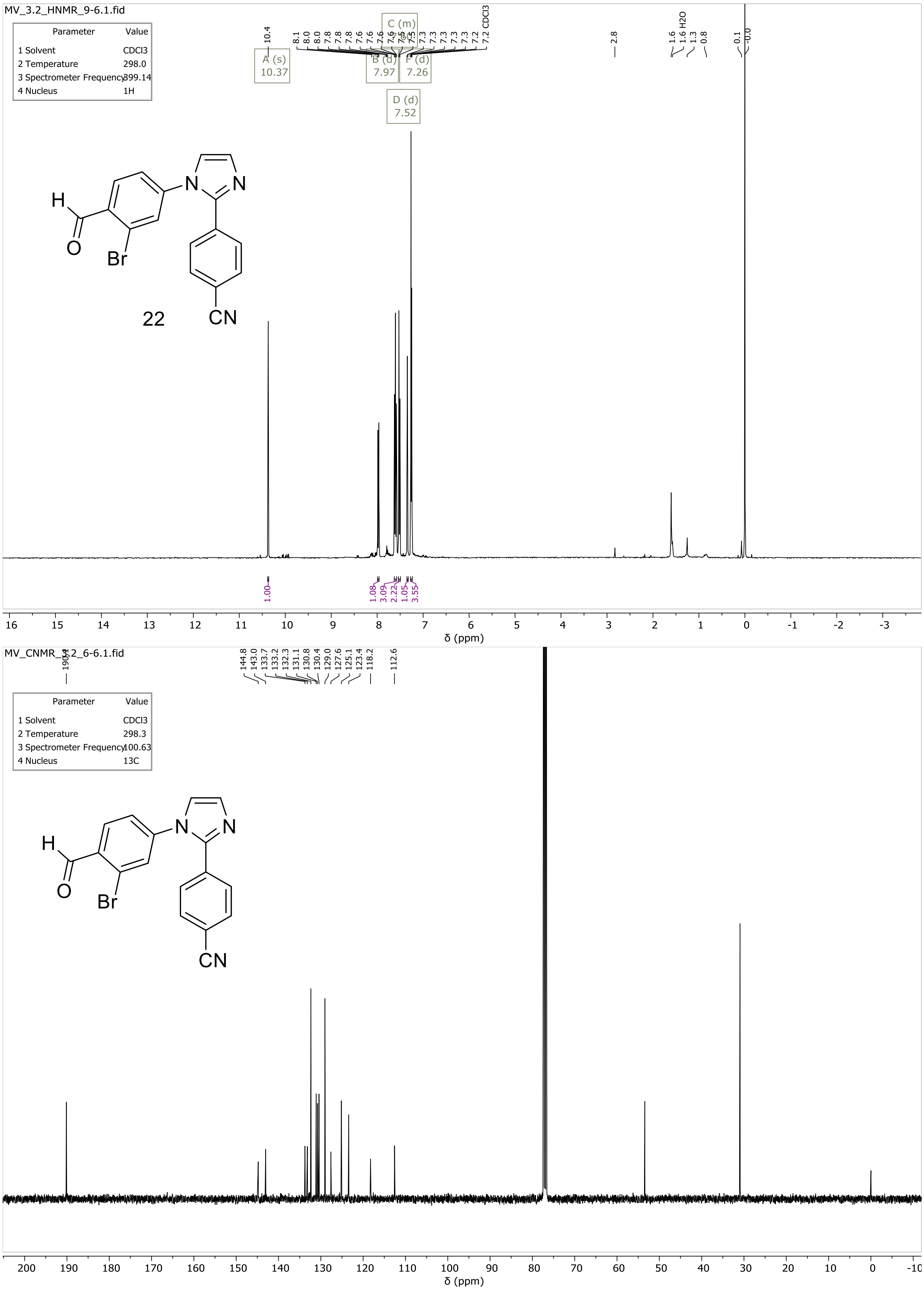

SI Figure S70: ^{1}H- and $^{13}C\{^{1}H\}$-NMR spectra of imidazole **22.**

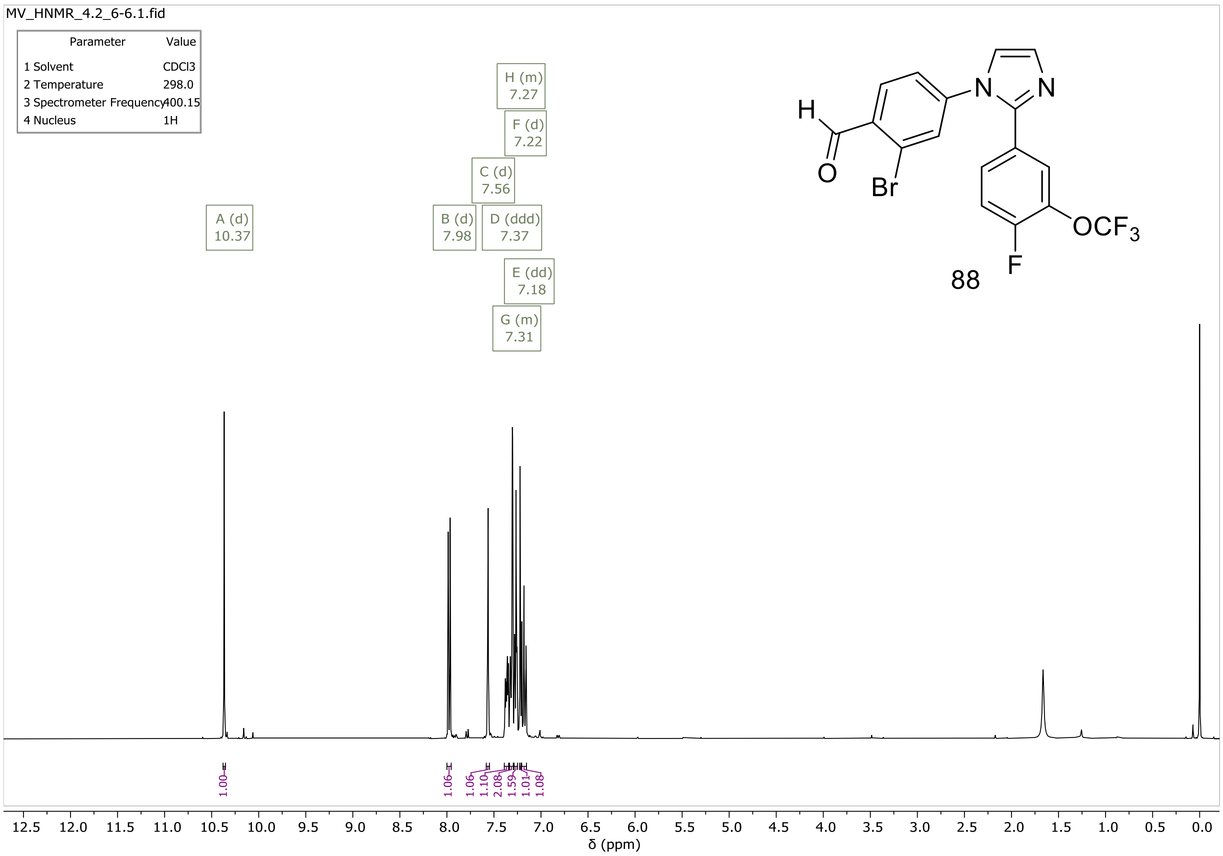

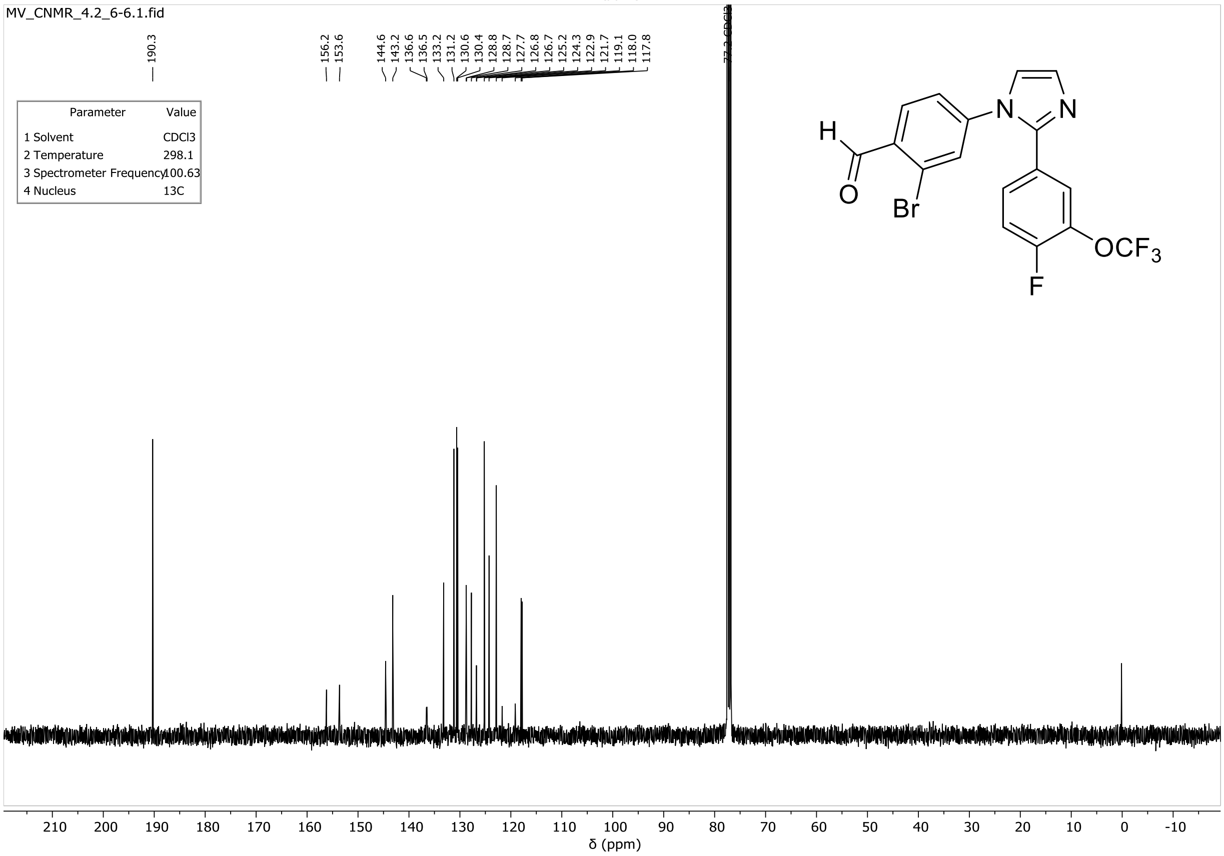

SI Figure S71: ^{1}H- and ^{13}C{^{1}H}-NMR spectra of imidazole **88.**

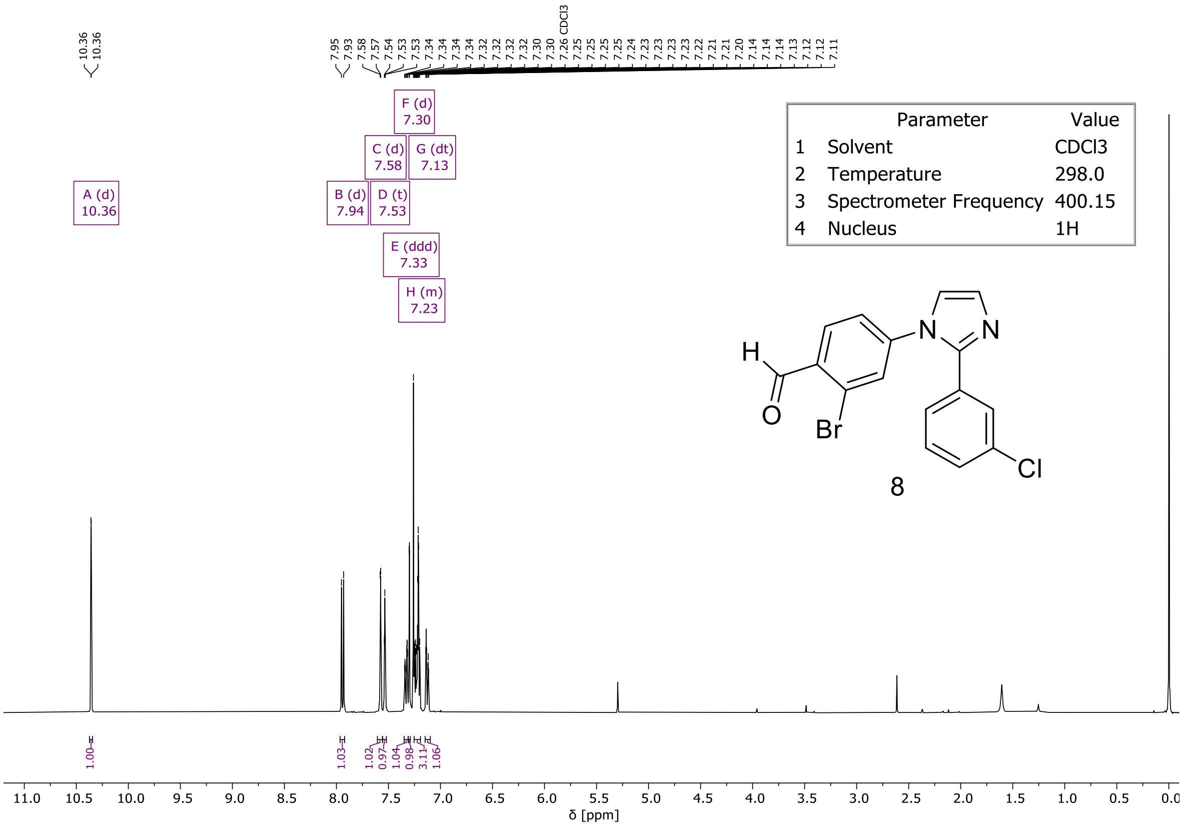

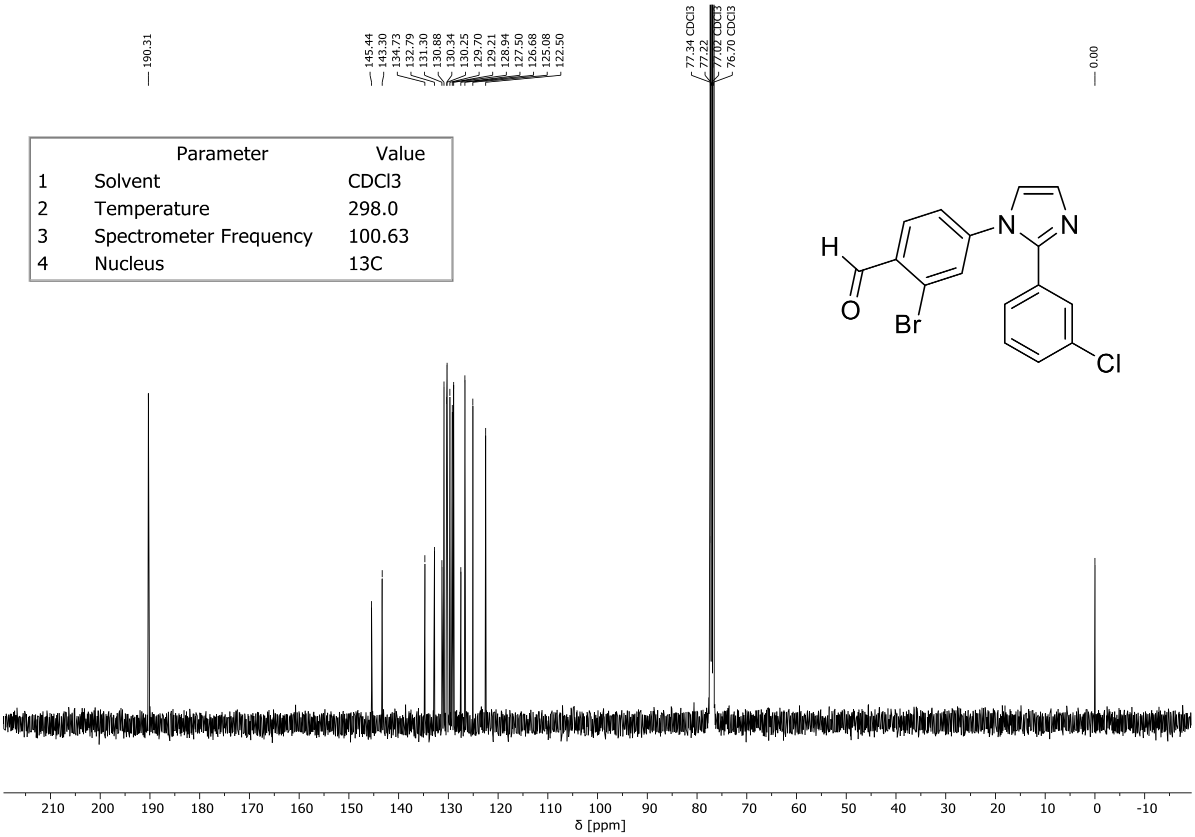

SI Figure S72: ^{1}H- and ^{13}C{^{1}H}-NMR spectra of imidazole **8**.

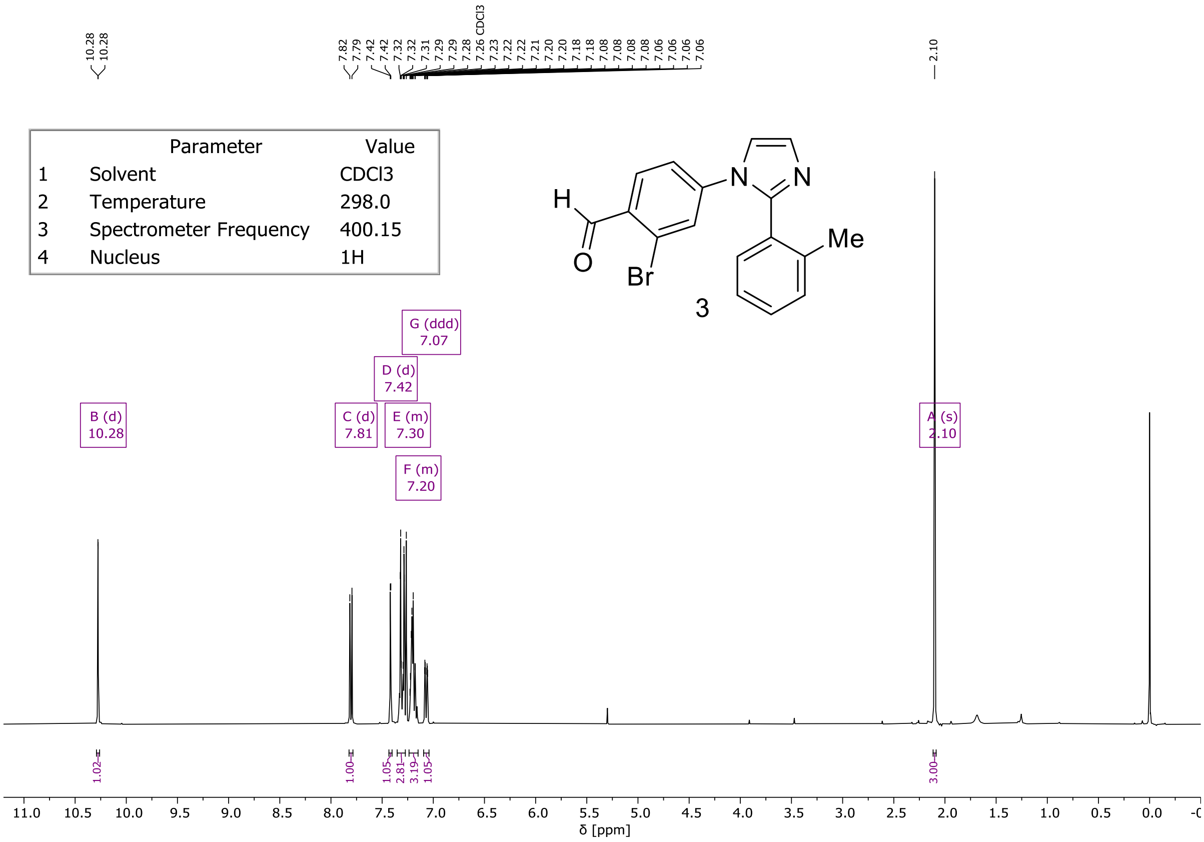

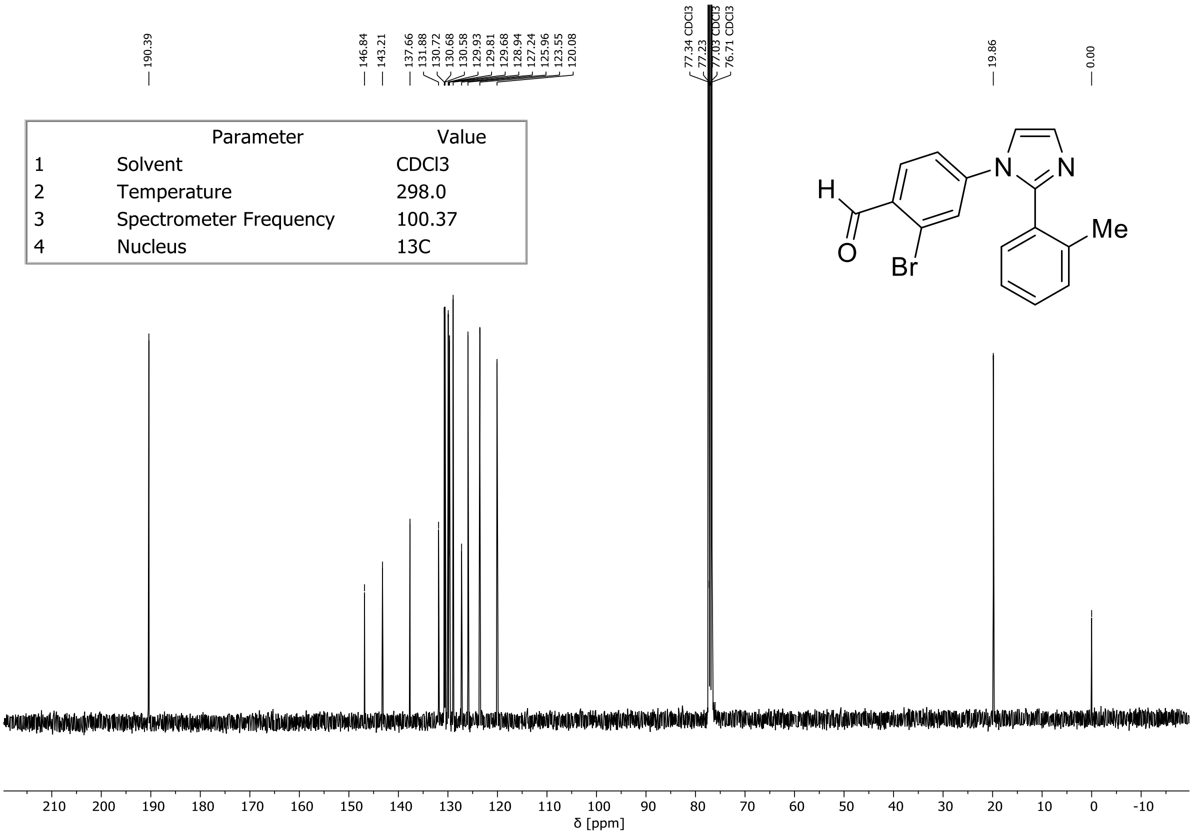

SI Figure S73: ^{1}H- and $^{13}C\{^{1}H\}$-NMR spectra of imidazole **3**.

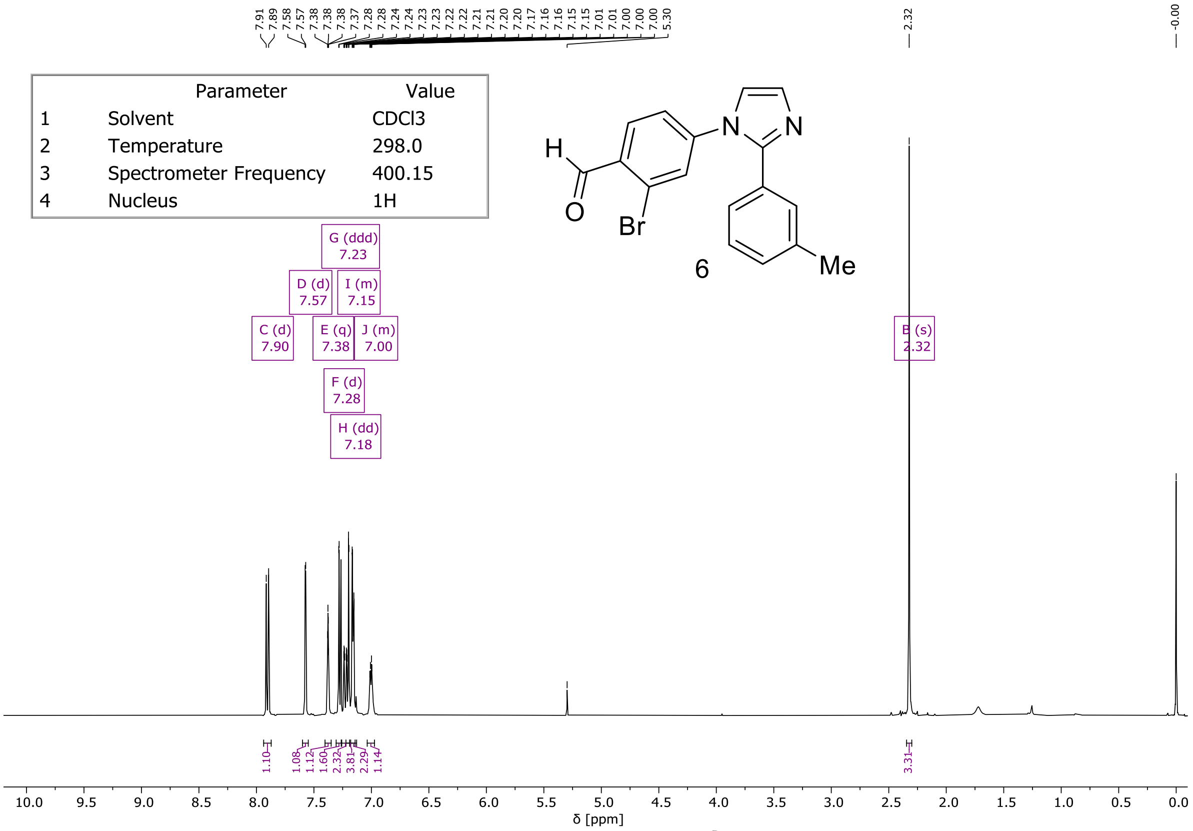

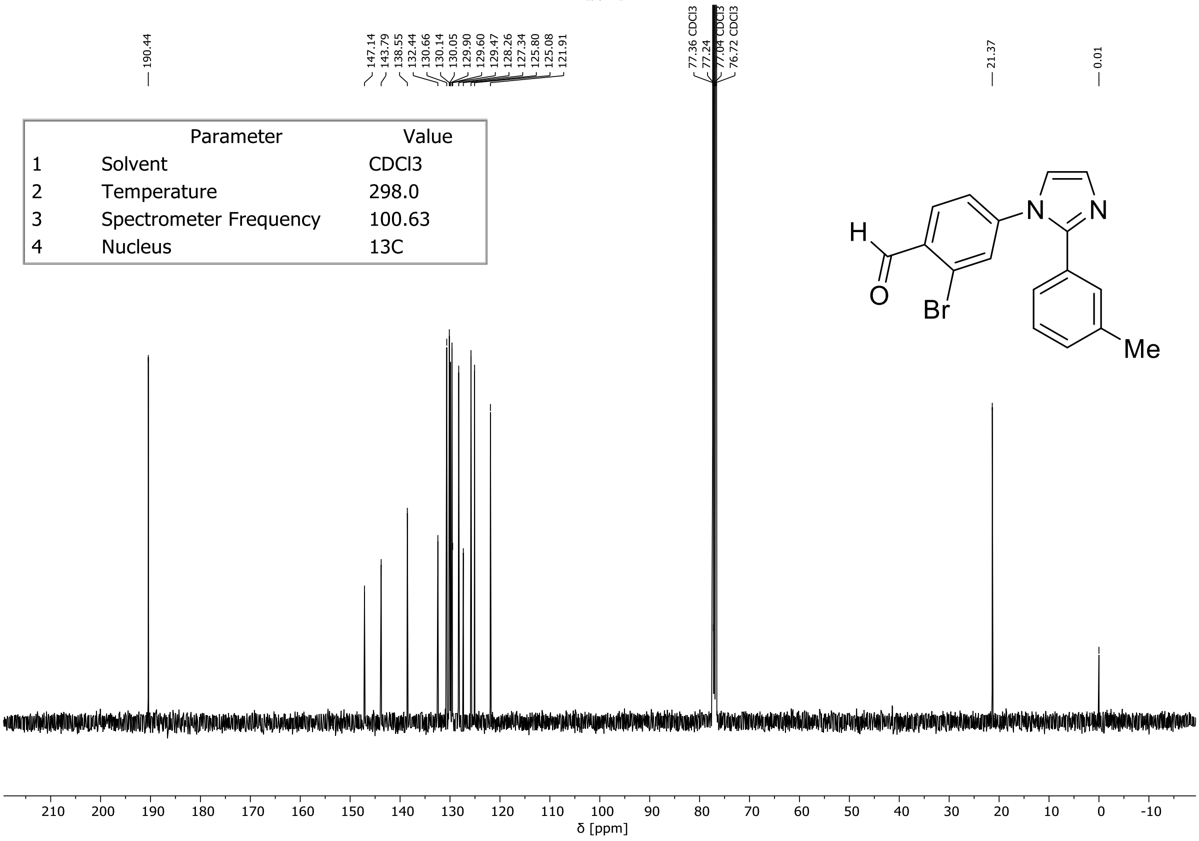

SI Figure S74: ^{1}H- and ^{13}C{^{1}H}-NMR spectra of imidazole **6**.

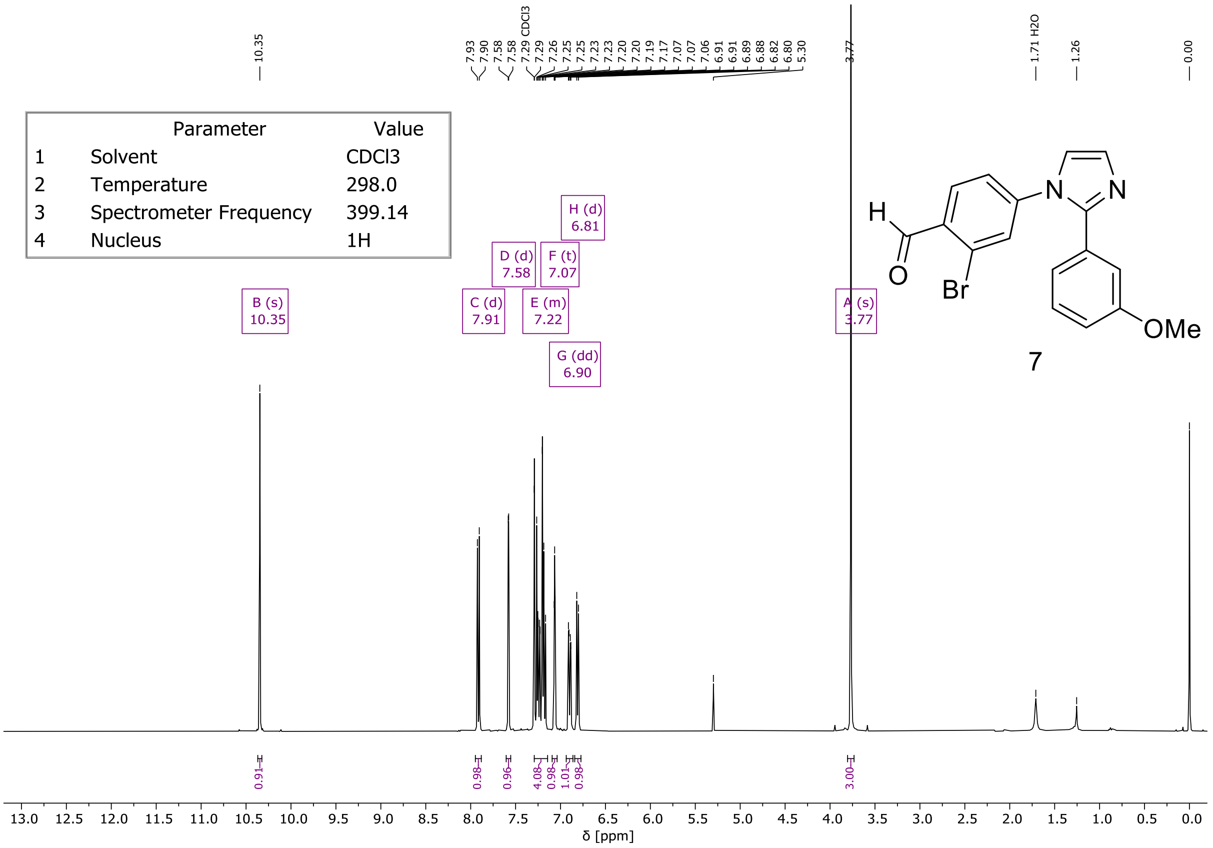

	Parameter	Value
1	Solvent	CDCl3
2	Temperature	298.0
3	Spectrometer Frequency	399.14
4	Nucleus	1H

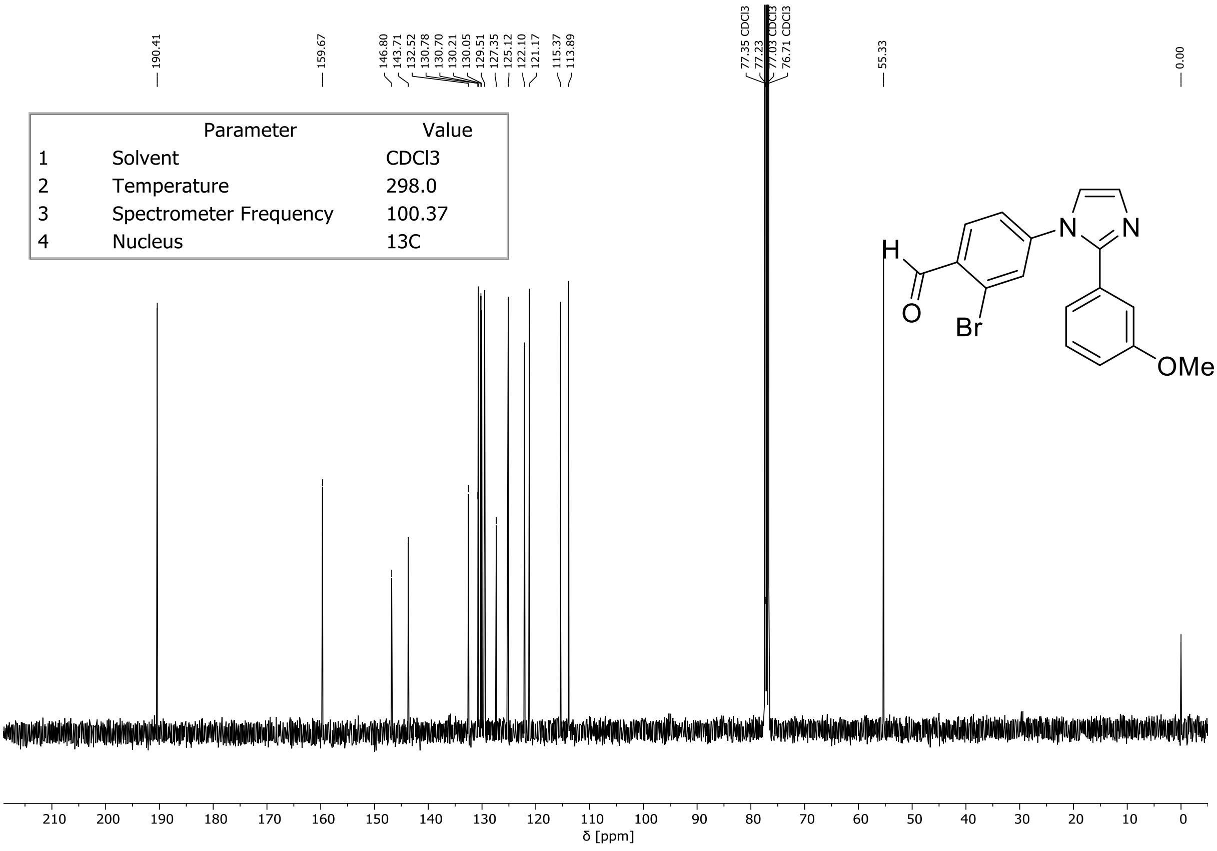

	Parameter	Value
1	Solvent	CDCl3
2	Temperature	298.0
3	Spectrometer Frequency	100.37
4	Nucleus	13C

SI Figure S75: ^{1}H- and ^{13}C{^{1}H}-NMR spectra of imidazole **7**.

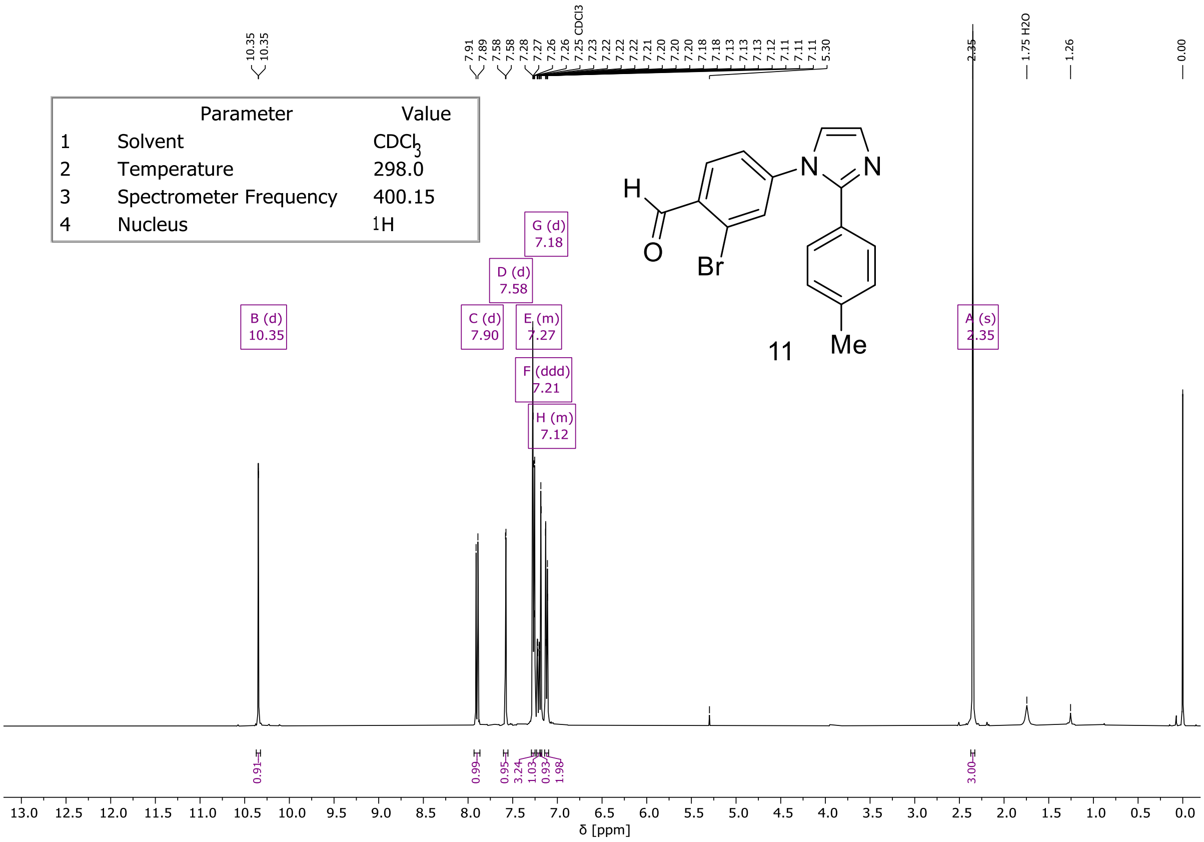

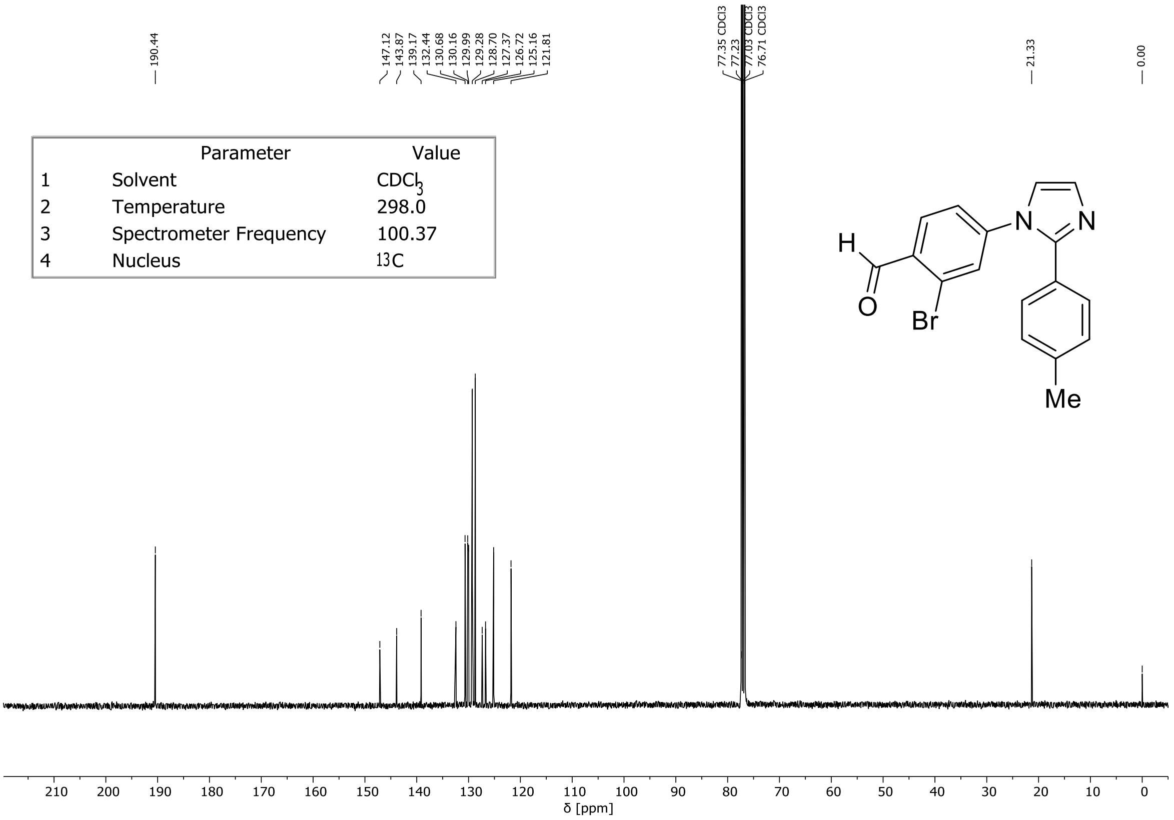

SI Figure S76: ^{1}H- and ^{13}C{^{1}H}-NMR spectra of imidazole **11**.

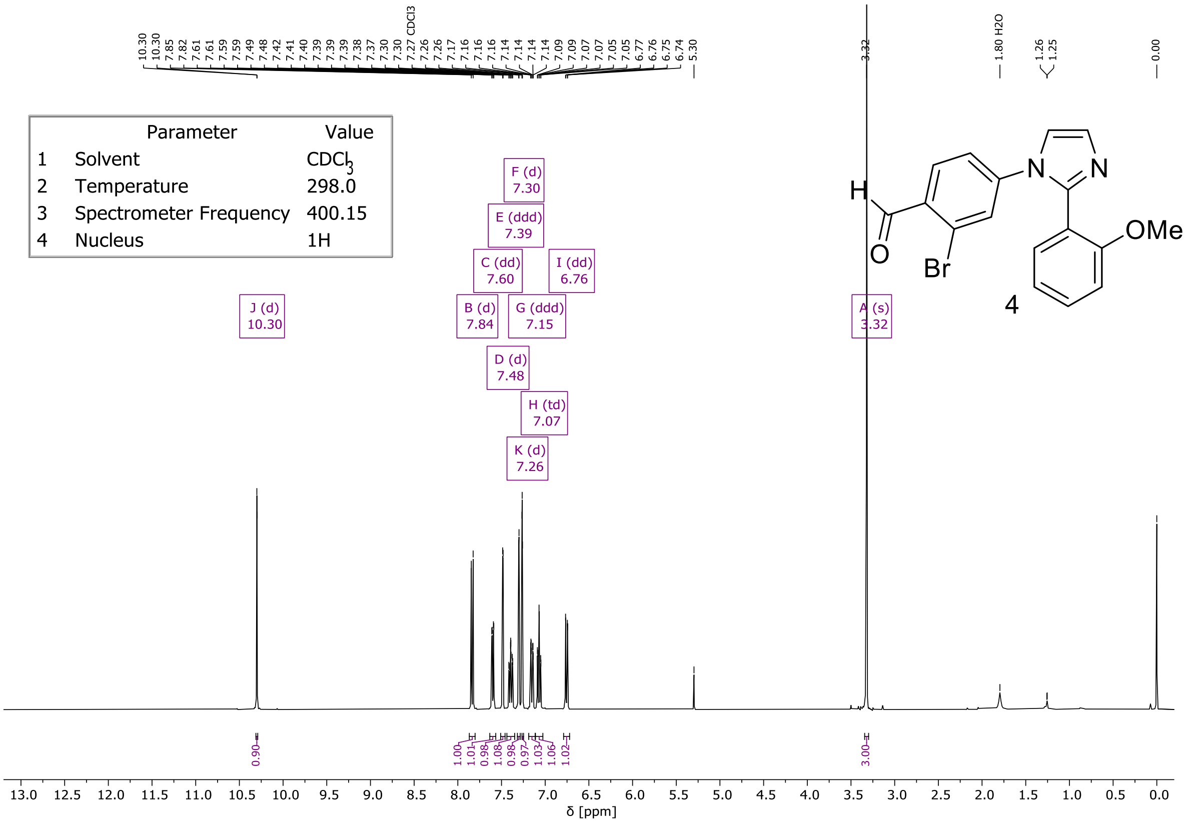

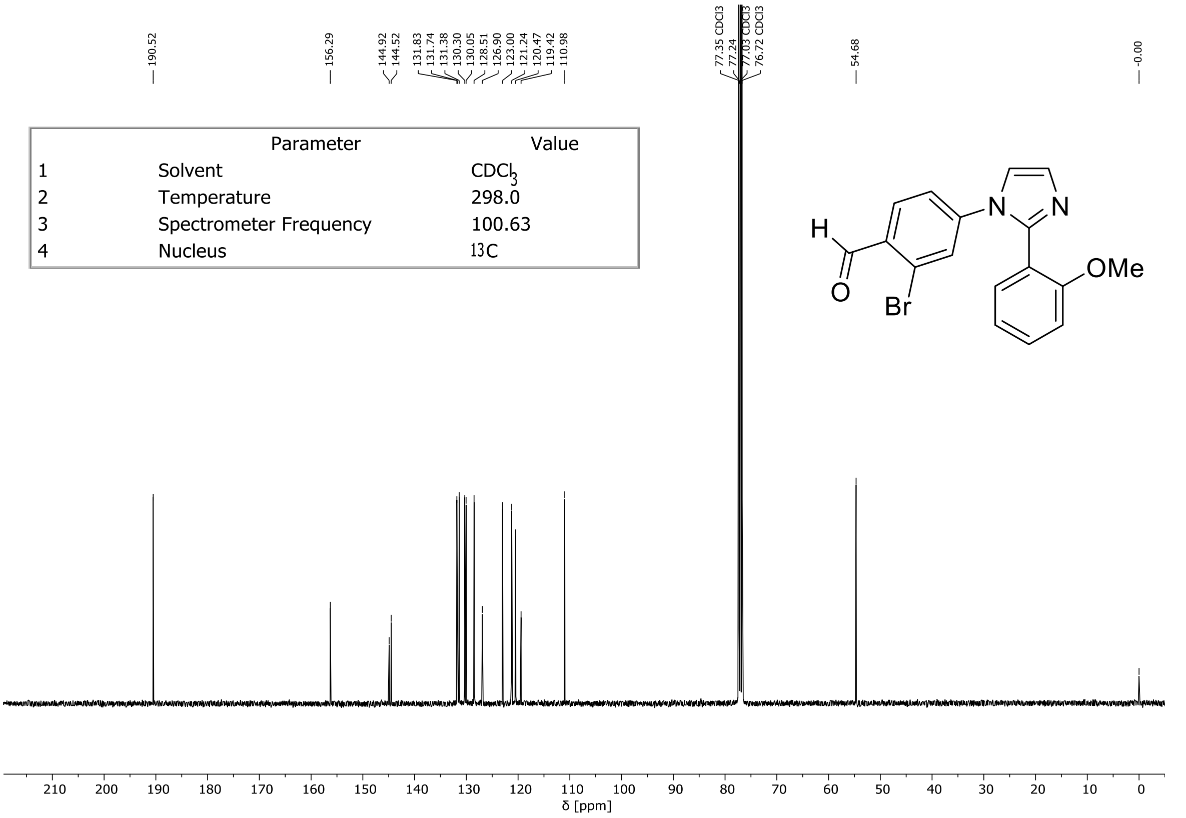

SI Figure S77: ^{1}H- and ^{13}C{^{1}H}-NMR spectra of imidazole **4**.

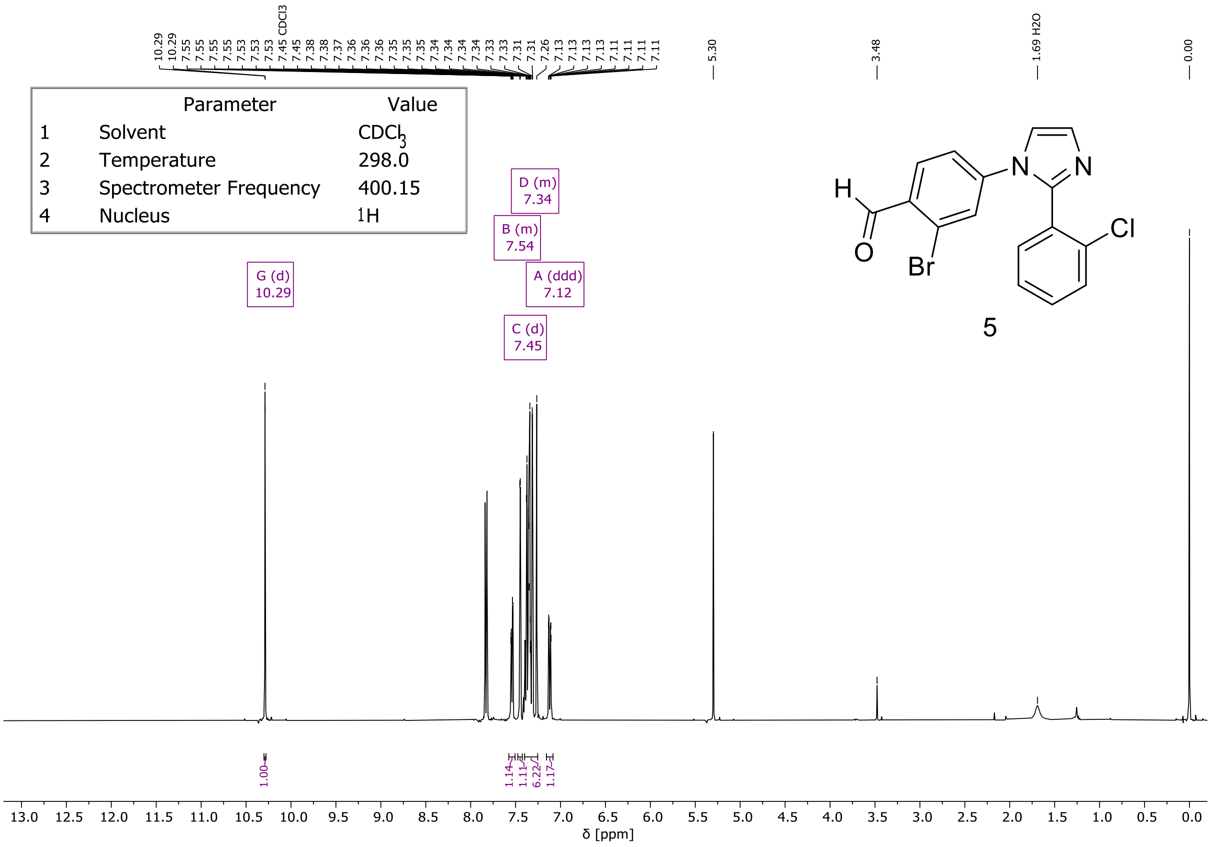

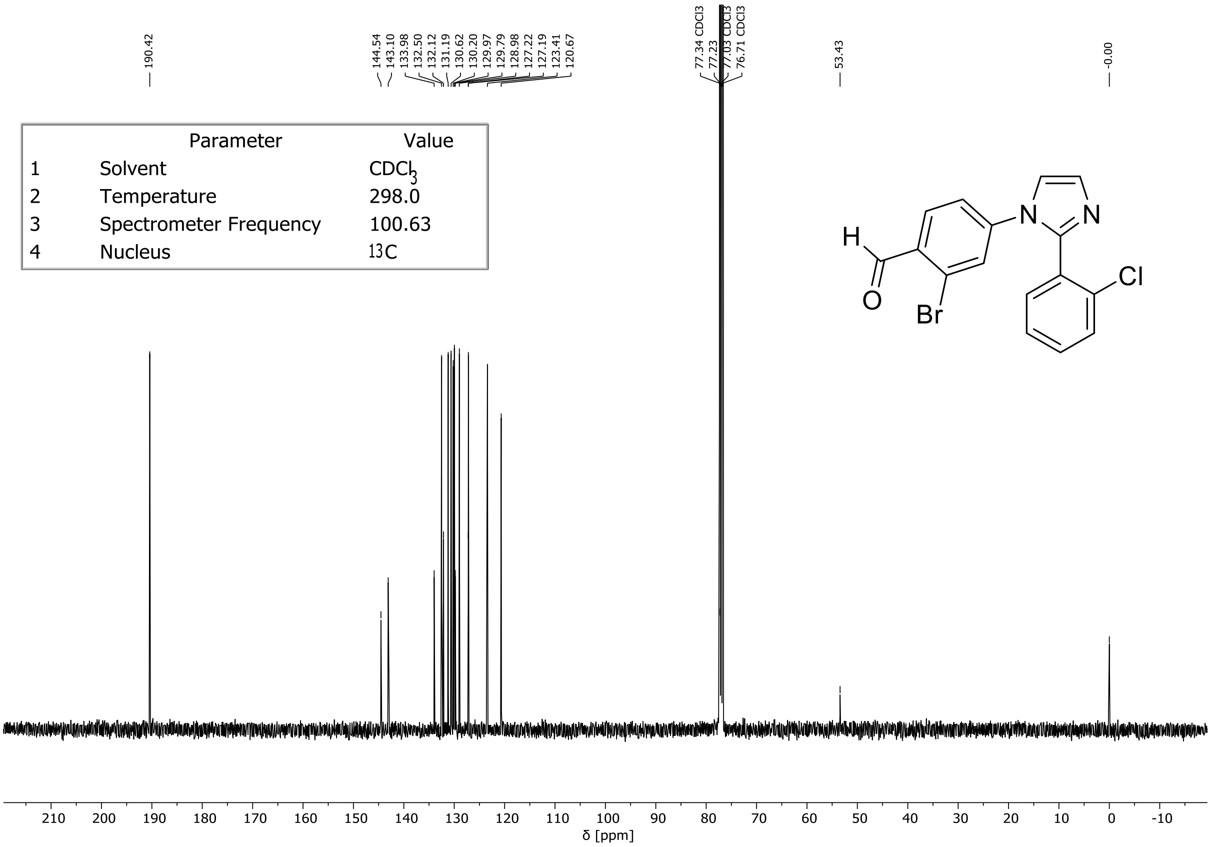

SI Figure S78: ^{1}H- and ^{13}C{^{1}H}-NMR spectra of imidazole **5**.

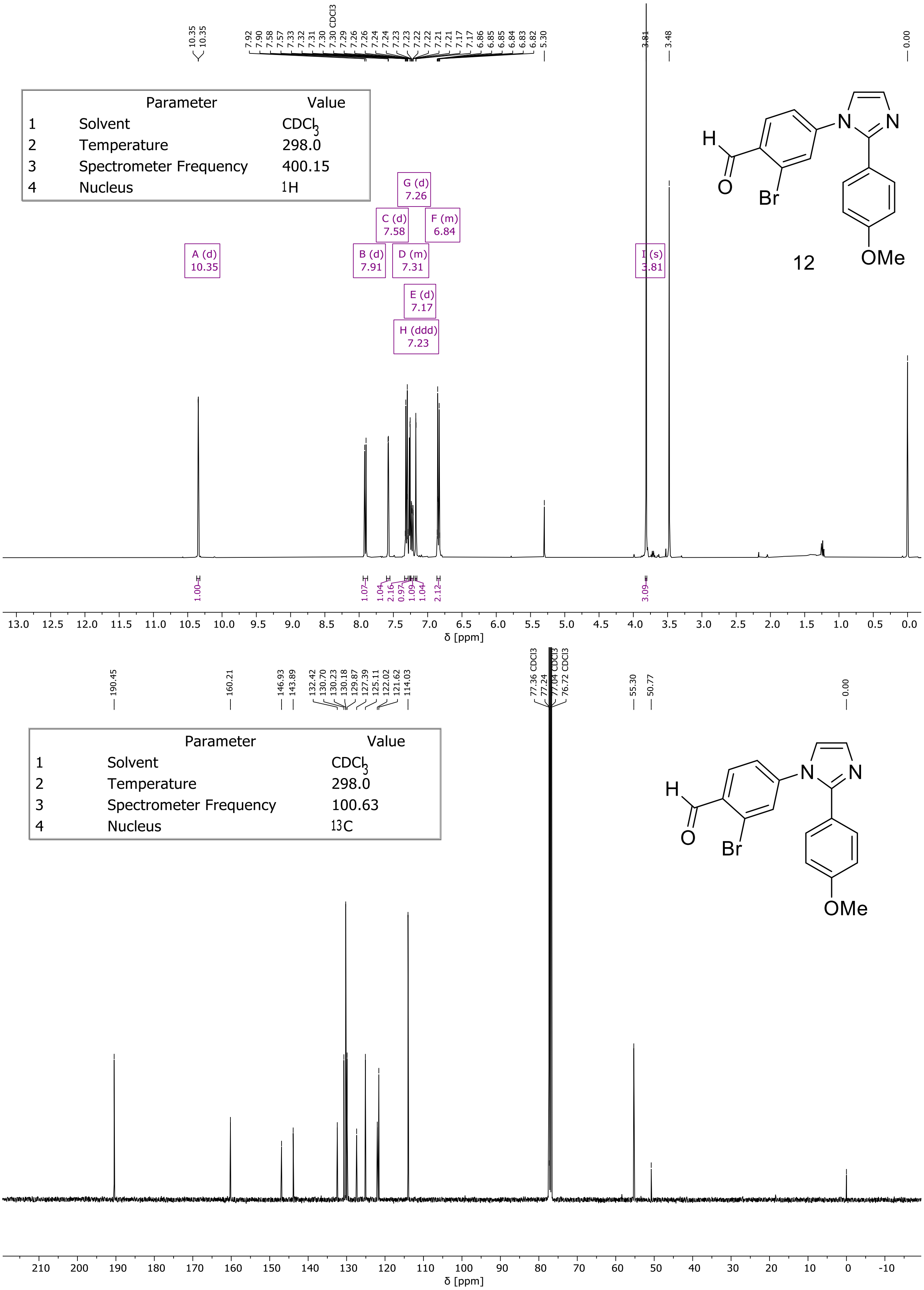

SI Figure S79: ^{1}H- and ^{13}C{^{1}H}-NMR spectra of imidazole **12**.

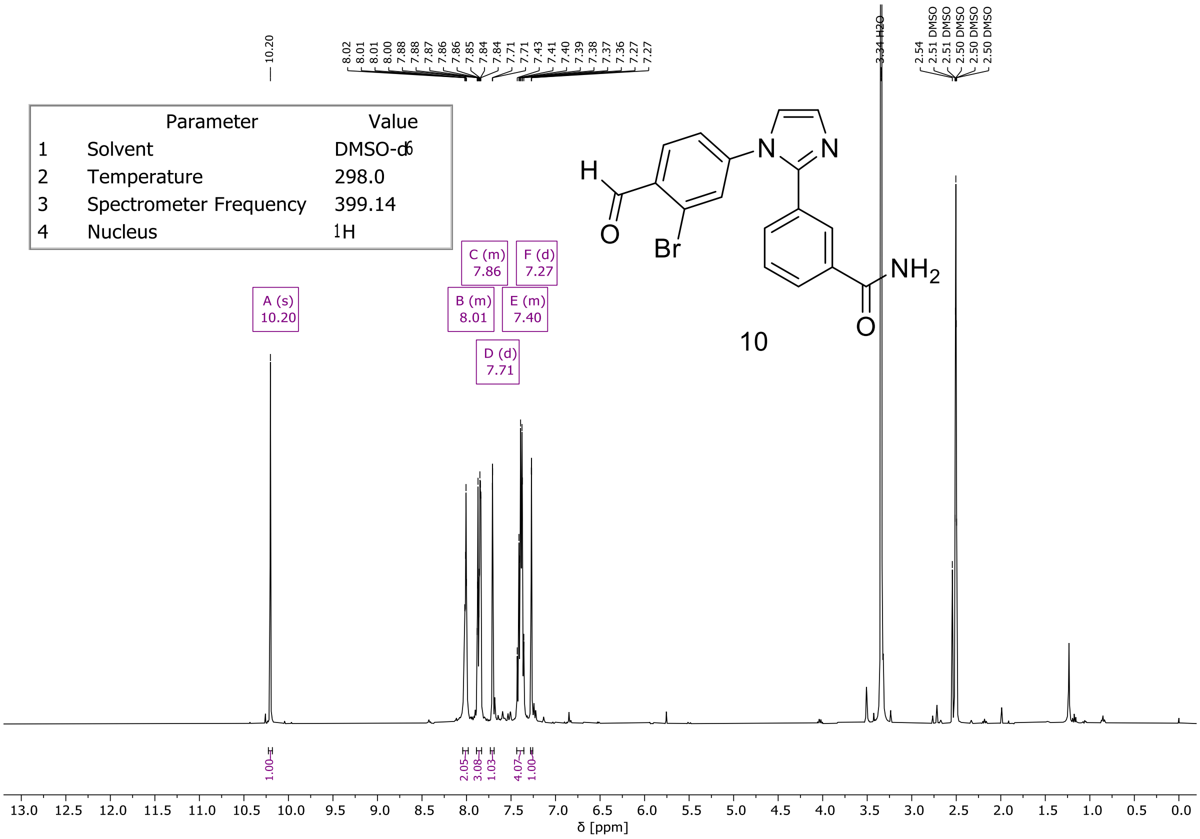

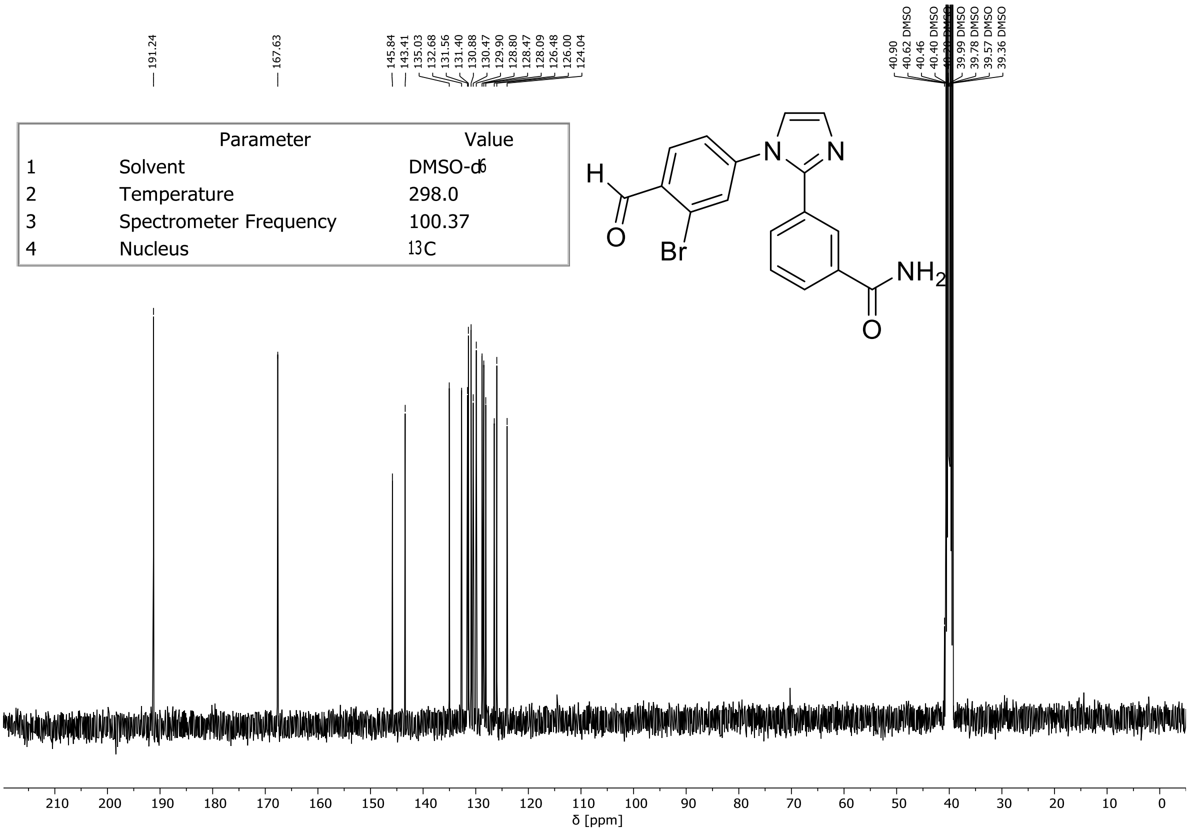

SI Figure S80: ^{1}H- and $^{13}C\{^{1}H\}$-NMR spectra of imidazole **10**.

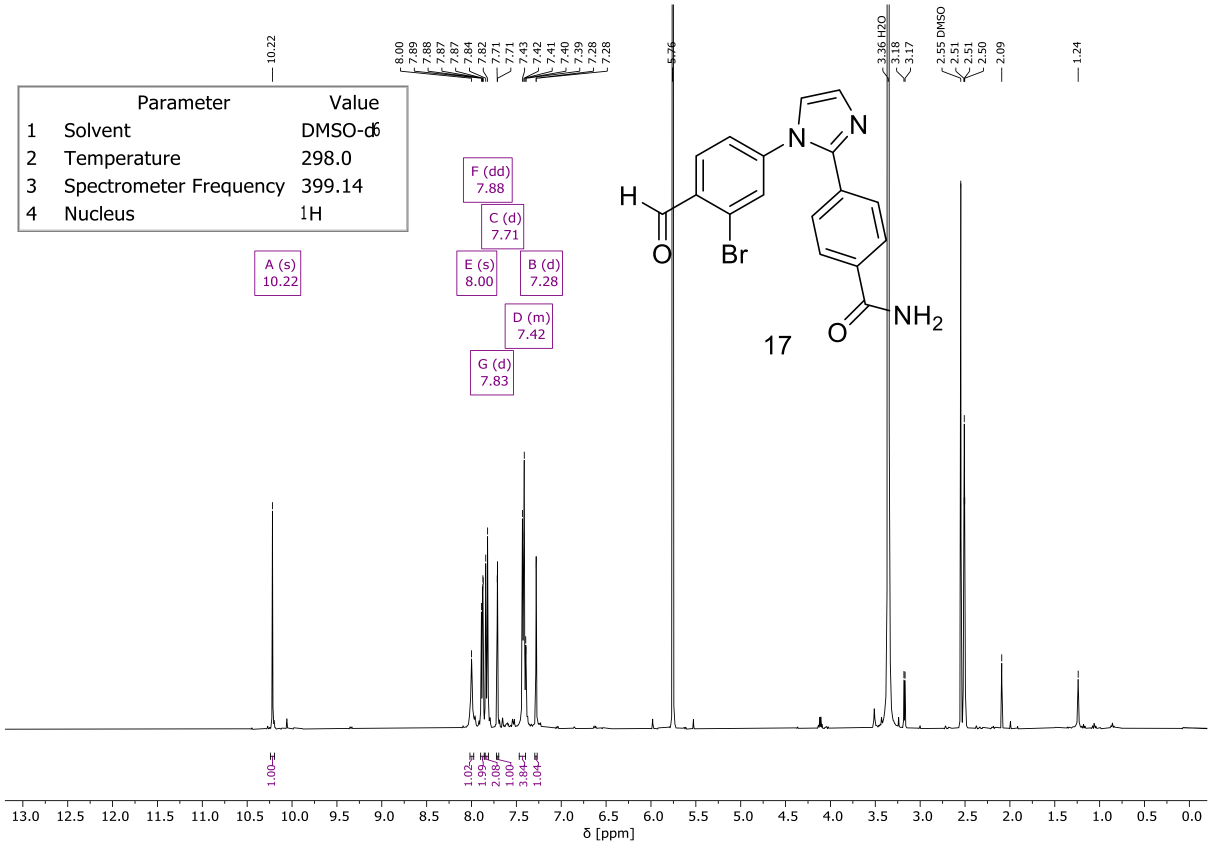

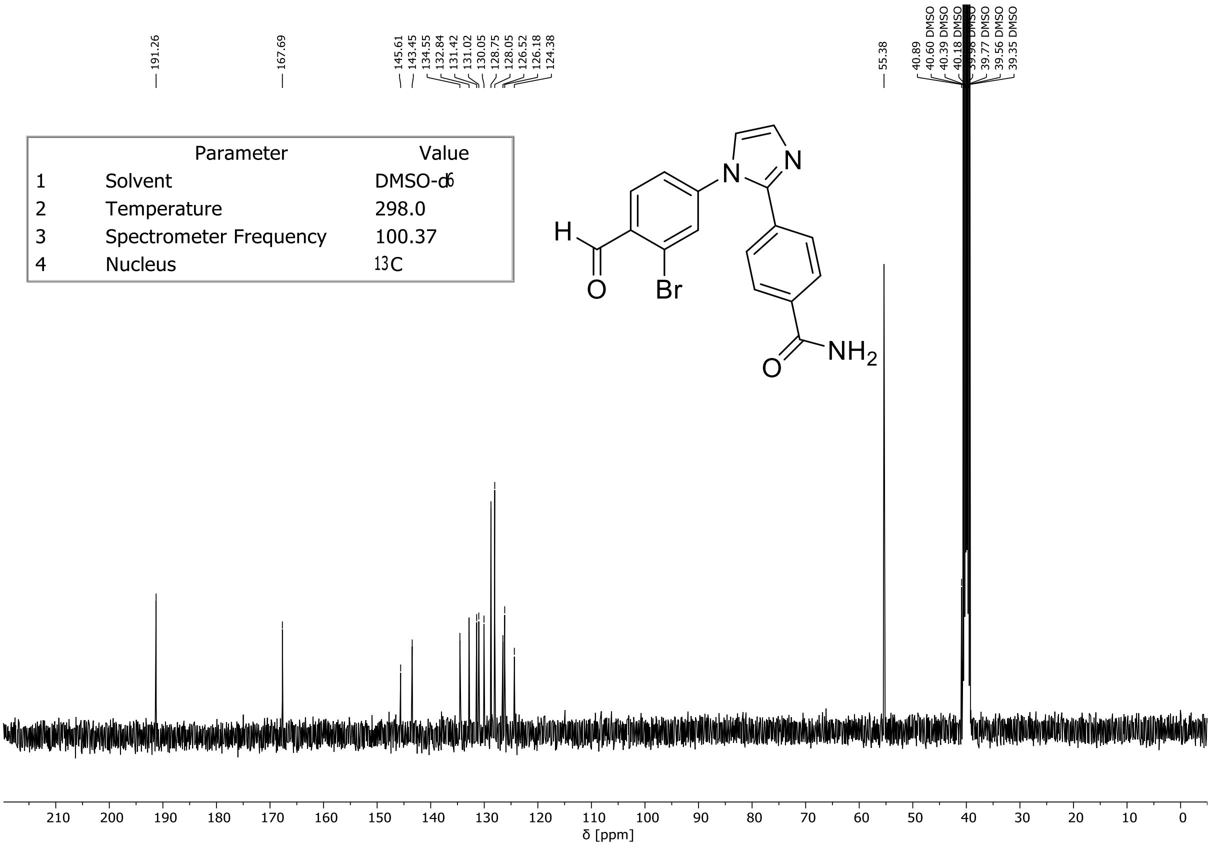

SI Figure S81: ^{1}H- and $^{13}C\{^{1}H\}$-NMR spectra of imidazole **17**.

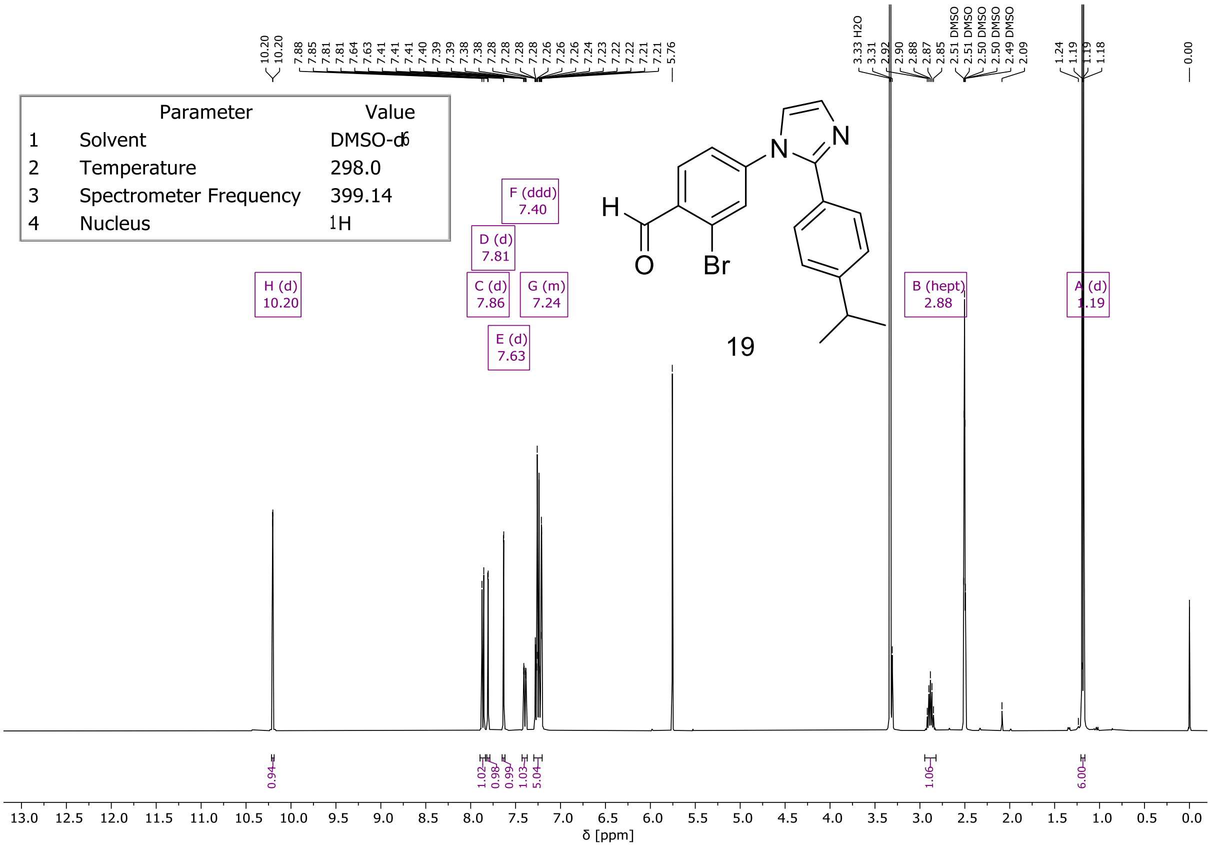

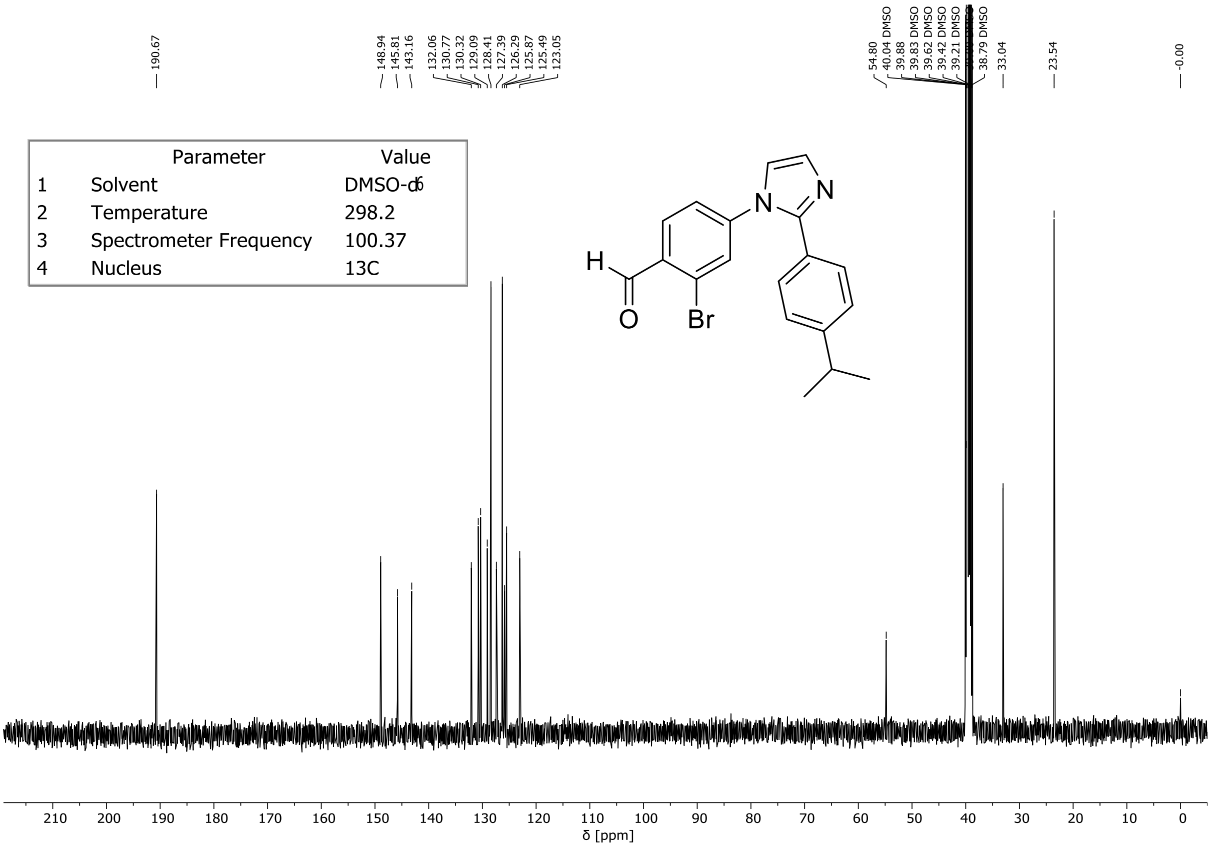

SI Figure S82: ^{1}H- and ^{13}C{^{1}H}-NMR spectra of imidazole **19**.

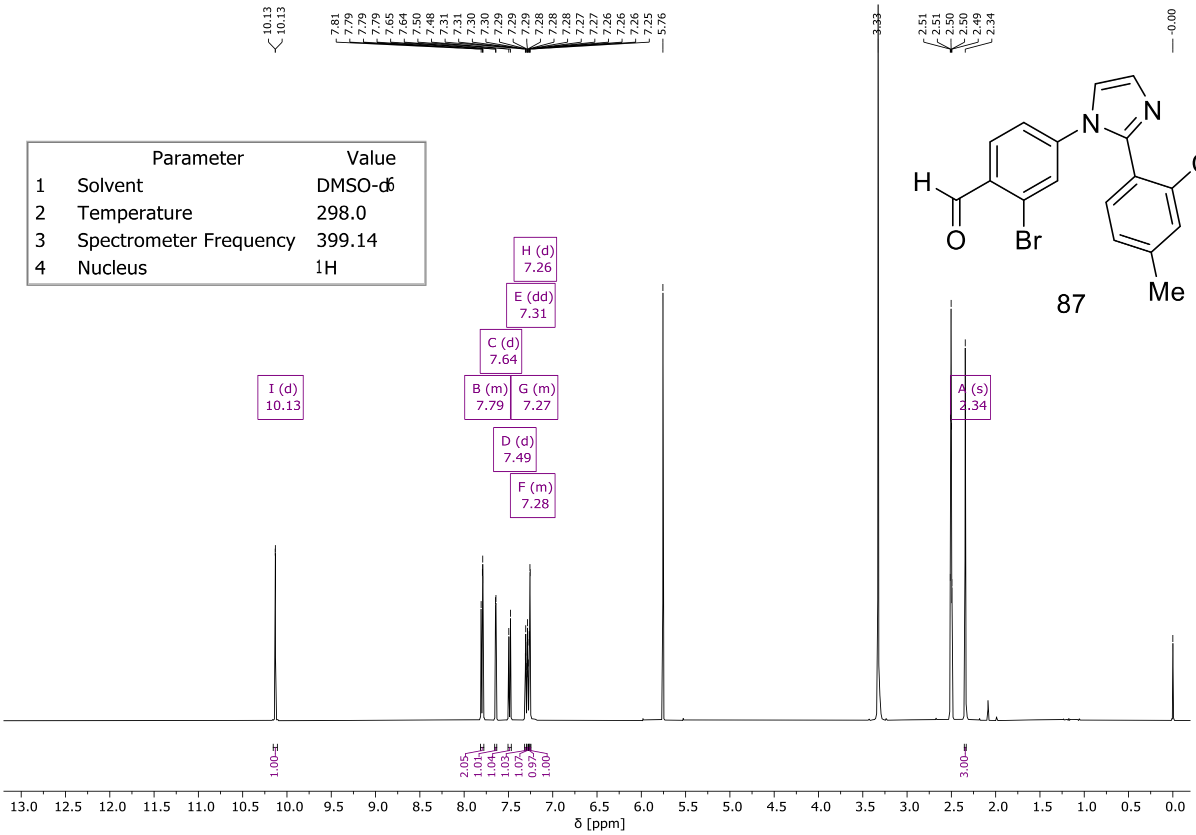

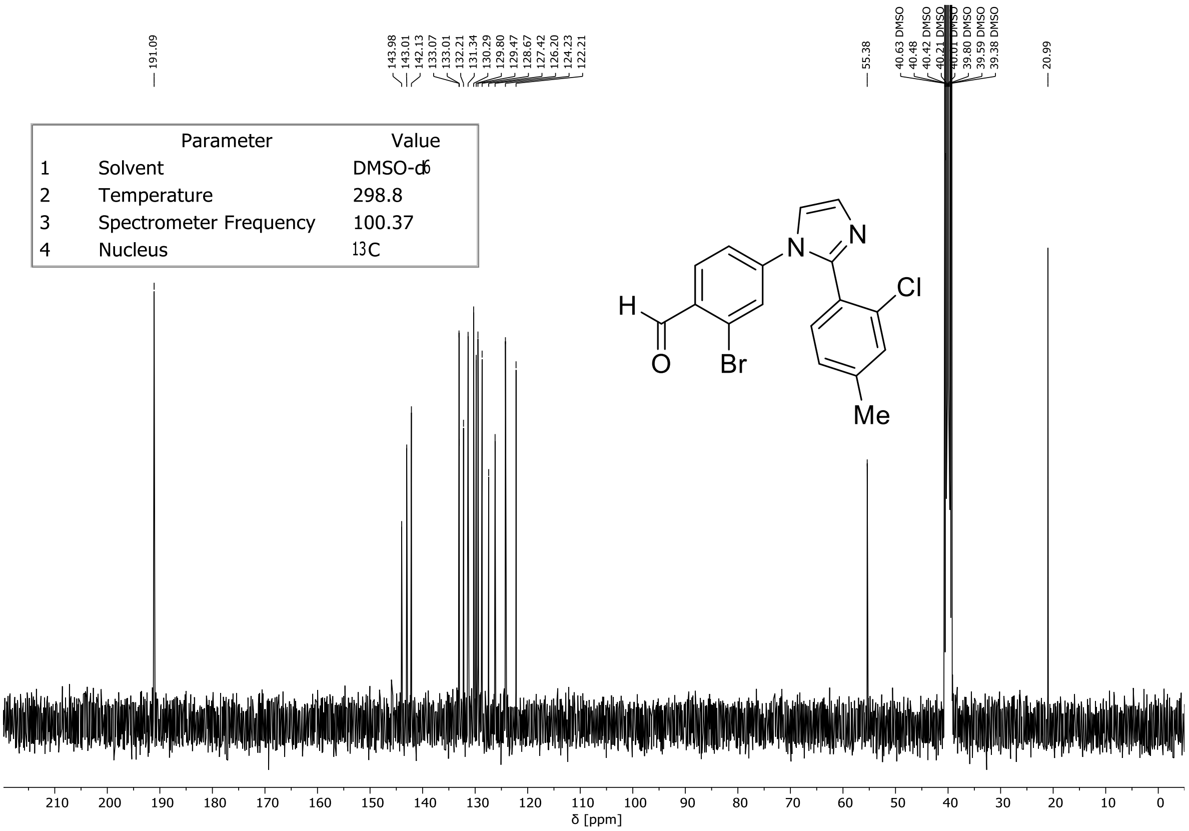

SI Figure S83: ^{1}H- and ^{13}C{^{1}H}-NMR spectra of imidazole **87**.

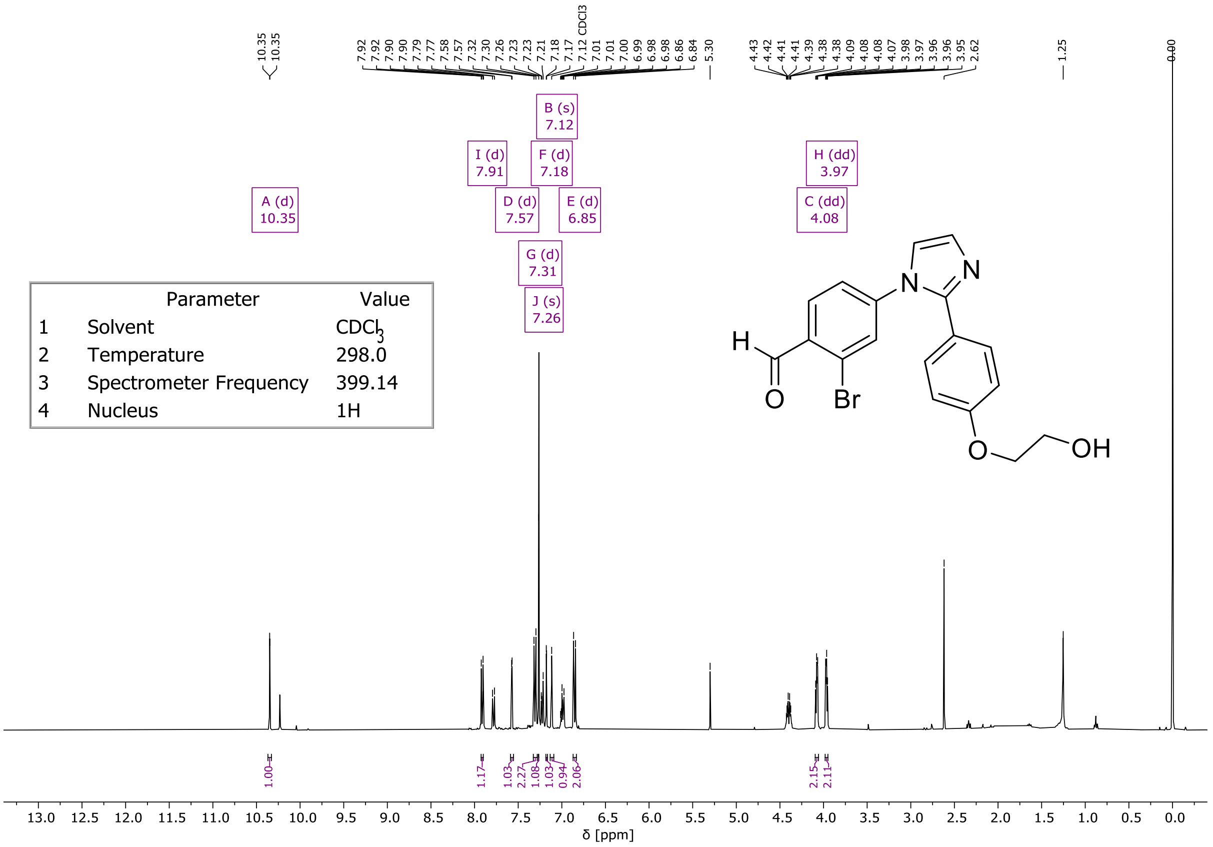

	Parameter	Value
1	Solvent	$CDCl_3$
2	Temperature	298.0
3	Spectrometer Frequency	399.14
4	Nucleus	1H

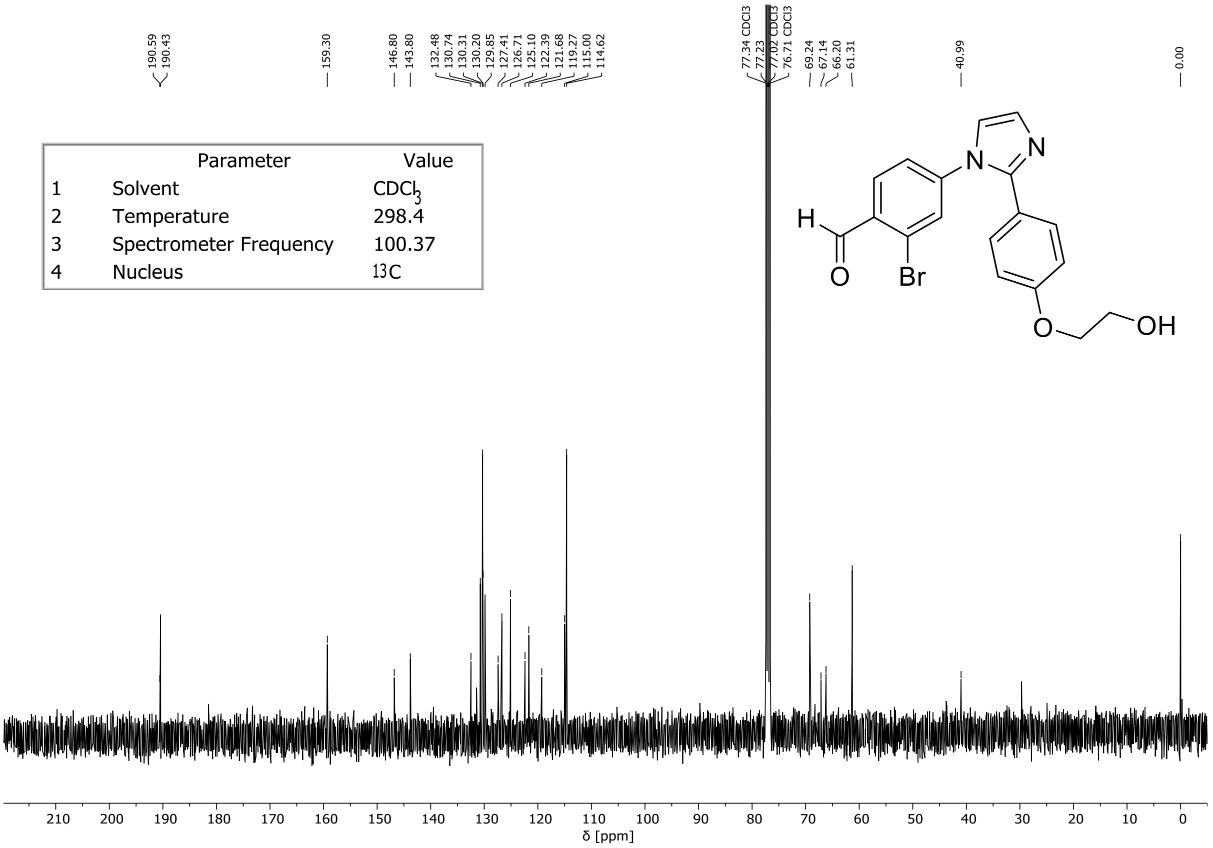

	Parameter	Value
1	Solvent	$CDCl_3$
2	Temperature	298.4
3	Spectrometer Frequency	100.37
4	Nucleus	13C

SI Figure S84: ^{1}H- and $^{13}C\{^{1}H\}$-NMR spectra of imidazole **24**.

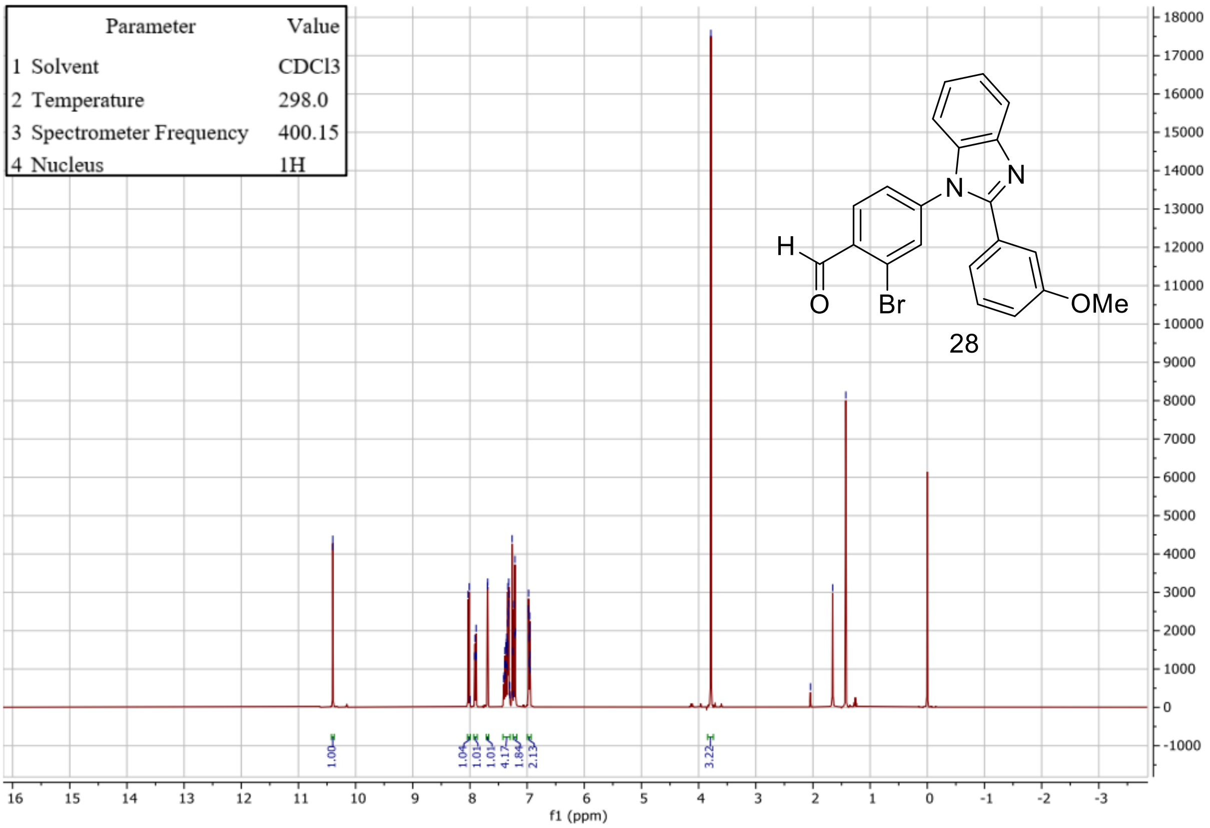

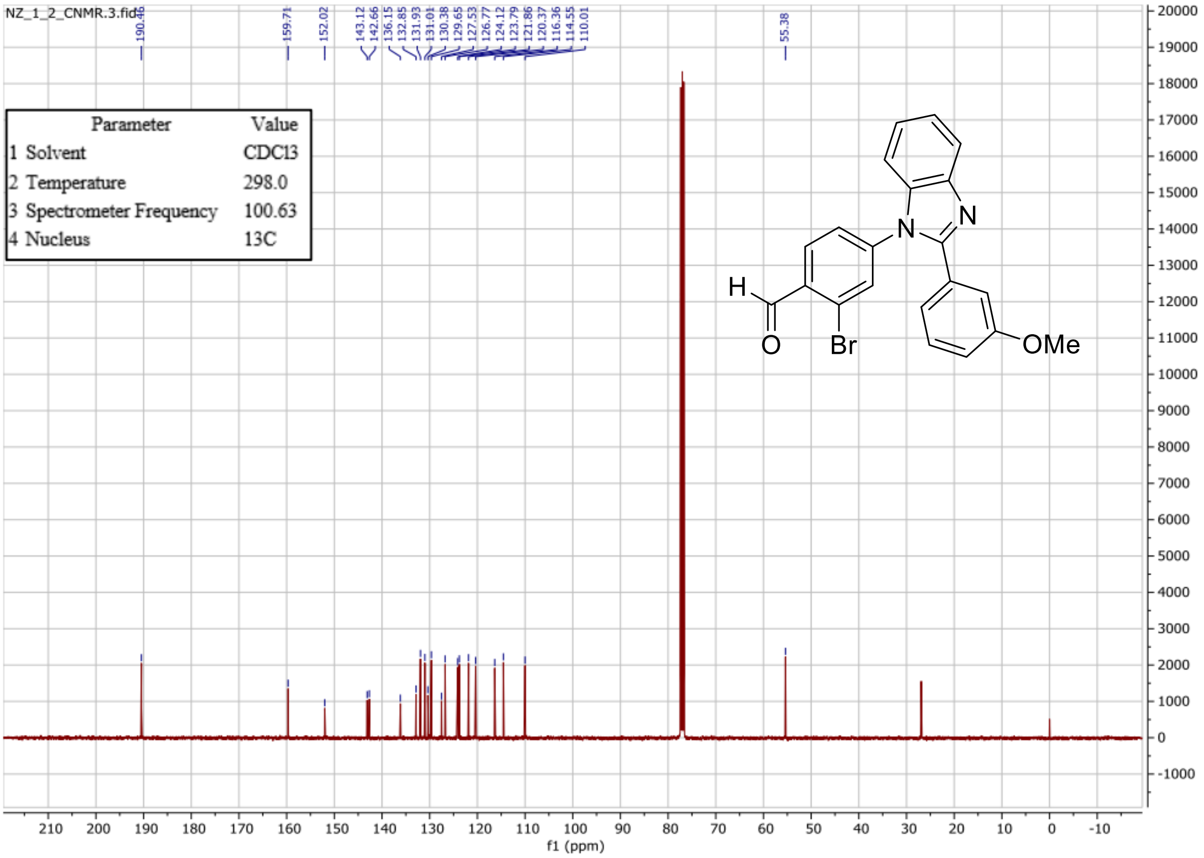

SI Figure S85: ^{1}H- and ^{13}C{^{1}H}-NMR spectra of imidazole **28.**

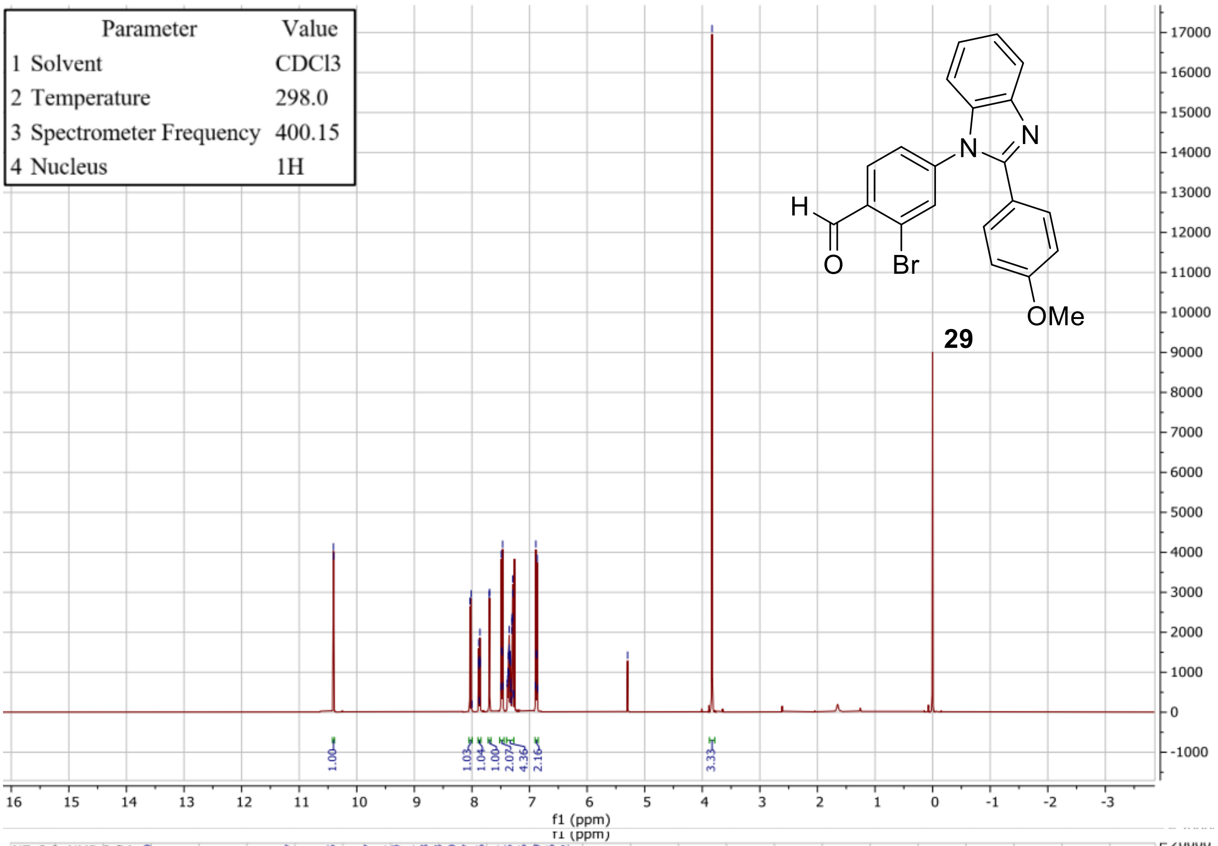

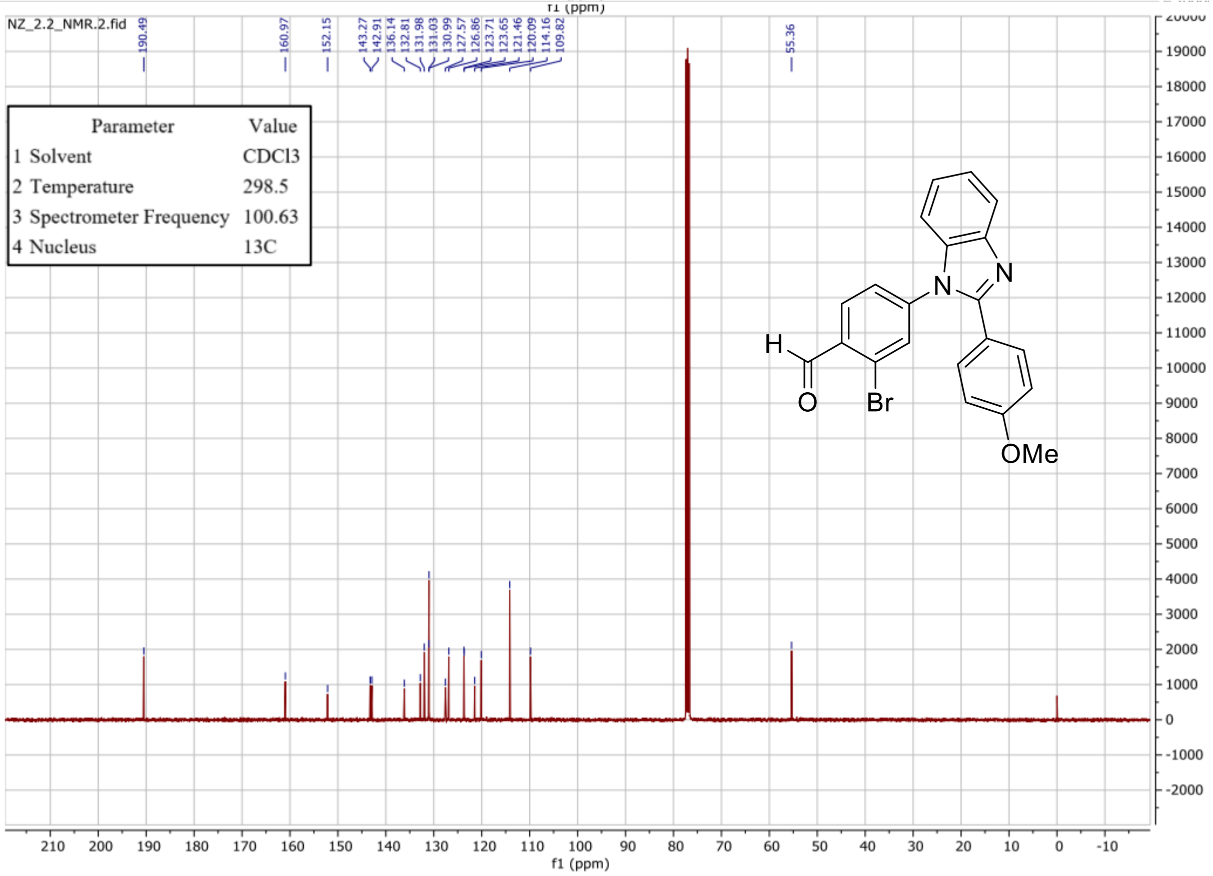

SI Figure S86: ^{1}H- and ^{13}C{^{1}H}-NMR spectra of imidazole **29.**

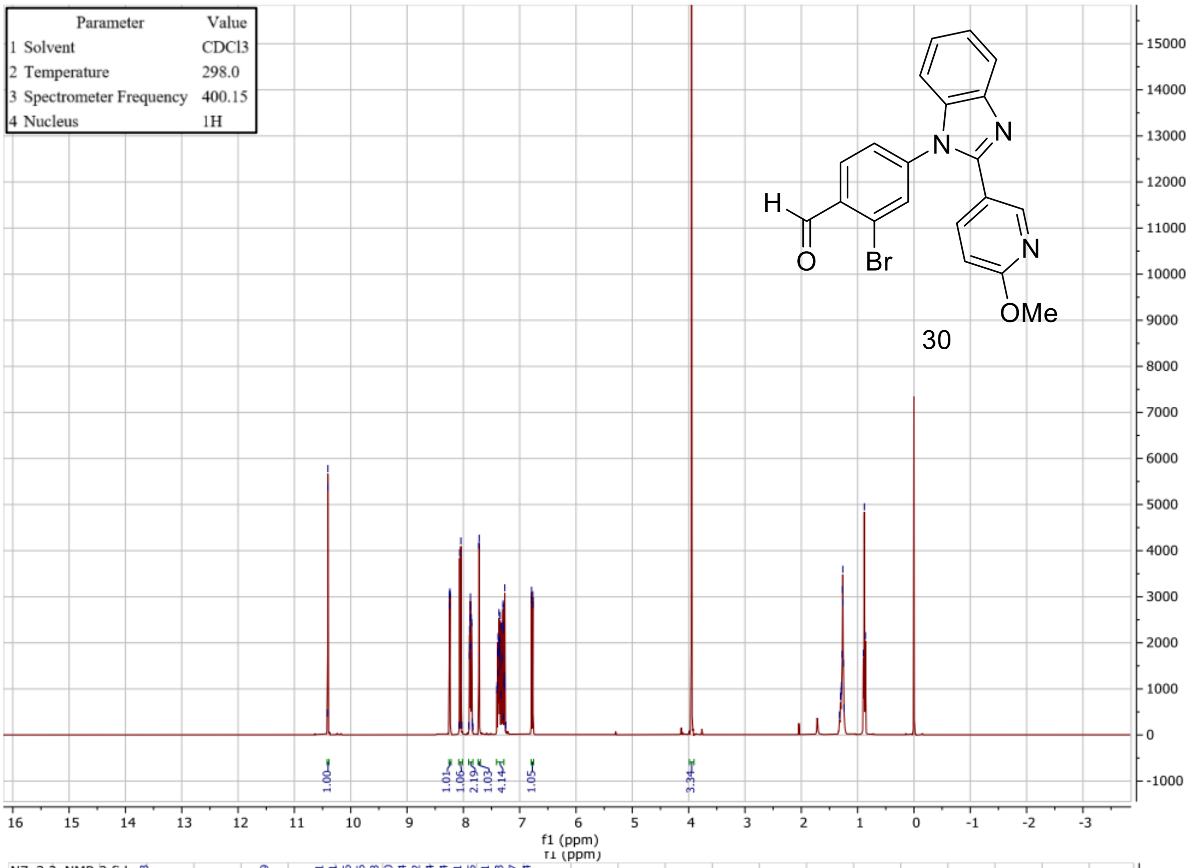

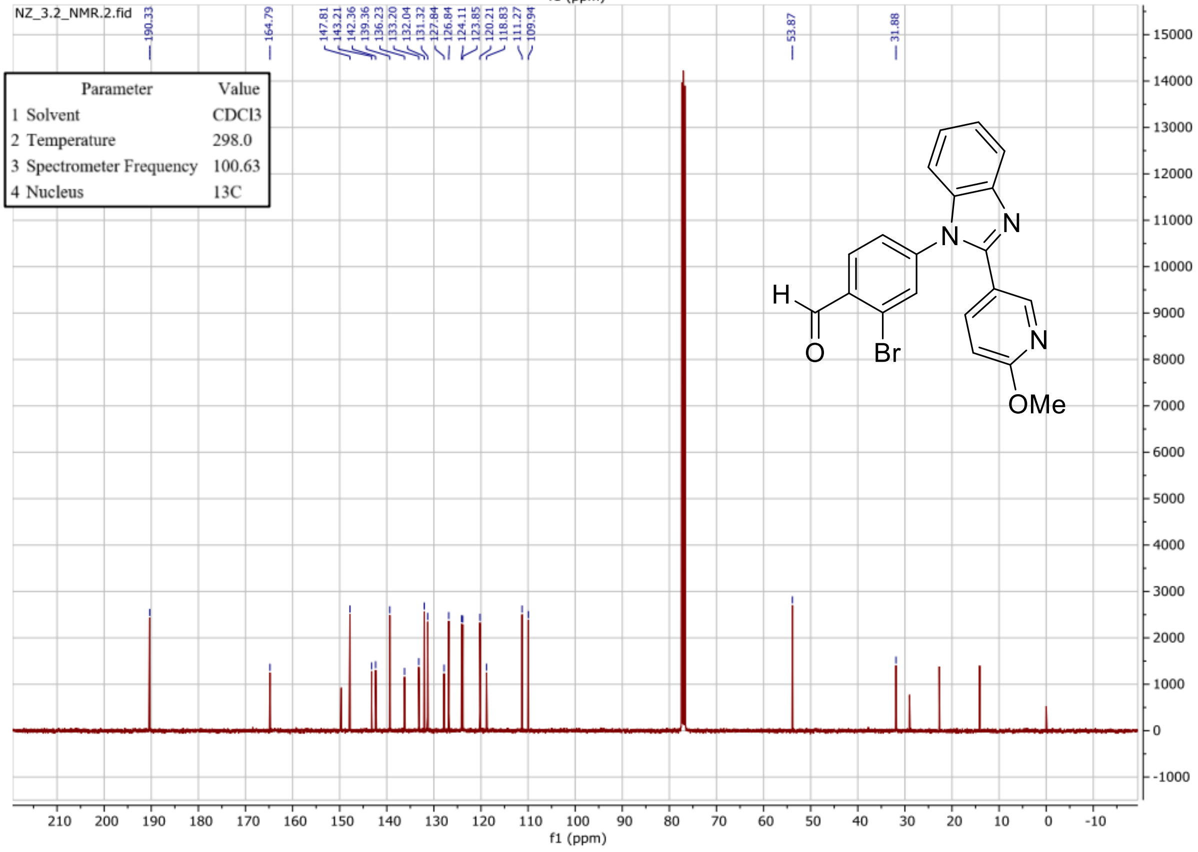

SI Figure S87: ^{1}H- and ^{13}C{^{1}H}-NMR spectra of imidazole **30**.

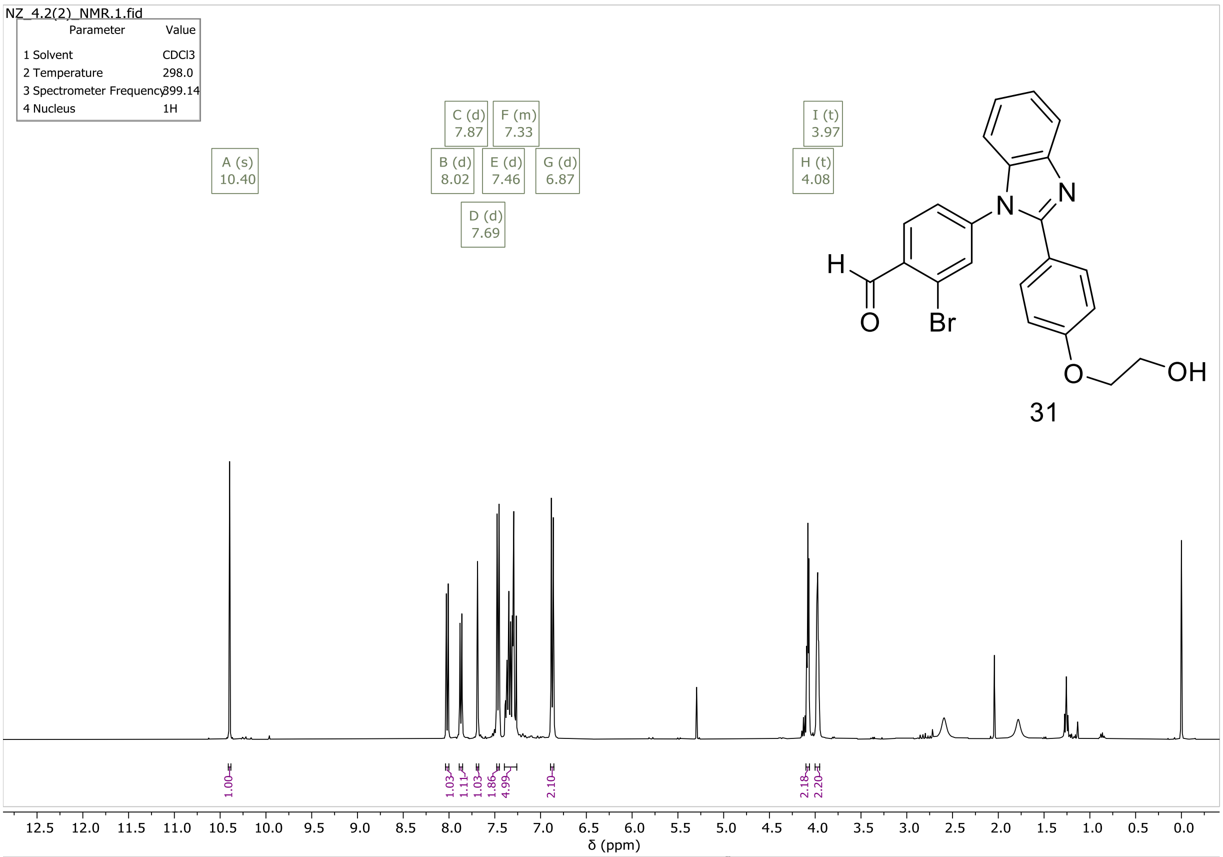

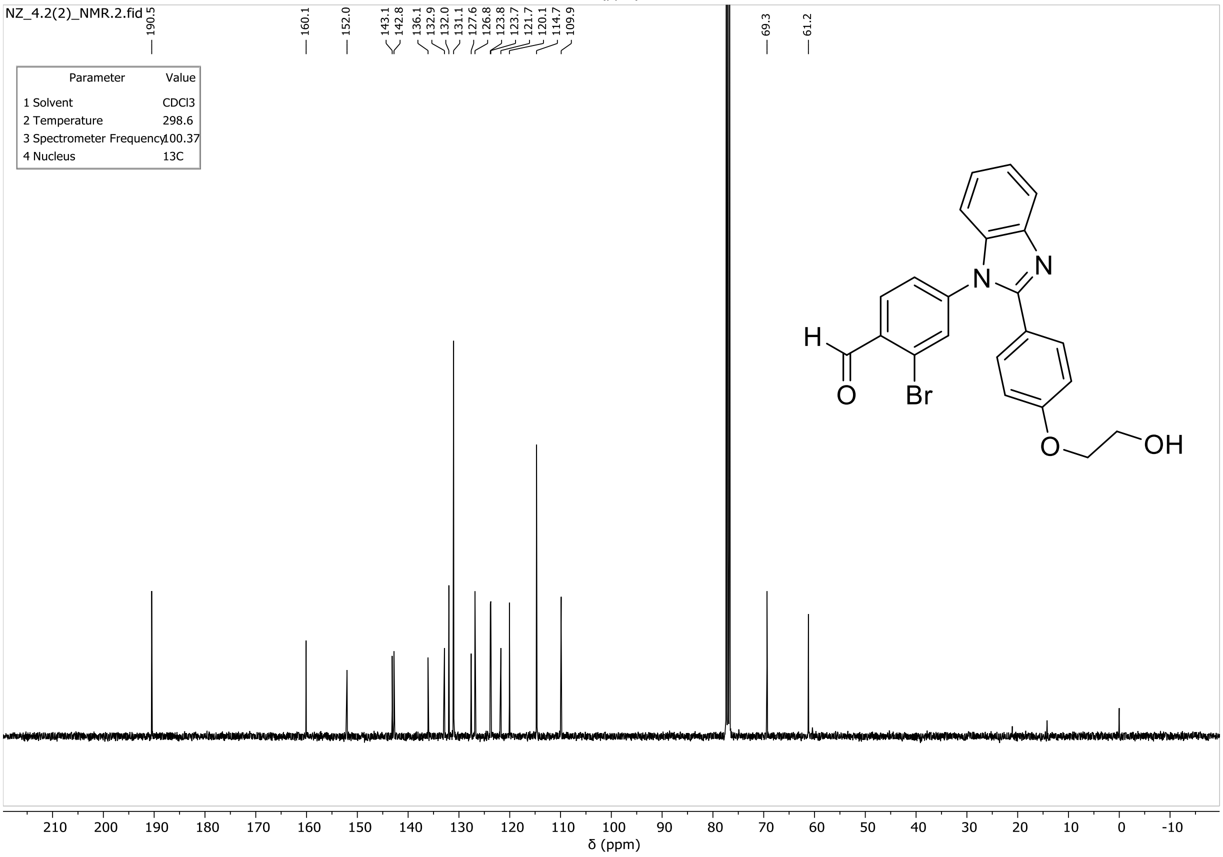

SI Figure S88: ^{1}H- and $^{13}C\{^{1}H\}$-NMR spectra of imidazole **31**.

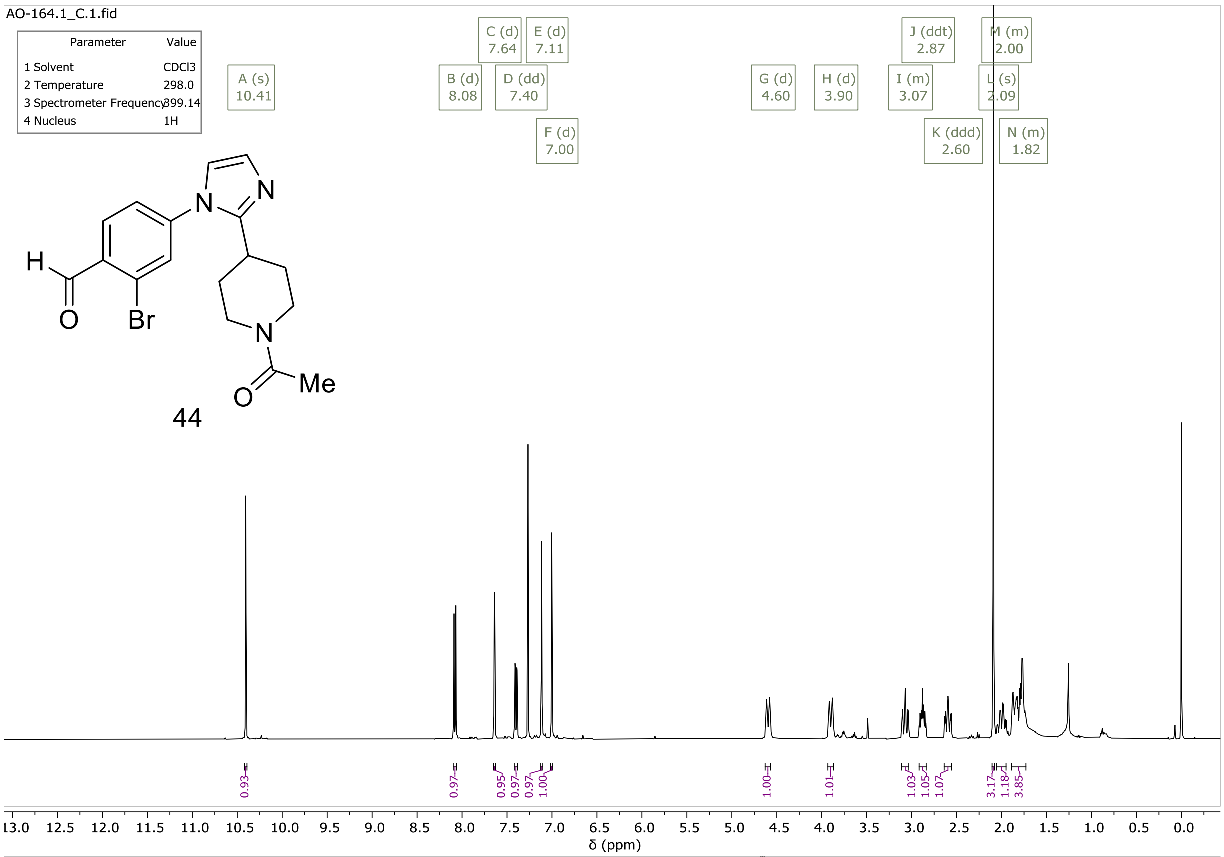

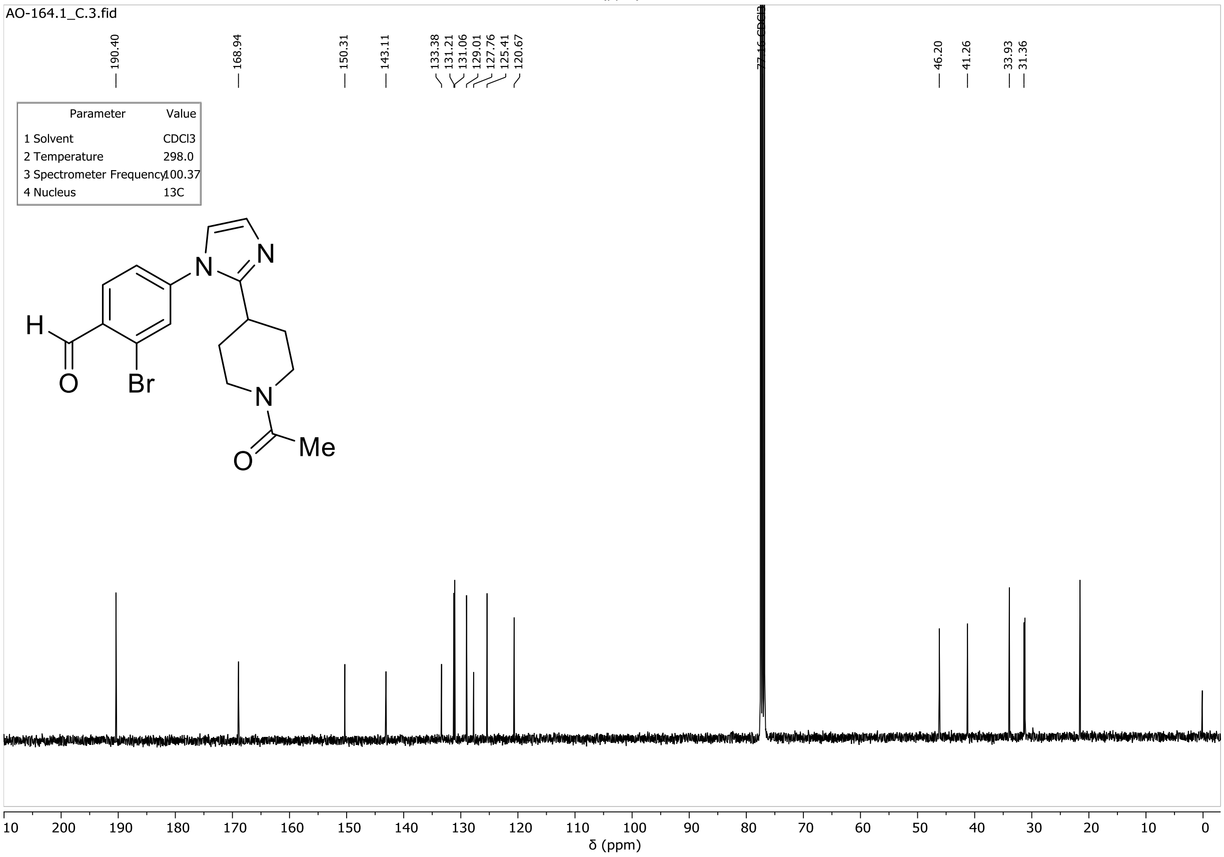

SI Figure S89: ^{1}H- and ^{13}C{^{1}H}-NMR spectra of imidazole **44.**

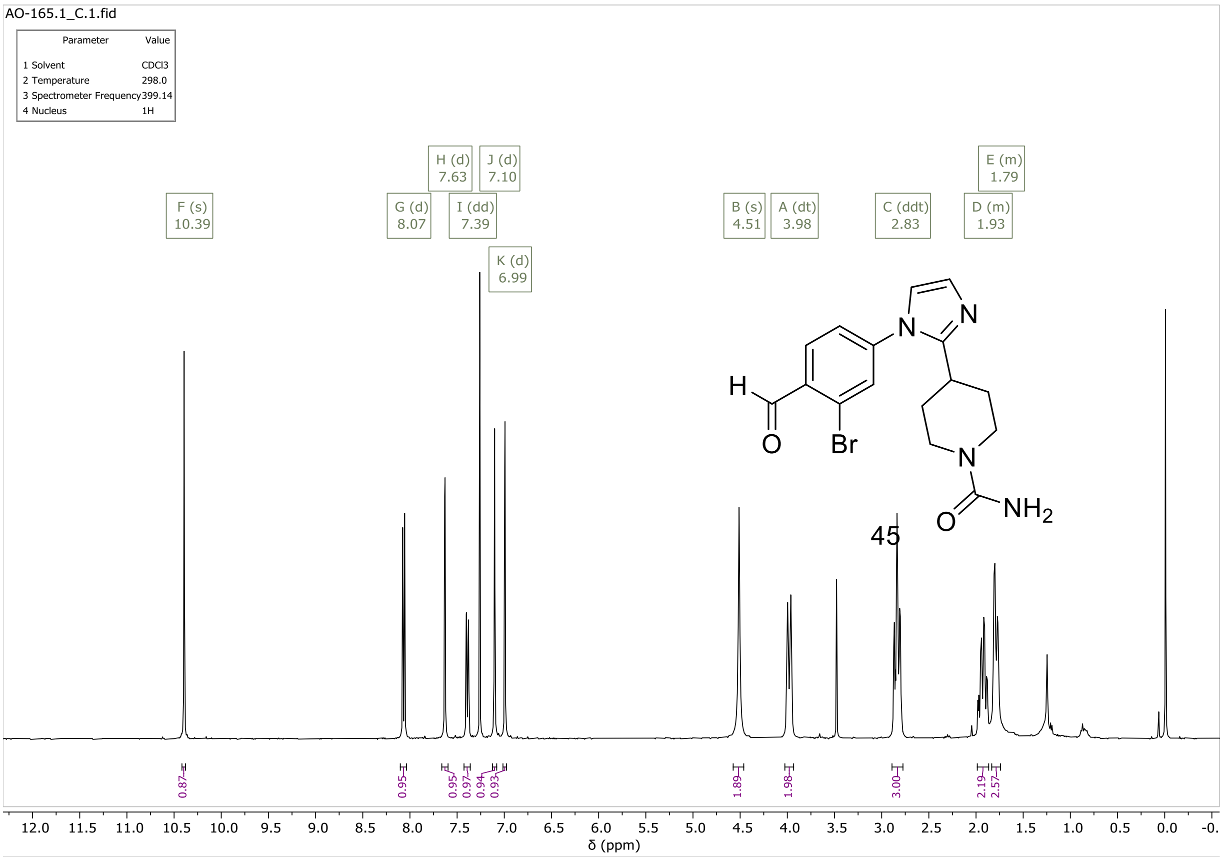

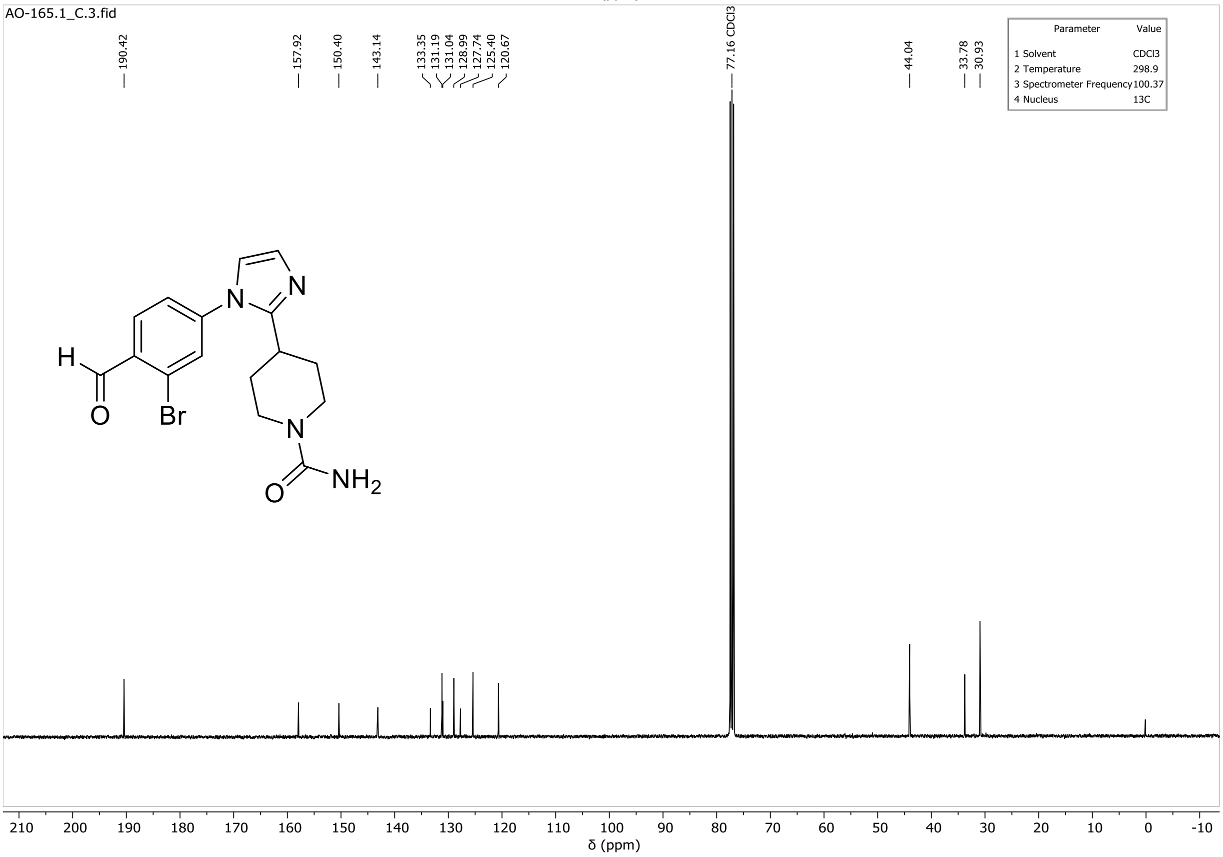

SI Figure S90: ^{1}H- and ^{13}C{^{1}H}-NMR spectra of imidazole **45.**

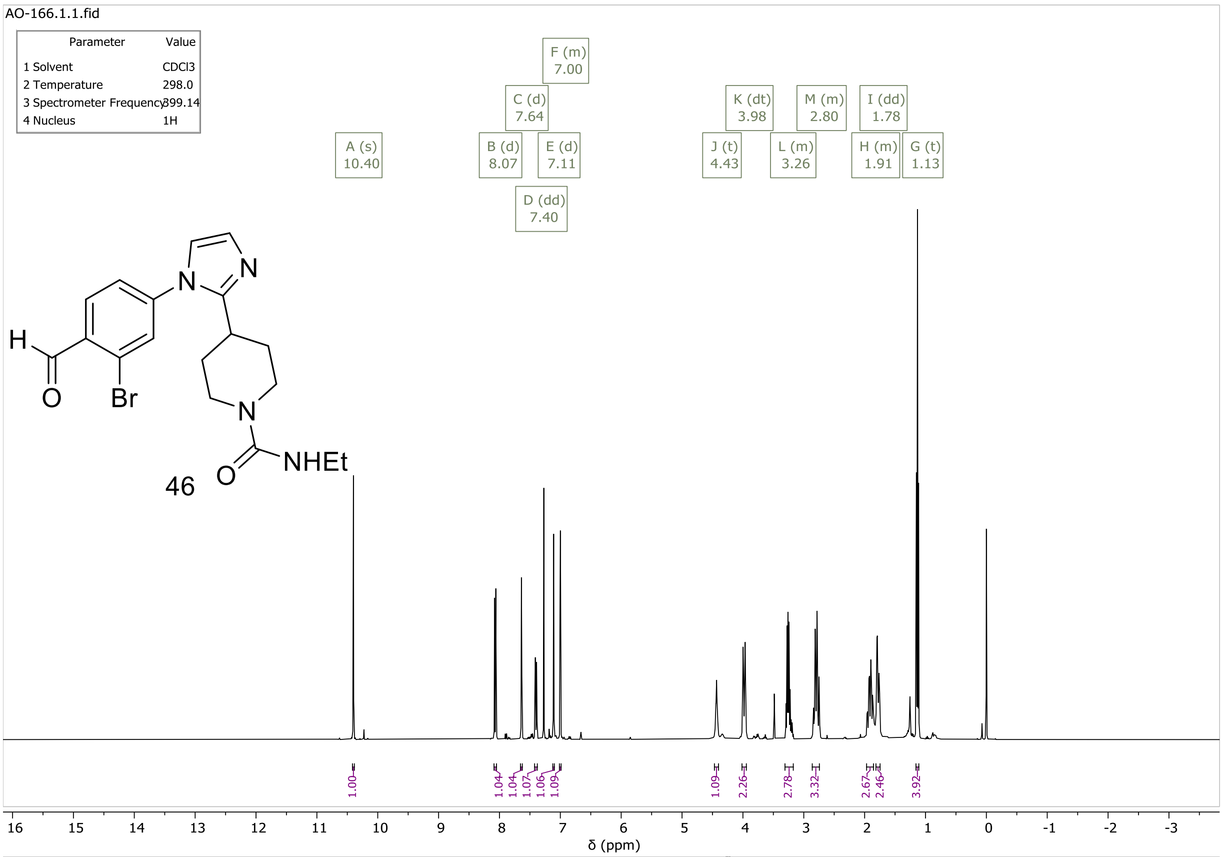

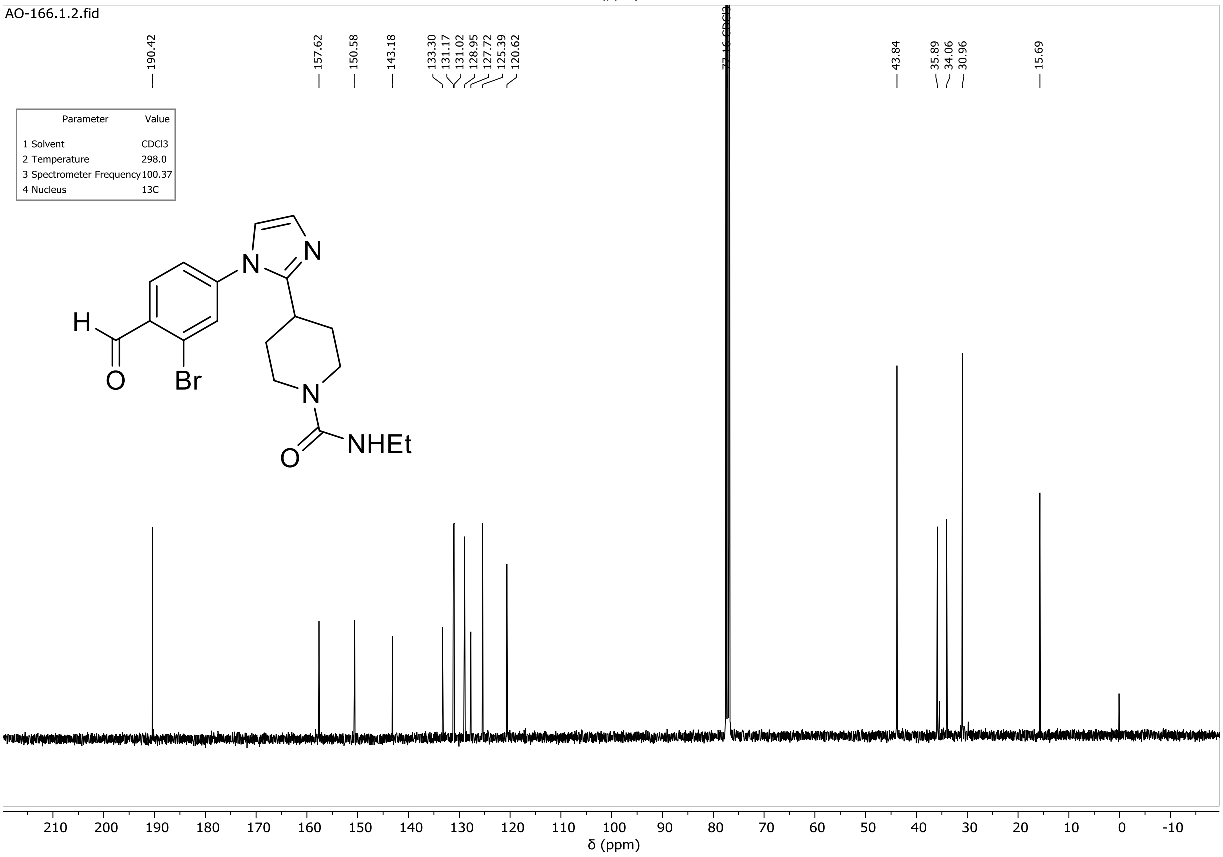

SI Figure S91: ^{1}H- and ^{13}C{^{1}H}-NMR spectra of imidazole **46.**

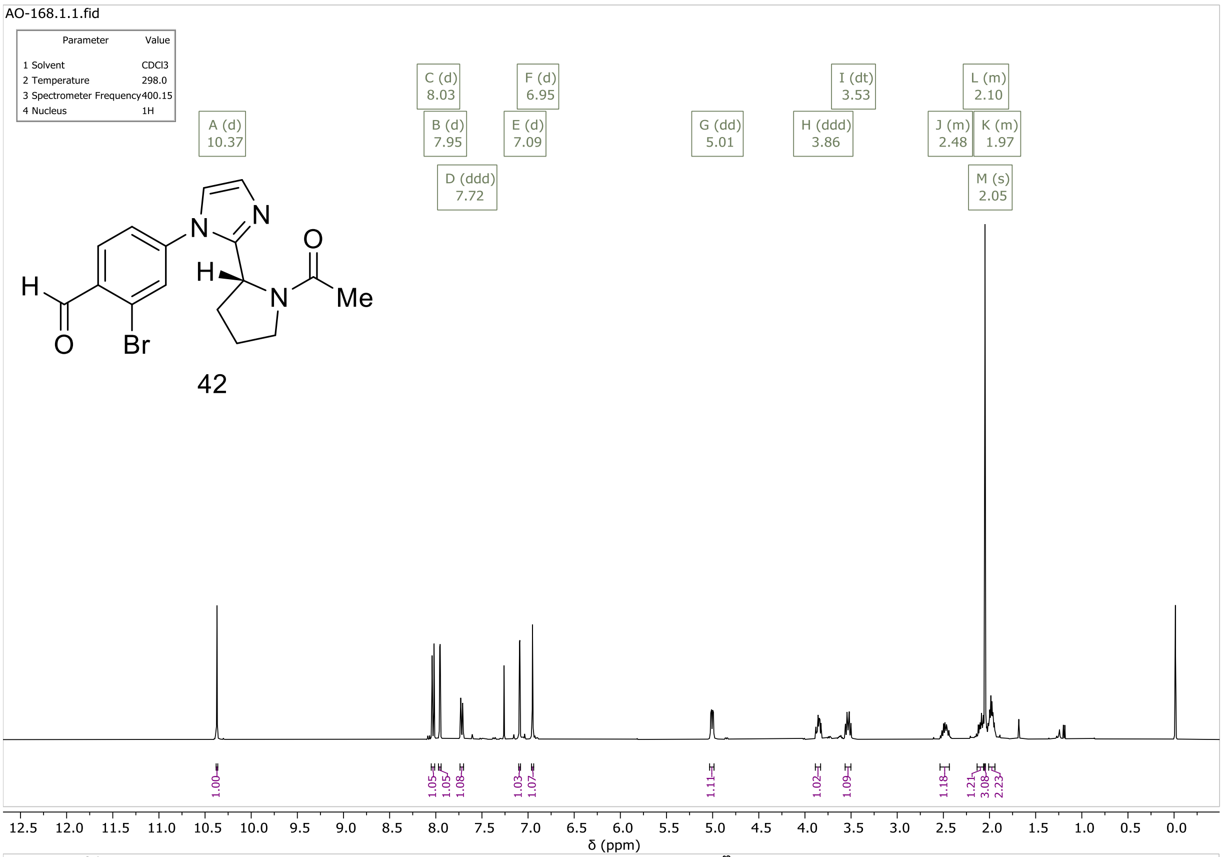

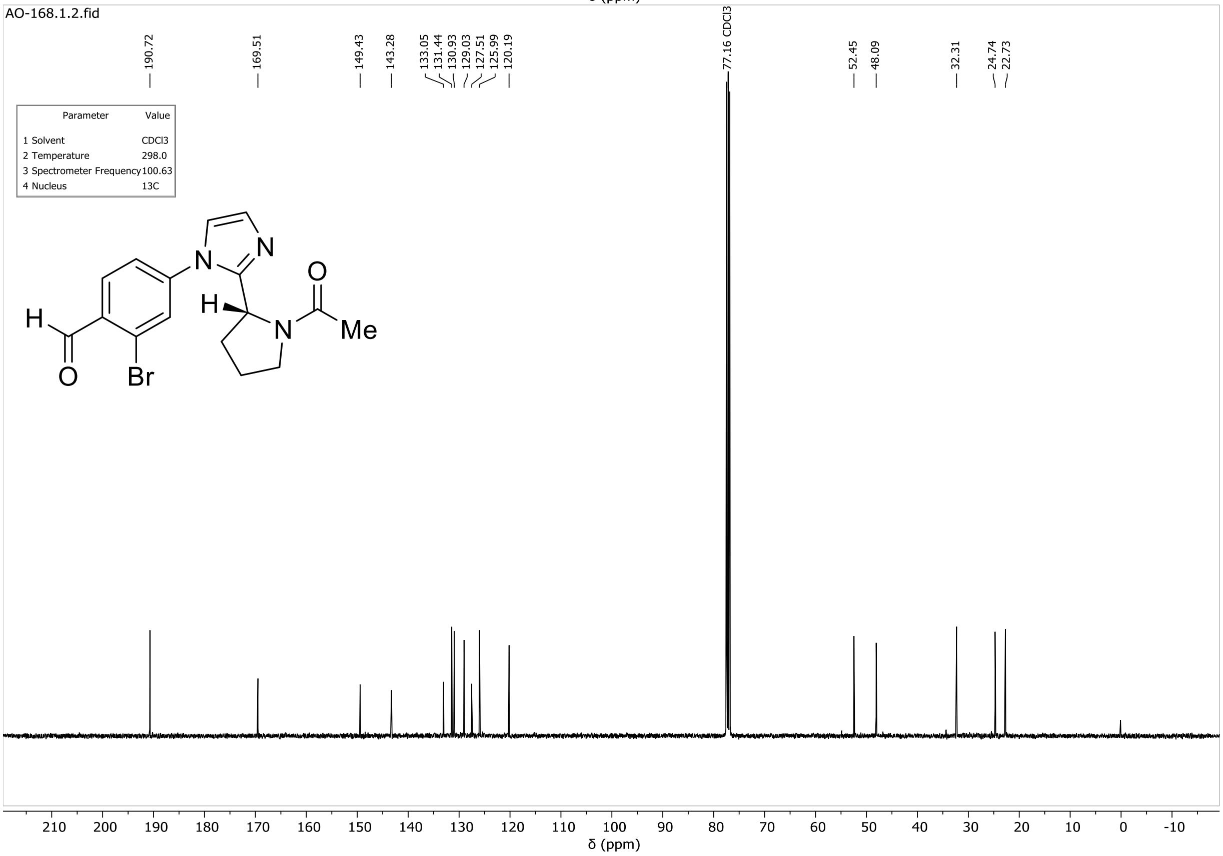

SI Figure S92: ^{1}H- and ^{13}C{^{1}H}-NMR spectra of imidazole **42**.

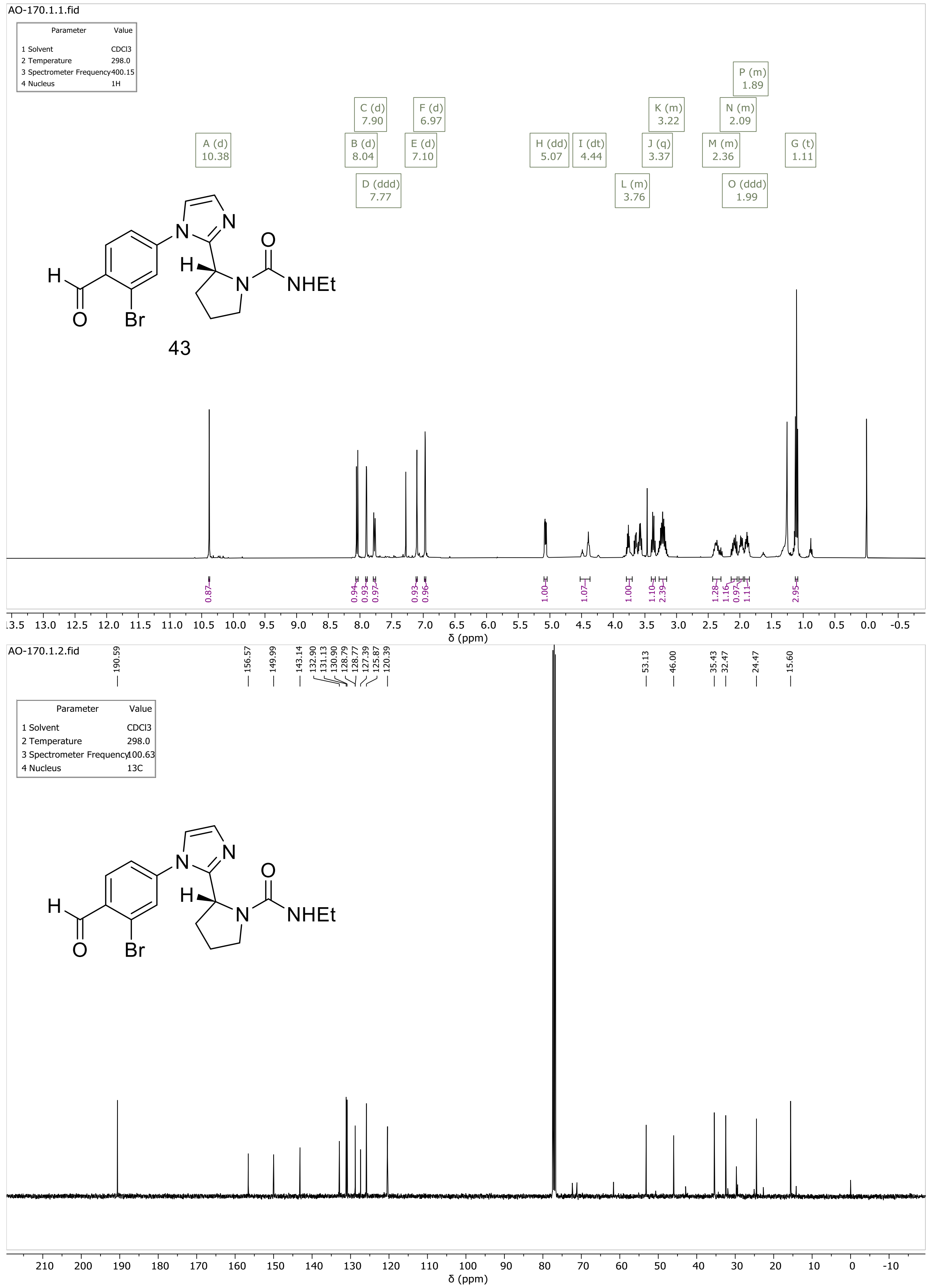

SI Figure S93: ^{1}H- and ^{13}C{^{1}H}-NMR spectra of imidazole **43.**

References

1. Hochmair, J. *et al.* 14-3-3zeta binding reduces phosphorylated Tau condensation, aggregation, and microtubule association. doi:10.1101/2024.03.15.585148.

2. Vonrhein, C. *et al.* Data processing and analysis with the autoPROC toolbox. *Acta Crystallogr D Biol Crystallogr* **67**, 293 (2011).

3. Potterton, L. *et al.* CCP4i2: the new graphical user interface to the CCP4 program suite. *Acta Crystallogr D Struct Biol* **74**, 68 (2018).

4. Evans, P. R. An introduction to data reduction: space-group determination, scaling and intensity statistics. *Acta Crystallogr D Biol Crystallogr* **67**, 282 (2011).

5. Evans, P. R. & Murshudov, G. N. How good are my data and what is the resolution? *Acta Crystallogr D Biol Crystallogr* **69**, 1204–1214 (2013).

6. Vagin, A. & Teplyakov, A. Molecular replacement with MOLREP. *Acta Crystallogr D Biol Crystallogr* **66**, 22–25 (2010).

7. Lebedev, A. A., Vagin, A. A. & Murshudov, G. N. Model preparation in MOLREP and examples of model improvement using X-ray data. *Acta Crystallogr D Biol Crystallogr* **64**, 33 (2008).

8. Emsley, P., Lohkamp, B., Scott, W. G. & Cowtan, K. Features and development of Coot. *Acta Crystallogr D Biol Crystallogr* **66**, 486–501 (2010).

9. Long, F. *et al.* AceDRG: A stereochemical description generator for ligands. *Acta Crystallogr D Struct Biol* **73**, 112–122 (2017).

10. Murshudov, G. N. *et al.* REFMAC5 for the refinement of macromolecular crystal structures. *Acta Crystallogr D Biol Crystallogr* **67**, 355–367 (2011).

11. Kovalevskiy, O., Nicholls, R. A., Long, F., Carlon, A. & Murshudov, G. N. Overview of refinement procedures within REFMAC 5: Utilizing data from different sources. *Acta Crystallogr D Struct Biol* **74**, 215–227 (2018).
